# Phylogenetic surveys on the newt genus *Tylototriton sensu lato* (Salamandridae, Caudata) reveal cryptic diversity and novel diversification promoted by historical climatic shifts

**DOI:** 10.7717/peerj.4384

**Published:** 2018-03-12

**Authors:** Bin Wang, Kanto Nishikawa, Masafumi Matsui, Truong Quang Nguyen, Feng Xie, Cheng Li, Janak Raj Khatiwada, Baowei Zhang, Dajie Gong, Yunming Mo, Gang Wei, Xiaohong Chen, Youhui Shen, Daode Yang, Rongchuan Xiong, Jianping Jiang

**Affiliations:** 1 CAS Key Laboratory of Mountain Ecological Restoration and Bioresource Utilization & Ecological Restoration and Biodiversity Conservation Key Laboratory of Sichuan Province, Chengdu Institute of Biology, Chinese Academy of Sciences, Chengdu, Sichuan, China; 2 Graduate School of Human and Environmental Studies, Kyoto University, Kyoto, Japan; 3 Institute of Ecology and Biological Resources, Hanoi, Vietnam; 4 Graduate University of Science and Technology, Vietnam Academy of Science and Technology, Hanoi, Vietnam; 5 College of Life Science, Anhui University, Hefei, China; 6 College of Life Science, Northwest Normal University, Lanzhou, China; 7 Natural History Museum of Guangxi, Nanning, China; 8 Biodiversity Conservation Key Laboratory, Guiyang College, Guiyang, China; 9 College of Life Science, Henan Normal University, Xinxiang, China; 10 College of Life Science, Hunan Normal University, Changsha, China; 11 Institute of Wildlife Conservation, Central South University of Forestry & Technology, Changsha, China; 12 Department of Life Science, Liupanshui Normal University, Liupanshui, China

**Keywords:** Cryptic diversity, Radiation, Diversification rate, Climate shifts, Tibetan plateau, *Tylototriton*

## Abstract

Global climatic transitions and Tibetan Plateau uplifts are hypothesized to have profoundly impacted biodiversity in southeastern Asia. To further test the hypotheses related to the impacts of these incidents, we investigated the diversification patterns of the newt genus *Tylototriton sensu lato*, distributed across the mountain ranges of southeastern Asia. Gene-tree and species-tree analyses of two mitochondrial genes and two nuclear genes revealed five major clades in the genus, and suggested several cryptic species. Dating estimates suggested that the genus originated in the early-to-middle Miocene. Under different species delimitating scenarios, diversification analyses with birth-death likelihood tests indicated that the genus held a higher diversification rate in the late Miocene-to-Pliocene era than that in the Pleistocene. Ancestral area reconstructions indicated that the genus originated from the northern Indochina Peninsula. Accordingly, we hypothesized that the Miocene Climatic Transition triggered the diversification of the genus, and the reinforcement of East Asian monsoons associated with the stepwise uplifts of the Tibetan Plateau promoted the radiation of the genus in southeastern Asia during the Miocene-to-Pliocene period. Quaternary glacial cycles likely had limited effects on speciation events in the genus, but mainly had contributions on their intraspecific differentiations.

## Introduction

Southeastern Asia contains several biodiversity-hotspots (Myers et al., 2000). Processes promoting origin of biodiversity in this region have been explained by several mechanisms, including vicariances due to alterations of biogeographical configurations (Peng et al., 2006; Zhang et al., 2010), rapid radiations (Liu et al., 2006; Sun et al., 2012; Wang et al., 2012a; Wang et al., 2012b; Kennedy et al., 2012) and diversification rate shifts (Leneveu, Chichvarkhin & Wahlberg, 2009; Mao et al., 2010; Yu et al., 2014) associated with the growths of the Tibetan Plateau.

Despite arguments on timing and patterns of the uplifts of the Tibetan Plateau and adjacent regions (Harrison et al., 1992; Li & Fang, 1999; An et al., 2001; Sun & Wang, 2005; Molnar, Boos & Battisti, 2010; Yao et al., 2011), this process was unequivocally estimated to have occurred within the late Tertiary, and episodes of uplifts probably continued throughout the Pliocene and even Quaternary (Wan et al., 2007; Favre et al., 2014). The uplifts of the Tibetan Plateau had reinforced the East Asian monsoons (EAMs) several times, such as that about ∼15 Mya and ∼8 Mya (An et al., 2001; Wan et al., 2007; Yao et al., 2011). These incidents were proposed to have been promoted high levels of biodiversity transformations and radiations which created the present extraordinary high biodiversity in this region (Favre et al., 2014; Klaus et al., 2016).

Quaternary climatic oscillations have been singled out as another kind of historical forces in shaping species distributions and modulating local diversification (Hewitt, 2000). The effects of Pleistocene glaciations on promoting speciation, however, remain controversial. The refugium hypothesis (Haffer, 1969) indicates that speciation rates have increased because of vicariances among glacial refugia (Knowles, 2001; Knowles & Richards, 2005). On the contrary, the model of decreased diversification rates as a result of the increased extinction rates as related to the Pleistocene glaciations was postulated (Coope, 2004; Zink, Klicka & Barber, 2004). Additionally, the pattern with a constant diversification rate was proposed (Zink & Klicka, 2006) because diversification rates were also likely to be influenced by biotic factors (Thomas et al., 2010), ecological features (Weir & Schluter, 2007) or random episodes (Blount, Borland & Lenski, 2008). Yet in southeastern Asia, a number of evolutionary studies mainly reflect the contributions of the Pleistocene glacial cycles on the intraspecific lineage heterogeneity and revealed many local refugia for lineages especially in isolated mountains (e.g., Ding et al., 2011; Wang et al., 2012a; Wang et al., 2012b; Wang et al., 2013; Gu et al., 2013; Lin et al., 2014).

To explore diversification dynamics of a species group, inclusion of a high ratio of the total number of natural “true” species is the most important factor (Cusimano, Stadler & Renner, 2012). However, species diversity and taxonomy are still poorly known in many regions (e.g., the tropical region; Dubey & Shine, 2012). Taxonomic uncertainty may be due to inadequate biodiversity inventories, limited data to delimit taxonomic units and inconsistent criteria for species delimitation (Renner & Cusimano, 2010; Dubey & Shine, 2012). Many areas in southeastern Asia have not been thoroughly explored especially in the high-endemism-diversity regions, such as Himalayas, Hengduan Mountains and the tropical region of Indochina. In these areas, recently, a large number of new amphibian species were described (see Frost, 2016). Moreover, in many presumably well-described groups, molecular data indicated that many nominal species are not monophyletic (e.g., *Tylototriton asperrimus* complex; Yuan et al., 2011), and taxonomic reclassifications are needed. Even in a complete biodiversity inventory, different species concepts would produce different sets of taxonomic units (e.g., the *Gynandropaa* complex; Zhang et al., 2010; Fei, Ye & Jiang, 2012). Obviously, taxonomic uncertainty may influence the reconstructions of diversification patterns. Therefore, we need to examine the role of taxonomic uncertainty on exploring diversification patterns.

The newt genus *Tylototriton sensu lato* (*s.l.*) Anderson, 1871 (Salamandridae, Caudata) currently contains 24 nominal species (Fei, Ye & Jiang, 2012; Zhao et al., 2012; Frost, 2016), and is distributed across southeastern Asia. *Tylototriton s.l.* was supported as a monophyletic group in previous phylogenetic studies (e.g., Nishikawa et al., 2013; Nishikawa, Matsui & Nguyen, 2013). But based on morphology, this group has ever been divided into three genera, i.e., *Tylototriton sensu stricto*, *Yaotriton* and *Liangshantriton* (Fei, Ye & Jiang, 2012). Twelve *Tylototriton s.l.* species have been described in the past five years (Zhao et al., 2012; Shen, Jiang & Mo, 2012; Hou, Li & Lu, 2012; Nishikawa et al., 2013; Nishikawa, Matsui & Nguyen, 2013; Nishikawa, Matsui & Rao, 2014; Yang et al., 2014; Khatiwada et al., 2015; Phimmachak, Aowphol & Stuart, 2015). But within all nominal species, *T. daweishanensis* was argued to be synonym of *T. yangi* (Nishikawa et al., 2015), and *T. shanjing* was argued to be synonym of *T. verrucosus* (Zhang et al., 2007). These inconsistencies indicate that deep phylogenetic investigations in the group were required to investigate the relationships within *Tylototriton* to resolve species relationships and uncover any cryptic diversity within this group using multiple genes. Additionally, most *Tylototriton s.l.* species are found to be distributed allopatrically throughout the Asian monsoon climate zone (Frost, 2016), with a preference for humid environments, and usually inhabit and breed near or in ponds in the forest because many of them have limited capability on dispersal capability (Fei, Ye & Jiang, 2012). Accordingly, it is presumed that their diversification may have been easily impacted by historical climatic changes. Therefore, this genus is an ideal model to examine the effects of past climatic shifts on the diversification in southeastern Asia.

We hypothesize that the past climatic changes, especially the reinforcement of the East Asian monsoons (EAMs) associated with the stepwise uplifts of the Tibetan Plateau during the late Tertiary and Quaternary glacial oscillations, drove the speciation diversification of *Tylototriton s.l.*. Thus, in this study, to test the hypotheses, based on the sampling of all 24 nominal *Tylototriton s.l.* species and molecular data of mitochondrial and nuclear genes, we reconstruct species phylogenetic relationships and infer a time frame and diversification patterns for the *Tylototriton s.l.* newts.

## Materials and Methods

### Sampling and sequencing

**Figure 1:**
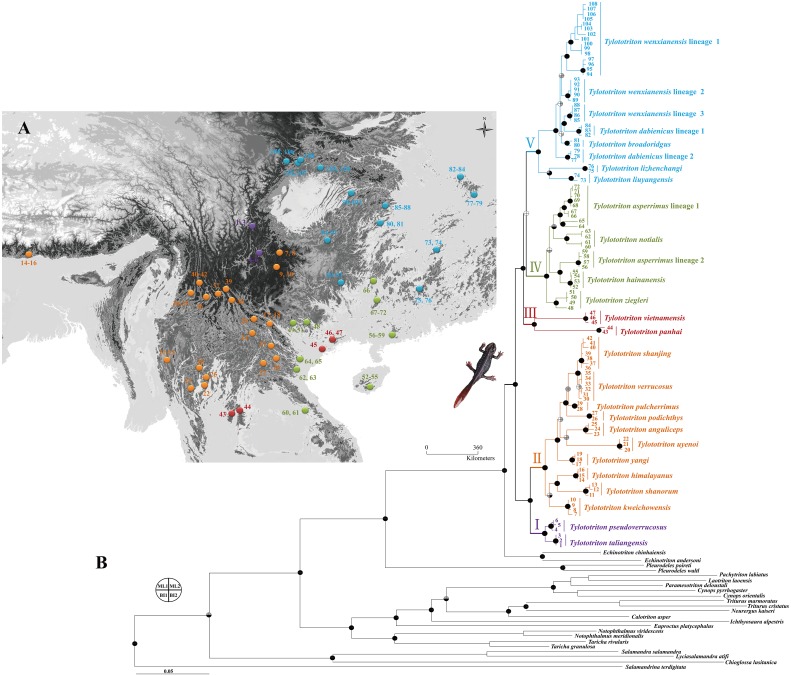
Sampling localities (A) and Maximum likelihood (ML) tree obtained based on mtDNA data of *Tylototriton s.l.* and relatives (B). Sample number 1–108 refer to Table S1. Five clades (I, II, III, IV and V) of the genus were denoted as different colors. Bootstrap supports (bs) resulted from ML analyses and posterior probability (pp) resulted from Bayesian inference (BI) method were labeled on major nodes. Node supports ML1 and BI1 were resulted from analyses on mtDNA data, while ML2 and BI2 were from analyses on four-gene concatenated data. Black: bs > 70% or pp > 0.95, grey: bs = 50–70% or pp = 0.85–0.95, white: bs < 50% and pp < 0.85.

A total of 108 samples was used, representing all 23 recognized species (we did not use “*T. daweishanensis*” because it is assigned as synonym of *T. yangi*) of *Tylototriton s.l.*, which were collected from 49 localities scattering across the range of the genus in the southeastern Asia (Fig. 1A; Table S1). Based on previous studies (Steinfartz et al., 2007; Zhang et al., 2008), 23 species representing major divisions within Salamandridae were included in our phylogenetic analyses, and one *Ambystoma mexicanum* was used as outgroup (Table S1). In these 23 Salamandridae species, two specimens of *Echinotriton chinhaiensis* (Zhang et al., 2008) were collected in this study. The Animal Care and Use Committee of Chengdu Institute of Biology, CAS provided full approval for this purely observational research (Number: CIB2010031015). Field experiments were approved by the Management Office of the Kuankuoshui Nature Reserve (number: KKSNR201204002) and the Management Office of the Dabashan Nature Reserve (number: DBSNR201204002).

The genomic DNA was extracted with the Qiagen DNeasy tissue kits (Qiagen, Hilden, Germany) from tissues preserved in 95% alcohol. Fragments of two mitochondrial genes, i.e., *16S* and *ND2*, and two nuclear genes, *NCX1* and *BDNF*, were amplified for all samples collected in this work. Primers for amplifying *ND2*, *NCX1* and *BDNF* genes were designed in this study (Table S2). The polymerase chain reaction (PCR) cycles included an initial denaturation step of 4 min at 94 °C and 35 cycles of denaturation for 30 s at 94 °C, primer annealing for 30 s at 48–58 °C, and extension for 1 min 30 s at 72 °C. Sequencing was performed in both directions using the primers in PCR on ABI3730 sequencer. New sequences were deposited in GenBank (accession numbers were shown in Table S1). *ND2* sequences of 28 specimens were retrieved from GenBank (see Table S1), and *ND2* sequences of two *T. yangi* samples were from Zhao et al. (2012).

Alignments were performed in the program MAFFT v.6 (Katoh et al., 2002) with default options. To avoid artificial bias in refining alignments, GBLOCKS v.0.91.b (Castresana, 2000) with default settings was used to extract regions of defined sequence conservation from the length-variable *16S* fragment. DNA sequences of each protein-coding gene were translated into amino acid sequences, and then the amino acid sequences of each gene were aligned in MEGA v.6 (Tamura et al., 2013). The nucleotide gaps were determined according to the positions of amino-acid gaps in the alignments of the amino acid sequences. Non-sequenced fragments were defined as missing loci. Recombination in the nuclear markers was tested in the program RDP3 (Martin et al., 2010) with default setting. The test indicated that there was no recombination in the nuclear markers.

### Phylogenetic analyses

Gene trees were reconstructed just for the mitochondrial DNA (mtDNA) data and four-gene concatenated data, respectively, because there were few variable sites in nuclear gene data for reconstructing reliable nuclear gene trees. Maximum likelihood (ML) and Bayesian Inference (BI) analyses were conducted in the programs RAXML v.8.1.24 (Stamatakis, 2014) and MRBAYES v.3.2.6 (Ronquist et al., 2012), respectively. To avoid under- or over-parameterization (Lemmon & Moriarty, 2004; McGuire et al., 2007), the best partition scheme and the best evolutionary model for each partition were selected for the phylogenetic analyses using the program PARTITIONFINDER v.1.1.1 (Lanfear et al., 2012). For PARTITIONFINDER analyses, *16S* gene and each codon position of each protein-coding gene was defined and the “greedy” search where branch length estimates were unlinked under the Bayesian Inference Criteria (BIC) as recommended by Lanfear et al. (2012) was performed. In ML analyses, the best tree was produced from 1,000 iterations of the random addition of taxa. For the ML tree, branch supports were drawn from 10,000 non-parametric bootstrap replicates. Four-gene concatenated data was analyzed with joint branch optimization to minimize the impact of missing loci on branch lengths. For BI analyses, gaps were scored as presence/absence data in the program GAP-CODER (Young & Healy, 2003) using the procedure of (Simmons, Ochoterena & Carr, 2001). In BI analyses, the parameters for each partition were unlinked, and branch lengths were allowed to vary proportionately across partitions. Two independent runs were initiated each with four simultaneous Markov Chain Monte Carlo (MCMC) chains for 100 million generations and sampled every 1,000 generations. Convergence of chains and burn-in periods were determined using the program TRACER v.1.6 (Rambaut et al., 2014). Then the burn-in samples (the first 25% defined by Tracer) were discarded, and then a maximum bifurcating consensus tree and posterior probability (pp) were derived from the remaining samples.

To explore the possibility that the unlinked gene trees might be incongruent among each other or with the species tree due to processes such as hybridization, incomplete lineage sorting, horizontal gene transfer, or gene duplication (Maddison, 1997; Kubatko & Degnan, 2007; Degnan & Rosenberg, 2009; Edwards & Beerli, 2000), the species tree was inferred using multispecies coalescent inference. The analyses were conducted using the *BEAST option (Heled & Drummond, 2010) in the program BEAST v.2.3.0 (Bouckaert et al., 2014), as described in the following section together with dating estimations.

### Dating analyses

Divergence times were estimated also based just on mitochondrial data and four-gene concatenated data, respectively, because there were few variable sites in nuclear gene data for reconstructing reliable nuclear gene trees. Time-calibrated phylogenies were reconstructed by incorporating fossil calibration points using BEAST. Four Salamandridae fossil records were used as calibration points. The first calibration point was the divergence between *Tylototriton s.l.* and *Pleurodeles* for which the minimum age was set to 44 Mya, based on a *Tylototriton*-related salamandrid fossil, *Chelotriton* (Herre, 1935; Estes, 1981; Milner, 2000). The relevant fossil of *Brachycormus* was not used because it was alleged to have been a neotenic amphibian or a derivate of *Chelotriton* (Roček, 1996a; Roček, 1996b). Secondly, the minimum age of ancestor of *Taricha* and *Notophthalmus* was presumed to be 22 Mya based on a fossil of *Taricha oligocenica* (Estes, 1981). Then the minimum age of the common ancestor of *Triturus* was given as 24 Mya based on a *Triturus* fossil (Böhme, 2003). Finally, based on the fossil of *Procynops miocenicus* closely related to *Cynops* (Estes, 1981), we presumed the minimum divergence time between *Cynops* and *Paramesotriton* at ∼15 Mya. A lognormal prior distribution was applied for each calibration point in view of the fact that this model allowed for uncertainty of the age estimation of the fossil and bias associated with the incompleteness of the fossil record (Forest, 2009).

For BEAST analyses, to avoid zero-length branches, each coalescent cluster was represented by one specimen. So the Generalized Mixed Yule Coalescent (GMYC) method (Pons et al., 2006; Fontaneto et al., 2007) was used to identify coalescent clusters within *Tylototriton s.l.* The GMYC method was proposed to potentially promote objective delimitation of coalescent clusters even based on single gene and supplies explanations for divergences between clusters by speciation models (Powell, 2012). GMYC analysis was implemented in the R package SPLITS (Species Limits by Threshold Statistic) using the single-threshold option. Because the GMYC analysis needs a time-scale tree, a preliminary BEAST analysis was conducted only based on mtDNA data set with the best partitioning scheme and the best model for each partition selected above. In this analysis, a conservative parameter setting (i.e., a relaxed lognormal clock with the mean rate set to 1 and a coalescent Yule model as tree prior) was assigned (Monaghan et al., 2009). MCMC chains were run for 20 million generations with sampling every 1,000 generations, and the first 25% generations were discarded as burn-in. GMYC analysis resulted in 39 cluster representatives (see ‘results’), which were used for the following analyses.

For BEAST analyses, the best partition scheme and the best evolutionary model for each partition were also selected for the phylogenetic analyses using the program PARTITIONFINDER. An unlinked uncorrected lognormal relaxed clock to each partition, a Yule speciation process for branching rates, and the model of four rate categories for nucleotide substitutions were defined for the analyses. A ML tree was used as a starting tree inferred by RAXML based on single-GMYC-cluster representatives. Two runs of 100 million generations sampling every 1,000 generations were conducted. The convergence of MCMC chains and burn-in periods were determined by Tracer. A maximum clade credibility (MCC) tree with posterior probability was calculated using TREEANNOTATOR.

Species tree was inferred using *BEAST. The method needs prior assignments of specimens to species. So the selection of specimens for *BEAST analyses was according to the GMYC clusters and the sampling number (>1) of each cluster. GMYC singletons and individuals with missing data genes were excluded. Because we obtained sequences of nuclear genes only for 91 *Tylototriton s.l.* samples and two *E. chinhaiensis* samples, outgroup fossil calibrations (see above) could not be utilized for the *BEAST analysis. So, the nucleotide substitution rate of each partition was set according to the rate estimations from the four-gene concatenated Beast analyses conducted above. For *BEAST analysis, mitochondrial DNA (*16S* + *ND2*) and each nuclear gene was defined as an independent partition, respectively. The best-fitting substitution model for each partition was selected by the program JMODELTEST v.2.1 (Guindon & Gascuel, 2003; Darriba et al., 2012). *BEAST analyses were carried out using the unlinked uncorrected lognormal relaxed clock to each partition and a Yule speciation process as tree prior. Lognormal prior distributions were assigned for mean rate of each partition. Two runs of 150 million generations were carried out with sampling every 1,000 generations. Convergence of MCMC chains and burn-in periods were assessed using TRACER.

Topology of the *BEAST tree with fewer number of species was almost consistent with the BEAST tree except for the position of one major clade (see ‘results’). Therefore, to produce a “complete species-time-tree”, a final topology-constrained BEAST analysis was conducted based on mtDNA of GMYC cluster representatives and all outgroups by applying a constraint of the topology as the *BEAST species tree. The fossil calibrations and parameter-settings were specified as constant as the mtDNA-BEAST analysis above.

### Ancestral area reconstructions

Ancestral area was reconstructed using the statistical dispersal-vicariance analysis (S-DIVA; Yu, Harris & He, 2010) and Bayesian Binary MCMC (BBM) all implemented in the program RASP v.3.2 (Yu, Harris & He, 2012). According to the zoogeographic regions defined by Fei, Ye & Jiang (2010) using amphibian data, the distribution range of *Tylototriton s.l.* could be delineated into four regions: A, Northern Indochina Peninsula and South Yunnan Province of China (NIP-SY); B, Southwest China mountains and Eastern Himalaya mountains (SCM-EHM); C, Coastal areas of Southern China region and Hainan Island (CSC-HI); D, Central China region (CC).

Ancestral area reconstructions were based on the final 39-GMYC-cluster MCC tree (see ‘results’) and randomly sampled 2,000 post-burn-in MCMC trees resulted from the final topology-constrained BEAST analysis above. The maximum number of ancestral areas was restricted to two based on distributional suggestions for each species or species group (Fei, Ye & Jiang, 2012) and preliminary analyses comparing the S-DIVA values, analogous to the fit of the data to the area model, of *n* = 2, 3 and 4. The dispersal event was restricted to adjacent regions. The BBM analysis was conducted for 500,000 generations of 10 chains at a temperature of 0.1. Fixed state frequencies of the JC model, equal rate variation among sites and a wide root distribution were specified, and null distribution and distributions outside target regions were prohibited in any reconstructions.

### Diversification analyses

Diversification analyses are dependent on the number of user-specified biodiversity units. Yet there is controversy on the criterion to delimit species-level lineages. To evaluate how robust models of diversification were to variation of number of species-level units, phylogenetic lineages were delimited according to three criteria: GMYC clusters (statistically inferred coalescent lineages), currently recognized nominal species (NS) and NS plus (NSP: NS plus independent lineages representing putative cryptic species, for example, *T. wenxianensis* lineage 2 and 3, see ‘results’). Two thousand trees were randomly sampled from the post-burn-in posterior trees resulted from the final topology-constrained Beast analysis. Then, MCC tree and 2,000 sampled posterior trees were pruned according to each lineage delimitating strategy. The number of lineages varied when we used different units of biodiversity: 39 GMYC-clusters, 23 NS, and 27 NSP (see results). Finally, the pruned 39-tips, 23-tips and 27-tips trees were used for diversification analyses.

To visualize the temporal accumulation of lineages, the log-transformed lineage-through-time (LTT) plots were constructed for each lineage delimitating strategy. To test if the time-lag between the origin and divergence of extant *Tylototriton s.l.* departed from random expectations under the birth-death model, simulations (*n* = 10,000) under birth-death process were run using five different sampling fractions: 100%, 90%, 80%, 70% and 50%, respectively. Parameters for the simulations included the speciation and extinction rates, the age of the stem group and the number of extant lineages all estimated from the observed data based on the birth-death model. Simulations were carried out in the R (R Development Core Team, 2008) package TREESIM (Stadler, 2011). LTT plots of the simulated trees were then constructed to compare with the average LTT plot of the empirical data.

To examine diversification rate shifts, the birth-death likelihood (BDL) test (Rabosky, 2006) was carried out in R packages LASER (Rabosky, 2006) and GEIGER (Harmon et al., 2008). In BDL analysis, six models (pure birth, birth-death, yule2rate, DDL (diversity-dependent linear decrease) and DDX (diversity-dependent exponential decrease)) were fitted to the observed data under the Akaike’s information criterion (AIC). The best model was selected by calculating the difference (ΔAIC) in AIC scores between the best rate-variable (RV) models and the best rate-constant (RC) models (Rabosky, 2006). The significance of the observed ΔAIC_RC_ statistic was assessed by one-tailed tests (Rabosky & Lovette, 2008a). Additionally, other three diversification models were also tested. SPVAR permits speciation rate to vary over time, EXVAR permits extinction rate to vary over time, and BOTHVAR allows both to vary over time (Rabosky & Lovette, 2008b). Finally, Akaike weights (Turkheimer, Hinz & Cunningham, 2003) were calculated for each model.

## Results

### Phylogenetic assignments and dating estimation

A total of 133 samples was included, and 108 individuals representing all currently nominal species of *Tylototriton s.l.* (Table S1). Alignments resulted in 1,035 bps for *ND2*, 501 bps for *NCX1* and 648 bps for *BDNF*, and GBLOCKS analysis resulted in 433 bps for *16S*. In the *ND2* alignment, there were few numbers of gaps existing in several outgroup species.

ML and BI analyses both supported the monophyly of the genus *Tylototriton s.l.*, and all revealed five major clades in it (Fig. 1B). ML and BI analyses supported the relationships among clades as ((I, II), (III, IV, V)). Note that, the presently so-called “*T. asperrimus*”, “*T. wenxianensis*” and “*T. dabienicus*” taxa were not resolved as monophyletic, and contained two, three and two polyphyletic lineages, respectively, which were indicated as containing cryptic species.

**Figure 2:**
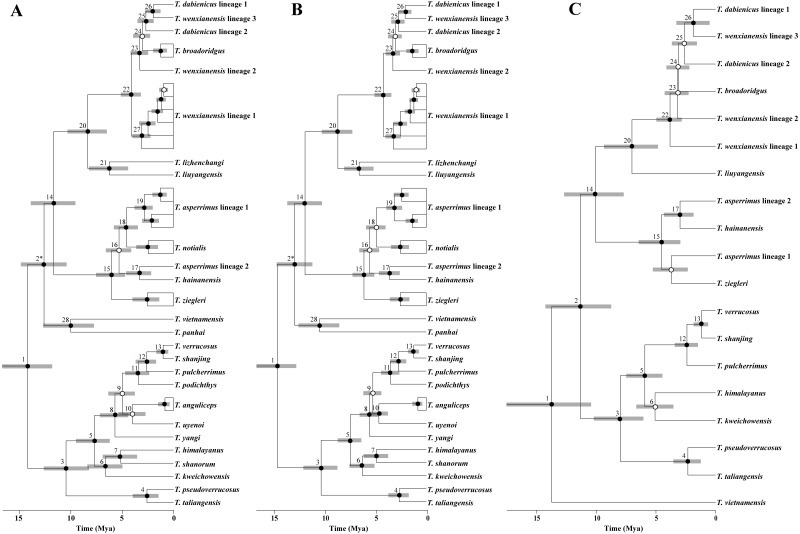
Time-calibrated gene trees and species tree of *Tylototriton s.l*. (A) BEAST gene tree of mtDNA; (B) BEAST gene tree of four-gene concatenated data; (C) species tree. Node number was denoted near each node. Node 2* in gene trees and node 2 in species tree had different topology. Bayesian posterior probabilities (pp) were denoted on nodes. Black circles: pp = 0.95–1.0; open circles: pp < 0.95.

GMYC analysis resulted in 39 independent coalescent clusters within *Tylototriton s.l.* (*P* < 0.01), consisting of 15 singletons and 24 clusters (Fig. S1). After excluding GMYC singletons and individuals with missing data genes, 19 GMYC clusters were used for species-tree analyses. Species tree had almost consistent framework with the gene trees except for one instance of incongruence, i.e., *T. vietnamensis* representing Clade III was clustered at the basal position of the genus in species tree, but in gene trees, this relationships was not resolved (Figs. 1 and 2).

**Figure 3:**
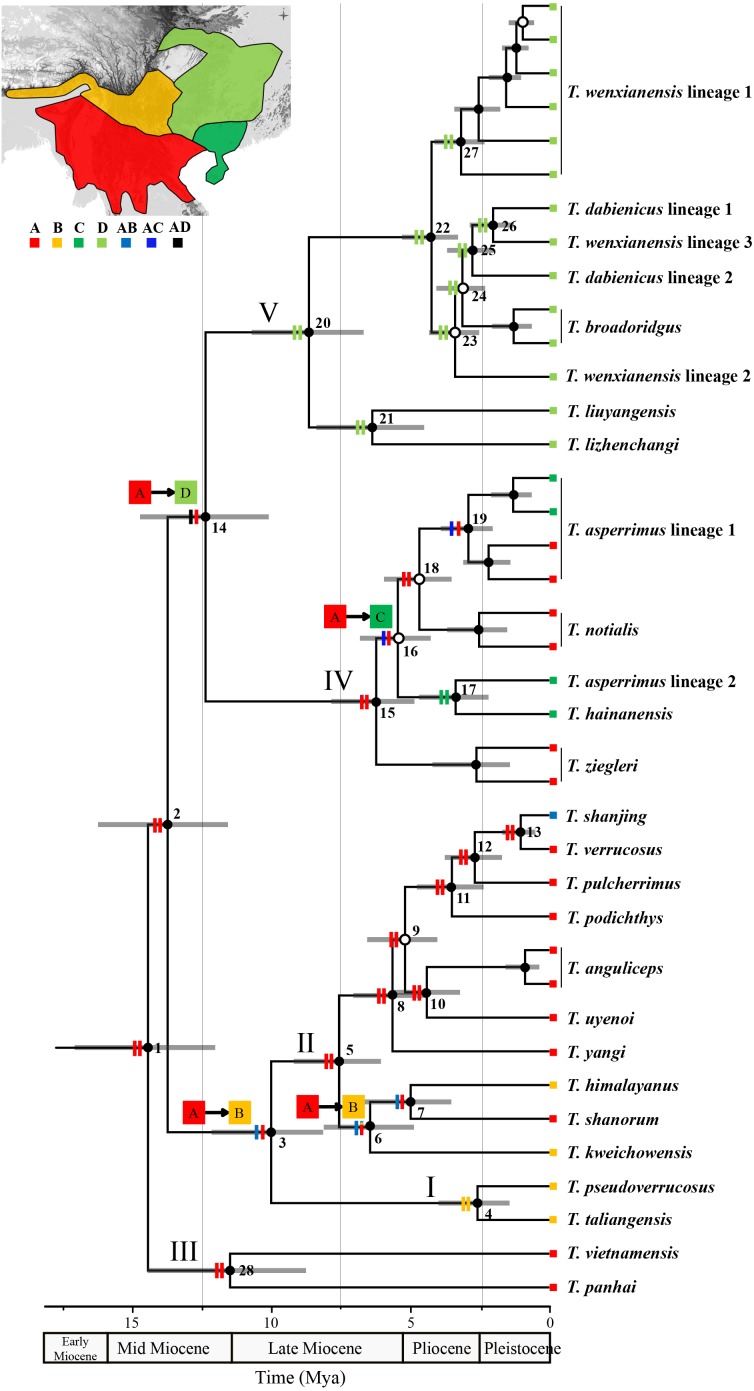
Chronogram and ancestral area reconstructions of *Tylototriton s.l*. This tree was pruned from the final topology-constrained BEAST tree. Grey bars at nodes show 95% highest posterior density intervals of divergence times. Node number was denoted near each node. Black circles, Bayesian posterior probabilities (pp) = 0.95–1.0; open circles, pp < 0.95. Clades I V refer to Fig. 1. Two vertical bars on branch showed the relative probabilities of alternative ancestral distributions obtained by the S-DIVA (first bar) and BBM (second bar), and the first two areas with the highest probability are shown on each bar corresponding to relative probability. Four significant dispersals were highlighted near corresponding nodes using arrows linking the originated region to the dispersed region. (*insert*) four regions was denoted as different color: (A) Northern Indochina Peninsula and South Yunnan Province of China; (B) Southwest China mountains and Eastern Himalaya mountains; (C) Coastal areas of Southern China region and Hainan Island; (D) Central China region.

The chronograms inferred from the gene-tree and species-tree approaches based on different data sets presented similar divergence time estimates (Table 1; Figs. 2 and 3). All dating estimations recovered a middle Miocene-to-present timeframe for *Tylototriton s.l.* (Table 1; Figs. 2 and 3; Fig. S2). Generally, the mean nodal ages estimated using the species-tree approach were little younger than the estimations resulted from the gene-tree approaches, although their 95% highest posterior densities (HPD) overlapped for each node. Except for the divergence of *T. dabienicus* lineage 1 and *T. wenxianensis* lineage 3, and that of *T. shanjing* and *T. verrucosus*, speciation of all nominal species basically predated the Pleistocene.

**Table 1:** Mean node age estimates and 95% highest posterior densities obtained from gene-tree (BEAST) and species-tree approaches (*BEAST). The difference between nodes 2 and 2* refer to Fig. 2.

Node	mtDNA Beast	Four-gene concatenated Beast	*BEAST	Topology-constrained Beast
1	14.1 (11.8–16.6)	14.6 (12.8–16.7)	13.7 (10.4–17.5)	14.4 (12.0–17.0)
2	/	/	11.3 (8.8–14.3)	13.7 (11.6–16.2)
2*	12.5 (10.4–14.8)	12.9 (11.2–14.7)	/	/
3	10.4 (8.2–12.6)	10.3 (8.7–12.1)	8.0 (6.1–10.2)	10.0 (8.1–12.1)
4	2.6 (1.4–3.9)	2.7 (1.7–3.7)	2.3 (1.2–3.5)	2.6 (1.4–4.0)
5	7.6 (6.2–9.4)	7.5 (6.4–8.7)	5.9 (4.5–7.5)	7.6 (6.1–9.2)
6	6.6 (4.9–8.3)	6.3 (5.1–7.5)	5.1 (3.5–6.7)	6.4 (4.9–8.1)
7	5.1 (3.5–6.8)	4.9 (3.7–6.2)	/	5.0 (3.5–6.6)
8	5.7 (4.4–7.1)	5.6 (4.6–6.5)	/	5.6 (4.4–7.0)
9	5.0 (3.7–6.3)	5.3 (3.8–5.6)	/	5.2 (4.0–6.6)
10	4.0 (2.7–5.3)	4.7 (4.4–6.2)	/	4.4 (3.2–5.7)
11	3.4 (2.3–4.7)	3.5 (2.6–4.4)	/	3.5 (2.4–4.8)
12	2.7 (1.7–3.7)	2.7 (2.0–3.6)	2.4 (1.5–3.4)	2.7 (1.7–3.8)
13	1.0 (0.5–1.6)	1.2 (0.7–1.8)	1.2 (0.6–1.8)	1.0 (0.5–1.7)
14	11.6 (9.5–13.8)	11.9 (10.3–13.7)	10.1 (7.7–12.7)	12.4 (10.1–14.7)
15	6.0 (4.7–7.5)	6.1 (5.1–7.2)	4.5 (3.0–6.5)	6.2 (4.8–7.8)
16	5.3 (4.1–6.5)	5.6 (4.6–6.6)	/	5.4 (4.3–6.8)
17	3.3 (2.2–4.6)	3.6 (2.6–4.6)	3.0 (1.8–4.3)	3.4 (2.2–4.7)
18	4.5 (3.4–5.8)	4.9 (4.0–5.9)	/	4.7 (3.5–6.0)
19	2.8 (2.0–3.8)	3.1 (2.4–3.9)	/	2.9 (2.0–3.9)
20	8.3 (6.5–10.3)	8.7 (7.3–10.3)	7.6 (4.8–9.4)	8.6 (6.7–10.7)
21	6.2 (4.4–8.2)	6.6 (5.2–8.1)	/	6.4 (4.5–8.4)
22	4.1 (3.2–5.1)	4.2 (3.4–5.1)	3.8 (2.8–5.0)	4.2 (3.3–5.3)
23	3.3 (2.4–4.2)	3.3 (2.6–4.0)	3.2 (2.3–4.3)	3.4 (2.5–4.3)
24	3.0 (2.3–3.9)	3.0 (2.4–3.8)	3.1 (2.2–4.1)	3.1 (2.3–4.1)
25	2.7 (1.9–3.5)	2.7 (2.1–3.4)	2.6 (1.6–3.7)	2.4 (2.0–3.7)
26	1.9 (1.2–2.7)	2.1 (1.5–2.7)	1.9 (1.0–2.8)	2.0 (1.3–2.8)
27	3.1 (2.2–4.0)	3.2 (2.5–4.0)	/	3.2 (2.3–4.1)
28	10.0 (7.7–12.6)	10.5 (8.6–12.6)	/	11.5 (8.7–14.4)

### Ancestral areas

Ancestral area reconstructions from S-DIVA and BMM analyses based on the “complete species-time” Beast trees were largely consistent (Fig. 3). The probability resulting from two methods for the occurrence of each node was greater than 80%. Bothe methods indicated NIP-SY as the ancestral area of *Tylototriton s.l*. Similarly, the ancestors of Clades II, III and IV were also indicated as originating from NIP-SY. Only Clade I originated from SCM-EHM and Clade V from CC. At least four dispersal events in the late Miocene were supported (Fig. 3). During the middle Miocene ∼12.5 Mya, a dispersal event from NIP-SY to CC was at the origin of the formation and expansion of Clade V. During the middle Miocene ∼10 Mya, a dispersal event from NIP-SY to SCM-EHM was at the origin of the formation of high plateau endemic species (i.e., *T. taliangensis* and *T. pseudoverrucosus*). Then the dispersal from NIP-SY to SCM-EHM during the late Miocene ∼7.5 Mya can explain the speciation of *T. kweichowensis* widely distributed in the Guizhou plateau of China. Finally, a dispersal event from NIP-SY to CSC-HI during the late Miocene can explain the formation of species presently living in the South China.

### Diversification analyses

**Figure 4:**
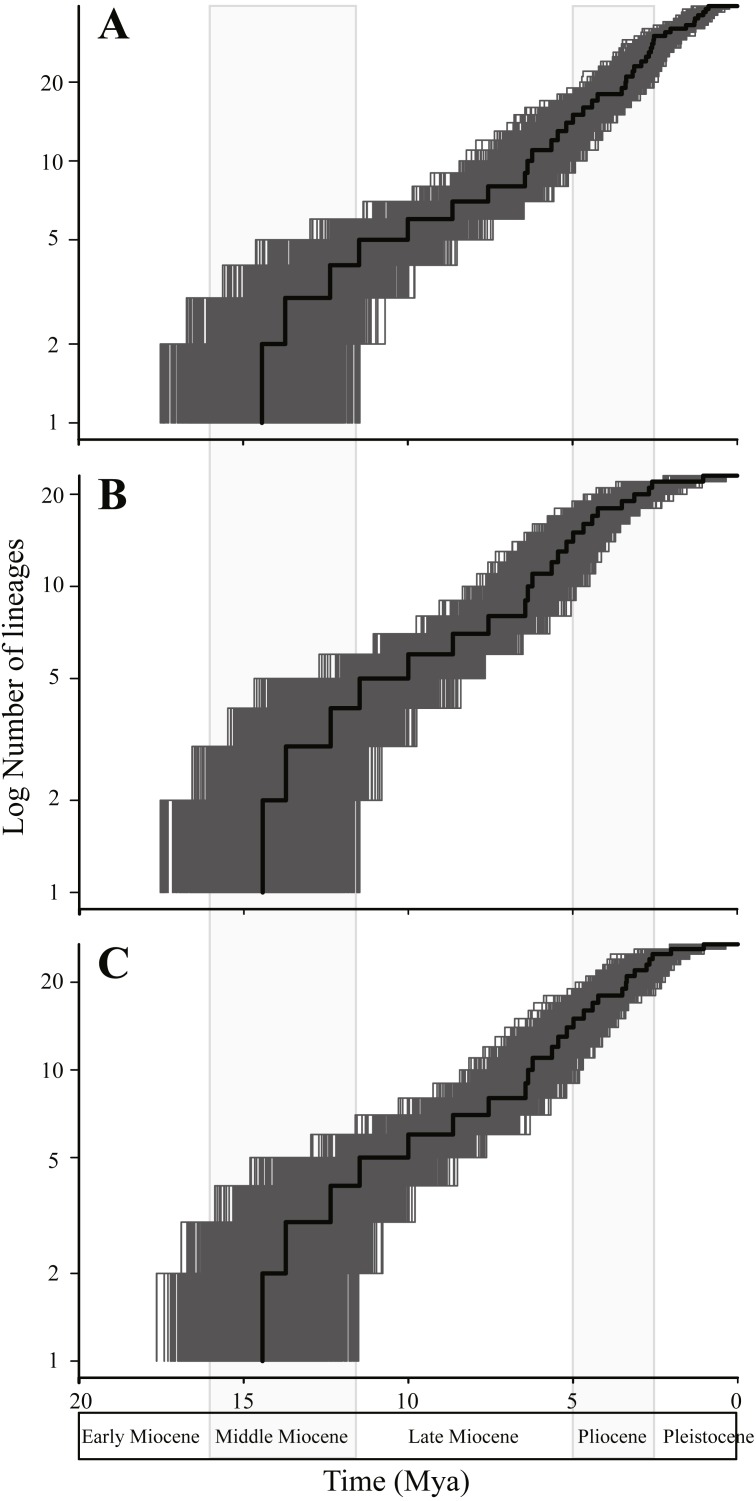
Lineage through time plots based on different lineage delimitating strategies. Different lineage delimitating strategies: (A) 39 statistically inferred coalescent lineages using the Generalized Mixed Yule Coalescent method (GMYC); (B) 23 currently nominal species (NS); and (C) 23 NS plus four independent lineages representing putative cryptic species (NSP). All trees were pruned from the final topology-constrained BEAST analysis. The maximum credibility tree was denoted by a thick black line, and 2,000 random trees from the posterior distribution were denoted by thin grey lines.

LTT plots for 39-GMYC-cluster, 23-NS and 27-NSP data were presented in Fig. 4. The appearance variations of LTT plots for different lineage delimitating dispositions were mainly derived from variations of the number of terminal tips. Using different undersampling scenarios, 10,000 trees were simulated for different lineage delimitating strategies on the observed number of extant species (*n* = 39, 27 and 23) and the observed fixed time since origin (stem age = 22.46 Ma) under the birth-death process (speciation rates and extinction rates for each delimitating strategies were shown in Fig. S3). Under full sampling or more than 50% sampling fraction scenarios, the observed data did not depart from random expectations (Fig. S3).

**Figure 5:**
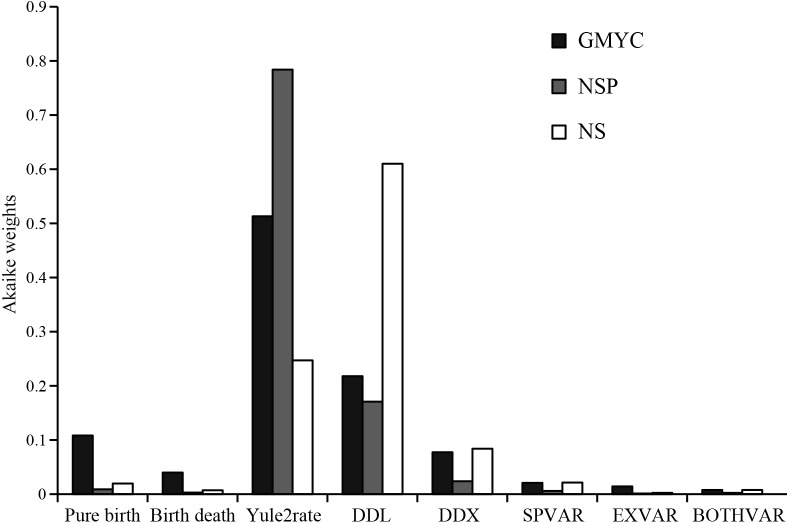
Information theory approach comparing best-fitting diversification rate models. Shown are the Akaike weights of eight diversification rate models (pure birth, birth death, DDL, DDX, yule2rate, SPVAR, EXVAR, and BOTHVAR) and data sets defined by different lineage delimitating strategies: GMYC, 39 statistically inferred coalescent lineages using the Generalized Mixed Yule Coalescent method; NS, 23 currently nominal species; and NSP, 23 NS plus four independent lineages representing putative cryptic species.

BDL tests based on AIC scores resulted in positive ΔAIC values (3.10868 for 39-GMYC-cluster data, 5.03466 for 23-NS data, and 8.98177 for 27-NSP data) being significantly departed from the null hypothesis of constant rate model (all *P* < 0.03). BDL tests (Table S3; Fig. 5) indicated the yule2rate model as the best-fitting model for the 39-GMYC-cluster data and 27-NSP data, but the DDL model as the best-fitting model for the 23-NS data. Yet the yule2rate was selected as the second fitting model for the 23-NS data, as well as the DDL model was selected as the second fitting model for the 39-GMYC-cluster and 27-NSP datasets. For 39-GMYC-cluster data, the diversification rates have sharply decreased around 1 Mya, but for 27-NSP data and 23-NS data, it was at the beginning of Pleistocene, ∼2.6 Mya (Table S3).

## Discussion

### Considerations for uncertainties and cryptic diversity

Our results on the divergence times of the genus *Tylototriton s.l.* and salamandrid major clades depend on the calibrations that we used for estimating these times (Fig. S2). In this case, previous phylogenetic investigations on Salamandridae using these fossils also proposed broadly consistent timeframes for its major clades (Steinfartz et al., 2007; Zhang et al., 2008; Shen et al., 2016). Also for *Tylototriton s.l.*, using these fossil calibrations, dating estimates based on complete mitochondrial genome data (Kurabayashi et al., 2012) were largely consistent with our results based on incorporating data of mitochondrial and nuclear genes (Figs. 2 and 3).

Alternatively, because of some methodological flaw, the dating estimations may be argued to be overestimated. For example, actual divergence times were tended to be overestimated by gene trees because a fraction of gene divergence may pre-date the species divergences (Edwards & Beerli, 2000; Arbogast et al., 2002), and meanwhile, dating time may be underestimated by species-tree approaches in some works due to the uncounted presence of gene flow among lineages (McCormack et al., 2010). In our study, divergence times resulted from gene-tree and species-tree approaches did not differ substantially (Figs. 2 and 3). Similar findings were proposed in many animal groups, such as true crocodiles (Oaks, 2011), iguanian lizards (Townsend et al., 2011), and spiders (Bidegaray-Batista, Ferrández & Arnedo, 2014). Certainly, using large number of unlinked nuclear loci may increase the accuracy of species-tree estimations (Heled & Drummond, 2010). In this study, use of only two nuclear genes could be argued as providing not enough information to obtain “true” time-tree. But in another sense, a low number of nuclear loci combined with mitochondrial genes has the possibility of similar effects on dating estimations as the use of more nuclear markers (Sánchez-Gracia & Castresana, 2012). *Tylototriton s.l.* has likely diversified during a large timescale, i.e., middle Miocene-to-Pleistocene era (Table 1), and therefore, major conclusions could be considered broadly.

Testing diversification rate shifts is considered to be sensitive to species delimitation in a phylogeny (Smith et al., 2013; Renner & Cusimano, 2010). For the genus *Tylototriton s.l.*, either based on biological species or statistically inferred species, BDL tests selected the rate-slowdown model as the best-fitting diversification model. Moreover, different lineage delimitating strategies likely just impacted the terminal characteristics of lineage accumulation (Fig. 4), indicating that our sampling of the “old” clades might be sufficient. This point was verified by the tree-simulation method, which suggested that the simulated data did not depart from the observed data just until under high undersampling scenarios (≥ 50%; see Fig. S3). Although species diversity of the genus will presumedly increase, the rate-slowdown model is still solidly based. It is expected that speciation time of the foreseeable newly-found species less likely will all fall in the recent Pleistocene period but may well spread over several epochs, just like the time of origin of many recently recognized species (e.g., *T. liuyangensis*, *T. panhai*, *T. ziegleri* and *T. yangi*; Fig. 3). Even for the independent lineages that were indicated as putative cryptic species, the dating estimates also suggested that origin of them generally predated the Pleistocene (Fig. 3). On the other side, if it is assumed that our phylogenetic framework has sampled all substantial species, and then we truncated potential synonyms of several species, such as “*T. shanjing*” sometimes indicated as *T. verrucosus* by phylogeographical investigations (Zhang et al., 2007; Khatiwada et al., 2015) and “*T. dabienicus*” previously classified as “*T. wenxianensis dabienicus*” (Chen, Wang & Tao, 2010), our empirical data will more closely resemble the declining rate model because the time of origin of these “redundant” lineages all fall in Pleistocene (Fig. 3).

Of course, evolution of *Tylototriton s.l.* has also been probably confined by other potential factors, such as density-dependent cladogenesis (Rabosky & Lovette, 2008a). Our diversification analyses selected the DDL model as the best-fitting model for 23-NS data, and selected the model as the second fitting model for 39-GMYC-cluster and 27-NSP data (Fig. 5; Table S3). Density-dependent cladogenesis also usually leads to early burst of diversification in old epochs and rate-slowdown appearances in the recent period (Phillimore & Price, 2008). In the DDL model, speciation slows down often since ecological opportunities and geographical space place limits on clade growth (Phillimore & Price, 2008). In regard of *Tylototriton s.l.*, most species prefer humid habitats in mountain forests, and their populations usually show high local endemism (Li, Zou & Luo, 2011; Fei, Ye & Jiang, 2012), indicating that *Tylototriton s.l.* newts probably have strict ecological selection, and niches of most species were probably narrow and easily filled. Presumably, in the Miocene-to-Pliocene epoch, the genus had slowly radiated in the southeastern Asia and increasing number of species had probably slowly filled the niches, and then in recent era, species biodiversity of the genus has been under niche restrictions leading to less speciation. Also, in the present “colder” epoch rather than in the early Miocene (Böhme, 2003), the newts had a low capacity to persist in new locations, most likely preventing populations from evolving into species.

Additionally, the results would be cautiously interpreted for lack of external information about extinction (Rabosky, 2010). Our results indicated that *Tylototriton s.l.* had occupied a long-term constant diversification rate before Pleistocene (Figs. 4 and 5; Table S3). Generally, “long” branches were not expected in the null model assuming a constant diversification rate in an evolutionary phase (Magallón & Sanderson, 2001). But the phylogenetic tree of *Tylototriton s.l.* had several quite long branches (e.g., *T. vietnamensis* and *T. panhai*), possessing basal evolutionary positions and inhabiting the ancestral region, i.e., the northern Indochina (Fig. 3). In the long branches, the pattern of high extinction rate, lack of speciation events and replacements as a result of species turn-over was difficult to be differentiated by only incorporating contemporary taxa (Magallón & Sanderson, 2001). Unfortunately, as there are no ingroup fossil known to now, even the parameter-rich methods might be less powerful for exploring influences from changes of extinction rates and random events (Rabosky, 2010; Quental & Marshall, 2010).

### Radiation of *Tylototriton s.l.* and climatic shifts

Our findings suggested that the genus had originated from the tropical area in NIP-SY, and divergence times between major clades had all fall in the middle-to-late Miocene epochs (Fig. 3). The extensive dispersals from NIP-SY to north especially the mainland of China during the middle-to-late Miocene (Fig. 3) indicate that *Tylototriton s.l.* newts had adaptive radiations in southeastern Asia. It is presumed that diversification of *Tylototriton s.l.* should be closely related with significant climatic and tectonic transformations in the area, just like the diversification history of amount of numerous organisms which was considered to be modulated by the Tibetan Plateau uplifts and the intensifications of EAMs in the late Tertiary (Favre et al., 2014).

Origin of *Tylototriton s.l.* was dated back to ∼15 Mya in the middle Miocene (Fig. 3), when several dramatic climatic transitions had taken place in Asia. Around 16 ∼15 Mya, global climate had been marked by the Miocene Climate Optimum (MCO; Böhme, 2003), but from 15 Mya, climate begun deteriorating, especially in Eurasia; this climate transition led to an increase of seasonality and aridity (Eronen et al., 2009; Bruch et al., 2011). At the same time, the central Tibetan Plateau potentially had a main-body uplift (Spicer et al., 2003; Harris, 2006). These incidents enhanced the EAMs that directly led to the warm and humid climate in the South China (Wan et al., 2007). The climatic shifts further led to turnovers of fauna and flora in Asia (e.g., groups in Klaus et al., 2016). Correspondingly, the northward extensive dispersals from NIP-SY to CC in common ancestors of major clades of *Tylototriton s.l.* (Fig. 3) were much likely caused by the prosperity of the suitable subtropical monsoon climates in the mainland.

Evidence from the thermochronology, sedimentology, oceanography, and palaeoclimatology supported a rapid uplift of the Tibetan Plateau during the late Miocene about 10∼7 Mya that further enhanced the EAMs (An et al., 2001). Diversification of biodiversity in southeastern Asia was reported to be promoted by these remarkable events (Mao et al., 2010; Sun et al., 2012; Wang et al., 2012a; Wang et al., 2012b; Yu et al., 2014). Likewise, origin of most clades in *Tylototriton s.l.* were also estimated from the late Miocene (Fig. 2), indicating the impact of these historical climatic shifts on diversification of the newt genus.

Radiation of *Tylototriton s.l.* in southeastern Asia could also be shown by the fact that the Miocene-derived clades occupy natural environments distinct from those of the common ancestor of the genus. The common ancestor of *Tylototriton s.l.* and most clades likely shared habitats in the tropical area or near-tropical area in the northern Indochina Peninsula with the putative exceptions of clades I and V (Fig. 3). Clade I, containing two species (i.e., *T. taliangensis* and *T. pseudoverrucosus*; Figs. 1 and 3), is of particular endemism to several sky-island mountains in eastern Hengduan Mountains, where most populations inhabit high plateau (about 2,200–3,300 m; Li, Zou & Luo, 2011; Fei, Ye & Jiang, 2012). Their habitats are generally located in the northern subtropical or even temperate zones (Fei, Ye & Jiang, 2012). Furthermore, most species in clade V occupy the northernmost distribution range (Fig. 1A). In particular, newts in these clades undergo a dormant phase in the longer-term “cold” seasons by hiding themselves in soil caves or underground tree holes. Our results suggest that the evolution of these “specialized” lineages have been triggered the dispersal events from tropical area to the subtropics and/or the temperate zone during the late Miocene. Moreover, these species show very distinct morphological characters (Fei, Ye & Jiang, 2012) and reproductive behavior (such as *T. taliangensis*). All these points support their origin through Miocene radiations to the subtropics and temperate regions. Of course, reinforcement of the EAMs following the rapid uplifts of the Tibetan Plateau during the late Miocene (An et al., 2001) already changing biodiversity profiles in the southeastern Asia had certainly contributed to the radiations of these lineages to subtropics and plateau zones.

Anyhow, the empirical data generally support that diversification rates of the genus have decreased at beginning of Pleistocene which seems to reject the Pleistocene speciation hypothesis in this group. The rate-slowdown model was also previously observed in other animal groups, such as reptiles (Rawlings et al., 2008), birds and mammals (Weir & Schluter, 2007) and beetles (McKenna & Farrell, 2006). Numerous studies on amphibians occurring in the southeastern Asia also revealed that interspecific and even intraspecific divergences observed predate the Pleistocene (Wang et al., 2012a; Wang et al., 2012b; Wang et al., 2013). They supported the hypothesis that speciation of many animal lineages need a relatively long-lasting time at least before the glaciations (Zink & Slowinski, 1995). On the other hand, it is speculated that the glaciations might promote extinction rates that would slow down the diversification rates (Zink, Klicka & Barber, 2004). Based on our empirical findings, it is expected to have caused a drastic increase in extinction rates at the beginning, followed by depressed diversification rates due to the smaller areas that remained or became available as the habitat for the newts. As mentioned above, any attempt to estimate or simulate diversification rates from data from extant organisms alone is bound to underestimate both extinction and diversification rates, because whole radiations have gone extinct.

## Conclusions

Our findings provided evidence for the profound influences of historical climate shifts especially associated with the uplift of the Tibetan Plateau on the diversification of animals occurring in the southeastern Asia. The results basically confirmed the reports on plant lineages, such as *Rheum* (Sun et al., 2012), *Lepisorus* (Wang et al., 2012a; Wang et al., 2012b), *Juniperus* (Mao et al., 2010) and *Isodon* (Yu et al., 2014), and on animal groups, such as *Chiastocheta* Pokorny, 1889 (Anthomyiidae, Diptera, Insecta; Espíndola, Buerki & Alvarez, 2012) and spiny frogs (Dicroglossidae, Anura, Amphibia; Che et al., 2010). Yet the present study further supplied some other indications. Primarily, although diversification of the newt group was promoted by the climatic shifts, several factors, for example, niche limits and their intrinsic low dispersal capacity, might have contributed to their Pleistocene rate-slowdown diversification. In addition, this study provided a putative “complete” species tree for *Tylototriton s.l.* to date. The inclusion of many putative cryptic species in the diversification analyses seems to be effective in finding diversification models of the group. But at present, wild populations of *Tylototriton s.l.* show a significant decline due to many reasons, such as climate changes, human capture and habitat deterioration. Further deep investigations of undetected cryptic lineages might supply the basic requirement for disclosing “true” diversification history of the taxa.

##  Supplemental Information

10.7717/peerj.4384/supp-1 Table S1 Sampling numbers, vouchers, localities and GenBank Accession numbers of sequences used in this study “*” sequences were downloaded from GenBank. “#” sequences were retrieved form Zhao et al. (2012). CIB, Chengdu Institute of Biology, Chinese Academy Science; HNNU, Henan Normal University; IEBR, Institute of Ecology and Biological Resources; VNUH, Vietnam National UniversityHanoi; VNMN, Vietnam National Museumof Nature; KUHE, Graduate School of Human and Environmental Studies of Kyoto University; CSUFT, Central South University of Forestry and Technology.

10.7717/peerj.4384/supp-2 Table S2 Primers for PCR and sequencing in this study

10.7717/peerj.4384/supp-3 Table S3 Testing diversification models in the genus *Tylototriton* s.l. using birth–death likelihood Different lineage delimitating strategies: GMYC, 39 statistically inferred coalescent lineages using the Generalized Mixed Yule Coalescent method; NS, 23 currently nominal species; NSP, 23 NS plus four independent lineages representing putative cryptic species. DDL and DDX, logistic and exponential density-dependent speciation models, respectively; yule2rate, multi-rate variant of the Yule model; SPVAR,time-varying speciation only, with a constant extinction rate; EXVAR, time-varying extinction only; BOTHVAR, both speciation and extinction vary over time; r1–r2, net diversification rate (speciation event per million years); a, extinction fraction; st, time of rate shift (Myr); *κ*, parameter in the logistic density dependent model; x, parameter in the density-dependent exponential model; *κ*, initial speciation rate for SPVAR model; *κ*, parameter of the exponential change in speciation rate for BOTHVAR model; z, parameter of exponential change in extinction rate; l, final extinction rate; Ln(L), loglikelihood; AIC, Akaike information criterion; deltaAIC, difference in AIC scores between Pure Birth model and the best-fit model.

10.7717/peerj.4384/supp-4 Figure S1 Ultrametric tree for the mitochondrial genes of *Tylototriton* s.l. resulted from GMYC analysis. Red shading branches indicate GMYC clusters. “*” individuals were used in *BEAST analyses Red shading branches indicate GMYC clusters. “*” individuals were used in *BEAST analyses.

10.7717/peerj.4384/supp-5 Figure S2 Divergence times within Salamandridae and fossil calibrations (C1–C4) for dating analyses The tree was resulted from the topology-constrained Beast analysis. Fossil calibrations C1–C4 were given in materials for details. Grey bars at nodes show 95% highest posterior density intervals of divergence times.

10.7717/peerj.4384/supp-6 Figure S3 Lineage through time plots for different lineage delimitating strategies based on 10,000 simulated trees under different sampling fractions Different lineage delimitating strategies: GMYC, 39 statistically inferred coalescent lineages using the Generalized Mixed Yule Coalescent method; NS, 23 currently nominal species; and NSP, 23 NS plus four independent lineages representing putative cryptic species. Trees were simulated under the observed fixed time since origin (stem age = 22.6 Ma) using the constant rate birth–death process, with speciation and extinction rate values estimated from the empirical data (see Table S3 for different delimitating strategies). Simulations run under full sampling scenario, 10% missing extant lineage, 20% missing extant lineages, 30% missing extant lineages and 30% missing extant lineages. Thick bars correspond to the observed age of the common ancestor of extant * Tylototriton* s.l., with grey rectangles representing the 95% highest posterior density (HPD) interval.

10.7717/peerj.4384/supp-7 Data S1 The raw data of DNA

## Additional Information and Declarations

### Field Study Permissions

The following information was supplied relating to field study approvals (i.e., approving body and any reference numbers): Field experiments were approved by the Management Office of the Kuankuoshui Nature Reserve (number: KKSNR201204002) and the Management Office of the Dabashan Nature Reserve (number: DBSNR201204002).

### DNA Deposition

The following information was supplied regarding the deposition of DNA sequences:
KY800540–KY800628, KT765173, KT765207, KT765208, KY800813–KY800892, KY800629–KY800719, KY800720–KY800812.

### Data Availability

The following information was supplied regarding data availability: The raw data has been provided as a Supplemental File.
