# Supplementary material for: Phylogenetic surveys on the newt genus Tylototriton sensu lato (Salamandridae, Caudata) reveal cryptic diversity and novel diversification promoted by historical climatic shifts
Source: PeerJ. 2018 Mar 12;6:e4384. doi: 10.7717/peerj.4384 (PMC5853667; doi:10.7717/peerj.4384)
Supplement: Table S1 — “*” sequences were downloaded from GenBank. “#” sequences were retrieved form Zhao et al. (2012). CIB, Chengdu Institute of Biology, Chinese Academy Science; HNNU, Henan Normal University; IEBR, Institute of Ecology and Biological Resources; VNUH, Vietnam National UniversityHanoi; VNMN, Vietnam National Museumof Nature; KUHE, Graduate School of Human and Environmental Studies of Kyoto University; CSUFT, Central South University of Forestry and Technology. [file peerj-06-4384-s001.docx]

| **Sample number** | **Species name** | **Voucher Number** | **Locality** | **16S** | **ND2** | **BNDF** | **NCX1** | **Combined sequences ID** |
| --- | --- | --- | --- | --- | --- | --- | --- | --- |
| 1 | *Tylototriton taliangensis* | CIBGG200110183 | Shimian Co., Yan'an City, Sichuan Province, China | KY800559 | KC147819* | KY800648 | KY800739 | X46 |
| 2 | *Tylototriton taliangensis* | CIBGG200110185 | Shimian Co., Yan'an City, Sichuan Province, China | KY800560 | KY800829 | KY800649 | KY800740 | X48 |
| 3 | *Tylototriton taliangensis* | CIBGG200110186 | Shimian Co., Yan'an City, Sichuan Province, China | KY800561 | KY800830 | KY800650 | KY800741 | X49 |
| 4 | *Tylototriton pseudoverrucosus* | CIBWCG2012003 | Ningnan Co., Liangshanyizu state, Sichuan Province, China | KY800597 | KY800861 | KY800687 | KY800778 | NN3 |
| 5 | *Tylototriton pseudoverrucosus* | CIBWCG2012007 | Ningnan Co., Liangshanyizu state, Sichuan Province, China | KY800598 | KY800862 | KY800688 | KY800779 | NN7 |
| 6 | *Tylototriton pseudoverrucosus* | CIBWCG2012012 | Ningnan Co., Liangshanyizu state, Sichuan Province, China | KY800599 | KY800860 | KY800686 | KY800777 | NN12 |
| 7 | *Tylototriton kweichowensis* | CIBWg20080818014 | Bijie City, Guizhou Province, China | KY800551 | KY800823 | KY800640 | KY800731 | X32 |
| 8 | *Tylototriton kweichowensis* | CIBWg20080818018 | Bijie City, Guizhou Province, China | KY800552 | KY800824 | KY800641 | KY800732 | X36 |
| 9 | *Tylototriton kweichowensis* | CIB20050213 | Shuicheng City, Guizhou Province, China | KY800557 | KY800827 | KY800646 | KY800737 | X42 |
| 10 | *Tylototriton kweichowensis* | CIB20050215 | Shuicheng City, Guizhou Province, China | KY800558 | KY800828 | KY800647 | KY800738 | X44 |
| 11 | *Tylototriton shanorum* | - | Taunggyi Township, Shan State, Myanmar | / | AB922822* | / | / | AB769544 |
| 12 | *Tylototriton shanorum* | - | Taunggyi Township, Shan State, Myanmar | / | AB922823* | / | / | AB922822 |
| 13 | *Tylototriton shanorum* | - | Taunggyi Township, Shan State, Myanmar | / | AB769544* | / | / | AB922823 |
| 14 | *Tylototriton himalayanus* | CIB201406246 | Mai Pokhari, Illam, Mechi,Nepal | KY800590 | KT765173 | KY800679 | KY800770 | JA246 |
| 15 | *Tylototriton himalayanus* | CIB201406284 | Bagh Khor, Illam, Mechi,Nepal | KY800591 | KT765207 | KY800680 | KY800771 | JA284 |
| 16 | *Tylototriton himalayanus* | CIB201406285 | Bagh Khor, Illam, Mechi,Nepal | KY800592 | KT765208 | KY800681 | KY800772 | JA285 |
| 17 | *Tylototriton yangi* | KIZ201203001 | Pingbian Co., Yunnan Province, China | / | # | / | / | yangi7191 |
| 18 | *Tylototriton yangi* | KIZ201203002 | Pingbian, Yunnan Province, China | / | # | / | / | yangi7192 |
| 19 | *Tylototriton yangi* | KUHE:42282 | Pet Trade | KY800624 | KY800887 | KY800714 | KY800807 | TY36 |
| 20 | *Tylototriton uyenoi* | - | Doi Ang Khang, Chiang Mai, Thailand | / | AB830729* | / | / | AB830729 |
| 21 | *Tylototriton uyenoi* | - | Doi Inthanon, Chiang Mai, Thailand | / | AB830730* | / | / | AB830730 |
| 22 | *Tylototriton uyenoi* | - | Doi Suthep, Chiang Mai, Thailand | / | AB830733* | / | / | AB830733 |
| 23 | *Tylototriton anguliceps* | - | Thuan Chau, Son La, Vietnam | / | LC017833* | / | / | LC017832 |
| 24 | *Tylototriton anguliceps* | - | Muong Nhe, Dien Bien, Vietnam | / | LC017832* | / | / | LC017833 |
| 25 | *Tylototriton anguliceps* | - | Doi Lahnga, Chiang Mai, Thailand | / | AB830728* | / | / | AB830728 |
| 26 | *Tylototriton podichthys* | - | Phu Pan, Xam Neua, Laos | / | AB830727* | / | / | AB830727 |
| 27 | *Tylototriton podichthys* | - | Xam Neua, Huaphanh, Laos | / | LC017835* | / | / | LC017835 |
| 28 | *Tylototriton pulcherrimus* | CIBTY040 | Lüchun Co., Yunnan Province, China | KY800626 | KY800890 | KY800717 | KY800810 | TY40 |
| 29 | *Tylototriton pulcherrimus* | KUHE:46406 | Pet Trade | KY800620 | KY800880 | KY800709 | KY800800 | TY29 |
| 30 | *Tylototriton verrucosus* | CIB-TSHS1 | Longchuan Co., Dehong state, Yunnan Province, China | KY800581 | KY800847 | KY800670 | KY800761 | X93 |
| 31 | *Tylototriton verrucosus* | CIB-TSHS2 | Longchuan Co., Dehong state, Yunnan Province, China | KY800582 | KY800848 | KY800671 | KY800762 | X94 |
| 32 | *Tylototriton verrucosus* | CIB-TSHS3 | Longchuan Co., Dehong state, Yunnan Province, China | KY800583 | KY800849 | KY800672 | KY800763 | X95 |
| 33 | *Tylototriton verrucosus* | CIB-TSHS4 | Longchuan Co., Dehong state, Yunnan Province, China | KY800584 | KY800850 | KY800673 | KY800764 | X96 |
| 34 | *Tylototriton verrucosus* | CIB-TSHS5 | Longchuan Co., Dehong state, Yunnan Province, China | KY800585 | KY800851 | KY800674 | KY800765 | X97 |
| 35 | *Tylototriton verrucosus* | CIB-TSHS6 | Longchuan Co., Dehong state, Yunnan Province, China | KY800586 | KY800852 | KY800675 | KY800766 | X98 |
| 36 | *Tylototriton shanjing* | KIZ201306081 | Yongde Co., Yunnan Province, China | KY800593 | KY800856 | KY800682 | KY800773 | LX652 |
| 37 | *Tylototriton shanjing* | KIZ201306098 | Yun Co., Yunnan Province, China | KY800594 | KY800857 | KY800683 | KY800774 | LX653 |
| 38 | *Tylototriton shanjing* | KIZ201306102 | Jingdong Co., Yunnan Province, China | KY800595 | KY800858 | KY800684 | KY800775 | LX654 |
| 39 | *Tylototriton shanjing* | KIZ201306108 | Nanjian Co., Yunnan Province, China | KY800596 | KY800859 | KY800685 | KY800776 | LX656 |
| 40 | *Tylototriton shanjing* | CIB980004 | Baoshan City, Yunnan Province, China | KY800562 | KY800831 | KY800651 | KY800742 | X50 |
| 41 | *Tylototriton shanjing* | CIB980005 | Baoshan City, Yunnan Province, China | KY800563 | KY800832 | KY800652 | KY800743 | X51 |
| 42 | *Tylototriton shanjing* | CIB980006 | Baoshan City, Yunnan Province, China | KY800564 | KY800833 | KY800653 | KY800744 | X52 |
| 43 | *Tylototriton panhai* | - | Phu Hin Rong Kla NP, Phitsanulok, Thailand | / | AB830735* | / | / | AB830735 |
| 44 | *Tylototriton panhai* | - | Phu Luang WS, Loei, Thailand | / | AB830736* | / | / | AB830736 |
| 45 | *Tylototriton vietnamensis* | IEBRA.3674 | Yen Tu, Bac. Giang Prov., Vietnam | KY800614 | KY800874 | KY800703 | KY800794 | TY13 |
| 46 | *Tylototriton vietnamensis* | IEBRA.0702 | Mauson, Lang Son Province, Vietnam | KY800612 | KY800872 | KY800701 | KY800792 | TY14 |
| 47 | *Tylototriton vietnamensis* | IEBRA.0701 | Mauson, Lang Son Province, Vietnam | KY800613 | KY800873 | KY800702 | KY800793 | TY15 |
| 48 | *Tylototriton ziegleri* | VNMN3389 | Bao Lac, Cao Bang, Vietnam | / | KY800888 | KY800715 | KY800808 | TY38 |
| 49 | *Tylototriton ziegleri* | VNMN3390 | Quan Ba, Ha Giang, Vietnam | KY800625 | KY800889 | KY800716 | KY800809 | TY39 |
| 50 | *Tylototriton ziegleri* | VNUHHG.082 | Quan Ba, Ha Giang, Vietnam | KY800610 | KY800870 | KY800699 | KY800790 | TY11 |
| 51 | *Tylototriton ziegleri* | VNUHHG.081 | Quan Ba, Ha Giang, Vietnam | KY800611 | KY800871 | KY800700 | KY800791 | TY12 |
| 52 | *Tylototriton hainanensis* | CIB20081048 | Mt. Diaoluo, Hainan Province, China | KY800553 | KC147817* | KY800642 | KY800733 | X37 |
| 53 | *Tylototriton hainanensis* | CIB20081049 | Mt. Diaoluo, Hainan Province, China | KY800554 | KC147818* | KY800643 | KY800734 | X38 |
| 54 | *Tylototriton hainanensis* | CIB20081051 | Mt. Diaoluo, Hainan Province, China | KY800555 | KY800825 | KY800644 | KY800735 | X40 |
| 55 | *Tylototriton hainanensis* | CIB20081052 | Mt. Diaoluo, Hainan Province, China | KY800556 | KY800826 | KY800645 | KY800736 | X41 |
| 56 | *Tylototriton asperrimus* lineage 2 | CIBXZ20091201 | Xinyi City, Guangdong Province, China | KY800616 | KY800876 | KY800705 | KY800796 | TY20 |
| 57 | *Tylototriton asperrimus* lineage 2 | CIBXZ20091204 | Xinyi City, Guangdong Province, China | KY800619 | KY800879 | KY800708 | KY800799 | TY23 |
| 58 | *Tylototriton asperrimus* lineage 2 | CIBXZ20091 | Xinyi City, Guangdong Province, China | KY800618 | KY800878 | KY800707 | KY800798 | TY22 |
| 59 | *Tylototriton asperrimus* lineage 2 | CIBXZ20092 | Xinyi City, Guangdong Province, China | KY800617 | KY800877 | KY800706 | KY800797 | TY21 |
| 60 | *Tylototriton notialis* | - | Boualapha Dist., Khammouan, Province, Laos | / | HM462061* | / | / | HM462061 |
| 61 | *Tylototriton notialis* | - | Boualapha Dist., Khammouan, Province, Laos | / | HM462062* | / | / | HM462062 |
| 62 | *Tylototriton notialis* | VNMNTAO1229 | Pu Hoat, Nghe An, Vietnam | / | KY800883 | KY800712 | KY800803 | TY32 |
| 63 | *Tylototriton notialis* | VNMNTAO1235 | Pu Hoat, Nghe An, Vietnam | / | KY800884 | KY800713 | KY800804 | TY33 |
| 64 | *Tylototriton asperrimus* lineage 1 | VNMNTAO1213 | Thuong Tien, Hoa Binh, Vietnam | KY800623 | KY800885 | / | KY800805 | TY34 |
| 65 | *Tylototriton asperrimus* lineage 1 | VNMNTAO1214 | Thuong Tien, Hoa Binh, Vietnam | / | KY800886 | / | KY800806 | TY35 |
| 66 | *Tylototriton asperrimus* lineage 1 | CIB70063 | Longsheng Co., Guangxi Province, China | KY800549 | KC147816* | KY800638 | KY800729 | X27 |
| 67 | *Tylototriton asperrimus* lineage 1 | CIBGX20080714 | Jinxiu Co., Guangxi Province, China | KY800546 | KY800819 | KY800635 | KY800726 | X8 |
| 68 | *Tylototriton asperrimus* lineage 1 | CIBGX200807010 | Jinxiu Co., Guangxi Province, China | KY800547 | KY800820 | KY800636 | KY800727 | X9 |
| 69 | *Tylototriton asperrimus* lineage 1 | CIBGX200807012 | Jinxiu Co., Guangxi Province, China | KY800548 | KY800821 | KY800637 | KY800728 | X10 |
| 70 | *Tylototriton asperrimus* lineage 1 | CIBGX200807016 | Jinxiu Co., Guangxi Province, China | KY800550 | KY800822 | KY800639 | KY800730 | X28 |
| 71 | *Tylototriton asperrimus* lineage 1 | CIB20070715 | Jinxiu Co., Guangxi Province, China | KY800565 | KY800834 | KY800654 | KY800745 | X55 |
| 72 | *Tylototriton asperrimus* lineage 1 | CIB200807055 | Jinxiu Co., Guangxi Province, China | KY800566 | KC147815* | KY800655 | KY800746 | X56 |
| 73 | *Tylototriton liuyangensis* | CSUFT20100108 | Liuyang City, Hunan Province, China | KY800606 | KJ205598* | KY800695 | KY800786 | qp1091 |
| 74 | *Tylototriton liuyangensis* | CIB110601F06 | Liuyang City, Hunan Province, China | KY800615 | KY800875 | KY800704 | KY800795 | TY17 |
| 75 | *Tylototriton lizhenchangi* | KUHE:42316 | Yizhang Co., Hunan, China | KY800621 | KY800881 | KY800710 | KY800801 | TY30 |
| 76 | *Tylototriton lizhenchangi* | KUHE:42317 | Yizhang Co., Hunan, China | KY800622 | KY800882 | KY800711 | KY800802 | TY31 |
| 77 | *Tylototriton dabienicus* lineage 2 | CIB08042905-2 | Yuexi Co. Anhui Prov., China | KY800587 | KY800853 | KY800676 | KY800767 | X108 |
| 78 | *Tylototriton dabienicus* lineage 2 | CIB08042905-3 | Yuexi Co. Anhui Prov., China | KY800588 | KY800854 | KY800677 | KY800768 | X109 |
| 79 | *Tylototriton dabienicus* lineage 2 | CIB08042905-4 | Yuexi Co. Anhui Prov., China | KY800589 | KY800855 | KY800678 | KY800769 | X110 |
| 80 | *Tylototriton broadoridgus* | CIB200085 | Sangzhi Co., Hunan Province, China | KY800569 | KC147814* | KY800658 | KY800749 | X74 |
| 81 | *Tylototriton broadoridgus* | CIB200084 | Sangzhi Co., Hunan Province, China | KY800570 | KY800837 | KY800659 | KY800750 | X75 |
| 82 | *Tylototriton dabienicus* lineage 1 | HNNU1004-015 | Shangcheng Co., Anhui Prov., China | KY800607 | KC147811* | KY800696 | KY800787 | TY01 |
| 83 | *Tylototriton dabienicus* lineage 1 | HNNU1004-024 | Shangcheng Co., Anhui Prov., China | KY800608 | KC147812* | KY800697 | KY800788 | TY03 |
| 84 | *Tylototriton dabienicus* lineage 1 | HNNU1004-026 | Shangcheng Co., Anhui Prov., China | KY800609 | KY800869 | KY800698 | KY800789 | TY05 |
| 85 | *Tylototriton wenxianensis* lineage 3 | CIBWH10001 | Wufeng Co., Hubei Province, China | KY800600 | KY800863 | KY800689 | KY800780 | qp1080 |
| 86 | *Tylototriton wenxianensis* lineage 3 | CIBWH10002 | Wufeng Co., Hubei Province, China | KY800601 | KY800864 | KY800690 | KY800781 | qp1081 |
| 87 | *Tylototriton wenxianensis* lineage 3 | CIBWH10003 | Wufeng Co., Hubei Province, China | KY800602 | KY800865 | KY800691 | KY800782 | qp1082 |
| 88 | *Tylototriton wenxianensis* lineage 3 | CIBWH10007 | Wufeng Co., Hubei Province, China | KY800603 | KY800866 | KY800692 | KY800783 | qp1086 |
| 89 | *Tylototriton wenxianensis* lineage 2 | CIBWg20090730001 | Libo Co., Guizhou Province, China | KY800575 | KY800842 | KY800664 | KY800755 | X82 |
| 90 | *Tylototriton wenxianensis* lineage 2 | CIBWg20090730002 | Libo Co., Guizhou Province, China | KY800576 | KY800843 | KY800665 | KY800756 | X83 |
| 91 | *Tylototriton wenxianensis* lineage 2 | CIBWg20090730003 | Libo Co., Guizhou Province, China | KY800577 | KY800844 | KY800666 | KY800757 | X84 |
| 92 | *Tylototriton wenxianensis* lineage 2 | CIBWg20090730005 | Libo Co., Guizhou Province, China | KY800578 | KY800845 | KY800667 | KY800758 | X86 |
| 93 | *Tylototriton wenxianensis* lineage 2 | CIBWg20090730004 | Libo Co., Guizhou Province, China | KY800580 | KY800846 | KY800669 | KY800760 | X92 |
| 94 | *Tylototriton wenxianensis* lineage 1 | CIBWG200600019 | Suiyang Co., Zunyi City, Guizhou Province, China | KY800544 | KY800817 | KY800633 | KY800724 | X5 |
| 95 | *Tylototriton wenxianensis* lineage 1 | CIBWG20060007 | Suiyang Co., Zunyi City, Guizhou Province, China | KY800545 | KY800818 | KY800634 | KY800725 | X7 |
| 96 | *Tylototriton wenxianensis* lineage 1 | CIBWG20090601002 | Suiyang Co., Zunyi City, Guizhou Province, China | KY800573 | KY800840 | KY800662 | KY800753 | X80 |
| 97 | *Tylototriton wenxianensis* lineage 1 | CIBWG20090601001 | Suiyang Co., Zunyi City, Guizhou Province, China | KY800574 | KY800841 | KY800663 | KY800754 | X81 |
| 98 | *Tylototriton wenxianensis* lineage 1 | CIB20090527 | Wenxian Co., Gansu Province, China | KY800579 | KC147813* | KY800668 | KY800759 | X2 |
| 99 | *Tylototriton wenxianensis* lineage 1 | CIB2010123101 | Pingwu Co., Gansu Province, China | KY800604 | KY800867 | KY800693 | KY800784 | X57 |
| 100 | *Tylototriton wenxianensis* lineage 1 | CIB2010123102 | Pingwu Co., Gansu Province, China | KY800605 | KY800868 | KY800694 | KY800785 | X58 |
| 101 | *Tylototriton wenxianensis* lineage 1 | CIB20070639 | Qingchuan Co., Sichuan Province, China | KY800542 | KY800815 | KY800631 | KY800722 | X1 |
| 102 | *Tylototriton wenxianensis* lineage 1 | CIB20070638 | Qingchuan Co., Sichuan Province, China | KY800543 | KY800816 | KY800632 | KY800723 | X4 |
| 103 | *Tylototriton wenxianensis* lineage 1 | CIB20080002 | Yunyang Co., Chongqing City, China | KY800540 | KY800813 | KY800629 | KY800720 | X78 |
| 104 | *Tylototriton wenxianensis* lineage 1 | CIB20080003 | Yunyang Co., Chongqing City, China | KY800541 | KY800814 | KY800630 | KY800721 | X79 |
| 105 | *Tylototriton wenxianensis* lineage 1 | CIB20081201 | Yunyang Co., Chongqing City, China | KY800567 | KY800835 | KY800656 | KY800747 | qp1089 |
| 106 | *Tylototriton wenxianensis* lineage 1 | CIB20081202 | Yunyang Co., Chongqing City, China | KY800568 | KY800836 | KY800657 | KY800748 | qp1088 |
| 107 | *Tylototriton wenxianensis* lineage 1 | CIBwa20090601 | Wangcang Co., Sichuan Province, China | KY800571 | KY800838 | KY800660 | KY800751 | X3 |
| 108 | *Tylototriton wenxianensis* lineage 1 | CIBwa20090602 | Wangcang Co., Sichuan Province, China | KY800572 | KY800839 | KY800661 | KY800752 | X91 |
| - | *Echinotriton chinhaiensis* | CIBZHJY1 | Zhenhai Co., Zhejiang Province, China | KY800627 | KY800891 | KY800718 | KY800811 | ZHJY1 |
| - | *Echinotriton chinhaiensis* | CIBZHJY2 | Zhenhai Co., Zhejiang Province, China | KY800628 | KY800892 | KY800719 | KY800812 | ZHJY2 |
| - | *Echinotriton andersoni* | - | - | / | EU880314* | / | / | EU880314 |
| - | *Pleurodeles poireti* | - | - | / | EU880329* | / | / | EU880329 |
| - | *Pleurodeles waltl* | - | - | / | EU880330* | / | / | EU880330 |
| - | *Taricha rivularis* | - | - | / | EU880334* | / | / | EU880334 |
| - | *Taricha granulosa* | - | - | / | EU880333* | / | / | EU880333 |
| - | *Notophthalmus viridescens* | - | - | / | EU880323* | / | / | EU880323 |
| - | *Notophthalmus meridionalis* | - | - | / | EU880322* | / | / | EU880322 |
| - | *Euproctus platycephalus* | - | - | / | EU880317* | / | / | EU880317 |
| - | *Ichthyosaura alpestris* | - | - | / | EU880335* | / | / | EU880335 |
| - | *Calotriton asper* | - | - | / | EU880307* | / | / | EU880307 |
| - | *Neurergus kaiseri* | - | - | / | EU880320* | / | / | EU880320 |
| - | *Triturus marmoratus* | - | - | / | EU880337* | / | / | EU880337 |
| - | *Triturus cristatus* | - | - | / | EU880336* | / | / | EU880336 |
| - | *Cynops orientalis* | - | - | / | EU880311* | / | / | EU880311 |
| - | *Cynops pyrrhogaster* | - | - | / | EU880313* | / | / | EU880313 |
| - | *Paramesotriton deloustali* | - | - | / | EU880327* | / | / | EU880327 |
| - | *Laotriton laoensis* | - | - | / | EU880328* | / | / | EU880328 |
| - | *Pachytriton labiatus* | - | - | / | EU880325* | / | / | EU880325 |
| - | *Salamandrina terdigitata* | - | - | / | EU880332* | / | / | EU880332 |
| - | *Chioglossa lusitanica* | - | - | / | EU880308* | / | / | EU880308 |
| - | *Lyciasalamandra atifi* | - | - | / | AF154053* | / | / | AF154053 |
| - | *Salamandra salamandra* | - | - | / | EU880331* | / | / | EU880331 |
| - | *Ambystoma mexicanum* | - | - | / | AY659991* | / | / | AY659991 |
