# Supplementary material for: Phylogenetic surveys on the newt genus Tylototriton sensu lato (Salamandridae, Caudata) reveal cryptic diversity and novel diversification promoted by historical climatic shifts
Source: PeerJ. 2018 Mar 12;6:e4384. doi: 10.7717/peerj.4384 (PMC5853667; doi:10.7717/peerj.4384)
Supplement: Table S2 [file peerj-06-4384-s002.docx]

| **Gene** | **Primer** | **Primer sequence** |
| --- | --- | --- |
| ND2 | SL-1 | 5’-ATAGAGGTTCAAACCCTCTC-3’ |
|  | SL-2 | 5’-TTAAAGTGTCTGGGTTGCATTCAG-3’ |
| 16S | p7 | 5'-CgCCTgTTTACCAAAAACAT-3’ |
|  | p8 | 5'-CCggTCTgAACTCAgATCACgT-3’ |
| NCX1 | NCX-1_F1 | 5'-GTGGAGGTCTGGGAAAGCAT-3' |
|  | NCX-1_R1 | 5'-ACACTGATACGTGGCTTGCT-3' |
| BNDF | BDNF_F1 | 5'-ACCATCCTTTTCCTKACTATGG-3' |
|  | BDNF_R1 | 5'-CTATCTTCCCCTTTTAATGGTC-3' |
