# Supplementary material for: Phylogenetic surveys on the newt genus Tylototriton sensu lato (Salamandridae, Caudata) reveal cryptic diversity and novel diversification promoted by historical climatic shifts
Source: PeerJ. 2018 Mar 12;6:e4384. doi: 10.7717/peerj.4384 (PMC5853667; doi:10.7717/peerj.4384)
Supplement: Table S3 — Different lineage delimitating strategies: GMYC, 39 statistically inferred coalescent lineages using the Generalized Mixed Yule Coalescent method; NS, 23 currently nominal species; NSP, 23 NS plus four independent lineages representing putative cryptic species. DDL and DDX, logistic and exponential density-dependent speciation models, respectively; yule2rate, multi-rate variant of the Yule model; SPVAR,time-varying speciation only, with a constant extinction rate; EXVAR, time-varying extinction only; BOTHVAR, both speciation and extinction vary over time; r1–r2, net diversification rate (speciation event per million years); a, extinction fraction; st, time of rate shift (Myr); κ, parameter in the logistic density dependent model; x, parameter in the density-dependent exponential model; κ, initial speciation rate for SPVAR model; κ, parameter of the exponential change in speciation rate for BOTHVAR model; z, parameter of exponential change in extinction rate; l, final extinction rate; Ln(L), loglikelihood; AIC, Akaike information criterion; deltaAIC, difference in AIC scores between Pure Birth model and the best-fit model. [file peerj-06-4384-s003.docx]

| **Model** | **Ln (L)** | **AICc** | **Akaike weights** | **r1** | **Extinction rate** | **r2** | **st** | **x** | **κ** | **z** |
| --- | --- | --- | --- | --- | --- | --- | --- | --- | --- | --- |
| **GMYC** |  |  |  |  |  |  |  |  |  |  |
| Pure Birth | 4.6863 | -7.3727 | 0.1084 | 0.1909 | 0 |  |  |  |  |  |
| Birth–Death | 4.6863 | -5.3727 | 0.0399 | 0.1909 | 0 |  |  |  |  |  |
| yule2rate | 8.2407 | -10.4814 | 0.5132 | 0.2275 | 0 | 0.0500 | 1.0325 |  |  |  |
| DDL | 6.3841 | -8.7683 | 0.2179 | 0.3184 | 0 |  |  |  | 57.9721 |  |
| DDX | 5.3489 | -6.6977 | 0.0774 | 0.3805 | 0 |  |  | 0.2415 |  |  |
| SPVAR | 5.0415 | -4.0829 | 0.0209 | 0.2850 | 0.001 |  |  |  | 0.0390 |  |
| EXVAR | 4.6745 | -3.3491 | 0.0145 | 0.1913 | 0.001 |  |  |  |  | 1.0012 |
| BOTHVAR | 5.0473 | -2.0947 | 0.0077 | 0.2835 | 0.001 |  |  |  | 0.0387 | 0.0010 |
| **NSP** |  |  |  |  |  |  |  |  |  |  |
| Pure Birth | -11.7698 | 25.5396 | 0.0088 | 0.1464 | 0 |  |  |  |  |  |
| Birth–Death | -11.7698 | 27.5396 | 0.0032 | 0.1464 | 0 | 0.0296 | 2.5845 |  |  |  |
| yule2rate | -5.2789 | 16.5579 | 0.7839 | 0.2232 | 0 |  |  |  |  |  |
| DDL | -7.8022 | 19.6043 | 0.1709 | 0.3631 | 0 |  |  |  | 30.1569 |  |
| DDX | -9.7776 | 23.5551 | 0.0237 | 0.6143 | 0 |  |  | 0.5527 |  |  |
| SPVAR | -10.1435 | 26.2871 | 0.0060 | 0.3602 | 0.001 |  |  |  | 0.0950 |  |
| EXVAR | -11.7885 | 29.5770 | 0.0012 | 0.1469 | 0.001 |  |  |  |  | 1.0016 |
| BOTHVAR | -10.1366 | 28.2733 | 0.0022 | 0.3584 | 0.001 |  |  |  | 0.0948 | 0.0010 |
| **NS** |  |  |  |  |  |  |  |  |  |  |
| Pure Birth | -15.0651 | 32.1301 | 0.0199 | 0.1319 | 0 |  |  |  |  |  |
| Birth–Death | -15.0651 | 34.1301 | 0.0073 | 0.1319 | 0 |  |  |  |  |  |
| yule2rate | -10.5477 | 27.0954 | 0.2467 | 0.1915 | 0 | 0.0334 | 2.6821 |  |  |  |
| DDL | -10.6425 | 25.2849 | 0.6100 | 0.3722 | 0 |  |  |  | 24.5411 |  |
| DDX | -12.6226 | 29.2452 | 0.0842 | 0.7498 | 0 |  |  | 0.7024 |  |  |
| SPVAR | -12.9945 | 31.9890 | 0.0214 | 0.3775 | 0.001 |  |  |  | 0.1154 |  |
| EXVAR | -15.0843 | 36.1685 | 0.0026 | 0.1324 | 0.001 |  |  |  |  | 1.0018 |
| BOTHVAR | -12.9883 | 33.9766 | 0.0079 | 0.3756 | 0.001 |  |  |  | 0.1152 | 0.0010 |
