# Supplementary figures and images for: Phylogenetic surveys on the newt genus Tylototriton sensu lato (Salamandridae, Caudata) reveal cryptic diversity and novel diversification promoted by historical climatic shifts
Source: PeerJ. 2018 Mar 12;6:e4384. doi: 10.7717/peerj.4384 (PMC5853667; doi:10.7717/peerj.4384)

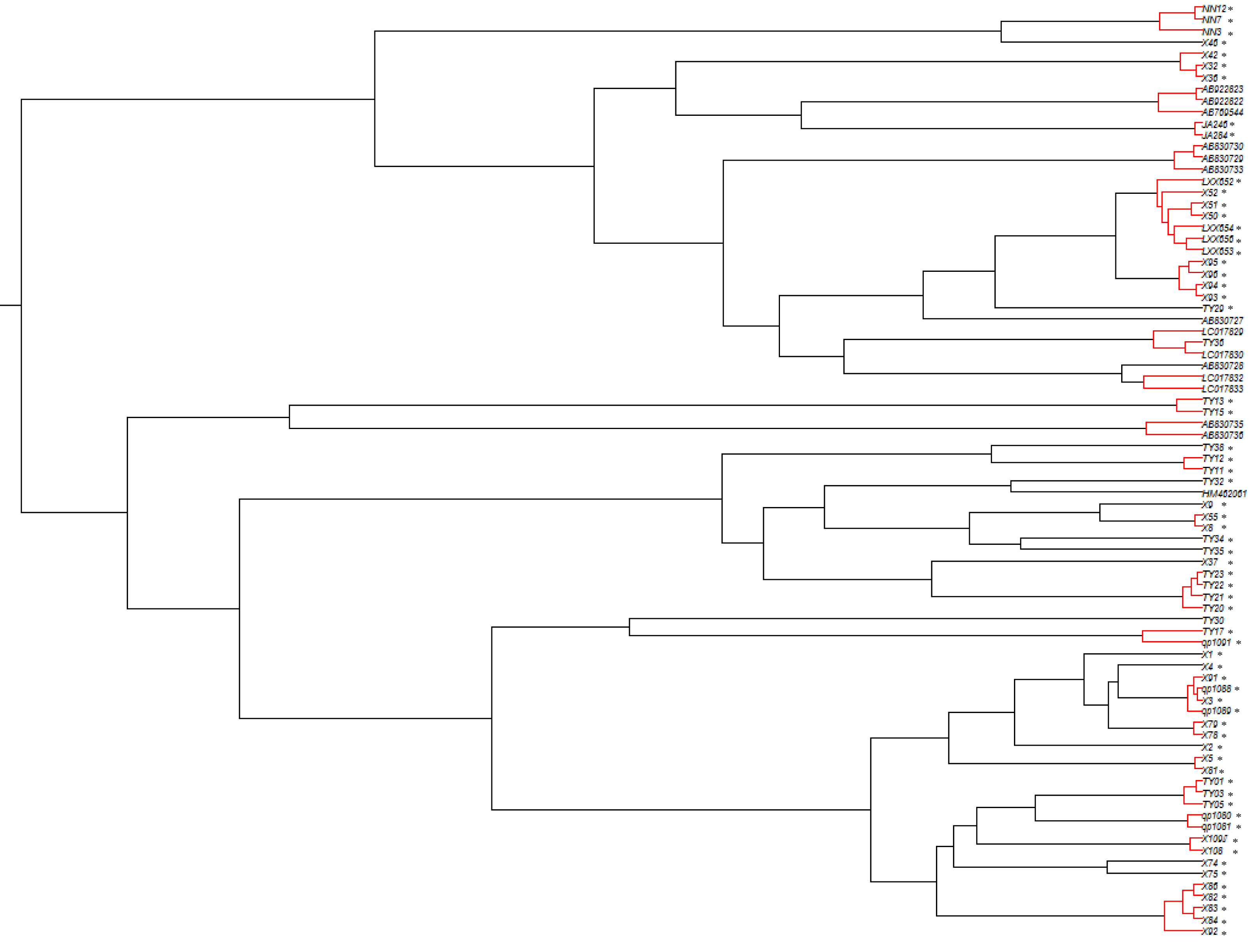

Supplement: Figure S1 — Red shading branches indicate GMYC clusters. “*” individuals were used in *BEAST analyses. [file peerj-06-4384-s004.png]

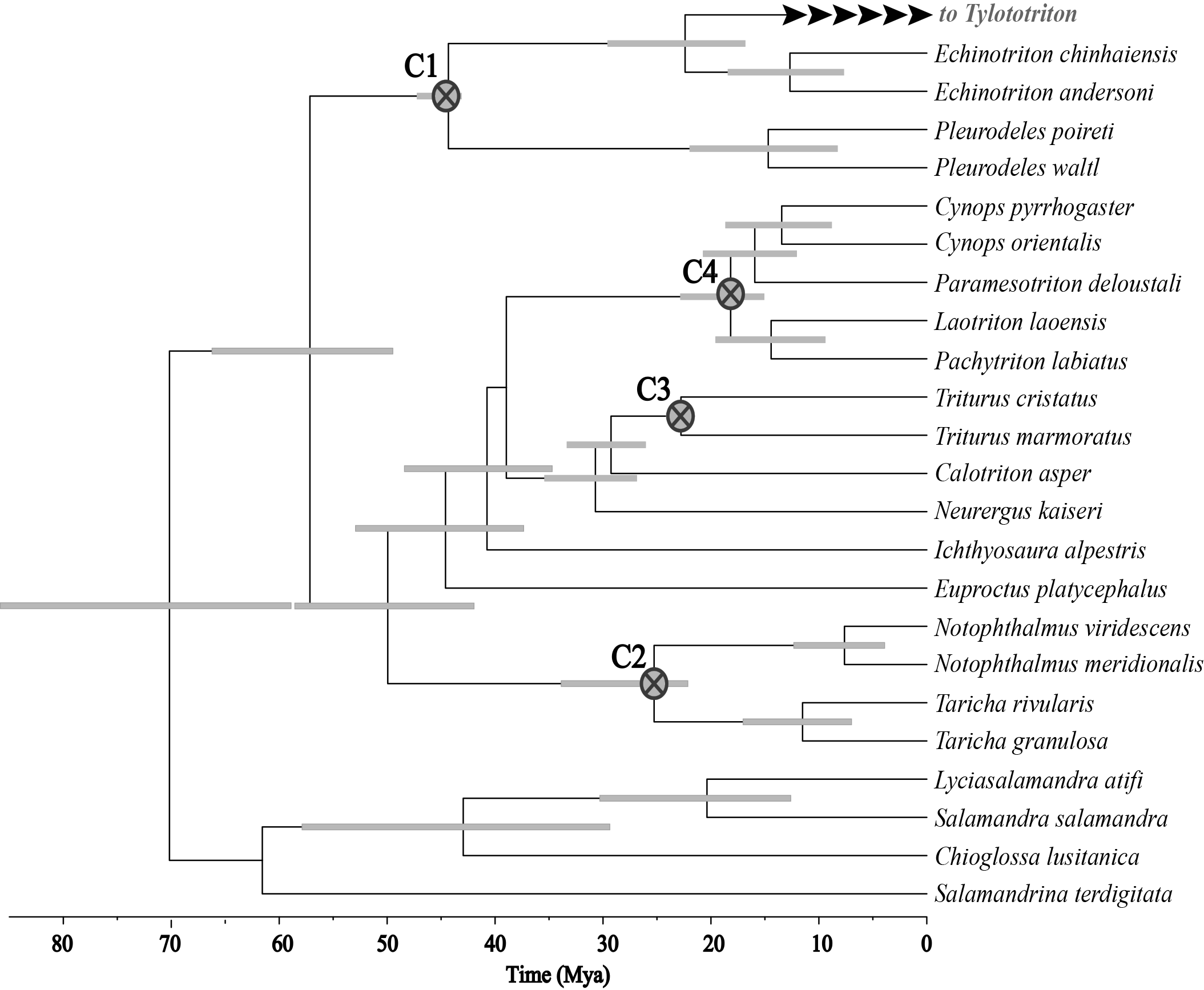

Supplement: Figure S2 — The tree was resulted from the topology-constrained Beast analysis. Fossil calibrations C1–C4 were given in materials for details. Grey bars at nodes show 95% highest posterior density intervals of divergence times. [file peerj-06-4384-s005.png]

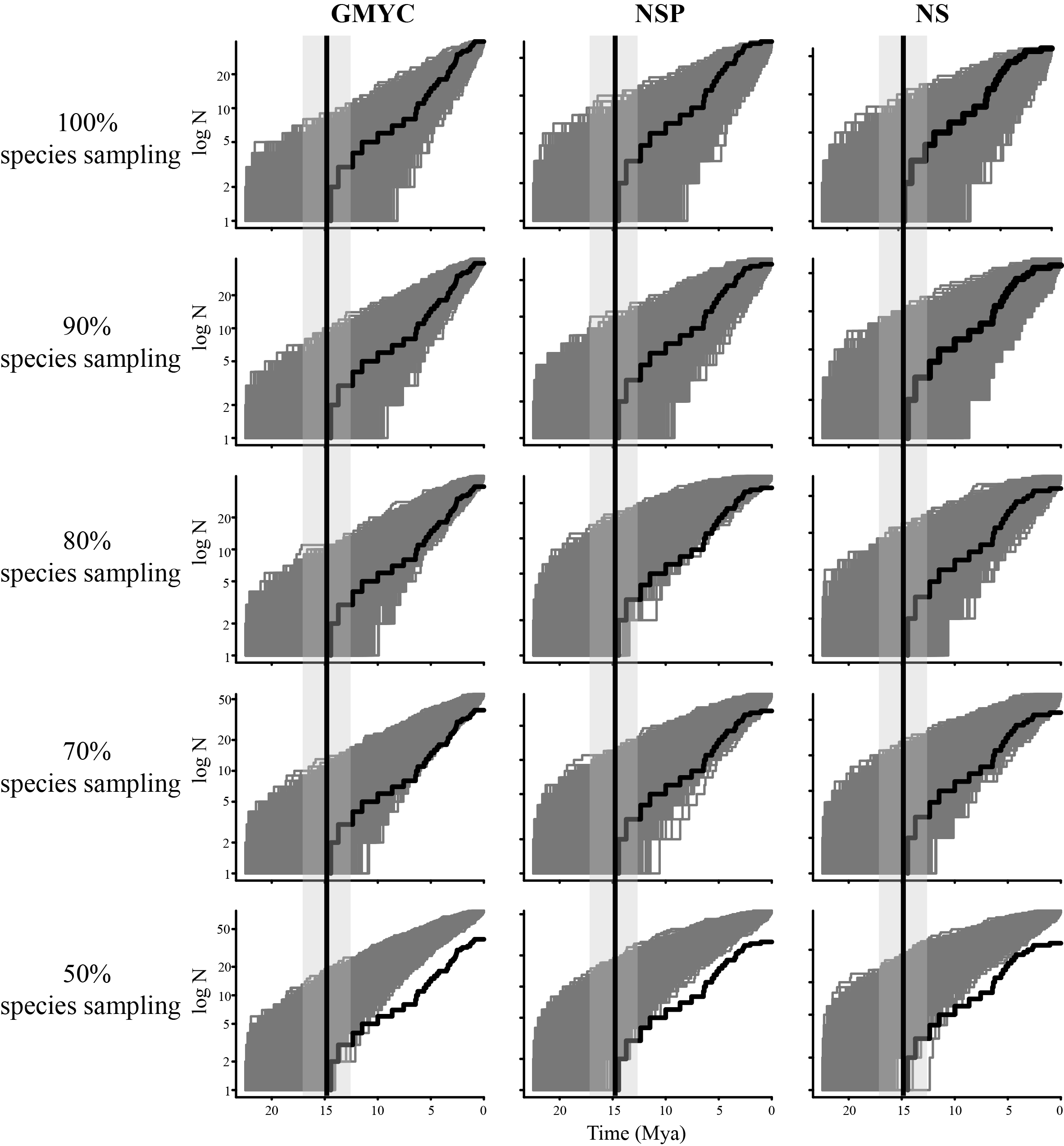

Supplement: Figure S3 — Different lineage delimitating strategies: GMYC, 39 statistically inferred coalescent lineages using the Generalized Mixed Yule Coalescent method; NS, 23 currently nominal species; and NSP, 23 NS plus four independent lineages representing putative cryptic species. Trees were simulated under the observed fixed time since origin (stem age = 22.6 Ma) using the constant rate birth–death process, with speciation and extinction rate values estimated from the empirical data (see Table S3 for different delimitating strategies). Simulations run under full sampling scenario, 10% missing extant lineage, 20% missing extant lineages, 30% missing extant lineages and 30% missing extant lineages. Thick bars correspond to the observed age of the common ancestor of extant Tylototriton s.l., with grey rectangles representing the 95% highest posterior density (HPD) interval. [file peerj-06-4384-s006.png]
