# Supplementary material for: Phylogenetic surveys on the newt genus Tylototriton sensu lato (Salamandridae, Caudata) reveal cryptic diversity and novel diversification promoted by historical climatic shifts
Source: PeerJ. 2018 Mar 12;6:e4384. doi: 10.7717/peerj.4384 (PMC5853667; doi:10.7717/peerj.4384)
Supplement: Data S1 [file peerj-06-4384-s007.docx]

Gene Partition:

DNA, 16s = 1-433

DNA, ND2 = 434-1468

DNA, NCX1 = 1469-2116

DNA, BDNF = 2117-2617

Combined sequences in this study (Combined sequence ID refer to Table S1):

>X1

CAACGGCCGCGGTATCATGACCGTGCAAAGGTAGCGTAATCACTTGTCTTTTAAATAAAG

ACCCGTATGAAAGGCAAAACGAAAGTTCGACTGTCTCTTTAATCCAATCAATGAAATTTA

TCTTTTCGTGCAGAGGCGGAGATAAATATATAAGACGAGAAGACCCTATGGAGCTTTAAA

CACACTATAATATAAGTTTTGGGTTGGGGCGACCACGGAGCAAAAAAATCCTCCGAGATA

AGAATAACACTTCGAAAATTAAAACATTTTGATCCACTAAGTGACCAACGAACCAAGTTA

CCCTAGGGATAACAGCGCAATCCTTTCTAAGAGTCCATATCGACGAATGGGTTTACGACC

TCGATGTTGGATCAGGACACCCAAATGGTGCAGCCGCTGTTAAGGTTCGTTTGTTCAACG

ATTAAAGTCCTACATGAACCCATATGCACTCTCAATTCTATTATCAAGCCTGGCAGTAGG

AACAATTACAACTCTTTCAAGCTCCCACTGATTTCTCGCATGACTAGGCTTAGAAATTAA

TACACTAGCAATCATCCCCTTAATAACAAAAATACATCACCCACGAGCAACAGAATCAGC

CACAAAATACTTCCTGACGCAAGCCACCGCATCAGCCCTAATCTTATTTTCTACAATCAC

AAACGCATGGGCCACAGGAGAATGAACAATTATCACCATAAATGACAACACATCAACACT

TATCTTAACAATCGCCCTAGCCATTAAACTGGGAATCGCCCCATTTCACCTGTGATTACC

AGACGTCATACAAGGACTAAACCTAGCAACATGTTTAATCCTTACAACATGACAAAAACT

AGCCCCAATATCATTAATAATCATAACTAGCCATCAATTAAATGCAAACCTGCTAATTAC

AATAGCCATACTATCAACAGTAATTGGGGGGTGGGGGGGCCTTAACCAAACACAAATACG

AAAAATAATAGCATATTCATCTATCGCCCACCTCGGATGAATAACACTAATCCTGTCTTT

CTCCCCAAACTTAACGACCCTAAACTTAATAATTTACCTAATACTAACCTCATCAATATT

TTTAATAATAATAACCCTAACCTCAACAAATATAAACAAACTTTCAACATCTTGACTGAA

AACGCCACTACTAGCATCATCAATAATAGTTACACTCATAGCCCTGGGAGGCCTACCCCC

AACCTCAGGCTTCTTACCAAAATGACTGATCCTACAAGAAATAACTAAACAACACCTAGG

AGTCGTATCTACCCTAATAGCTATATCTGCCCTACTAAGCCTATTCTTTTATTTACGACT

ATCATATGCAATCTCACTAACCACTGCGCCAAACACCTCAAACTCCAACATAACCTGACG

AGTAGCCCACAACCAAATACAAGTCCTCCCAACAATAATCACACTAACTACAATAATACT

ACCAATCACACCAACACTATTAGCAACAGGACAAGGCAGCTTGGCGTACCCAGGTCTGAG

GACCCACGGGACTTTGGAGAGCATAGGTGGGCCTAAAGGCAGTTCAAGAGGAGGGTTGCC

CTCACTAGCAGACACTTTTGAACAAGTCATTGAGGAGATGCTTGAAGATGAGCAGAGCAT

GAGGCCCAGTGAGGAGAACAAGGATGCCGACATGTTTACATCTCGCGTAATGCTGAGCAG

TCAAGTGCCTTTGGAGCCTCCTCTTCTCTTTCTGCTGGAGGAGTACAAAAACTACTTGGA

TGCTGCAAATATGTCCATGAGGGTCCGACGTCACTCTGACCCCGCCCGCCGCGGGGAGCT

GAGTGTGTGTGACAGTATTAGTGAGTGGGTGACGGCATCTGACAAAAAGACTGCCGTGGA

CATGTCGGGGCAGACGGTAACTGTTCTAGAAAAAGTTCCAGTACCCAAAGGCCAACTGAA

GCAATACTTCTACGAGACCAAATGCAACCCTATGGGTTACATGAAGGAGGGCTGCAGAGG

CATTGACAAGAGGTACTGGAACTCCCAGTGCCGAACTACTCAGTCGTATGTGCGCGCGCT

CACCATGGATAACAAGAAGAAAGTGGGCTGGCGGTTTATCAGAATAGACACTTCATGTGT

ATGTACATTGACCATTGTTTGCTTGGGTTGCAGACCGAAGGCTGTTGTTCTACAAATATG

TCTACAAGCGATATAGAGCTGGGAAGCAAAGGGGTATGATTATAGAAACCGAAGGTGACA

GGCCTTCTTCAAAAGCTGACATTGAAATGGATGGAAAGATGGTCAACTCTCATACTGAAA

ATTTCATTGATGGCTCTCTAGTTCTGGAAGTCGATGAGAAAGATCAGGATGATGAAGAAG

CCAGGCGCGATATGGCCAGAATCCTAAAGGAGCTAAAGCAGAAACATCCTGACAAGGAAA

TAGAGCAACTGATTGAGTTAGCGAACTACCAGGTCTTGAGTCAACAGCAAAAAAGTCGAG

CATTCTATCGTATTCAAGCTACTCGCTTGATGACTGGGGCCGGAAATATTCTAAAGAGAC

ATGCAGCAGACCAGGCAAGGAAAGCTGTGAGCATGCAGGAAGTTAACTCTGAAGTGATCG

AAAATGAGCCCGTCAGTAAGATCTATTTCGAGCAAGC

>X2

CAACGGCCGCGGTATCATGACCGTGCAAAGGTAGCGTAATCACTTGTCTTTTAAATAAAG

ACCCGTATGAAAGGCAAAACGAAAGTTCAACTGTCTCTTTAATCCAATCAATGAAATTTA

TCTTTTCGTGCAGAGGCGGAGATAAATATATAAGACGAGAAGACCCTATGGAGCTTTAAA

CACACTATAGTATAAGTTTTGGGTTGGGGCGACCGCGGAGCAAAAAAATCCTCCGAGATA

AGAATAACACTTCGAAAATTAAAACATTTTGATCCACTAAGTGACCAACGAACCAAGTTA

CCCTAGGGATAACAGCGCAATCCTTTCTAAGAGTCCATATCGACGAATGGGTTTACGACC

TCGATGTTGGATCAGGACACCCAAATGGTGCAGCCGCTGTTAAGGTTCGTTTGTTCAACG

ATTAAAGTCCTACATGAACCCATATGCACTCTCAATTCTATTATCAAGCCTGGCAGTAGG

AACAATTACAACTCTCTCAAGCTCCCACTGATTTCTCGCATGACTAGGCTTAGAAATTAA

TACACTAGCAATCATCCCCCTGATAACAAAAATACACCACCCACGAGCAACAGAATCAGC

CACAAAATACTTCCTAACGCAAGCCACCGCATCAGCCCTAATCTTATTTTCTACAATCAC

AAACGCATGGGCCACAGGAGAATGAACAATTATCACCATAAATGACAACACATCAACACT

TATCTTAACAATCGCCCTAGCCACCAAACTGGGAATCGCCCCATTTCATCTGTGATTACC

AGACGTCATACAAGGACTAAACCTAGCAACATGTTTAATCCTTACAACATGGCAAAAACT

AGCCCCAATATCATTAATAATCATAACTAGCCATCAATTAAACACAAACCTACTAATTAC

AATAGCCATACTATCAACAGTAATTGGGGGGTGGGGGGGCCTTAACCAAACACAAATACG

AAAAATAATAGCATATTCATCTATCGCCCACCTCGGATGAATAACACTAATCCTATCTTT

CTCCCCAAACTTAACGACCCTAAACTTAATAATTTACCTAATACTAACCTCATCAATATT

TTTAATAATAATAACCATAACCTCAACAAATATAAACAAACTTTCAACATCTTGACTGAA

AACGCCACTACTAGCATCATCAATAATAGTTACACTCATAGCCCTTGGAGGCCTACCCCC

AACCTCAGGCTTCTTACCAAAATGACTAATCCTACAAGAAATAACTAAACAACACCTAGT

AGCCGTATCTACACTAATAGCTATATCTGCCCTACTAAGCCTTTTCTTCTATTTACGACT

ATCATATGCGATCTCACTAACCACTGCGCCAAACACCTCAAACTCCAACATAACCTGACG

AGTAGCCCACAACCAAATACAAGTCCTCCCAACAATAATCACACTAACTACAATAATACT

ACCAATCACACCAACACTACTAGCAACAGGACAAGGCAGCTTGGCGTACCCAGGTCTGAG

GACCCACGGGACTTTGGAGAGCATAGGTGGGCCTAAAGGCAGTTCAAGAGGAGGGTTGCC

CTCACTAGCAGACACTTTTGAACAAGTCATTGAGGAGATGCTTGAAGATGAGCAGAGCAT

GAGGCCCAGTGAGGAGAACAAGGATGCCGACATGTTTACATCTCGCGTAATGCTGAGCAG

TCAAGTGCCTTTGGAGCCTCCTCTTCTCTTTCTGCTGGAGGAGTACAAAAACTACTTGGA

TGCTGCAAATATGTCCATGAGGGTCCGACGTCACTCTGACCCCGCCCGCCGCGGGGAGCT

GAGTGTGTGTGACAGTATTAGTGAGTGGGTGACGGCATCTGACAAAAAGACTGCCGTGGA

CATGTCGGGGCAGACGGTAACTGTTCTAGAAAAAGTTCCAGTACCCAAAGGCCAACTGAA

GCAATACTTCTACGAGACCAAATGCAACCCTATGGGTTACATGAAGGAGGGCTGCAGAGG

CATTGACAAGAGGTACTGGAACTCCCAGTGCCGAACTACTCAGTCGTATGTGCGCGCGCT

CACCATGGATAACAAGAAGAAAGTGGGCTGGCGGTTTATCAGAATAGACACTTCATGTGT

ATGTACATTGACCATTGTTTGCTTGGGTTGCAGACCGAAGGCTGTTGTTCTACAAATATG

TCTACAAGCGATATAGAGCTGGGAAGCAAAGGGGTATGATTATAGAAACCGAAGGTGACA

GGCCTTCTTCAAAAGCTGACATTGAAATGGATGGAAAGATGGTCAACTCTCATACTGAAA

ATTTCATTGATGGCTCTCTAGTTCTGGAAGTCGATGAGAAAGATCAGGATGATGAAGAAG

CCAGGCGCGATATGGCCAGAATCCTAAAGGAGCTAAAGCAGAAACATCCTGACAAGGAAA

TAGAGCAACTGATTGAGTTAGCGAACTACCAGGTCTTGAGTCAACAGCAAAAAAGTCGAG

CATTCTATCGTATTCAAGCTACTCGCTTGATGACTGGGGCCGGAAATATTCTAAAGAGAC

ATGCAGCAGACCAGGCAAGGAAAGCTGTGAGCATGCAGGAAGTTAACTCTGAAGTGATCG

AAAATGAGCCCGTCAGTAAGATCTATTTCGAGCAAGC

>X3

CAACGGCCGCGGTATCATGACCGTGCAAAGGTAGCGTAATCACTTGTCTTTTAAATAAAG

ACCCGTATGAAAGGCAAAACGAAAGTTCGACTGTCTCTTTAATCCAATCAATGAAATTTA

TCTTTTCGTGCAGAGGCGGAGATAAATATATAAGACGAGAAGACCCTATGGAGCTTTAAA

CACACTATAATATAAGTTTTAGGTTGGGGCGACCACGGAGCAAAAAAATCCTCCGAGATA

AGAATAACACTTCGAAAATTAAAACATTTTGATCCACTAAGTGACCAACGAACCAAGTTA

CCCTAGGGATAACAGCGCAATCCTTTCTAAGAGTCCATATCGACGAATGGGTTTACGACC

TCGATGTTGGATCAGGACACCCAAATGGTGCAGCCGCTGTTAAGGTTCGTTTGTTCAACG

ATTAAAGTCCTACATGAACCCATATGCACTCTCAATTCTATTATCAAGCCTGGCAGTAGG

AACAATTACAACTCTTTCAAGCTCCCACTGATTTCTCGCATGACTAGGCTTAGAAATAAA

TACACTAGCAATCATCCCCCTAATAACAAAAATACATCACCCACGAGCAACAGAATCAGC

CACAAAATACTTCCTAACGCAAGCCGCCGCATCAGCCCTAATCTTATTTTCTACAATCAC

AAACGCATGGGCCACAGGAGAATGAACAATTATCACCATAAATGACAACACATCAACACT

TATCTTAACAATCGCCCTAGCCATTAAACTGGGAATCGCCCCATTTCACCTGTGATTACC

AGACGTCATACAAGGGCTAAACCTAGCAACATGTTTAATCCTTACAACATGACAAAAACT

AGCCCCAATATCATTAATAATCATAACTAGCCATCAATTAAACGCAAACCTACTAATTAC

AATAGCCATACTATCAACAGTAATTGGGGGGTGGGGGGGCCTTAACCAAACACAAATCCG

AAAAATAATAGCATATTCATCTATCGCTCACCTCGGATGAATAACACTTATCCTGTCTTT

CTCCCCAAACTTAACGACCCTAAACTTAATAATTTACCTAATACTAACCTCATCAATATT

TTTAATAATAATAACCCTAACCTCAACAAATATAAACAAACTTTCAACATCTTGACTGAA

AACGCCACTACTAGCATCATCAATAATAATTACACTCATAGCCCTGGGAGGCCTACCCCC

AACCTCAGGCTTCTTACCAAAATGATTGATCCTACAAGAAATAACTAAACAACACCTAGG

AGCCGTATCTACACTAATAGCTATATCTGCCCTACTAAGCCTATTCTTCTATTTACGACT

ATCATATGCAATCTCACTAACCACTGCACCAAACACCTCAAACTCCAACATAACCTGACG

AGTAACCCACAACCAAATACAAGTCCTCCCAACAATAATCACACTAACTACAATTATACT

ACCAATCACACCAACACTATTAGCAACAGGACAAGGCAGCTTGGCGTACCCAGGTCTGAG

GACCCACGGGACTTTGGAGAGCATAGGTGGGCCTAAAGGCAGTTCAAGAGGAGGGTTGCC

CTCACTAGCAGACACTTTTGAACAAGTCATTGAGGAGATGCTTGAAGATGAGCAGAGCAT

GAGGCCCAGTGAGGAGAACAAGGATGCGGACATGTTTACATCTCGCGTAATGCTGAGCAG

TCAAGTGCCTTTGGAGCCTCCTCTTCTCTTTCTGCTGGAGGAGTACAAAAACTACTTGGA

TGCTGCAAATATGTCCATGAGGGTCCGACGTCACTCTGACCCCGCCCGCCGCGGGGAGCT

GAGTGTGTGTGACAGTATTAGTGAGTGGGTGACGGCATCTGACAAAAAGACTGCCGTGGA

CATGTCGGGGCAGACGGTAACTGTTCTAGAAAAAGTTCCAGTACCCAAAGGCCAACTGAA

GCAATACTTCTACGAGACCAAATGCAACCCTATGGGTTACATGAAGGAGGGCTGCAGAGG

CATTGACAAGAGGTACTGGAACTCCCAGTGCCGAACTACTCAGTCGTATGTGCGCGCGCT

CACCATGGATAACAAGAAGAAAGTGGGCTGGCGGTTTATCAGAATAGACACTTCATGTGT

ATGTACATTGACCATTGTTTGCTTGGGTTGCAGACCGAAGGCTGTTGTTCTACAAATATG

TCTACAAGCGATATAGAGCTGGGAAGCAAAGGGGTATGATTATAGAAACCGAAGGTGACA

GGCCTTCTTCAAAAGCTGACATTGAAATGGATGGAAAGATGGTCAACTCTCATACTGAAA

ATTTCATTGATGGCTCTCTAGTTCTGGAAGTCGATGAGAAAGATCAGGATGATGAAGAAG

CCAGGCGCGATATGGCCAGAATCCTAAAGGAGCTAAAGCAGAAACATCCTGACAAGGAAA

TAGAGCAACTGATTGAGTTAGCGAACTACCAGGTCTTGAGTCAACAGCAAAAAAGTCGAG

CATTCTATCGTATTCAAGCTACTCGCTTGATGACTGGGGCCGGAAATATTCTAAAGAGAC

ATGCAGCAGACCAGGCAAGGAAAGCTGTGAGCATGCAGGAAGTTAACTCTGAAGTGATCG

AAAATGAGCCCGTCAGTAAGATCTATTTCGAGCAAGC

>X4

CAACGGCCGCGGTATCATGACCGTGCAAAGGTAGCGTAATCACTTGTCTTTTAAATAAAG

ACCCGTATGAAAGGCAAAACGAAAGTTCGACTGTCTCTTTAATCCAATCAATGAAATTTA

TCTTTTCGTGCAGAGGCGGAGATAAATATATAAGACGAGAAGACCCTATGGAGCTTTAAA

CACACTATAATATAAGTTTTGGGTTGGGGCGACCACGGAGCAAAAAAATCCTCCGAGATA

AGAATAACACTTCGAAAATTAAAACATTTTGATCCACTAAGTGACCAACGAACCAAGTTA

CCCTAGGGATAACAGCGCAATCCTTTCTAAGAGTCCATATCGACGAATGGGTTTACGACC

TCGATGTTGGATCAGGACACCCAAATGGTGCAGCCGCTGTTAAGGTTCGTTTGTTCAACG

ATTAAAGTCCTACATGAACCCATATGCACTCTCAATTCTATTATCAAGCCTGGCAGTAGG

AACAATTACAACTCTTTCAAGCTCCCACTGATTTCTCGCATGACTAGGCTTAGAAATAAA

CACACTAGCAATCATCCCCTTAATAACAAAAATACATCACCCACGAGCAACAGAATCAGC

CACAAAATACTTCCTGACACAAGCCGCCGCATCAGCCCTAATCTTATTTTCTACAATCAC

AAACGCATGGGCCACAGGAGAATGAACAATTATCACCATAAATGACAACACATCAACACT

TATCTTAACAATCGCCCTAGCCATTAAACTGGGAATCGCCCCATTTCACCTGTGATTACC

AGACGTTATACAAGGACTAAACCTAGCAACATGTTTAATCCTTACAACATGACAAAAACT

AGCCCCAATATCATTAATAATCATAACTAGCCATCAATTAAACGCAAACCTACTAATTAC

AATAGCCACACTATCAACAGTAATTGGGGGGTGGGGGGGCCTTAACCAAACACAAATCCG

AAAAATAATAGCATATTCATCTATCGCCCACCTCGGATGAATAACACTAATTTTGTCTTT

CTCCCCAAACTTAACGACCCTAAACTTAATAATTTACCTAATACTAACCTCATCAATATT

TTTAATAATAATAACCCTAACCTCAACAAATATAAACAAACTTTCAACATCTTGACTGAA

AACGCCACTACTAGCATCATCAATAATAGTTACACTCATAGCCCTGGGAGGCCTACCTCC

AACCTCAGGATTCTTACCAAAATGATTGATCCTACAAGAAATAACTAAACAACACCTAGG

AGCCGTATCTACACTAATAGCTATATCTGCCCTACTAAGCCTATTCTTCTATTTACGACT

ATCATATGCAATCTCATTAACCACTGCGCCAAACACCTCAAACTCCAACATAACCTGACG

AGTAGCCCACAACCAAATACAAATCCTACCAACGATAATCACACTAACTACAATAATACT

ACCAATCACACCAACACTATTAGCAACAGGACAAGGCAGCTTGGCGTACCCAGGTCTGAG

GACCCACGGGACTTTGGAGAGCATAGGTGGGCCTAAAGGCAGTTCAAGAGGAGGGTTGCC

CTCACTAGCAGACACTTTTGAACAAGTCATTGAGGAGATGCTTGAAGATGAGCAGAGCAT

GAGGCCCAGTGAGGAGAACAAGGATGCGGACATGTTTACATCTCGCGTAATGCTGAGCAG

TCAAGTGCCTTTGGAGCCTCCTCTTCTCTTTCTGCTGGAGGAGTACAAAAACTACTTGGA

TGCTGCAAATATGTCCATGAGGGTCCGACGTCACTCTGACCCCGCCCGCCGCGGGGAGCT

GAGTGTGTGTGACAGTATTAGTGAGTGGGTGACGGCATCTGACAAAAAGACTGCCGTGGA

CATGTCGGGGCAGACGGTAACTGTTCTAGAAAAAGTTCCAGTACCCAAAGGCCAACTGAA

GCAATACTTCTACGAGACCAAATGCAACCCTATGGGTTACATGAAGGAGGGCTGCAGAGG

CATTGACAAGAGGTACTGGAACTCCCAGTGCCGAACTACTCAGTCGTATGTGCGCGCGCT

CACCATGGATAACAAGAAGAAAGTGGGCTGGCGGTTTATCAGAATAGACACTTCATGTGT

ATGTACATTGACCATTGTTTGCTTGGGTTGCAGACCGAAGGCTGTTGTTCTACAAATATG

TCTACAAGCGATATAGAGCTGGGAAGCAAAGGGGTATGATTATAGAAACCGAAGGTGACA

GGCCTTCTTCAAAAGCTGACATTGAAATGGATGGAAAGATGGTCAACTCTCATACTGAAA

ATTTCATTGATGGCTCTCTAGTTCTGGAAGTCGATGAGAAAGATCAGGATGATGAAGAAG

CCAGGCGCGATATGGCCAGAATCCTAAAGGAGCTAAAGCAGAAACATCCTGACAAGGAAA

TAGAGCAACTGATTGAGTTAGCGAACTACCAGGTCTTGAGTCAACAGCAAAAAAGTCGAG

CATTCTATCGTATTCAAGCTACTCGCTTGATGACTGGGGCCGGAAATATTCTAAAGAGAC

ATGCAGCAGACCAGGCAAGGAAAGCTGTGAGCATGCAGGAAGTTAACTCTGAAGTGATCG

AAAATGAGCCCGTCAGTAAGATCTATTTCGAGCAAGC

>X5

CAACGGCCGCGGTATCATGACCGTGCAAAGGTAGCGTAATCACTTGTCTTTTAAATAAAG

ACCCGTATGAAAGGCAAAACGAAAGTTCAACTGTCTCTTTAATCCAATCAATGAAATTTA

TCTTTTCGTGCAGAGGCGGAAATAAATATATAAGACGAGAAGACCCTATGGAGCTTTAAA

CACACTATAATACAAGTTTTGGGTTGGGGCGACCACGGAGCAAAAAAATCCTCCGAGATA

AGAATAACACTTCAAAAATTAAAACATTTTGATCCACTAAGTGACCAACGAACCAAGTTA

CCCTAGGGATAACAGCGCAATCCTTTCTAAGAGTCCATATCGACGAATGGGTTTACGACC

TCGATGTTGGATCAGGACACCCAAATGGTGCAGCCGCTGTTAAGGTTCGTTTGTTCAACG

ATTAAAGTCCTACATGAACCCATATGCACTATCAATTCTATTATCAAGCCTGGCAGTAGG

GACAATTACAACTCTTTCAAGCTCCCACTGATTCCTCGCATGACTAGGCTTAGAAATTAA

TACATTAGCAATCATCCCCCTGATAACAAAAATACACCACCCACGAGCGACAGAATCAGC

CACAAAATACTTCCTGACGCAAGCTACCGCATCGGCCCTAATCTTATTTTCTACAATCAC

AAACGCATGGGCCACAGGAGAATGAACAATTATTACCATAAATGACAACACATCAACACT

TATCTTAACAATCGCCCTAGCCATTAAACTAGGAATCGCCCCATTTCACCTGTGATTACC

AGACGTCATACAAGGACTAAACCTAGCAACATGTTTAATCCTTACAACATGACAAAAACT

GGCCCCAATATCACTAATAATCATAACTAGCCATCAATTAAACACAAACCTACTAGTTAC

AATAGCCATACTATCAACGGTAATTGGGGGGTGGGGGGGTCTTAACCAAACACAAATACG

AAAAATAATAGCATATTCATCTATTGCCCACCTCGGATGAATAACACTAATCCTGTCTTT

CTCCCCAAACTTAACAACCCTAAACTTAATAATTTACCTAATACTAACCTCATCAATATT

TTTAATAATAATAACCCTAACCTCAACAAATATAAACAAACTTTCAACATCTTGACTGAA

AACACCACTTCTAGCATCATCAATAATAATTACACTCATAGCCCTAGGAGGCCTACCCCC

AACCTCAGGCTTCTTACCAAAATGACTGATCCTACAAGAAATAACTAAACAACACCTAGG

AGCCGTATCTACACTAATAGCCATATCTGCCCTACTAAGCCTATTCTTCTATTTACGACT

ATCATATGCGATCTCACTAACCACTGCACCAAACACCTCAAACTCCAACATAACCTGACG

AGTGGCCCACAACCAAATACAAGTCCTCCCAACAATAATTACACTGACTACAGTAATACT

ACCAATCACACCAACACTACTAGCAACAGGACAAGGCAGCTTGGCGTACCCAGGTCTGAG

GACCCACGGGACTTTGGAGAGCATAGGTGGGCCTAAAGGCAGTTCAAGAGGAGGGTTGCC

CTCACTAGCAGACACTTTTGAACAAGTCATTGAGGAGATGCTTGAAGATGAGCAGAGCAT

GAGGCCCAGTGAGGAGAACAAGGATGCGGACATGTTTACATCTCGCGTAATGCTGAGCAG

TCAAGTGCCTTTGGAGCCTCCTCTTCTCTTTCTGCTGGAGGAGTACAAAAACTACTTGGA

TGCTGCAAATATGTCCATGAGGGTCCGACGTCACTCTGACCCCGCCCGCCGCGGGGAGCT

GAGTGTGTGTGACAGTATTAGTGAGTGGGTGACGGCATCTGACAAAAAGACTGCCGTGGA

CATGTCGGGGCAGACGGTAACTGTTCTAGAAAAAGTTCCAGTACCCAAAGGCCAACTGAA

GCAATACTTCTACGAGACCAAATGCAACCCTATGGGTTACATGAAGGAGGGCTGCAGAGG

CATTGACAAGAGGTACTGGAACTCCCAGTGCCGAACTACTCAGTCGTATGTGCGCGCGCT

CACCATGGATAACAAGAAGAAAGTGGGCTGGCGGTTTATCAGAATAGACACTTCATGTGT

ATGTACATTGACCATTGTTTGCTTGGGTTGCAGACCGAAGGCTGTTGTTCTACAAATATG

TCTACAAGCGATATAGAGCTGGGAAGCAAAGGGGTATGATTATAGAAACCGAAGGTGACA

GGCCTTCTTCAAAAGCTGACATTGAAATGGATGGAAAGATGGTCAACTCTCATACTGAAA

ATTTCATTGATGGCTCTCTAGTTCTGGAAGTCGATGAGAAAGATCAGGATGATGAAGAAG

CCAGGCGCGATATGGCCAGAATCCTAAAGGAGCTAAAGCAGAAACATCCTGACAAGGAAA

TAGAGCAACTGATTGAGTTAGCGAACTACCAGGTCTTGAGTCAACAGCAAAAAAGTCGAG

CATTCTATCGTATTCAAGCTACTCGCTTGATGACTGGGGCCGGAAATATTCTAAAGAGAC

ATGCAGCAGACCAGGCAAGGAAAGCTGTGAGCATGCAGGAAGTTAACTCTGAAGTGATCG

AAAATGAGCCCGTCAGTAAGATCTATTTCGAGCAAGC

>X7

CAACGGCCGCGGTATCATGACCGTGCAAAGGTAGCGTAATCACTTGTCTTTTAAATAAAG

ACCCGTATGAAAGGCAAAACGAAAGTTCAACTGTCTCTTTAATCCAATCAATGAAATTTA

TCTTTTCGTGCAGAGGCGGAAATAAATATATAAGACGAGAAGACCCTATGGAGCTTTAAA

CACACTATAATACAAGTTTTGGGTTGGGGCGACCACGGAGCAAAAAAATCCTCCGAGATA

AGAATAACACTTCAAAAATTAAAACATTTTGATCCACTAAGTGACCAACGAACCAAGTTA

CCCTAGGGATAACAGCGCAATCCTTTCTAAGAGTCCATATCGACGAATGGGTTTACGACC

TCGATGTTGGATCAGGACACCCAAATGGTGCAGCCGCTGTTAAGGTTCGTTTGTTCAACG

ATTAAAGTCCTACATGAACCCATATGCACTATCAATTCTATTATCAAGCCTGGCAGTAGG

GACAATTACAACTCTTTCAAGCTCCCACTGATTCCTCGCATGACTAGGCTTAGAAATTAA

TACATTAGCAATCATCCCCCTGATAACAAAAATACACCACCCACGAGCGACAGAATCAGC

CACAAAATACTTCCTGACGCAAGCTACCGCATCGGCCCTAATCTTATTTTCTACAATCAC

AAACGCATGGGCCACAGGAGAATGAACAATTATTACCATAAATGACAACACATCAACACT

TATCTTAACAATCGCCCTAGCCATTAAACTAGGAATCGCCCCATTTCACCTGTGATTACC

AGACGTCATACAAGGACTAAACCTAGCAACATGTTTAATCCTTACAACATGACAAAAACT

GGCCCCAATATCACTAATAATCATAACTAGCCATCAATTAAACACAAACCTACTAGTTAC

AATAGCCATACTATCAACGGTAATTGGGGGGTGGGGGGGTCTTAACCAAACACAAATACG

AAAAATAATAGCATATTCATCTATTGCCCACCTCGGATGAATAACACTAATCCTGTCTTT

CTCCCCAAACTTAACAACCCTAAACTTAATAATTTACCTAATACTAACCTCATCAATATT

TTTAATAATAATAACCCTAACCTCAACAAATATAAACAAACTTTCAACATCTTGACTGAA

AACACCACTTCTAGCATCATCAATAATAATTACACTCATAGCCCTAGGAGGCCTACCCCC

AACCTCAGGCTTCTTACCAAAATGACTGATCCTACAAGAAATAACTAAACAACACCTAGG

AGCCGTATCTACACTAATAGCCATATCTGCCCTACTAAGCCTATTCTTCTATTTACGACT

ATCATATGCGATCTCACTAACCACTGCACCAAACACCTCAAACTCCAACATAACCTGACG

AGTGGCCCACAACCAAATACAAGTCCTCCCAACAATAATTACACTGACTACAGTAATACT

ACCAATCACACCAACACTACTAGCAACAGGACAAGGCAGCTTGGCGTACCCAGGTCTGAG

GACCCACGGGACTTTGGAGAGCATAGGTGGGCCTAAAGGCAGTTCAAGAGGAGGGTTGCC

CTCACTAGCAGACACTTTTGAACAAGTCATTGAGGAGATGCTTGAAGATGAGCAGAGCAT

GAGGCCCAGTGAGGAGAACAAGGATGCGGACATGTTTACATCTCGCGTAATGCTGAGCAG

TCAAGTGCCTTTGGAGCCTCCTCTTCTCTTTCTGCTGGAGGAGTACAAAAACTACTTGGA

TGCTGCAAATATGTCCATGAGGGTCCGACGTCACTCTGACCCCGCCCGCCGCGGGGAGCT

GAGTGTGTGTGACAGTATTAGTGAGTGGGTGACGGCATCTGACAAAAAGACTGCCGTGGA

CATGTCGGGGCAGACGGTAACTGTTCTAGAAAAAGTTCCAGTACCCAAAGGCCAACTGAA

GCAATACTTCTACGAGACCAAATGCAACCCTATGGGTTACATGAAGGAGGGCTGCAGAGG

CATTGACAAGAGGTACTGGAACTCCCAGTGCCGAACTACTCAGTCGTATGTGCGCGCGCT

CACCATGGATAACAAGAAGAAAGTGGGCTGGCGGTTTATCAGAATAGACACTTCATGTGT

ATGTACATTGACCATTGTTTGCTTGGGTTGCAGACCGAAGGCTGTTGTTCTACAAATGTG

TCTACAAGCGATATAGAGCTGGGAAGCAAAGGGGTATGATTATAGAAACCGAAGGTGACA

GGCCTTCTTCAAAAGCTGACATTGAAATGGATGGAAAGATGGTCAACTCTCATACTGAAA

ATTTCATTGATGGCTCTCTAGTTCTGGAAGTCGATGAGAAAGATCAGGATGATGAAGAAG

CCAGGCGCGATATGGCCAGAATCCTAAAGGAGCTAAAGCAGAAACATCCTGACAAGGAAA

TAGAGCAACTGATTGAGTTAGCGAACTACCAGGTCTTGAGTCAACAGCAAAAAAGTCGAG

CATTCTATCGTATTCAAGCTACTCGCTTGATGACTGGGGCCGGAAATATTCTAAAGAGAC

ATGCAGCAGACCAGGCAAGGAAAGCTGTGAGCATGCAGGAAGTTAACTCTGAAGTGATCG

AAAATGAGCCCGTCAGTAAGATCTATTTCGAGCAAGC

>X8

CAACGGCCGCGGTATCATGACCGTGCAAAGGTAGCGTAATCACTTGTCTTTTAAATAAAG

ACCTGTATGAAAGGCAAAACGAAAGTTCAACTGTCTCTTTAATCCAATCAATAAAATTTA

TCTTTCCGTGCAGAAGCGGAAATAAATATATAAGACGAGAAGACCCTATGGAGCTTTAAA

CACACCATAATATAAGTTTTAGGTTGGGGCGACCGCGGAAGAAAAAAATCCTCCGAGATA

AGAATAACACTTCGAAAATTAAAACATTTTGATCCACTAAGTGACCAACGAACCAAGTTA

CCCTAGGGATAACAGCGCAATCCTTTCTAAGAGTCCATATCGACGAACGGGTTTACGACC

TCGATGTTGGATCAGGACACCCAAATGGTGCAGCCGCTATTAAGGTTCGTTTGTTCAACG

ATTAAAGTCCTACATGAACCCATATGCACTGTCAATTCTATTGTCGAGCCTAGCAGTAGG

AACAATCACAACCCTTTCAAGCTCCCATTGATTCCTCGCATGAGTGGGCCTAGAAATTAA

TACACTAGCAATCATCCCGCTAATAACAAAAATACACCACCCACGAGCAACGGAATCAGC

CACAAAATACTTTCTAACGCAAGCCACCGCATCAGCCCTAATCTTATTTTCCACAATCAC

AAACGCATGGGCCACAGGAGAATGAACAATTATTACCATAAATGACAACACGTCAACATT

TATCTTAACAATTGCCCTGGCCATCAAACTGGGAATCGCCCCATTTCACCTCTGATTACC

AGACGTTATACAAGGATTAACCCTAACAACATGTCTAATCCTTACAACATGACAAAAATT

AGCCCCAATATCCTTAATAATTATAACCGGCCATCAATTAAACACAAACCTACTAATTAC

AATGGCCATATTATCAACAGTACTTGGGGGGTGGGGGGGCCTTAACCAAACACAAATGCG

AAAAATAATAGCATATTCATCTATTGCCCACCTCGGATGAATAACCCTAATCCTATCCTT

CTCCCCAAGCTTAACAACATTAAACCTAGTAATCTACCTAATACTAACCTCATCAATATT

CTTAATAATAATAACAATAACCTCAACAAATATAAATAAACTCTCAACATCTTGACTGAA

GACCCCCCTACTGGCATCATCAATGATAATTACACTTATAGCCCTGGGGGGCCTACCCCC

GACCTCAGGCTTCTTGCCAAAATGATTAATCCTACAAGAAATAACCAAACAACACCTGGG

AGCTGTATCTGCACTAATAGCTATATCTGCCCTACTAAGCCTATTCTTCTACCTACGACT

ATCATATGCGGTCTCATTAACCACTGCGCCAAACACCTCAAACTCCAACATGACCTGACG

AATAACCCACAACCATATACAAATCCACCCCACAATAATTACACTAACTACAATAATACT

ACCAATCACACCAACACTACTAACAATAGGACAAGGCAGCTTGGCGTACCCAGGTCTGAG

GACCCACGGGACTTTGGAGAGCATAGGTGGGCCTAAAGGCAGTTCAAGAGGAGGGTTGCC

CTCACTAGCAGACACTTTTGAACAAGTCATTGAGGAGATGCTTGAAGATGAGCAGAGCAT

GAGGCCCAGTGAGGAGAACAAGGATGCGGACATGTTTACATCTCGCGTAATGCTGAGCAG

TCAAGTGCCTTTGGAGCCTCCTCTTCTCTTTCTGCTGGAGGAGTACAAAAACTACTTGGA

TGCTGCAAATATGTCCATGAGGGTCCGACGTCACTCTGACCCCGCCCGCCGCGGGGAGCT

GAGTGTGTGTGACAGTATTAGTGAGTGGGTGACGGCATCTGACAAAAAGACTGCCGTGGA

CATGTCGGGGCAGACGGTAACTGTTCTAGAAAAAGTTCCAGTACCCAAAGGCCAACTGAA

GCAATACTTCTACGAGACCAAATGCAACCCTATGGGTTACATGAAGGAGGGCTGCAGAGG

CATTGACAAGAGGTACTGGAACTCCCAGTGCCGAACTACTCAGTCGTATGTGCGCGCGCT

CACCATGGATAACAAGAAGAAAGTGGGCTGGCGGTTTATCAGAATAGACACTTCATGTGT

ATGTACATTGACCATTGTTTGCTTGGGTTGCAGACCGAAGGCTGTTGTTCTACAAATATG

TCTACAAGCGATATAGAGCTGGGAAGCAAAGGGGTATGATTATAGAAACCGAAGGTGACA

GGCCTTCTTCAAAAGCTGACATTGAAATGGATGGAAAGATGGTCAACTCTCATACTGAAA

ATTTCATTGATGGCTCTCTAGTTCTGGAAGTCGATGAGAAAGATCAGGATGATGAAGAAG

CCAGGCGCGATATGGCCAGAATCCTAAAGGAGCTAAAGCAGAAACATCCTGACAAGGAAA

TAGAGCAACTGATTGAGTTAGCGAACTACCAGGTCTTGAGTCAACAGCAAAAAAGTCGAG

CATTCTATCGTATTCAAGCTACTCGCTTGATGACTGGGGCCGGAAATATTCTAAAGAGAC

ATGCAGCAGACCAGGCAAGGAAAGCTGTGAGCATGCAGGAAGTTAACTCTGAAGTGATCG

AAAATGAGCCCGTCAGTAAGATCTATTTCGAGCAAGC

>X9

CAACGGCCGCGGTATCATGACCGTGCAAAGGTAGCGTAATCACTTGTCTTTTAAATAAAG

ACCTGTATGAAAGGCAAAACGAAAGTTCAACTGTCTCTTTAATCCAATCAATAAAATTTA

TCTTTCCGTGCAGAAGCGGAAATAGATATATAAGACGAGAAGACCCTATGGAGCTTTAAA

CACACTATAATATAAGTTTTAGGTTGGGGCGACCGCGGAAGAAAAAAATCCTCCGAGATA

AGAATAACACTTCGAAAATTAAAACATTTTGATCCACTAAGTGACCAACGAACCAAGTTA

CCCTAGGGATAACAGCGCAATCCTTTCTAAGAGTCCATATCGACGAACGGGTTTACGACC

TCGATGTTGGATCAGGACACCCAAATGGTGCAGCCGCTATTAAGGTTCGTTTGTTCAACG

ATTAAAGTCCTACATGAACCCATATGCACTGTCAATTCTATTGTCAAGCCTAGCAGTAGG

AACAATCACAACCCTTTCAAGCTCCCATTGATTCCTCGCATGAGTGGGCCTAGAAATTAA

TACACTAGCAATCATCCCGCTAATAACAAAAATACACCACCCACGAGCAACGGAATCAGC

TACAAAATACTTTCTAACGCAAGCCACCGCATCAGCCCTAATCTTATTTTCCACAATCAC

AAACGCATGAGCCACAGGAGAATGAACAATTATTACCATAAATGACAACACGTCAACATT

TATCTTAACAATTGCCCTAGCCATCAAACTGGGAATCGCCCCATTTCACCTCTGATTACC

AGACGTTATACAAGGATTAACCCTAACAACATGTCTAATCCTTACAACATGACAAAAATT

AGCCCCAATATCCTTAATAATTATAACCGGCCATCAATTAAACACAAATCTACTAATTAC

AATGGCCATATTATCAACAGTACTTGGGGGGTGAGGGGGCCTTAACCAAACACAAATGCG

AAAAATAATAGCATATTCATCTATTGCCCACCTCGGATGAATAACCCTAATCCTATCCTT

CTCCCCAAGCTTAACAACATTAAACCTAGTAATTTACCTAATACTAACCTCATCAATATT

CTTAATAATAATAACAATAACCTCAACAAATATAAATAAACTCTCAACATCTTGACTGAA

AACCCCCCTACTGGCATCATCAATGATAATTACACTTATAGCCCTGGGGGGCCTACCCCC

AACCTCAGGCTTCTTGCCAAAATGACTAATCCTACAAGAAATAACCAAACAACACCTGGG

AGCTGTATCTACACTAATAGCTATATCTGCCCTACTAAGCCTATTCTTCTACCTACGACT

ATCATATGCAGTCTCATTAACCACTGCACCAAACACCTCAAACTCCAACATGGCCTGACG

AATAACCCACAACCACATACAAATCCACCCCACAATAATTACACTAACTACAATAATACT

ACCAATCACACCAACACTACTAACAATAGGACAAGGCAGCTTGGCGTACCCAGGTCTGAG

GACCCACGGGACTTTGGAGAGCATAGGTGGGCCTAAAGGCAGTTCAAGAGGAGGGTTGCC

CTCACTAGCAGACACTTTTGAACAAGTCATTGAGGAGATGCTTGAAGATGAGCAGAGCAT

GAGGCCCAGTGAGGAGAACAAGGATGCGGACATGTTTACATCTCGCGTAATGCTGAGCAG

TCAAGTGCCTTTGGAGCCTCCTCTTCTCTTTCTGCTGGAGGAGTACAAAAACTACTTGGA

TGCTGCAAATATGTCCATGAGGGTCCGACGTCACTCTGACCCCGCCCGCCGCGGGGAGCT

GAGTGTGTGTGACAGTATTAGTGAGTGGGTGACGGCATCTGACAAAAAGACTGCCGTGGA

CATGTCGGGGCAGACGGTAACTGTTCTAGAAAAAGTTCCAGTACCCAAAGGCCAACTGAA

GCAATACTTCTACGAGACCAAATGCAACCCTATGGGTTACATGAAGGAGGGCTGCAGAGG

CATTGACAAGAGGTACTGGAACTCCCAGTGCCGAACTACTCAGTCGTATGTGCGCGCGCT

CACCATGGATAACAAGAAGAAAGTGGGCTGGCGGTTTATCAGAATAGACACTTCATGTGT

ATGTACATTGACCATTGTTTGCTTGGGTTGCAGACCGAAGGCTGTTGTTCTACAAATATG

TCTACAAGCGATATAGAGCTGGGAAGCAAAGGGGTATGATTATAGAAACCGAAGGTGACA

GGCCTTCTTCAAAAGCTGACATTGAAATGGATGGAAAGATGGTCAACTCTCATACTGAAA

ATTTCATTGATGGCTCTCTAGTTCTGGAAGTCGATGAGAAAGATCAGGATGATGAAGAAG

CCAGGCGCGATATGGCCAGAATCCTAAAGGAGCTAAAGCAGAAACATCCTGACAAGGAAA

TAGAGCAACTGATTGAGTTAGCGAACTACCAGGTCTTGAGTCAACAGCAAAAAAGTCGAG

CATTCTATCGTATTCAAGCTACTCGCTTGATGACTGGGGCCGGAAATATTCTAAAGAGAC

ATGCAGCAGACCAGGCAAGGAAAGCTGTGAGCATGCAGGAAGTTAACTCTGAAGTGATCG

AAAATGAACCCGTCAGTAAGATCTATTTCGAGCAAGC

>X10

CAACGGCCGCGGTATCATGACCGTGCAAAGGTAGCGTAATCACTTGTCTTTTAAATAAAG

ACCTGTATGAAAGGCAAAACGAAAGTTCAACTGTCTCTTTAATCCAATCAATAAAATTTA

TCTTTCCGTGCAGAAGCGGAAATAAATATATAAGACGAGAAGACCCTATGGAGCTTTAAA

CACACCATAATATAAGTTTTAGGTTGGGGCGACCGCGGAAGAAAAAAATCCTCCGAGATA

AGAATAACACTTCGAAAATTAAAACATTTTGATCCACTAAGTGACCAACGAACCAAGTTA

CCCTAGGGATAACAGCGCAATCCTTTCTAAGAGTCCATATCGACGAACGGGTTTACGACC

TCGATGTTGGATCAGGACACCCAAATGGTGCAGCCGCTATTAAGGTTCGTTTGTTCAACG

ATTAAAGTCCTACATGAACCCATATGCACTGTCAATTCTATTGTCGAGCCTAGCAGTAGG

AACAATCACAACCCTTTCAAGCTCCCATTGATTCCTCGCATGAGTGGGCCTAGAAATTAA

TACACTAGCAATCATCCCGCTAATAACAAAAATACACCACCCACGAGCAACGGAATCAGC

CACAAAATACTTTCTAACGCAAGCCACCGCATCAGCCCTAATCTTATTTTCCACAATCAC

AAACGCATGGGCCACAGGAGAATGAACAATTATTACCATAAATGACAACACGTCAACATT

TATCTTAACAATTGCCCTGGCCATCAAACTGGGAATCGCCCCATTTCACCTCTGATTACC

AGACGTTATACAAGGATTAACCCTAACAACATGTCTAATCCTTACAACATGACAAAAATT

AGCCCCAATATCCTTAATAATTATAACCGGCCATCAATTAAACACAAACCTACTAATTAC

AATGGCCATATTATCAACAGTACTTGGGGGGTGGGGGGGCCTTAACCAAACACAAATGCG

AAAAATAATAGCATATTCATCTATTGCCCACCTCGGATGAATAACCCTAATCCTATCCTT

CTCCCCAAGCTTAACAACATTAAACCTAGTAATCTACCTAATACTAACCTCATCAATATT

CTTAATAATAATAACAATAACCTCAACAAATATAAATAAACTCTCAACATCTTGACTGAA

GACCCCCCTACTGGCATCATCAATGATAATTACACTTATAGCCCTGGGGGGCCTACCCCC

GACCTCAGGCTTCTTGCCAAAATGATTAATCCTACAAGAAATAACCAAACAACACCTGGG

AGCTGTATCTGCACTAATAGCTATATCTGCCCTACTAAGCCTATTCTTCTACCTACGACT

ATCATATGCGGTCTCATTAACCACTGCGCCAAACACCTCAAACTCCAACATGACCTGACG

AATAACCCACAACCATATACAAATCCACCCCACAATAATTACACTAACTACAATAATACT

ACCAATCACACCAACACTACTAACAATAGGACAAGGCAGCTTGGCGTACCCAGGTCTGAG

GACCCACGGGACTTTGGAGAGCATAGGTGGGCCTAAAGGCAGTTCAAGAGGAGGGTTGCC

CTCACTAGCAGACACTTTTGAACAAGTCATTGAGGAGATGCTTGAAGATGAGCAGAGCAT

GAGGCCCAGTGAGGAGAACAAGGATGCGGACATGTTTACATCTCGCGTAATGCTGAGCAG

TCAAGTGCCTTTGGAGCCTCCTCTTCTCTTTCTGCTGGAGGAGTACAAAAACTACTTGGA

TGCTGCAAATATGTCCATGAGGGTCCGACGTCACTCTGACCCCGCCCGCCGCGGGGAGCT

GAGTGTGTGTGACAGTATTAGTGAGTGGGTGACGGCATCTGACAAAAAGACTGCCGTGGA

CATGTCGGGGCAGACGGTAACTGTTCTAGAAAAAGTTCCAGTACCCAAAGGCCAACTGAA

GCAATACTTCTACGAGACCAAATGCAACCCTATGGGTTACATGAAGGAGGGCTGCAGAGG

CATTGACAAGAGGTACTGGAACTCCCAGTGCCGAACTACTCAGTCGTATGTGCGCGCGCT

CACCATGGATAACAAGAAGAAAGTGGGCTGGCGGTTTATCAGAATAGACACTTCATGTGT

ATGTACATTGACCATTGTTTGCTTGGGTTGCAGACCGAAGGCTGTTGTTCTACAAATATG

TCTACAAGCGATATAGAGCTGGGAAGCAAAGGGGTATGATTATAGAAACCGAAGGTGACA

GGCCTTCTTCAAAAGCTGACATTGAAATGGATGGAAAGATGGTCAACTCTCATACTGAAA

ATTTCATTGATGGCTCTCTAGTTCTGGAAGTCGATGAGAAAGATCAGGATGATGAAGAAG

CCAGGCGCGATATGGCCAGAATCCTAAAGGAGCTAAAGCAGAAACATCCTGACAAGGAAA

TAGAGCAACTGATTGAGTTAGCGAACTACCAGGTCTTGAGTCAACAGCAAAAAAGTCGAG

CATTCTATCGTATTCAAGCTACTCGCTTGATGACTGGGGCCGGAAATATTCTAAAGAGAC

ATGCAGCAGACCAGGCAAGGAAAGCTGTGAGCATGCAGGAAGTTAACTCTGAAGTGATCG

AAAATGAACCCGTCAGTAAGATCTATTTCGAGCAAGC

>X27

CAACGGCCGCGGTATCATGACCGTGCAAAGGTAGCGTAATCACTTGTCTTTTAAATAAAG

ACCTGTATGAAAGGCAAAACGAAAGTTCAACTGTCTCTTTAATCCAATCAATAAAATTTA

TCTTTCCGTGCAGAAGCGGAAATAAATATATAAGACGAGAAGACCCTATGGAGCTTTAAA

CACACCATAATATAAGTTTTAGGTTGGGGCGACCGCGGAAGAAAAAAATCCTCCGAGATA

AGAATAACACTTCGAAAATTAAAACATTTTGATCCACTAAGTGACCAACGAACCAAGTTA

CCCTAGGGATAACAGCGCAATCCTTTCTAAGAGTCCATATCGACGAACGGGTTTACGACC

TCGATGTTGGATCAGGACACCCAAATGGTGCAGCCGCTATTAAGGTTCGTTTGTTCAACG

ATTAAAGTCCTACATGAACCCATATGCACTGTCAATTCTATTGTCGAGCCTAGCAGTAGG

AACAATCACAACCCTTTCAAGCTCCCATTGATTCCTCGCATGAGTGGGCCTAGAAATTAA

TACACTAGCAATCATCCCGCTAATAACAAAAATACACCACCCACGAGCAACGGAATCAGC

CACAAAATACTTTCTAACGCAAGCCACCGCATCAGCCCTAATCTTATTTTCCACAATCAC

AAACGCATGGGCCACAGGAGAATGAACAATTATTACCATAAATGACAACACGTCAACATT

TATCTTAACAATTGCCCTGGCCATCAAACTGGGAATCGCCCCATTTCACCTCTGATTACC

AGACGTTATACAAGGATTAACCCTAACAACATGTCTAATCCTTACAACATGACAAAAATT

AGCCCCAATATCCTTAATAATTATAACCGGCCATCAATTAAACACAAACCTACTAATTAC

AATGGCCATATTATCAACAGTACTTGGGGGGTGGGGGGGCCTTAACCAAACACAAATGCG

AAAAATAATAGCATATTCATCTATTGCCCACCTCGGATGAATAACCCTAATCCTATCCTT

CTCCCCAAGCTTAACAACATTAAACCTAGTAATCTACCTAATACTAACCTCATCAATATT

CTTAATAATAATAACAATAACCTCAACAAATATAAATAAACTCTCAACATCTTGACTGAA

GACCCCCCTACTGGCATCATCAATGATAATTACACTTATAGCCCTGGGGGGCCTACCCCC

GACCTCAGGCTTCTTGCCAAAATGATTAATCCTACAAGAAATAACCAAACAACACCTGGG

AGCTGTATCTGCACTAATAGCTATATCTGCCCTACTAAGCCTATTCTTCTACCTACGACT

ATCATATGCGGTCTCATTAACCACTGCGCCAAACACCTCAAACTCCAACATGACCTGACG

AATAACCCACAACCATATACAAATCCACCCCACAATAATTACACTAACTACAATAATACT

ACCAATCACACCAACACTACTAACAATAGGACAAGGCAGCTTGGCGTACCCAGGTCTGAG

GACCCACGGGACTTTGGAGAGCATAGGTGGGCCTAAAGGCAGTTCAAGAGGAGGGTTGCC

CTCACTAGCAGACACTTTTGAACAAGTCATTGAGGAGATGCTTGAAGATGAGCAGAGCAT

GAGGCCCAGTGAGGAGAACAAGGATGCCGACATGTTTACATCTCGCGTAATGCTGAGCAG

TCAAGTGCCTTTGGAGCCTCCTCTTCTCTTTCTGCTGGAGGAGTACAAAAACTACTTGGA

TGCTGCAAATATGTCCATGAGGGTCCGACGTCACTCTGACCCCGCCCGCCGCGGGGAGCT

GAGTGTGTGTGACAGTATTAGTGAGTGGGTGACGGCATCTGACAAAAAGACTGCCGTGGA

CATGTCGGGGCAGACGGTAACTGTTCTAGAAAAAGTTCCAGTACCCAAAGGCCAACTGAA

GCAATACTTCTACGAGACCAAATGCAACCCTATGGGTTACATGAAGGAGGGCTGCAGAGG

CATTGACAAGAGGTACTGGAACTCCCAGTGCCGAACTACTCAGTCGTATGTGCGCGCGCT

CACCATGGATAACAAGAAGAAAGTGGGCTGGCGGTTTATCAGAATAGACACTTCATGTGT

ATGTACATTGACCATTGTTTGCTTGGGTTGCAGACCGAAGGCTGTTGTTCTACAAATATG

TCTACAAGCGATATAGAGCTGGGAAGCAAAGGGGTATGATTATAGAAACCGAAGGTGACA

GGCCTTCTTCAAAAGCTGACATTGAAATGGATGGAAAGATGGTCAACTCTCATACTGAAA

ATTTCATTGATGGCTCTCTAGTTCTGGAAGTCGATGAGAAAGATCAGGATGATGAAGAAG

CCAGGCGCGATATGGCCAGAATCCTAAAGGAGCTAAAGCAGAAACATCCTGACAAGGAAA

TAGAGCAACTGATTGAGTTAGCGAACTACCAGGTCTTGAGTCAACAGCAAAAAAGTCGAG

CATTCTATCGTATTCAAGCTACTCGCTTGATGACTGGGGCCGGAAATATTCTAAAGAGAC

ATGCAGCAGACCAGGCAAGGAAAGCTGTGAGCATGCAGGAAGTTAACTCTGAAGTGATCG

AAAATGAACCCGTCAGTAAGATCTATTTCGAGCAAGC

>X28

CAACGGCCGCGGTATCATGACCGTGCAAAGGTAGCGTAATCACTTGTCTTTTAAATAAAG

ACCTGTATGAAAGGCAAAACGAAAGTTCAACTGTCTCTTTAATCCAATCAATAAAATTTA

TCTTTCCGTGCAGAAGCGGAAATAGATATATAAGACGAGAAGACCCTATGGAGCTTTAAA

CACACTATAATATAAGTTTTAGGTTGGGGCGACCGCGGAAGAAAAAAATCCTCCGAGATA

AGAATAACACTTCGAAAATTAAAACATTTTGATCCACTAAGTGACCAACGAACCAAGTTA

CCCTAGGGATAACAGCGCAATCCTTTCTAAGAGTCCATATCGACGAACGGGTTTACGACC

TCGATGTTGGATCAGGACACCCAAATGGTGCAGCCGCTATTAAGGTTCGTTTGTTCAACG

ATTAAAGTCCTACATGAACCCATATGCACTGTCAATTCTATTGTCAAGCCTAGCAGTAGG

AACAATCACAACCCTTTCAAGCTCCCATTGATTCCTCGCATGAGTGGGCCTAGAAATTAA

TACACTAGCAATCATCCCGCTAATAACAAAAATACACCACCCACGAGCAACGGAATCAGC

TACAAAATACTTTCTAACGCAAGCCACCGCATCAGCCCTAATCTTATTTTCCACAATCAC

AAACGCATGAGCCACAGGAGAATGAACAATTATTACCATAAATGACAACACGTCAACATT

TATCTTAACAATTGCCCTAGCCATCAAACTGGGAATCGCCCCATTTCACCTCTGATTACC

AGACGTTATACAAGGATTAACCCTAACAACATGTCTAATCCTTACAACATGACAAAAATT

AGCCCCAATATCCTTAATAATTATAACCGGCCATCAATTAAACACAAATCTACTAATTAC

AATGGCCATATTATCAACAGTACTTGGGGGGTGAGGGGGCCTTAACCAAACACAAATGCG

AAAAATAATAGCATATTCATCTATTGCCCACCTCGGATGAATAACCCTAATCCTATCCTT

CTCCCCAAGCTTAACAACATTAAACCTAGTAATTTACCTAATACTAACCTCATCAATATT

CTTAATAATAATAACAATAACCTCAACAAATATAAATAAACTCTCAACATCTTGACTGAA

AACCCCCCTACTGGCATCATCAATGATAATTACACTTATAGCCCTGGGGGGCCTACCCCC

AACCTCAGGCTTCTTGCCAAAATGACTAATCCTACAAGAAATAACCAAACAACACCTGGG

AGCTGTATCTACACTAATAGCTATATCTGCCCTACTAAGCCTATTCTTCTACCTACGACT

ATCATATGCAGTCTCATTAACCACTGCACCAAACACCTCAAACTCCAACATGGCCTGACG

AATAACCCACAACCACATACAAATCCACCCCACAATAATTACACTAACTACAATAATACT

ACCAATCACACCAACACTACTAACAATAGGACAAGGCAGCTTGGCGTACCCAGGTCTGAG

GACCCACGGGACTTTGGAGAGCATAGGTGGGCCTAAAGGCAGTTCAAGAGGAGGGTTGCC

CTCACTAGCAGACACTTTTGAACAAGTCATTGAGGAGATGCTTGAAGATGAGCAGAGCAT

GAGGCCCAGTGAGGAGAACAAGGATGCGGACATGTTTACATCTCGCGTAATGCTGAGCAG

TCAAGTGCCTTTGGAGCCTCCTCTTCTCTTTCTGCTGGAGGAGTACAAAAACTACTTGGA

TGCTGCAAATATGTCCATGAGGGTCCGACGTCACTCTGACCCCGCCCGCCGCGGGGAGCT

GAGTGTGTGTGACAGTATTAGTGAGTGGGTGACGGCATCTGACAAAAAGACTGCCGTGGA

CATGTCGGGGCAGACGGTAACTGTTCTAGAAAAAGTTCCAGTACCCAAAGGCCAACTGAA

GCAATACTTCTACGAGACCAAATGCAACCCTATGGGTTACATGAAGGAGGGCTGCAGAGG

CATTGACAAGAGGTACTGGAACTCCCAGTGCCGAACTACTCAGTCGTATGTGCGCGCGCT

CACCATGGATAACAAGAAGAAAGTGGGCTGGCGGTTTATCAGAATAGACACTTCATGTGT

ATGTACATTGACCATTGTTTGCTTGGGTTGCAGACCGAAGGCTGTTGTTCTACAAATATG

TCTACAAGCGATATAGAGCTGGGAAGCAAAGGGGTATGATTATAGAAACCGAAGGTGACA

GGCCTTCTTCAAAAGCTGACATTGAAATGGATGGAAAGATGGTCAACTCTCATACTGAAA

ATTTCATTGATGGCTCTCTAGTTCTGGAAGTCGATGAGAAAGATCAGGATGATGAAGAAG

CCAGGCGCGATATGGCCAGAATCCTAAAGGAGCTAAAGCAGAAACATCCTGACAAGGAAA

TAGAGCAACTGATTGAGTTAGCGAACTACCAGGTCTTGAGTCAACAGCAAAAAAGTCGAG

CATTCTATCGTATTCAAGCTACTCGCTTGATGACTGGGGCCGGAAATATTCTAAAGAGAC

ATGCAGCAGACCAGGCAAGGAAAGCTGTGAGCATGCAGGAAGTTAACTCTGAAGTGATCG

AAAATGAGCCCGTCAGTAAGATCTATTTCGAGCAAGC

>X32

CAACGGCCGCGGTATCATGACCGTGCAAAGGTAGCGTAATCACTTGTCTTTTAAATAAAG

ACCTGTATGAAAGGCAAAACGAAAGTTCAACTGTCTCTTTAATCCAATCAATGAAATTTA

TCTTTTCGTGCAGAAGCGGAAATAAATATATAAGACGAGAAGACCCTATGGAGCTTTAAA

CACACTATAATATAAGTTTTGGGTTGGGGCGACCACGGAGAAAAGAAATCCTCCGAGATA

AGAATAACAATTCGAAAATTAAAACATTTTGATCCACTAAGTGACCAACGAACCAAGTTA

CCCTAGGGATAACAGCGCAATCCTTTCTAAGAGTCCATATCGACGAATGGGTTTACGACC

TCGATGTTGGATCAGGACACCCAAATGGTGCAGCCGCTATTAAGGTTCGTTTGTTCAACG

ATTAAAGTCCTACATGAACCCACATGCACTATCAATCCTCTTGTCGAGCCTAGCAGTAGG

AACAATTACAACTCTCTCAAGCTCCCACTGATTTCTAGCATGAGTAGGACTAGAAATTAA

CACACTAGCAATAATCCCACTAATAACAAAAATACACCACCCACGATCAACAGAATCAGC

CACAAAATATTTCCTAACGCAAGCCACCGCATCAGCCTTAATCTTATTTTCTACAATCAC

AAACGCATGAGCTACAGGAGAATGAACAATCACCACCATAAATGACAGCACATCAACACT

TATATTAACAATTGCCCTAACTATTAAGCTTGGAATCGCCCCTTTTCATCTATGACTACC

AGACGTCATACAAGGATTAAACCTAACAACATGCCTAATCCTTGCAACATGACAAAAATT

AGCCCCAATGTCCTTAATAATTATAACCAACCACCAATTAAACACAAACCTGCTAATTAC

AATAGCCGTATTATCAACAGTAATTGGGGGGTGGGGGGGCCTTAACCAAACACAAATCCG

AAAAATAATAGCATATTCATCTATCGCCCACCTAGGATGAATAACACTAATCCTATCTTT

CTCCCCAAGTTTAACAACCCTTAACCTAATAATCTACCTAATATTAACATCATCAATATT

CTTAATAATAACAACCCTAAACTCAACAAGTATTAACAAACTTTCAACATCTTGACTAAA

GACCCCCCTACTAGCATCATCAATAATAATTACACTCATAGCCCTAGGGGGCCTGCCCCC

AACATCAGGGTTTCTACCAAAATGACTCATCCTACAAGAAATAACCAAACAACACCTAGG

AGCTGTATCCACACTAATAGCTATATCTGCACTACTAAGCCTATTCTTCTACCTACGACT

ATCATATGCAATCTCACTAACTACTACACCAAACACCTCAAACTCCAACATAACCTGACG

AATAACCCACAACCAAATACAAATATTACCAACAATAATTACACTAACTACAATAATATT

ACCAATCACACCAACACTACTAGCAATAGGACAAGGCAGCTTGGCGTACCCAGGTCTGAG

GACCCACGGGACTTTGGAGAGCATAGGTGGGCCTAAAGGCAGTTCAAGAGGAGGGTTGCC

CTCACTAGCAGACACTTTTGAACAAGTCATTGAGGAGATGCTTGAAGATGAGCAGAGCAT

GAGGCCCAGTGAGGAGAACAAGGATGCGGACATGTTTACATCTCGCGTAATGCTGAGCAG

TCAAGTGCCTTTGGAGCCTCCTCTTCTCTTTCTGCTGGAGGAGTACAAAAACTACTTGGA

TGCTGCAAATATGTCCATGAGGGTCCGACGTCACTCTGACCCCGCCCGCCGCGGGGAGTT

GAGTGTGTGTGACAGTATTAGTGAGTGGGTGACGGCATCTGACAAAAAGACTGCCGTGGA

CATGTCGGGGCAGACGGTAACTGTTCTAGAAAAAGTTCCGGTACCCAAAGGCCAACTGAA

GCAATACTTCTACGAGACCAAATGCAACCCTATGGGTTACATGAAGGAGGGCTGCAGAGG

CATTGACAAGAGGTACTGGAACTCCCAGTGCCGAACTACTCAGTCGTATGTGCGCGCGCT

CACCATGGATAACAAGAAGAAAGTGGGCTGGCGGTTTATCAGAATAGACACTTCATGTGT

ATGTACATTGACCATTGTTTGCTTGGGTTGCAGACCGAAGGCTGTTGTTCTACAAATATG

TCTACAAGCGATATAGAGCTGGGAAGCAAAGGGGTATGATTATAGAAACCGAAGGAGACA

GGCCTTCTTCAAAAGCTGACATTGAAATGGATGGAAAGATGGTCAACTCTCATACTGAAA

ATTTCATTGATGGCTCTCTAGTTCTGGAAGTCGATGAGAAAGATCAGGATGATGAAGAAG

CCAGGCGCGATATGGCCAGAATCCTAAAGGAGCTAAAGCAGAAACATCCTGACAAGGAAA

TAGAGCAACTGATTGAGTTAGCGAACTACCAGGTCTTGAGTCAACAGCAAAAAAGTCGAG

CATTCTATCGTATTCAAGCTACTCGCTTGATGACTGGGGCCGGAAATATTCTAAAGAGAC

ATGCAGCAGACCAGGCAAGGAAAGCTGTGAGCATGCAGGAAGTTAACTCTGAAGTGATCG

AAAATGAGCCCGTCAGTAAGATCTATTTCGAGCAAGC

>X36

CAACGGCCGCGGTATCATGACCGTGCAAAGGTAGCGTAATCACTTGTCTTTTAAATAAAG

ACCTGTATGAAAGGCAAAACGAAAGTTCAACTGTCTCTTTAATCCAATCAATGAAATTTA

TCTTTTCGTGCAGAAGCGGAAATAAATATATAAGACGAGAAGACCCTATGGAGCTTTAAA

CACACTATAATATAAGTTTTGGGTTGGGGCGACCACGGAGAAAAGAAATCCTCCGAGATA

AGAATAACAATTCGAAAATTAAAACATTTTGATCCACTAAGTGACCAACGAACCAAGTTA

CCCTAGGGATAACAGCGCAATCCTTTCTAAGAGTCCATATCGACGAATGGGTTTACGACC

TCGATGTTGGATCAGGACACCCAAATGGTGCAGCCGCTATTAAGGTTCGTTTGTTCAACG

ATTAAAGTCCTACATGAACCCACATGCACTATCAATCCTCTTGTCGAGCCTAGCAGTAGG

AACAATTACAACTCTCTCAAGCTCCCACTGATTTCTAGCATGAGTAGGACTAGAAATTAA

CACACTAGCAATAATCCCACTAATAACAAAAATACACCACCCACGATCAACAGAATCAGC

CACAAAATATTTCCTAACGCAAGCCACCGCATCAGCCTTAATCTTATTTTCTACAATCAC

AAACGCATGAGCTACAGGAGAATGAACAATCACCACCATAAATGACAGCACATCAACACT

TATATTAACAATTGCCCTAACTATTAAGCTTGGAATCGCCCCTTTTCATCTATGACTACC

AGACGTCATACAAGGATTAAACCTAACAACATGCCTAATCCTTGCAACATGACAAAAATT

AGCCCCAATGTCCTTAATAATTATAACCAACCACCAATTAAACACAAACCTGCTAATTAC

AATAGCCGTATTATCAACAGTAATTGGGGGGTGGGGGGGCCTTAACCAAACACAAATCCG

AAAAATAATAGCATATTCATCTATCGCCCACCTAGGATGAATAACACTAATCCTATCTTT

CTCCCCAAGTTTAACAACCCTTAACCTAATAATCTACCTAATATTAACATCATCAATATT

CTTAATAATAACAACCCTAAACTCAACAAGTATTAACAAACTTTCAACATCTTGACTAAA

GACCCCCCTACTAGCATCATCAATAATAATTACACTCATAGCCCTAGGGGGCCTGCCCCC

AACATCAGGGTTTCTACCAAAATGACTCATCCTACAAGAAATAACCAAACAACACCTAGG

AGCTGTATCCACACTAATAGCTATATCTGCACTACTAAGCCTATTCTTCTACCTACGACT

ATCATATGCAATCTCACTAACTACTACACCAAACACCTCAAACTCCAACATAACCTGACG

AATAACCCACAACCAAATACAAATACTACCAACAATAATTACACTAACTACAATAATATT

ACCAATCACACCAACACTACTAGCAATAGGACAAGGCAGCTTGGCGTACCCAGGTCTGAG

GACCCACGGGACTTTGGAGACCATAGGTGGGCCTAAAGGCAGTTCAAGAGGAGGGTTGCC

CTCACTAGCAGACACTTTTGAACAAGTCATTGAGGAGATGCTTGAAGATGAGCAGAGCAT

GAGGCCCAGTGAGGAGAACAAGGATGCGGACATGTTTACATCTCGCGTAATGCTGAGCAG

TCAAGTGCCTTTGGAGCCTCCTCTTCTCTTTCTGCTGGAGGAGTACAAAAACTACTTGGA

TGCTGCAAATATGTCCATGAGGGTCCGACGTCACTCTGACCCCGCCCGCCGCGGGGAGCT

GAGTGTGTGTGACAGTATTAGTGAGTGGGTGACGGCATCTGACAAAAAGACTGCCGTGGA

CATGTCGGGGCAGACGGTAACTGTTCTAGAAAAAGTTCCAGTACCCAAAGGCCAACTGAA

GCAATACTTCTACGAGACCAAATGCAACCCTATGGGTTACATGAAGGAGGGCTGCAGAGG

CATTGACAAGAGGTACTGGAACTCCCAGTGCCGAACTACTCAGTCGTATGTGCGCGCGCT

CACCATGGATAACAAGAAGAAAGTGGGCTGGCGGTTTATCAGAATAGACACTTCATGTGT

ATGTACATTGACCATTGTTTGCTTGGGTTGCAGACCGAAGGCTGTTGTTCTACAAATATG

TCTACAAGCGATATAGAGCTGGGAAGCAAAGGGGTATGATTATAGAAACCGAAGGTGACA

GGCCTTCTTCAAAAGCTGACATTGAAATGGATGGAAAGATGGTCAACTCTCATACTGAAA

ATTTCATTGATGGCTCTCTAGTTCTGGAAGTCGATGAGAAAGATCAGGATGATGAAGAAG

CCAGGCGCGATATGGCCAGAATCCTAAAGGAGCTAAAGCAGAAACATCCTGACAAGGAAA

TAGAGCAACTGATTGAGTTAGCGAACTACCAGGTCTTGAGTCAACAGCAAAAAAGTCGAG

CATTCTATCGTATTCAAGCTACTCGCTTGATGACTGGGGCCGGAAATATTCTAAAGAGAC

ATGCAGCAGACCAGGCAAGGAAAGCTGTGAGCATGCAGGAAGTTAACTCTGAAGTGATCG

AAAATGAGCCCGTCAGTAAGATCTATTTCGAGCAAGC

>X37

CAACGGCCGCGGTATCATGACCGTGCAAAGGTAGCGTAATCACTTGTCTTTTAAATAAAG

ACCTGTATGAAAGGCAAAACGAAAGTTCAACTGTCTCTTTAATCCAATCAATAAAATTTA

TCTTTCCGTGCAGAAGCGGAAATAAATATATAAGACGAGAAGACCCTATGGAGCTTTAAA

CACACTATAATATAAGTTTTGGGTTGGGGCGACCGCGGAAGAAAAAAATCCTCCGAGATA

AGAATAACACTTCGAAAATTAAAACATTTTGATCCACTTAGTGACCAACGAACCAAGTTA

CCCTAGGGATAACAGCGCAATCCTTTCTAAGAGTCCATATCGACGAATGGGTTTACGACC

TCGATGTTGGATCAGGACACCCAAATGGTGCAGCCGCTATTAAGGTTCGTTTGTTCAACG

ATTAAAGTCCTACATGAACCCATATGCACTGTCAATTCTATTGTCGAGCCTAGCAGTAGG

AACAATCACAACCCTTTCGAGCTCCCATTGATTCCTCGCATGAGTGGGCCTAGAAATTAA

CACGCTAGCAATCATCCCACTAATAACAAAAATACACCACCCACGAGCAACAGAATCAGC

CACAAAATACTTCCTAACGCAAGCCACCGCATCAGCCTTAATCTTATTTTCTACAATCAC

AAACGCATGAGCCACAGGAGAATGAACAATCATCACCATAAATGACAACACATCAACATT

CATCTTAACAATTGCCCTAGCCATTAAACTGGGAATCGCCCCATTTCACCTCTGACTACC

AGACGTCATACAAGGATTAACCCTAACAACATGTCTAATCCTTACAACATGACAAAAATT

AGCCCCAATATCCTTAATAATTATAATCAGCCATCAATTAAACACAAACCTACTAATTAC

AATAGCCGTATTATCAACAGTACTAGGGGGGTGGGGGGGCCTTAACCAAACACAAATACG

AAAAATAATAGCATACTCATCTATTGCCCACCTCGGATGAATAACGCTAATCCTATCCTT

CTCCCCAAACTTAACAACATTAAACCTAATAATTTACCTAATACTAACCTCATCAATATT

CTTAATAATAATAACAATAACCTCAACAAATATAAATAAACTTTCAACATCTTGACTAAA

AACTCCCCTACTGGCATCATCAATAATAATTACACTTATAGCCCTAGGAGGCCTACCCCC

AACCTCAGGCTTCTTGCCAAAATGACTAATCCTACAAGAAATAATCAAACAACACCTGGG

AGCTGTATCCACACTAATGGCTATATCTGCACTACTAAGCCTATTCTTTTACCTACGACT

ATCATATGCAATCTCATTAACTACTGCACCAAACACCTCAAACTCCAACATGGCCTGACG

AATAACCCACAACCAAATACAAATCCACCCCCAAATAATTACACTAACTACAATAATACT

ACCAATCACACCAACACTACTAGCAATAGGACAAGGCAGCTTGGCGTACCCAGGTCTGAG

GACCCACGGGACTTTGGAGAGCATAGGTGGGCCTAAAGGCAGTTCAAGAGGAGGGTTGCC

CTCACTAGCAGACACTTTTGAACAAGTCATTGAGGAGATGCTTGAAGATGAGCAGAGTAT

GAGGCCCAGTGAGGAGAACAAGGATGCGGACATGTTTACATCTCGCGTAATGCTGAGCAG

TCAAGTGCCTTTGGAGCCTCCTCTTCTCTTTCTGCTGGAGGAGTACAAAAACTACTTGGA

TGCTGCAAATATGTCCATGAGGGTCCGACGTCACTCTGACCCCGCCCGCCGCGGGGAGCT

GAGTGTGTGTGACAGTATTAGTGAGTGGGTGACGGCATCTGACAAAAAGACTGCCGTGGA

CATGTCGGGGCAGACGGTAACTGTTCTAGAAAAAGTTCCAGTACCCAAAGGCCAACTGAA

GCAATACTTCTACGAGACCAAATGCAACCCTATGGGTTACATGAAGGAGGGCTGCAGAGG

CATTGACAAGAGGTACTGGAACTCCCAGTGCCGAACTACTCAGTCGTATGTGCGCGCGCT

CACCATGGATAACAAGAAGAAAGTGGGCTGGCGGTTTATCAGAATAGACACTTCATGTGT

ATGTACATTGACCATTGTTTGCTTGGGTTGCAGACCGAAGGCTGTTGTTCTACAAATATG

TCTACAAGCGATATAGAGCTGGGAAGCAAAGGGGTATGATTATAGAAACCGAAGGTGACA

GGCCTTCTTCAAAAGCTGACATTGAAATGGATGGAAAGATGGTCAACTCTCATACTGAAA

ATTTCATTGATGGCTCTCTAGTTCTGGAAGTCGATGAGAAAGATCAGGATGATGAAGAAG

CCAGGCGCGATATGGCCAGAATCCTAAAGGAGCTAAAGCAGAAACATCCTGACAAGGAAA

TAGAGCAACTGATTGAGTTAGCGAACTACCAGGTCTTGAGTCAACAGCAAAAAAGTCGAG

CATTCTATCGTATTCAAGCTACTCGCTTGATGACTGGGGCCGGAAATATTCTAAAGAGAC

ATGCAGCAGACCAGGCAAGGAAAGCTGTGAGCATGCAGGAAGTTAACTCTGAAGTGATCG

AAAATGAGCCCGTCAGTAAGATCTATTTCGAGCAAGC

>X38

CAACGGCCGCGGTATCATGACCGTGCAAAGGTAGCGTAATCACTTGTCTTTTAAATAAAG

ACCTGTATGAAAGGCAAAACGAAAGTTCAACTGTCTCTTTAATCCAATCAATAAAATTTA

TCTTTCCGTGCAGAAGCGGAAATAAATATATAAGACGAGAAGACCCTATGGAGCTTTAAA

CACACTATAATATAAGTTTTGGGTTGGGGCGACCGCGGAAGAAAAAAATCCTCCGAGATA

AGAATAACACTTCGAAAATTAAAACATTTTGATCCACTTAGTGACCAACGAACCAAGTTA

CCCTAGGGATAACAGCGCAATCCTTTCTAAGAGTCCATATCGACGAATGGGTTTACGACC

TCGATGTTGGATCAGGACACCCAAATGGTGCAGCCGCTATTAAGGTTCGTTTGTTCAACG

ATTAAAGTCCTACATGAACCCATATGCACTGTCAATTCTATTGTCGAGCCTAGCAGTAGG

AACAATCACAACCCTTTCGAGCTCCCATTGATTCCTCGCATGAGTGGGCCTAGAAATTAA

CACGCTAGCAATCATCCCACTAATAACAAAAATACACCACCCACGAGCAACAGAATCAGC

CACAAAATACTTCCTAACGCAAGCCACCGCATCAGCCTTAATCTTATTTTCTACAATCAC

AAACGCATGAGCCACAGGAGAATGAACAATCATCACCATAAATGACAACACATCAACATT

CATCTTAACAATTGCCCTAGCCATTAAACTGGGAATCGCCCCATTTCACCTCTGACTACC

AGACGTCATACAAGGATTAACCCTAACAACATGTCTAATCCTTACAACATGACAAAAATT

AGCCCCAATATCCTTAATAATTATAATCAGCCATCAATTAAACACAAACCTACTAATTAC

AATAGCCGTATTATCAACAGTACTAGGGGGGTGGGGGGGCCTTAACCAAACACAAATACG

AAAAATAATAGCATACTCATCTATTGCCCACCTCGGATGAATAACGCTAATCCTATCCTT

CTCCCCAAACTTAACAACATTAAACCTAATAATTTACCTAATACTAACCTCATCAATATT

CTTAATAATAATAACAATAACCTCAACAAATATAAATAAACTTTCAACATCTTGACTAAA

AACTCCCCTACTGGCATCATCAATAATAATTACACTTATAGCCCTAGGAGGCCTACCCCC

AACCTCAGGCTTCTTGCCAAAATGACTAATCCTACAAGAAATAATCAAACAACACCTGGG

AGCTGTATCCACACTAATGGCTATATCTGCACTACTAAGCCTATTCTTTTACCTACGACT

ATCATATGCAATCTCATTAACTACTGCACCAAACACCTCAAACTCCAACATGGCCTGACG

AATAACCCACAACCAAATACAAATCCACCCCCAAATAATTACACTAACTACAATAATACT

ACCAATCACACCAACACTACTAGCAATAGGACAAGGCAGCTTGGCGTACCCAGGTCTGAG

GACCCACGGGACTTTGGAGAGCATAGGTGGGCCTAAAGGCAGTTCAAGAGGAGGGTTGCC

CTCACTAGCAGACACTTTTGAACAAGTCATTGAGGAGATGCTTGAAGATGAGCAGAGTAT

GAGGCCCAGTGAGGAGAACAAGGATGCGGACATGTTTACATCTCGCGTAATGCTGAGCAG

TCAAGTGCCTTTGGAGCCTCCTCTTCTCTTTCTGCTGGAGGAGTACAAAAACTACTTGGA

TGCTGCAAATATGTCCATGAGGGTCCGACGTCACTCTGACCCCGCCCGCCGCGGGGAGCT

GAGTGTGTGTGACAGTATTAGTGAGTGGGTGACGGCATCTGACAAAAAGACTGCCGTGGA

CATGTCGGGGCAGACGGTAACTGTTCTAGAAAAAGTTCCAGTACCCAAAGGCCAACTGAA

GCAATACTTCTACGAGACCAAATGCAACCCTATGGGTTACATGAAGGAGGGCTGCAGAGG

CATTGACAAGAGGTACTGGAACTCCCAGTGCCGAACTACTCAGTCGTATGTGCGCGCGCT

CACCATGGATAACAAGAAGAAAGTGGGCTGGCGGTTTATCAGAATAGACACTTCATGTGT

ATGTACATTGACCATTGTTTGCTTGGGTTGCAGACCGAAGGCTGTTGTTCTACAAATATG

TCTACAAGCGATATAGAGCTGGGAAGCAAAGGGGTATGATTATAGAAACCGAAGGTGACA

GGCCTTCTTCAAAAGCTGACATTGAAATGGATGGAAAGATGGTCAACTCTCATACTGAAA

ATTTCATTGATGGCTCTCTAGTTCTGGAAGTCGATGAGAAAGATCAGGATGATGAAGAAG

CCAGGCGCGATATGGCCAGAATCCTAAAGGAGCTAAAGCAGAAACATCCTGACAAGGAAA

TAGAGCAACTGATTGAGTTAGCGAACTACCAGGTCTTGAGTCAACAGCAAAAAAGTCGAG

CATTCTATCGTATTCAAGCTACTCGCTTGATGACTGGGGCCGGAAATATTCTAAAGAGAC

ATGCAGCAGACCAGGCAAGGAAAGCTGTGAGCATGCAGGAAGTTAACTCTGAAGTGATCG

AAAATGAGCCCGTCAGTAAGATCTATTTCGAGCAAGC

>X40

CAACGGCCGCGGTATCATGACCGTGCAAAGGTAGCGTAATCACTTGTCTTTTAAATAAAG

ACCTGTATGAAAGGCAAAACGAAAGTTCAACTGTCTCTTTAATCCAATCAATAAAATTTA

TCTTTCCGTGCAGAAGCGGAAATAAATATATAAGACGAGAAGACCCTATGGAGCTTTAAA

CACACTATAATATAAGTTTTGGGTTGGGGCGACCGCGGAAGAAAAAAATCCTCCGAGATA

AGAATAACACTTCGAAAATTAAAACATTTTGATCCACTTAGTGACCAACGAACCAAGTTA

CCCTAGGGATAACAGCGCAATCCTTTCTAAGAGTCCATATCGACGAATGGGTTTACGACC

TCGATGTTGGATCAGGACACCCAAATGGTGCAGCCGCTATTAAGGTTCGTTTGTTCAACG

ATTAAAGTCCTACATGAACCCATATGCACTGTCAATTCTATTGTCGAGCCTAGCAGTAGG

AACAATCACAACCCTTTCGAGCTCCCATTGATTCCTCGCATGAGTGGGCCTAGAAATTAA

CACGCTAGCAATCATCCCACTAATAACAAAAATACACCACCCACGAGCAACAGAATCAGC

CACAAAATACTTCCTAACGCAAGCCACCGCATCAGCCTTAATCTTATTTTCTACAATCAC

AAACGCATGAGCCACAGGAGAATGAACAATCATCACCATAAATGACAACACATCAACATT

CATCTTAACAATTGCCCTAGCCATTAAACTGGGAATCGCCCCATTTCACCTCTGACTACC

AGACGTCATACAAGGATTAACCCTAACAACATGTCTAATCCTTACAACATGACAAAAATT

AGCCCCAATATCCTTAATAATTATAATCAGCCATCAATTAAACACAAACCTACTAATTAC

AATAGCCGTATTATCAACAGTACTAGGGGGGTGGGGGGGCCTTAACCAAACACAAATACG

AAAAATAATAGCATACTCATCTATTGCCCACCTCGGATGAATAACGCTAATCCTATCCTT

CTCCCCAAACTTAACAACATTAAACCTAATAATTTACCTAATACTAACCTCATCAATATT

CTTAATAATAATAACAATAACCTCAACAAATATAAATAAACTTTCAACATCTTGACTAAA

AACTCCCCTACTGGCATCATCAATAATAATTACACTTATAGCCCTAGGAGGCCTACCCCC

AACCTCAGGCTTCTTGCCAAAATGACTAATCCTACAAGAAATAATCAAACAACACCTGGG

AGCTGTATCCACACTAATGGCTATATCTGCACTACTAAGCCTATTCTTTTACCTACGACT

ATCATATGCAATCTCATTAACTACTGCACCAAACACCTCAAACTCCAACATGGCCTGACG

AATAACCCACAACCAAATACAAATCCACCCCCAAATAATTACACTAACTACAATAATACT

ACCAATCACACCAACACTACTAGCAATAGGACAAGGCAGCTTGGCGTACCCAGGTCTGAG

GACCCACGGGACTTTGGAGAGCATAGGTGGGCCTAAAGGCAGTTCAAGAGGAGGGTTGCC

CTCACTAGCAGACACTTTTGAACAAGTCATTGAGGAGATGCTTGAAGATGAGCAGAGTAT

GAGGCCCAGTGAGGAGAACAAGGATGCGGACATGTTTACATCTCGCGTAATGCTGAGCAG

TCAAGTGCCTTTGGAGCCTCCTCTTCTCTTTCTGCTGGAGGAGTACAAAAACTACTTGGA

TGCTGCAAATATGTCCATGAGGGTCCGACGTCACTCTGACCCCGCCCGCCGCGGGGAGCT

GAGTGTGTGTGACAGTATTAGTGAGTGGGTGACGGCATCTGACAAAAAGACTGCCGTGGA

CATGTCGGGGCAGACGGTAACTGTTCTAGAAAAAGTTCCAGTACCCAAAGGCCAACTGAA

GCAATACTTCTACGAGACCAAATGCAACCCTATGGGTTACATGAAGGAGGGCTGCAGAGG

CATTGACAAGAGGTACTGGAACTCCCAGTGCCGAACTACTCAGTCGTATGTGCGCGCGCT

CACCATGGATAACAAGAAGAAAGTGGGCTGGCGGTTTATCAGAATAGACACTTCATGTGT

ATGTACATTGACCATTGTTTGCTTGGGTTGCAGACCGAAGGCTGTTGTTCTACAAATATG

TCTACAAGCGATATAGAGCTGGGAAGCAAAGGGGTATGATTATAGAAACCGAAGGTGACA

GGCCTTCTTCAAAAGCTGACATTGAAATGGATGGAAAGATGGTCAACTCTCATACTGAAA

ATTTCATTGATGGCTCTCTAGTTCTGGAAGTCGATGAGAAAGATCAGGATGATGAAGAAG

CCAGGCGCGATATGGCCAGAATCCTAAAGGAGCTAAAGCAGAAACATCCTGACAAGGAAA

TAGAGCAACTGATTGAGTTAGCGAACTACCAGGTCTTGAGTCAACAGCAAAAAAGTCGAG

CATTCTATCGTATTCAAGCTACTCGCTTGATGACTGGGGCCGGAAATATTCTAAAGAGAC

ATGCAGCAGACCAGGCAAGGAAAGCTGTGAGCATGCAGGAAGTTAACTCTGAAGTGATCG

AAAATGAGCCCGTCAGTAAGATCTATTTCGAGCAAGC

>X41

CAACGGCCGCGGTATCATGACCGTGCAAAGGTAGCGTAATCACTTGTCTTTTAAATAAAG

ACCTGTATGAAAGGCAAAACGAAAGTTCAACTGTCTCTTTAATCCAATCAATAAAATTTA

TCTTTCCGTGCAGAAGCGGAAATAAATATATAAGACGAGAAGACCCTATGGAGCTTTAAA

CACACTATAATATAAGTTTTGGGTTGGGGCGACCGCGGAAGAAAAAAATCCTCCGAGATA

AGAATAACACTTCGAAAATTAAAACATTTTGATCCACTTAGTGACCAACGAACCAAGTTA

CCCTAGGGATAACAGCGCAATCCTTTCTAAGAGTCCATATCGACGAATGGGTTTACGACC

TCGATGTTGGATCAGGACACCCAAATGGTGCAGCCGCTATTAAGGTTCGTTTGTTCAACG

ATTAAAGTCCTACATGAACCCATATGCACTGTCAATTCTATTGTCGAGCCTAGCAGTAGG

AACAATCACAACCCTTTCGAGCTCCCATTGATTCCTCGCATGAGTGGGCCTAGAAATTAA

CACGCTAGCAATCATCCCACTAATAACAAAAATACACCACCCACGAGCAACAGAATCAGC

CACAAAATACTTCCTAACGCAAGCCACCGCATCAGCCTTAATCTTATTTTCTACAATCAC

AAACGCATGAGCCACAGGAGAATGAACAATCATCACCATAAATGACAACACATCAACATT

CATCTTAACAATTGCCCTAGCCATTAAACTGGGAATCGCCCCATTTCACCTCTGACTACC

AGACGTCATACAAGGATTAACCCTAACAACATGTCTAATCCTTACAACATGACAAAAATT

AGCCCCAATATCCTTAATAATTATAATCAGCCATCAATTAAACACAAACCTACTAATTAC

AATAGCCGTATTATCAACAGTACTAGGGGGGTGGGGGGGCCTTAACCAAACACAAATACG

AAAAATAATAGCATACTCATCTATTGCCCACCTCGGATGAATAACGCTAATCCTATCCTT

CTCCCCAAACTTAACAACATTAAACCTAATAATTTACCTAATACTAACCTCATCAATATT

CTTAATAATAATAACAATAACCTCAACAAATATAAATAAACTTTCAACATCTTGACTAAA

AACTCCCCTACTGGCATCATCAATAATAATTACACTTATAGCCCTAGGAGGCCTACCCCC

AACCTCAGGCTTCTTGCCAAAATGACTAATCCTACAAGAAATAATCAAACAACACCTGGG

AGCTGTATCCACACTAATGGCTATATCTGCACTACTAAGCCTATTCTTTTACCTACGACT

ATCATATGCAATCTCATTAACTACTGCACCAAACACCTCAAACTCCAACATGGCCTGACG

AATAACCCACAACCAAATACAAATCCACCCCCAAATAATTACACTAACTACAATAATACT

ACCAATCACACCAACACTACTAGCAATAGGACAAGGCAGCTTGGCGTACCCAGGTCTGAG

GACCCACGGGACTTTGGAGAGCATAGGTGGGCCTAAAGGCAGTTCAAGAGGAGGGTTGCC

CTCACTAGCAGACACTTTTGAACAAGTCATTGAGGAGATGCTTGAAGATGAGCAGAGTAT

GAGGCCCAGTGAGGAGAACAAGGATGCGGACATGTTTACATCTCGCGTAATGCTGAGCAG

TCAAGTGCCTTTGGAGCCTCCTCTTCTCTTTCTGCTGGAGGAGTACAAAAACTACTTGGA

TGCTGCAAATATGTCCATGAGGGTCCGACGTCACTCTGACCCCGCCCGCCGCGGGGAGCT

GAGTGTGTGTGACAGTATTAGTGAGTGGGTGACGGCATCTGACAAAAAGACTGCCGTGGA

CATGTCGGGGCAGACGGTAACTGTTCTAGAAAAAGTTCCAGTACCCAAAGGCCAACTGAA

GCAATACTTCTACGAGACCAAATGCAACCCTATGGGTTACATGAAGGAGGGCTGCAGAGG

CATTGACAAGAGGTACTGGAACTCCCAGTGCCGAACTACTCAGTCGTATGTGCGCGCGCT

CACCATGGATAACAAGAAGAAAGTGGGCTGGCGGTTTATCAGAATAGACACTTCATGTGT

ATGTACATTGACCATTGTTTGCTTGGGTTGCAGACCGAAGGCTGTTGTTCTACAAATATG

TCTACAAGCGATATAGAGCTGGGAAGCAAAGGGGTATGATTATAGAAACCGAAGGTGACA

GGCCTTCTTCAAAAGCTGACATTGAAATGGATGGAAAGATGGTCAACTCTCATACTGAAA

ATTTCATTGATGGCTCTCTAGTTCTGGAAGTCGATGAGAAAGATCAGGATGATGAAGAAG

CCAGGCGCGATATGGCCAGAATCCTAAAGGAGCTAAAGCAGAAACATCCTGACAAGGAAA

TAGAGCAACTGATTGAGTTAGCGAACTACCAGGTCTTGAGTCAACAGCAAAAAAGTCGAG

CATTCTATCGTATTCAAGCTACTCGCTTGATGACTGGGGCCGGAAATATTCTAAAGAGAC

ATGCAGCAGACCAGGCAAGGAAAGCTGTGAGCATGCAGGAAGTTAACTCTGAAGTGATCG

AAAATGAGCCCGTCAGTAAGATCTATTTCGAGCAAGC

>X42

CAACGGCCGCGGTATCATGACCGTGCAAAGGTAGCGTAATCACTTGTCTTTTAAATAAAG

ACCTGTATGAAAGGCAAAACGAAAGTTCAACTGTCTCTTTAATCCAATCAATGAAATTTA

TCTTTTCGTGCAGAAGCGGAAATAAATATATAAGACGAGAAGACCCTATGGAGCTTTAAA

CACACTATAATATAAGTTTTGGGTTGGGGCGACCACGGAGAAAAGAAATCCTCCGAGATA

AGAATAACAATTCGAAAATTAAAACATTTTGATCCACTAAGTGACCAACGAACCAAGTTA

CCCTAGGGATAACAGCGCAATCCTTTCTAAGAGTCCATATCGACGAATGGGTTTACGACC

TCGATGTTGGATCAGGACACCCAAATGGTGCAGCCGCTATTAAGGTTCGTTTGTTCAACG

ATTAAAGTCCTACATGAACCCACATGCACTATCAATCCTCTTGTCGAGCCTAGCAGTAGG

AACAATTACAACTCTCTCAAGCTCCCACTGATTTCTAGCATGAGTAGGACTAGAAATTAA

CACACTAGCAATAATCCCACTAATAACAAAAATACACCACCCACGATCAACAGAATCAGC

CACAAAATATTTCCTAACGCAAGCCACCGCATCAGCCTTAATCTTATTTTCTACAATCAC

AAACGCATGAGCTACAGGAGAATGAACAATCACCACCATAAATGACAGCACATCAACACT

TATATTAACAATTGCCCTAACTATTAAGCTTGGAATCGCCCCTTTTCATCTATGACTACC

AGACGTCATACAAGGATTAAACCTAACAACATGCCTAATCCTTGCAACATGACAAAAATT

AGCCCCAATGTCCTTAATAATTATAACCAACCACCAATTAAACACAAACCTGCTAATTAC

AATAGCCGTATTATCAACAGTAATTGGGGGGTGGGGGGGCCTTAACCAAACACAAATCCG

AAAAATAATAGCATATTCATCTATCGCCCACCTAGGATGAATAACACTAATCCTATCTTT

CTCCCCAAGTTTAACAACCCTTAACCTAATAATCTACCTAATATTAACATCATCAATATT

CTTAATAATAACAACCCTAAACTCAACAAGTATTAACAAACTTTCAACATCTTGACTAAA

AACCCCCCTACTAGCATCATCAATAATAATTACACTCATAGCCCTAGGGGGCCTGCCCCC

AACATCAGGGTTTCTACCAAAATGACTCATCCTACAAGAAATAACCAAACAACACCTAGG

AGCTGTATCCACACTAATAGCTATATCTGCACTACTAAGCCTATTCTTCTACCTACGACT

ATCATATGCAATCTCACTAACTACTACACCAAACACCTCAAACTCCAACATAACCTGACG

AATAACCCACAACCAAATACAAATCCTACCAACAATAATTACACTAACTACAATAATATT

ACCAATCACACCAACACTACTAGCAATAGGACAAGGCAGCTTGGCGTACCCAGGTCTGAG

GACCCACGGGACTTTGGAGAGCATAGGTGGGCCTAAAGGCAGTTCAAGAGGAGGGTTGCC

CTCACTAGCAGACACTTTTGAACAAGTCATTGAGGAGATGCTTGAAGATGAGCAGAGCAT

GAGGCCCAGTGAGGAGAACAAGGATGCCGACATGTTTACATCTCGCGTAATGCTGAGCAG

TCAAGTGCCTTTGGAGCCTCCTCTTCTCTTTCTGCTGGAGGAGTACAAAAACTACTTGGA

TGCTGCAAATATGTCCATGAGGGTCCGACGTCACTCTGACCCCGCCCGCCGCGGGGAGCT

GAGTGTGTGTGACAGTATTAGTGAGTGGGTGACGGCATCTGACAAAAAGACTGCCGTGGA

CATGTCGGGGCAGACGGTAACTGTTCTAGAAAAAGTTCCAGTACCCAAAGGCCAACTGAA

GCAATACTTCTACGAGACCAAATGCAACCCTATGGGTTACATGAAGGAGGGCTGCAGAGG

CATTGACAAGAGGTACTGGAACTCCCAGTGCCGAACTACTCAGTCGTATGTGCGCGCGCT

CACCATGGATAACAAGAAGAAAGTGGGCTGGCGGTTTATCAGAATAGACACTTCATGTGT

ATGTACATTGACCATTGTTTGCTTGGGTTGCAGACCGAAGGCTGTTGTTCTACAAATATG

TCTACAAGCGATATAGAGCTGGGAAGCAAAGGGGTATGATTATAGAAACCGAAGGTGACA

GGCCTTCTTCAAAAGCTGACATTGAAATGGATGGAAAGATGGTCAACTCTCATACTGAAA

ATTTCATTGATGGCTCTCTAGTTCTGGAAGTCGATGAGAAAGATCAGGATGATGAAGAAG

CCAGGCGCGATATGGCCAGAATCCTAAAGGAGCTAAAGCAGAAACATCCTGACAAGGAAA

TAGAGCAACTGATTGAGTTAGCGAACTACCAGGTCTTGAGTCAACAGCAAAAAAGTCGAG

CATTCTATCGTATTCAAGCTACTCGCTTGATGACTGGGGCCGGAAATATTCTAAAGAGAC

ATGCAGCAGACCAGGCAAGGAAAGCTGTGAGCATGCAGGAAGTTAACTCTGAAGTGATCG

AAAATGAGCCCGTCAGTAAGATCTATTTCGAGCAAGC

>X44

CAACGGCCGCGGTATCATGACCGTGCAAAGGTAGCGTAATCACTTGTCTTTTAAATAAAG

ACCTGTATGAAAGGCAAAACGAAAGTTCAACTGTCTCTTTAATCCAATCAATGAAATTTA

TCTTTTCGTGCAGAAGCGGAAATAAATATATAAGACGAGAAGACCCTATGGAGCTTTAAA

CACACTATAATATAAGTTTTGGGTTGGGGCGACCACGGAGAAAAGAAATCCTCCGAGATA

AGAATAACAATTCGAAAATTAAAACATTTTGATCCACTAAGTGACCAACGAACCAAGTTA

CCCTAGGGATAACAGCGCAATCCTTTCTAAGAGTCCATATCGACGAATGGGTTTACGACC

TCGATGTTGGATCAGGACACCCAAATGGTGCAGCCGCTATTAAGGTTCGTTTGTTCAACG

ATTAAAGTCCTACATGAACCCACATGCACTATCAATCCTCTTGTCGAGCCTAGCAGTAGG

AACAATTACAACTCTCTCAAGCTCCCACTGATTTCTAGCATGAGTAGGACTAGAAATTAA

CACACTAGCAATAATCCCACTAATAACAAAAATACACCACCCACGATCAACAGAATCAGC

CACAAAATATTTCCTAACGCAAGCCACCGCATCAGCCTTAATCTTATTTTCTACAATCAC

AAACGCATGAGCTACAGGAGAATGAACAATCACCACCATAAATGACAGCACATCAACACT

TATATTAACAATTGCCCTAACTATTAAGCTTGGAATCGCCCCTTTTCATCTATGACTACC

AGACGTCATACAAGGATTAAACCTAACAACATGCCTAATCCTTGCAACATGACAAAAATT

AGCCCCAATGTCCTTAATAATTATAACCAACCACCAATTAAACACAAACCTGCTAATTAC

AATAGCCGTATTATCAACAGTAATTGGGGGGTGGGGGGGCCTTAACCAAACACAAATCCG

AAAAATAATAGCATATTCATCTATCGCCCACCTAGGATGAATAACACTAATCCTATCTTT

CTCCCCAAGTTTAACAACCCTTAACCTAATAATCTACCTAATATTAACATCATCAATATT

CTTAATAATAACAACCCTAAACTCAACAAGTATTAACAAACTTTCAACATCTTGACTAAA

AACCCCCCTACTAGCATCATCAATAATAATTACACTCATAGCCCTAGGGGGCCTGCCCCC

AACATCAGGGTTTCTACCAAAATGACTCATCCTACAAGAAATAACCAAACAACACCTAGG

AGCTGTATCCACACTAATAGCTATATCTGCACTACTAAGCCTATTCTTCTACCTACGACT

ATCATATGCAATCTCACTAACTACTACACCAAACACCTCAAACTCCAACATAACCTGACG

AATAACCCACAACCAAATACAAATCCTACCAACAATAATTACACTAACTACAATAATATT

ACCAATCACACCAACACTACTAGCAATAGGACAAGGCAGCTTGGCGTACCCAGGTCTGAG

GACCCACGGGACTTTGGAGAGCATAGGTGGGCCTAAAGGCAGTTCAAGAGGAGGGTTGCC

CTCACTAGCAGACACTTTTGAACAAGTCATTGAGGAGATGCTTGAAGATGAGCAGAGCAT

GAGGCCCAGTGAGGAGAACAAGGATGCCGACATGTTTACATCTCGCGTAATGCTGAGCAG

TCAAGTGCCTTTGGAGCCTCCTCTTCTCTTTCTGCTGGAGGAGTACAAAAACTACTTGGA

TGCTGCAAATATGTCCATGAGGGTCCGACGTCACTCTGACCCCGCCCGCCGCGGGGAGCT

GAGTGTGTGTGACAGTATTAGTGAGTGGGTGACGGCATCTGACAAAAAGACTGCCGTGGA

CATGTCGGGGCAGACGGTAACTGTTCTAGAAAAAGTTCCAGTACCCAAAGGCCAACTGAA

GCAATACTTCTACGAGACCAAATGCAACCCTATGGGTTACATGAAGGAGGGCTGCAGAGG

CATTGACAAGAGGTACTGGAACTCCCAGTGCCGAACTACTCAGTCGTATGTGCGCGCGCT

CACCATGGATAACAAGAAGAAAGTGGGCTGGCGGTTTATCAGAATAGACACTTCATGTGT

ATGTACATTGACCATTGTTTGCTTGGGTTGCAGACCGAAGGCTGTTGTTCTACAAATATG

TCTACAAGCGATATAGAGCTGGGAAGCAAAGGGGTATGATTATAGAAACCGAAGGTGACA

GGCCTTCTTCAAAAGCTGACATTGAAATGGATGGAAAGATGGTCAACTCTCATACTGAAA

ATTTCATTGATGGCTCTCTAGTTCTGGAAGTCGATGAGAAAGATCAGGATGATGAAGAAG

CCAGGCGCGATATGGCCAGAATCCTAAAGGAGCTAAAGCAGAAACATCCTGACAAGGAAA

TAGAGCAACTGATTGAGTTAGCGAACTACCAGGTCTTGAGTCAACAGCAAAAAAGTCGAG

CATTCTATCGTATTCAAGCTACTCGCTTGATGACTGGGGCCGGAAATATTCTAAAGAGAC

ATGCAGCAGACCAGGCAAGGAAAGCTGTGAGCATGCAGGAAGTTAACTCTGAAGTGATCG

AAAATGAGCCCGTCAGTAAGATCTATTTCGAGCAAGC

>X46

CAACGGCCGCGGTATCATGACCGTGCAAAGGTAGCGTAATCACTTGTCTTTTAAATAAAG

ACCTGTATGAAAGGCAAAACGAAAGTTCAACTGTCTCTTTAATCCAATCAATGAAATTTA

TCTTTTCGTGCAGAAGCGGAAATAAATATATAAGACGAGAAGACCCTATGGAGCTTTAAA

CACACTATAATACAAGTTTTGGGTTGGGGCGACCGCGGAGAAAAAAAATCCTCCGAGATA

AGAATAACACTTCGAAAATTAAAACATTTTGATCCACTAAGTGATCAACGAACCAAGTTA

CCCTAGGGATAACAGCGCAATCCTTTCTAAGAGTCCATATCGACGAATGGGTTTACGACC

TCGATGTTGGATCAGGACACCCAAATGGTGCAGCCGCTATTAAGGTTCGTTTGTTCAACG

ATTAAAGTCCTACATGAACCCACATGCGCTATCAATCCTACTATCGAGCCTGGCAGTAGG

AACAATTACAACTCTCTCAAGCTCCCATTGATTCCTAGCATGAGCGGGCCTAGAAATTAA

TACACTAGCAATCATCCCACTAATAACAAAAATACACCACCCACGAGCAACAGAATCAGC

CACAAAATACTTCCTAACCCAGGCCGCCGCATCAGCCTTAATCTTATTTTCTACAATCAC

AAACGCATGGACTACAGGAGAATGAACAATTATTACCATAAATGACAACACATCAACATT

TATATTAACAATCGCCCTAGCCATTAAACTTGGAATCGCCCCATTTCACCTATGGTTACC

AGACGTCATACAAGGGTTAAGCCTAACAACATGCCTAATCCTTGCAACATGACAAAAATT

AGCCCCAATATCCTTAATAATTATAACTGGCCATCAGTTAAACACAAACCTACTAATTAC

AATAGCCGTGTTATCAACAGTAGTTGGGGGATGGGGGGGCCTTAACCAAACACAAATGCG

AAAAATAATAGCATACTCATCTATCGCCCACCTCGGATGAATAACACTAATCCTATCTTT

CTCCCCAAATTTAACAACCCTAAACCTAATAATCTACCTAATATTAACATCATCAATATT

CTTAATAATAACAACCCTAAACTCAACAAATATTAACAAACTTTCAACATCTTGACTAAA

AACGCCCCTACTAGCATCATCAATAATAATCACACTTATAGCCCTAGGAGGACTGCCCCC

TACATCAGGATTCCTACCAAAATGACTCATCCTACAAGAAATAACCAAACAACACCTAGG

GGCCGTATCTACACTAATAGCTATATCTGCCCTACTAAGCCTATTCTTCTACCTACGACT

ATCATATGCAATCTCACTAACTACTACACCAAACACTTCAAACTCCAACATAACCTGACG

AATAACCAACAACCAAATACAAATCCTACCAACAATAATTACACTAACTACAATAATATT

ACCAATCACACCAACACTACTAGCAATAGGACAAGGCAGCTTGGCGTACCCAGGTCTGAG

GACCCACGGGACTTTGGAGAGCATAGGTGGGCCTAAAGGCAGTTCAAGAGGAGGGTTGCC

CTCACTAGCAGACACTTTTGAACAAGTCATTGAGGAGATGCTTGAAGATGAGCAGAGCAT

GAGGCCCAGTGAGGAGAACAAGGATGCGGACATGTTTACATCTCGCGTAATGCTGAGCAG

TCAAGTGCCTTTGGAGCCTCCTCTTCTCTTTCTGCTGGAGGAGTACAAAAACTACTTGGA

TGCTGCAAATATGTCCATGAGGGTCCGACGTCACTCTGACCCCGCCCGCCGCGGGGAGCT

GAGTGTGTGTGACAGTATTAGTGAGTGGGTGACGGCATCTGACAAAAAGACTGCCGTGGA

CATGTCGGGGCAGACGGTAACTGTTCTAGAAAAAGTTCCAGTACCCAAAGGCCAACTGAA

GCAATACTTCTACGAGACCAAATGCAACCCTATGGGTTACATGAAGGAGGGCTGCAGAGG

CATTGACAAGAGGTACTGGAACTCCCAGTGCCGAACTACTCAGTCGTATGTGCGCGCGCT

CACCATGGATAACAAGAAGAAAGTGGGCTGGCGGTTTATCAGAATAGACACTTCATGTGT

ATGTACATTGACCATTGTTTGCTTGGGTTGCAGACCGAAGGCTGTTGTTCTACAAATATG

TCTACAAGCGATATAGAGCTGGGAAGCAAAGGGGTATGATTATAGAAACCGAAGGTGACA

GGCCTTCTTCAAAAGCTGACATTGAAATGGATGGAAAGATGGTCAACTCTCATACTGAAA

ATTTCATTGATGGCTCTCTAGTTCTGGAAGTCGATGAGAAAGATCAGGATGATGAAGAAG

CCAGGCGCGATATGGCCAGAATCCTAAAGGAGCTAAAGCAGAAACATCCTGACAAGGAAA

TAGAGCAACTGATTGAGTTAGCGAACTACCAGGTCTTGAGTCAACAGCAAAAAAGTCGAG

CATTCTATCGTATTCAAGCTACTCGCTTGATGACTGGGGCCGGAAATATTCTAAAGAGAC

ATGCAGCAGACCAGGCAAGGAAAGCTGTGAGCATGCAGGAAGTTAACTCTGAAGTGATCG

AAAATGAGCCCGTCAGTAAGATCTATTTCGAGCAAGC

>X48

CAACGGCCGCGGTATCATGACCGTGCAAAGGTAGCGTAATCACTTGTCTTTTAAATAAAG

ACCTGTATGAAAGGCAAAACGAAAGTTCAACTGTCTCTTTAATCCAATCAATGAAATTTA

TCTTTTCGTGCAGAAGCGGAAATAAATATATAAGACGAGAAGACCCTATGGAGCTTTAAA

CACACTATAATACAAGTTTTGGGTTGGGGCGACCGCGGAGAAAAAAAATCCTCCGAGATA

AGAATAACACTTCGAAAATTAAAACATTTTGATCCACTAAGTGATCAACGAACCAAGTTA

CCCTAGGGATAACAGCGCAATCCTTTCTAAGAGTCCATATCGACGAATGGGTTTACGACC

TCGATGTTGGATCAGGACACCCAAATGGTGCAGCCGCTATTAAGGTTCGTTTGTTCAACG

ATTAAAGTCCTACATGAACCCACATGCGCTATCAATCCTACTATCGAGCCTGGCAGTAGG

AACAATTACAACTCTCTCAAGCTCCCATTGATTCCTAGCATGAGCGGGCCTAGAAATTAA

TACACTAGCAATCATCCCACTAATAACAAAAATACACCACCCACGAGCAACAGAATCAGC

CACAAAATACTTCCTAACCCAGGCCGCCGCATCAGCCTTAATCTTATTTTCTACAATCAC

AAACGCATGGACTACAGGAGAATGAACAATTATTACCATAAATGACAACACATCAACATT

TATATTAACAATCGCCCTAGCCATTAAACTTGGAATCGCCCCATTTCACCTATGGTTACC

AGACGTCATACAAGGGTTAAGCCTAACAACATGCCTAATCCTTGCAACATGACAAAAATT

AGCCCCAATATCCTTAATAATTATAACTGGCCATCAGTTAAACACAAACCTACTAATTAC

AATAGCCGTGTTATCAACAGTAGTTGGGGGATGGGGGGGCCTTAACCAAACACAAATGCG

AAAAATAATAGCATACTCATCTATCGCCCACCTCGGATGAATAACACTAATCCTATCTTT

CTCCCCAAATTTAACAACCCTAAACCTAATAATCTACCTAATATTAACATCATCAATATT

CTTAATAATAACAACCCTAAACTCAACAAATATTAACAAACTTTCAACATCTTGACTAAA

AACGCCCCTACTAGCATCATCAATAATAATCACACTTATAGCCCTAGGAGGACTGCCCCC

TACATCAGGATTCCTACCAAAATGACTCATCCTACAAGAAATAACCAAACAACACCTAGG

GGCCGTATCTACACTAATAGCTATATCTGCCCTACTAAGCCTATTCTTCTACCTACGACT

ATCATATGCAATCTCACTAACTACTACACCAAACACTTCAAACTCCAACATAACCTGACG

AATAACCAACAACCAAATACAAATCCTACCAACAATAATTACACTAACTACAATAATATT

ACCAATCACACCAACACTACTAGCAATAGGACAAGGCAGCTTGGCGTACCCAGGTCTGAG

GACCCACGGGACTTTGGAGAGCATAGGTGGGCCTAAAGGCAGTTCAAGAGGAGGGTTGCC

CTCACTAGCAGACACTTTTGAACAAGTCATTGAGGAGATGCTTGAAGATGAGCAGAGCAT

GAGGCCCAGTGAGGAGAACAAGGATGCGGACATGTTTACATCTCGCGTAATGCTGAGCAG

TCAAGTGCCTTTGGAGCCTCCTCTTCTCTTTCTGCTGGAGGAGTACAAAAACTACTTGGA

TGCTGCAAATATGTCCATGAGGGTCCGACGTCACTCTGACCCCGCCCGCCGCGGGGAGCT

GAGTGTGTGTGACAGTATTAGTGAGTGGGTGACGGCATCTGACAAAAAGACTGCCGTGGA

CATGTCGGGGCAGACGGTAACTGTTCTAGAAAAAGTTCCAGTACCCAAAGGCCAACTGAA

GCAATACTTCTACGAGACCAAATGCAACCCTATGGGTTACATGAAGGAGGGCTGCAGAGG

CATTGACAAGAGGTACTGGAACTCCCAGTGCCGAACTACTCAGTCGTATGTGCGCGCGCT

CACCATGGATAACAAGAAGAAAGTGGGCTGGCGGTTTATCAGAATAGACACTTCATGTGT

ATGTACATTGACCATTGTTTGCTTGGGTTGCAGACCGAAGGCTGTTGTTCTACAAATATG

TCTACAAGCGATATAGAGCTGGGAAGCAAAGGGGTATGATTATAGAAACCGAAGGTGACA

GGCCTTCTTCAAAAGCTGACATTGAAATGGATGGAAAGATGGTCAACTCTCATACTGAAA

ATTTCATTGATGGCTCTCTAGTTCTGGAAGTCGATGAGAAAGATCAGGATGATGAAGAAG

CCAGGCGCGATATGGCCAGAATCCTAAAGGAGCTAAAGCAGAAACATCCTGACAAGGAAA

TAGAGCAACTGATTGAGTTAGCGAACTACCAGGTCTTGAGTCAACAGCAAAAAAGTCGAG

CATTCTATCGTATTCAAGCTACTCGCTTGATGACTGGGGCCGGAAATATTCTAAAGAGAC

ATGCAGCAGACCAGGCAAGGAAAGCTGTGAGCATGCAGGAAGTTAACTCTGAAGTGATCG

AAAATGAGCCCGTCAGTAAGATCTATTTCGAGCAAGC

>X49

CAACGGCCGCGGTATCATGACCGTGCAAAGGTAGCGTAATCACTTGTCTTTTAAATAAAG

ACCTGTATGAAAGGCAAAACGAAAGTTCAACTGTCTCTTTAATCCAATCAATGAAATTTA

TCTTTTCGTGCAGAAGCGGAAATAAATATATAAGACGAGAAGACCCTATGGAGCTTTAAA

CACACTATAATACAAGTTTTGGGTTGGGGCGACCGCGGAGAAAAAAAATCCTCCGAGATA

AGAATAACACTTCGAAAATTAAAACATTTTGATCCACTAAGTGATCAACGAACCAAGTTA

CCCTAGGGATAACAGCGCAATCCTTTCTAAGAGTCCATATCGACGAATGGGTTTACGACC

TCGATGTTGGATCAGGACACCCAAATGGTGCAGCCGCTATTAAGGTTCGTTTGTTCAACG

ATTAAAGTCCTACATGAACCCACATGCGCTATCAATCCTACTATCGAGCCTGGCAGTAGG

AACAATTACAACTCTCTCAAGCTCCCATTGATTCCTAGCATGAGCGGGCCTAGAAATTAA

TACACTAGCAATCATCCCACTAATAACAAAAATACACCACCCACGAGCAACAGAATCAGC

CACAAAATACTTCCTAACCCAGGCCGCCGCATCAGCCTTAATCTTATTTTCTACAATCAC

AAACGCATGGACTACAGGAGAATGAACAATTATTACCATAAATGACAACACATCAACATT

TATATTAACAATCGCCCTAGCCATTAAACTTGGAATCGCCCCATTTCACCTATGGTTACC

AGACGTCATACAAGGGTTAAGCCTAACAACATGCCTAATCCTTGCAACATGACAAAAATT

AGCCCCAATATCCTTAATAATTATAACTGGCCATCAGTTAAACACAAACCTACTAATTAC

AATAGCCGTGTTATCAACAGTAGTTGGGGGATGGGGGGGCCTTAACCAAACACAAATGCG

AAAAATAATAGCATACTCATCTATCGCCCACCTCGGATGAATAACACTAATCCTATCTTT

CTCCCCAAATTTAACAACCCTAAACCTAATAATCTACCTAATATTAACATCATCAATATT

CTTAATAATAACAACCCTAAACTCAACAAATATTAACAAACTTTCAACATCTTGACTAAA

AACGCCCCTACTAGCATCATCAATAATAATCACACTTATAGCCCTAGGAGGACTGCCCCC

TACATCAGGATTCCTACCAAAATGACTCATCCTACAAGAAATAACCAAACAACACCTAGG

GGCCGTATCTACACTAATAGCTATATCTGCCCTACTAAGCCTATTCTTCTACCTACGACT

ATCATATGCAATCTCACTAACTACTACACCAAACACTTCAAACTCCAACATAACCTGACG

AATAACCAACAACCAAATACAAATCCTACCAACAATAATTACACTAACTACAATAATATT

ACCAATCACACCAACACTACTAGCAATAGGACAAGGCAGCTTGGCGTACCCAGGTCTGAG

GACCCACGGGACTTTGGAGAGCATAGGTGGGCCTAAAGGCAGTTCAAGAGGAGGGTTGCC

CTCACTAGCAGACACTTTTGAACAAGTCATTGAGGAGATGCTTGAAGATGAGCAGAGCAT

GAGGCCCAGTGAGGAGAACAAGGATGCGGACATGTTTACATCTCGCGTAATGCTGAGCAG

TCAAGTGCCTTTGGAGCCTCCTCTTCTCTTTCTGCTGGAGGAGTACAAAAACTACTTGGA

TGCTGCAAATATGTCCATGAGGGTCCGACGTCACTCTGACCCCGCCCGCCGCGGGGAGCT

GAGTGTGTGTGACAGTATTAGTGAGTGGGTGACGGCATCTGACAAAAAGACTGCCGTGGA

CATGTCGGGGCAGACGGTAACTGTTCTAGAAAAAGTTCCAGTACCCAAAGGCCAACTGAA

GCAATACTTCTACGAGACCAAATGCAACCCTATGGGTTACATGAAGGAGGGCTGCAGAGG

CATTGACAAGAGGTACTGGAACTCCCAGTGCCGAACTACTCAGTCGTATGTGCGCGCGCT

CACCATGGATAACAAGAAGAAAGTGGGCTGGCGGTTTATCAGAATAGACACTTCATGTGT

ATGTACATTGACCATTGTTTGCTTGGGTTGCAGACCGAAGGCTGTTGTTCTACAAATATG

TCTACAAGCGATATAGAGCTGGGAAGCAAAGGGGTATGATTATAGAAACCGAAGGTGACA

GGCCTTCTTCAAAAGCTGACATTGAAATGGATGGAAAGATGGTCAACTCTCATACTGAAA

ATTTCATTGATGGCTCTCTAGTTCTGGAAGTCGATGAGAAAGATCAGGATGATGAAGAAG

CCAGGCGCGATATGGCCAGAATCCTAAAGGAGCTAAAGCAGAAACATCCTGACAAGGAAA

TAGAGCAACTGATTGAGTTAGCGAACTACCAGGTCTTGAGTCAACAGCAAAAAAGTCGAG

CATTCTATCGTATTCAAGCTACTCGCTTGATGACTGGGGCCGGAAATATTCTAAAGAGAC

ATGCAGCAGACCAGGCAAGGAAAGCTGTGAGCATGCAGGAAGTTAACTCTGAAGTGATCG

AAAATGAGCCCGTCAGTAAGATCTATTTCGAGCAAGC

>X50

CAACGGCCGCGGTATCATGACCGTGCAAAGGTAGCGTAATCACTTGTCTTTTAAATAAAG

ACCTGTATGAAAGGCAAAACGAAAGTTCAACTGTCTCTTTAATCCAATCAATGAAATTTA

TCTTTTCGTGCAGAAGCGGAAATGAATATATAAGACGAGAAGACCCTATGGAGCTTTAAA

CACGCTATAATACAAGTTTTTGGTTGGGGCGACCGCGGAGAAAAGAAATCCTCCGAGATA

AGAATAACACTTCGAAAATTAAAAAATTTTGATCCACTAAGTGACCAACGAACCAAGTTA

CCCTAGGGATAACAGCGCAATCCTTTCTAAGAGTCCATATCGACGAATGGGTTTACGACC

TCGATGTTGGATCAGGACACCCAAATGGTGCAGCCGCTATTAAGGTTCGTTTGTTCAACG

ATTAAAGTCCTACATGAACCCGTATGCACTATCAATCCTATTGTCGAGCCTAGCAGTAGG

AACAATTACAACTCTCTCAAGCTCCCATTGATTTCTGGCATGAGTAGGACTAGAAATTAA

TACACTAGCAATAATCCCCCTAATAACAAAAATACACCACCCACGATCAACAGAATCAGC

CACAAAATACTTCCTAACGCAAGCCACCGCATCGGCCTTAATCTTATTTTCTACAATCAT

AAACGCATGGACTACAGGAGAATGAACAATTATCACCATAAATGATAACTCATCAACATT

TATATTAACAGTTGCCCTAGCTATTAAACTTGGAATCGCCCCTTTTCATCTGTGACTACC

AGACGTCATACAAGGGTTAAACCTAACAACATGCCTAATCCTTGCAACATGACAAAAATT

AGCCCCAATATCCTTAATAATTATAACCAATCACCAATTAAACACAAACCTGCTAATTAC

AATAGCCGTATTATCAGCAGTAATTGGAGGATGAGGGGGACTTAACCAAACACAAATCCG

AAAAATAATAGCATATTCATCTATCGCCCACCTTGGGTGAATAACACTAGTCCTCTCTTT

CTCCCCAAATTTAACAACCCTTAACCTAATAATCTACCTAATACTAACATCATCAATGTT

CTTAATAATAACAACCCTAAACTCAACAAATATTAACAAACTTTCAACATCTTGACTGAA

AACCCCACTACTAGCATCATCAATAATAGTCACACTCATGGCCCTAGGAGGCCTACCCCC

CACATCAGGATTCCTACCAAAATGACTTATCCTACAAGAAATAACCAAACAGCACCTAGG

GGCCGTGTCCACACTAATAGCTATATCTGCACTACTAAGCCTATTCTTCTACTTACGACT

ATCATATGCAACCTCACTAACTACCGCACCAAACACATCAAACTCCAACATAACCTGACG

AATAACCCACAACCAAATACAAATCTTACCAACAATAATTATACTAACTACAATAATACT

ACCAATCACACCAACACTACTAGCAATAGGACAAGGCAGCTTGGCGTACCCAGGTCTGAG

GACCCACGGGACTTTGGAGAGCATAGGTGGGCCTAAAGGCAGTTCAAGAGGAGGGTTGCC

CTCACTAGCAGACACTTTTGAACAAGTCATTGAGGAGATGCTTGAAGATGAGCAGAGCAT

GAGGCCCAGTGAGGAGAACAAGGATGCGGACATGTTTACATCTCGCGTAATGCTGAGCAG

TCAAGTGCCTTTGGAGCCTCCTCTTCTCTTTCTGCTGGAGGAGTACAAAAACTACTTGGA

TGCTGCAAATATGTCCATGAGGGTCCGACGTCACTCTGACCCCGCCCGCCGCGGGGAGCT

GAGTGTGTGTGACAGTATTAGTGAGTGGGTGACGGCATCTGACAAAAAGACTGCCGTGGA

CATGTCGGGGCAGACGGTAACTGTTCTAGAAAAAGTTCCAGTACCCAAAGGCCAACTGAA

GCAATACTTCTACGAGACCAAATGCAACCCTATGGGTTACATGAAGGAGGGCTGCAGAGG

CATTGACAAGAGGTACTGGAACTCCCAGTGCCGAACTACTCAGTCGTATGTGCGCGCGCT

CACCATGGATAACAAGAAGAAAGTGGGCTGGCGGTTTATCAGAATAGACACTTCATGTGT

ATGTACATTGACCATTGTTTGCTTGGGTTGCAGACCGAAGGCTGTTGTTCTACAAATATG

TCTACAAGCGATATAGAGCTGGGAAGCAAAGGGGTATGATTATAGAAACCGAAGGTGACA

GGCCTTCTTCAAAAGCTGACATTGAAATGGATGGAAAGATGGTCAACTCTCATACTGAAA

ATTTCATTGATGGCTCTCTAGTTCTGGAAGTCGATGAGAAAGATCAGGATGATGAAGAAG

CCAGGCGCGATATGGCCAGAATCCTAAAGGAGCTAAAGCAGAAACATCCTGACAAGGAAA

TAGAGCAACTGATTGAGTTAGCGAACTACCAGGTCTTGAGTCAACAGCAAAAAAGTCGAG

CATTCTATCGTATTCAAGCTACTCGCTTGATGACTGGGGCCGGAAATATTCTAAAGAGAC

ATGCAGCAGACCAGGCAAGGAAAGCTGTGAGCATGCAGGAAGTTAACTCTGAAGTGATCG

AAAATGAGCCCGTCAGTAAGATCTATTTCAAGCAAGC

>X51

CAACGGCCGCGGTATCATGACCGTGCAAAGGTAGCGTAATCACTTGTCTTTTAAATAAAG

ACCTGTATGAAAGGCAAAACGAAAGTTCAACTGTCTCTTTAATCCAATCAATGAAATTTA

TCTTTTCGTGCAGAAGCGGAAATGAATATATAAGACGAGAAGACCCTATGGAGCTTTAAA

CACGCTATAATACAAGTTTTTGGTTGGGGCGACCGCGGAGAAAAGAAATCCTCCGAGATA

AGAATAACACTTCGAAAATTAAAAAATTTTGATCCACTAAGTGACCAACGAACCAAGTTA

CCCTAGGGATAACAGCGCAATCCTTTCTAAGAGTCCATATCGACGAATGGGTTTACGACC

TCGATGTTGGATCAGGACACCCAAATGGTGCAGCCGCTATTAAGGTTCGTTTGTTCAACG

ATTAAAGTCCTACATGAACCCGTATGCACTATCAATCCTATTGTCGAGCCTAGCAGTAGG

AACAATTACAACTCTCTCAAGCTCCCATTGATTTCTGGCATGAGTAGGACTAGAAATTAA

TACACTAGCAATAATCCCCCTAATAACAAAAATACACCACCCACGATCAACAGAATCAGC

CACAAAATACTTCCTAACGCAAGCCACCGCATCGGCCTTAATCTTATTTTCTACAATCAT

AAACGCATGGACTACAGGAGAATGAACAATTATCACCATAAATGATAACTCATCAACATT

TATATTAACAGTTGCCCTAGCTATTAAACTTGGAATCGCCCCTTTTCATCTGTGACTACC

AGACGTCATACAAGGGTTAAACCTAACAACATGCCTAATCCTTGCAACATGACAAAAATT

AGCCCCAATATCCTTAATAATTATAACCAATCACCAATTAAACACAAACCTGCTAATTAC

AATAGCCGTATTATCAGCAGTAATTGGAGGATGAGGGGGACTTAACCAAACACAAATCCG

AAAAATAATAGCATATTCATCTATCGCCCACCTTGGGTGAATAACACTAGTCCTCTCTTT

CTCCCCAAATTTAACAACCCTTAACCTAATAATCTACCTAATACTAACATCATCAATGTT

CTTAATAATAACAACCCTAAACTCAACAAATATTAACAAACTTTCAACATCTTGACTGAA

AACCCCACTACTAGCATCATCAATAATAGTCACACTCATGGCCCTAGGAGGCCTACCCCC

CACATCAGGATTCCTACCAAAATGACTTATCCTACAAGAAATAACCAAACAGCACCTAGG

GGCCGTGTCCACACTAATAGCTATATCTGCACTACTAAGCCTATTCTTCTACTTACGACT

ATCATATGCAATCTCACTAACTACCGCACCAAACACATCAAACTCCAACATAACCTGACG

AATAACCCACAACCAAATACAAATCTTACCAACAATAATTATACTAACTACAATAATATT

ACCAATCACACCAACACTACTAGCAATAGGACAAGGCAGCTTGGCGTACCCAGGTCTGAG

GACCCACGGGACTTTGGAGAGCATAGGTGGGCCTAAAGGCAGTTCAAGAGGAGGGTTGCC

CTCACTAGCAGACACTTTTGAACAAGTCATTGAGGAGATGCTTGAAGATGAGCAGAGCAT

GAGGCCCAGTGAGGAGAACAAGGATGCGGACATGTTTACATCTCGCGTAATGCTGAGCAG

TCAAGTGCCTTTGGAGCCTCCTCTTCTCTTTCTGCTGGAGGAGTACAAAAACTACTTGGA

TGCTGCAAATATGTCCATGAGGGTCCGACGTCACTCTGACCCCGCCCGCCGCGGGGAGCT

GAGTGTGTGTGACAGTATTAGTGAGTGGGTGACGGCATCTGACAAAAAGACTGCCGTGGA

CATGTCGGGGCAGACGGTAACTGTTCTAGAAAAAGTTCCAGTACCCAAAGGCCAACTGAA

GCAATACTTCTACGAGACCAAATGCAACCCTATGGGTTACATGAAGGAGGGCTGCAGAGG

CATTGACAAGAGGTACTGGAACTCCCAGTGCCGAACTACTCAGTCGTATGTGCGCGCGCT

CACCATGGATAACAAGAAGAAAGTGGGCTGGCGGTTTATCAGAATAGACACTTCATGTGT

ATGTACATTGACCATTGTTTGCTTGGGTTGCAGACCGAAGGCTGTTGTTCTACAAATATG

TCTACAAGCGATATAGAGCTGGGAAGCAAAGGGGTATGATTATAGAAACCGAAGGTGACA

GGCCTTCTTCAAAAGCTGACATTGAAATGGATGGAAAGATGGTCAACTCTCATACTGAAA

ATTTCATTGATGGCTCTCTAGTTCTGGAAGTCGATGAGAAAGATCAGGATGATGAAGAAG

CCAGGCGCGATATGGCCAGAATCCTAAAGGAGCTAAAGCAGAAACATCCTGACAAGGAAA

TAGAGCAACTGATTGAGTTAGCGAACTACCAGGTCTTGAGTCAACAGCAAAAAAGTCGAG

CATTCTATCGTATTCAAGCTACTCGCTTGATGACTGGGGCCGGAAATATTCTAAAGAGAC

ATGCAGCAGACCAGGCAAGGAAAGCTGTGAGCATGCAGGAAGTTAACTCTGAAGTGATCG

AAAATGAGCCCGTCAGTAAGATCTATTTCAAGCAAGC

>X52

CAACGGCCGCGGTATCATGACCGTGCAAAGGTAGCGTAATCACTTGTCTTTTAAATAAAG

ACCTGTATGAAAGGCAAAACGAAAGTTCAACTGTCTCTTTAATCCAATCAATGAAATTTA

TCTTTTCGTGCAGAAGCGGAAATGAATATATAAGACGAGAAGACCCTATGGAGCTTTAAA

CACGCTATAATACAAGTTTTTGGTTGGGGCGACCGCGGAGAAAAGAAATCCTCCGAGATA

AGAATAACACTTCGAAAATTAAAAAATTTTGATCCACTAAGTGACCAACGAACCAAGTTA

CCCTAGGGATAACAGCGCAATCCTTTCTAAGAGTCCATATCGACGAATGGGTTTACGACC

TCGATGTTGGATCAGGACACCCAAATGGTGCAGCCGCTATTAAGGTTCGTTTGTTCAACG

ATTAAAGTCCTACATGAACCCGTATGCACTATCAATCCTGTTGTCGAGCCTAGCAGTAGG

AACAATTACAACTCTCTCAAGCTCCCATTGATTTCTGGCATGAGTAGGACTAGAAATTAA

TACACTAGCAATAATCCCCCTAATAACAAAAATACACCACCCACGATCAACAGAATCAGC

CACAAAATACTTCCTAACGCAAGCCACCGCATCGGCCTTAATCTTATTTTCTACAATCAT

AAACGCATGGACTACAGGAGAATGAACAATTATCACCATAAATGATAACTCATCAACATT

TATATTAACAGTTGCCCTAGCTATTAAACTTGGAATCGCCCCTTTTCATCTGTGACTACC

AGACGTCATACAAGGGTTAAACCTAACAACATGCCTAATCCTTGCAACATGACAAAAATT

AGCCCCAATATCCTTAATAATTATAACCAATCACCAATTAAACACAAACCTGCTAATTAC

AATAGCCGTATTATCAGCAGTAATTGGAGGATGAGGGGGACTTAACCAAACACAAATCCG

AAAAATAATAGCATATTCATCTATCGCCCACCTTGGGTGAATAACACTAGTCCTCTCTTT

CTCCCCAAATTTAACAACCCTTAACCTAATAATCTACCTAATACTAACATCATCAATGTT

CTTAATAATAACAACCATAAACTCAACAAATATTAACAAACTTTCAACATCTTGACTGAA

AACCCCACTACTAGCATCATCAATAATAGTCACACTCATGGCCCTAGGAGGCCTACCCCC

AACATCAGGATTCCTACCAAAATGACTTATCCTACAAGAAATAACCAAACAGCACCTAGG

GGCCGTGTCCACACTAATAGCTATATCTGCACTACTAAGCCTATTCTTGTGATTACGACT

ATCATATGCAATCTCACTAACTACCGCACCAAACACATCAAACTCCAACATAACCTGACG

AATAACCCACAACCAAATACAAATCTTACCAACAATAATCATACTAACTACAATAATATT

ACCAATCACACCAACACTACTAGCAATAGGACAAGGCAGCTTGGCGTACCCAGGTCTGAG

GACCCACGGGACTTTGGAGAGCATAGGTGGGCCTAAAGGCAGTTCAAGAGGAGGGTTGCC

CTCACTAGCAGACACTTTTGAACAAGTCATTGAGGAGATGCTTGAAGATGAGCAGAGCAT

GAGGCCCAGTGAGGAGAACAAGGATGCCGACATGTTTACATCTCGCGTAATGCTGAGCAG

TCAAGTGCCTTTGGAGCCTCCTCTTCTCTTTCTGCTGGAGGAGTACAAAAACTACTTGGA

TGCTGCAAATATGTCCATGAGGGTCCGACGTCACTCTGACCCCGCCCGCCGCGGGGAGCT

GAGTGTGTGTGACAGTATTAGTGAGTGGGTGACGGCATCTGACAAAAAGACTGCCGTGGA

CATGTCGGGGCAGACGGTAACTGTTCTAGAAAAAGTTCCAGTACCCAAAGGCCAACTGAA

GCAATACTTCTACGAGACCAAATGCAACCCTATGGGTTACATGAAGGAGGGCTGCAGAGG

CATTGACAAGAGGTACTGGAACTCCCAGTGCCGAACTACTCAGTCGTATGTGCGCGCGCT

CACCATGGATAACAAGAAGAAAGTGGGCTGGCGGTTTATCAGAATAGACACTTCATGTGT

ATGTACATTGACCATTGTTTGCTTGGGTTGCAGACCGAAGGCTGTTGTTCTACAAATATG

TCTACAAGCGATATAGAGCTGGGAAGCAAAGGGGTATGATTATAGAAACCGAAGGTGACA

GGCCTTCTTCAAAAGCTGACATTGAAATGGATGGAAAGATGGTCAACTCTCATACTGAAA

ATTTCATTGATGGCTCTCTAGTTCTGGAAGTCGATGAGAAAGATCAGGATGATGAAGAAG

CCAGGCGCGATATGGCCAGAATCCTAAAGGAGCTAAAGCAGAAACATCCTGACAAGGAAA

TAGAGCAACTGATTGAGTTAGCGAACTACCAGGTCTTGAGTCAACAGCAAAAAAGTCGAG

CATTCTATCGTATTCAAGCTACTCGCTTGATGACTGGGGCCGGAAATATTCTAAAGAGAC

ATGCAGCAGACCAGGCAAGGAAAGCTGTGAGCATGCAGGAAGTTAACTCTGAAGTGATCG

AAAATGAGCCCGTCAGTAAGATCTATTTCAAGCAAGC

>X55

CAACGGCCGCGGTATCATGACCGTGCAAAGGTAGCGTAATCACTTGTCTTTTAAATAAAG

ACCTGTATGAAAGGCAAAACGAAAGTTCAACTGTCTCTTTAATCCAATCAATAAAATTTA

TCTTTCCGTGCAGAAGCGGAAATAAATATATAAGACGAGAAGACCCTATGGAGCTTTAAA

CACACCATAATATAAGTTTTAGGTTGGGGCGACCGCGGAAGAAAAAAATCCTCCGAGATA

AGAATAACACTTCGAAAATTAAAACATTTTGATCCACTAAGTGACCAACGAACCAAGTTA

CCCTAGGGATAACAGCGCAATCCTTTCTAAGAGTCCATATCGACGAACGGGTTTACGACC

TCGATGTTGGATCAGGACACCCAAATGGTGCAGCCGCTATTAAGGTTCGTTTGTTCAACG

ATTAAAGTCCTACATGAACCCATATGCACTGTCAATTCTATTGTCGAGCCTAGCAGTAGG

AACAATCACAACCCTTTCAAGCTCCCATTGATTCCTCGCATGAGTGGGCCTAGAAATTAA

TACACTAGCAATCATCCCGCTAATAACAAAAATACACCACCCACGAGCAACGGAATCAGC

CACAAAATACTTTCTAACGCAAGCCACCGCATCAGCCCTAATCTTATTTTCCACAATCAC

AAACGCATGGGCCACAGGAGAATGAACAATTATTACCATAAATGACAACACGTCAACATT

TATCTTAACAATTGCCCTGGCCATCAAACTGGGAATCGCCCCATTTCACCTCTGATTACC

AGACGTTATACAAGGATTAACCCTAACAACATGTCTAATCCTTACAACATGACAAAAATT

AGCCCCAATATCCTTAATAATTATAACCGGCCATCAATTAAACACAAACCTACTAATTAC

AATGGCCATATTATCAACAGTACTTGGGGGGTGGGGGGGCCTTAACCAAACACAAATGCG

AAAAATAATAGCATATTCATCTATTGCCCACCTCGGATGAATAACCCTAATCCTATCCTT

CTCCCCAAGCTTAACAACATTAAACCTAGTAATCTACCTAATACTAACCTCATCAATATT

CTTAATAATAATAACAATAACCTCAACAAATATAAATAAACTCTCAACATCTTGACTGAA

GACCCCCCTACTGGCATCATCAATGATAATTACACTTATAGCCCTGGGGGGCCTACCCCC

GACCTCAGGCTTCTTGCCAAAATGATTAATCCTACAAGAAATAACCAAACAACACCTGGG

AGCTGTATCTGCACTAATAGCTATATCTGCCCTACTAAGCCTATTCTTCTACCTACGACT

ATCATATGCGGTCTCATTAACCACTGCACCAAACACCTCAAACTCCAACATGACCTGACG

AATAACCCACAACCATATACAAATCCACCCCACAATAATTACACTAACTACAATAATACT

ACCAATCACACCAACACTACTAACAATAGGACAAGGCAGCTTGGAGTACCCAGGTCTGAG

GACCCACGGGACTTTGGAGAGCATAGGTGGGCCTAAAGGCAGTTCAAGAGGAGGGTTGCC

CTCACTAGCAGACACTTTTGAACAAGTCATTGAGGAGATGCTTGAAGATGAGCAGAGCAT

GAGGCCCAGTGAGGAGAACAAGGATGCGGACATGTTTACATCTCGCGTAATGCTGAGCAG

TCAAGTGCCTTTGGAGCCTCCTCTTCTCTTTCTGCTGGAGGAGTACAAAAACTACTTGGA

TGCTGCAAATATGTCCATGAGGGTCCGACGTCACTCTGACCCCGCCCGCCGCGGGGAGCT

GAGTGTGTGTGACAGTATTAGTGAGTGGGTGACGGCATCTGACAAAAAGACTGCCGTGGA

CATGTCGGGGCAGACGGTAACTGTTCTAGAAAAAGTTCCAGTACCCAAAGGCCAACTGAA

GCAATACTTCTACGAGACCAAATGCAACCCTATGGGTTACATGAAGGAGGGCTGCAGAGG

CATTGACAAGAGGTACTGGAACTCCCAGTGCCGAACTACTCAGTCGTATGTGCGCGCGCT

CACCATGGATAACAAGAAGAAAGTGGGCTGGCGGTTTATCAGAATAGACACTTCATGTGT

ATGTACATTGACCATTGTTTGCTTGGGTTGCAGACCGAAGGCTGTTGTTCTACAAATATG

TCTACAAGCGATATAGAGCTGGGAAGCAAAGGGGTATGATTATAGAAACCGAAGGTGACA

GGCCTTCTTCAAAAGCTGACATTGAAATGGATGGAAAGATGGTCAACTCTCATACTGAAA

ATTTCATTGATGGCTCTCTAGTTCTGGAAGTCGATGAGAAAGATCAGGATGATGAAGAAG

CCAGGCGCGATATGGCCAGAATCCTAAAGGAGCTAAAGCAGAAACATCCTGACAAGGAAA

TAGAGCAACTGATTGAGTTAGCGAACTACCAGGTCTTGAGTCAACAGCAAAAAAGTCGAG

CATTCTATCGTATTCAAGCTACTCGCTTGATGACTGGGGCCGGAAATATTCTAAAGAGAC

ATGCAGCAGACCAGGCAAGGAAAGCTGTGAGCATGCAGGAAGTTAACTCTGAAGTGATCG

AAAATGAACCCGTCAGTAAGATCTATTTCGAGCAAGC

>X56

CAACGGCCGCGGTATCATGACCGTGCAAAGGTAGCGTAATCACTTGTCTTTTAAATAAAG

ACCTGTATGAAAGGCAAAACGAAAGTTCAACTGTCTCTTTAATCCAATCAATAAAATTTA

TCTTTCCGTGCAGAAGCGGAAATAAATATATAAGACGAGAAGACCCTATGGAGCTTTAAA

CACACCATAATATAAGTTTTAGGTTGGGGCGACCGCGGAAGAAAAAAATCCTCCGAGATA

AGAATAACACTTCGAAAATTAAAACATTTTGATCCACTAAGTGACCAACGAACCAAGTTA

CCCTAGGGATAACAGCGCAATCCTTTCTAAGAGTCCATATCGACGAACGGGTTTACGACC

TCGATGTTGGATCAGGACACCCAAATGGTGCAGCCGCTATTAAGGTTCGTTTGTTCAACG

ATTAAAGTCCTACATGAACCCATATGCACTGTCAATTCTATTGTCGAGCCTAGCAGTAGG

AACAATCACAACCCTTTCAAGCTCCCATTGATTCCTCGCATGAGTGGGCCTAGAAATTAA

TACACTAGCAATCATCCCGCTAATAACAAAAATACACCACCCACGAGCAACGGAATCAGC

CACAAAATACTTTCTAACGCAAGCCACCGCATCAGCCCTAATCTTATTTTCCACAATCAC

AAACGCATGGGCCACAGGAGAATGAACAATTATTACCATAAATGACAACACGTCAACATT

TATCTTAACAATTGCCCTGGCCATCAAACTGGGAATCGCCCCATTTCACCTCTGATTACC

AGACGTTATACAAGGATTAACCCTAACAACATGTCTAATCCTTACAACATGACAAAAATT

AGCCCCAATATCCTTAATAATTATAACCGGCCATCAATTAAACACAAACCTACTAATTAC

AATGGCCATATTATCAACAGTACTTGGGGGGTGGGGGGGCCTTAACCAAACACAAATGCG

AAAAATAATAGCATATTCATCTATTGCCCACCTCGGATGAATAACCCTAATCCTATCCTT

CTCCCCAAGCTTAACAACATTAAACCTAGTAATCTACCTAATACTAACCTCATCAATATT

CTTAATAATAATAACAATAACCTCAACAAATATAAATAAACTCTCAACATCTTGACTGAA

GACCCCCCTACTGGCATCATCAATGATAATTACACTTATAGCCCTGGGGGGCCTACCCCC

GACCTCAGGCTTCTTGCCAAAATGATTAATCCTACAAGAAATAACCAAACAACACCTGGG

AGCTGTATCTGCACTAATAGCTATATCTGCCCTACTAAGCCTATTCTTCTACCTACGACT

ATCATATGCGGTCTCATTAACCACTGCACCAAACACCTCAAACTCCAACATGACCTGACG

AATAACCCACAACCATATACAAATCCACCCCACAATAATTACACTAACTACAATAATACT

ACCAATCACACCAACACTACTAACAATAGGACAAGGCAGCTTGGCGTACCCAGGTCTGAG

GACCCACGGGACTTTGGAGAGCATAGGTGGGCCTAAAGGCAGTTCAAGAGGAGGGTTGCC

CTCACTAGCAGACACTTTTGAACAAGTCATTGAGGAGATGCTTGAAGATGAGCAGAGCAT

GAGGCCCAGTGAGGAGAACAAGGATGCGGACATGTTTACATCTCGCGTAATGCTGAGCAG

TCAAGTGCCTTTGGAGCCTCCTCTTCTCTTTCTGCTGGAGGAGTACAAAAACTACTTGGA

TGCTGCAAATATGTCCATGAGGGTCCGACGTCACTCTGACCCCGCCCGCCGCGGGGAGCT

GAGTGTGTGTGACAGTATTAGTGAGTGGGTGACGGCATCTGACAAAAAGACTGCCGTGGA

CATGTCGGGGCAGACGGTAACTGTTCTAGAAAAAGTTCCAGTACCCAAAGGCCAACTGAA

GCAATACTTCTACGAGACCAAATGCAACCCTATGGGTTACATGAAGGAGGGCTGCAGAGG

CATTGACAAGAGGTACTGGAACTCCCAGTGCCGAACTACTCAGTCGTATGTGCGCGCGCT

CACCATGGATAACAAGAAGAAAGTGGGCTGGCGGTTTATCAGAATAGACACTTCATGTGT

ATGTACATTGACCATTGTTTGCTTGGGTTGCAGACCGAAGGCTGTTGTTCTACAAATATG

TCTACAAGCGATATAGAGCTGGGAAGCAAAGGGGTATGATTATAGAAACCGAAGGTGACA

GGCCTTCTTCAAAAGCTGACATTGAAATGGATGGAAAGATGGTCAACTCTCATACTGAAA

ATTTCATTGATGGCTCTCTAGTTCTGGAAGTCGATGAGAAAGATCAGGATGATGAAGAAG

CCAGGCGCGATATGGCCAGAATCCTAAAGGAGCTAAAGCAGAAACATCCTGACAAGGAAA

TAGAGCAACTGATTGAGTTAGCGAACTACCAGGTCTTGAGTCAACAGCAAAAAAGTCGAG

CATTCTATCGTATTCAAGCTACTCGCTTGATGACTGGGGCCGGAAATATTCTAAAGAGAC

ATGCAGCAGACCAGGCAAGGAAAGCTGTGAGCATGCAGGAAGTTAACTCTGAAGTGATCG

AAAATGAACCCGTCAGTAAGATCTATTTCGAGCAAGC

>X57

CAACGGCCGCGGTATCATGACCGTGCAAAGGTAGCGTAATCACTTGTCTTTTAAATAAAG

ACCCGTATGAAAGGCAAAACGAAAGTTCAACTGTCTCTTTAATCCAATCAATGAAATTTA

TCTTTTCGTGCAGAGGCGGAGATAAATATATAAGACGAGAAGACCCTATGGAGCTTTAAA

CACACTATAGTATAAGTTTTGGGTTGGGGCGACCGCGGAGCAAAAAAATCCTCCGAGATA

AGAATAACACTTCGAAAATTAAAACATTTTGATCCACTAAGTGACCAACGAACCAAGTTA

CCCTAGGGATAACAGCGCAATCCTTTCTAAGAGTCCATATCGACGAATGGGTTTACGACC

TCGATGTTGGATCAGGACACCCAAATGGTGCAGCCGCTGTTAAGGTTCGTTTGTTCAACG

ATTAAAGTCCTACATGAACCCATATGCACTCTCAATTCTATTATCAAGCCTGGCAGTAGG

AACAATTACAACTCTCTCAAGCTCCCACTGATTTCTCGCATGACTAGGCTTAGAAATTAA

TACACTAGCAATCATCCCCCTGATAACAAAAATACACCACCCACGAGCAACAGAATCAGC

CACAAAATACTTCCTAACGCAAGCCACCGCATCAGCCCTAATCTTATTTTCTACAATCAC

AAACGCATGGGCCACAGGAGAATGAACAATTATCACCATAAATGACAACACATCAACACT

TATCTTAACAATCGCCCTAGCCACCAAACTGGGAATCGCCCCATTTCATCTGTGATTACC

AGACGTCATACAAGGACTAAACCTAGCAACATGTTTAATCCTTACAACATGGCAAAAACT

AGCCCCAATATCATTAATAATCATAACTAGCCATCAATTAAACACAAACCTACTAATTAC

AATAGCCATACTATCAACAGTAATTGGGGGGTGGGGGGGCCTTAACCAAACACAAATACG

AAAAATAATAGCATATTCATCTATCGCCCACCTCGGATGAATAACACTAATCCTATCTTT

CTCCCCAAACTTAACGACCCTAAACTTAATAATTTACCTAATACTAACCTCATCAATATT

TTTAATAATAATAACCATAACCTCAACAAATATAAACAAACTTTCAACATCTTGACTGAA

AACGCCACTACTAGCATCATCAATAATAGTTACACTCATAGCCCTTGGAGGCCTACCCCC

AACCTCAGGCTTCTTACCAAAATGACTAATCCTACAAGAAATAACTAAACAACACCTAGT

AGCCGTATCTACACTAATAGCTATATCTGCCCTACTAAGCCTTTTCTTCTATTTACGACT

ATCATATGCGATCTCACTAACCACTGCGCCAAACACCTCAAACTCCAACATAACCTGACG

AGTAGCCCACAACCAAATACAAGTCCTCCCAACAATAATCACACTAACTACAATAATACT

ACCAATCACACCAACACTACTAGCAACAGGACAAGGCAGCTTGGCGTACCCAGGTCTGAG

GACCCACGGGACTTTGGAGAGCATAGGTGGGCCTAAAGGCAGTTCAAGAGGAGGGTTGCC

CTCACTAGCAGACACTTTTGAACAAGTCATTGAGGAGATGCTTGAAGATGAGCAGAGCAT

GAGGCCCAGTGAGGAGAACAAGGATGCCGACATGTTTACATCTCGCGTAATGCTGAGCAG

TCAAGTGCCTTTGGAGCCTCCTCTTCTCTTTCTGCTGGAGGAGTACAAAAACTACTTGGA

TGCTGCAAATATGTCCATGAGGGTCCGACGTCACTCTGACCCCGCCCGCCGCGGGGAGCT

GAGTGTGTGTGACAGTATTAGTGAGTGGGTGACGGCATCTGACAAAAAGACTGCCGTGGA

CATGTCGGGGCAGACGGTAACTGTTCTAGAAAAAGTTCCAGTACCCAAAGGCCAACTGAA

GCAATACTTCTACGAGACCAAATGCAACCCTATGGGTTACATGAAGGAGGGCTGCAGAGG

CATTGACAAGAGGTACTGGAACTCCCAGTGCCGAACTACTCAGTCGTATGTGCGCGCGCT

CACCATGGATAACAAGAAGAAAGTGGGCTGGCGGTTTATCAGAATAGACACTTCATGTGT

ATGTACATTGACCATTGTTTGCTTGGGTTGCAGACCGAAGGCTGTTGTTCTACAAATATG

TCTACAAGCGATATAGAGCTGGGAAGCAAAGGGGTATGATTATAGAAACCGAAGGTGACA

GGCCTTCTTCAAAAGCTGACATTGAAATGGATGGAAAGATGGTCAACTCTCATACTGAAA

ATTTCATTGATGGCTCTCTAGTTCTGGAAGTCGATGAGAAAGATCAGGATGATGAAGAAG

CCAGGCGCGATATGGCCAGAATCCTAAAGGAGCTAAAGCAGAAACATCCTGACAAGGAAA

TAGAGCAACTGATTGAGTTAGCGAACTACCAGGTCTTGAGTCAACAGCAAAAAAGTCGAG

CATTCTATCGTATTCAAGCTACTCGCTTGATGACTGGGGCCGGAAATATTCTAAAGAGAC

ATGCAGCAGACCAGGCAAGGAAAGCTGTGAGCATGCAGGAAGTTAACTCTGAAGTGATCG

AAAATGAGCCCGTCAGTAAGATCTATTTCGAGCAAGC

>X58

CAACGGCCGCGGTATCATGACCGTGCAAAGGTAGCGTAATCACTTGTCTTTTAAATAAAG

ACCCGTATGAAAGGCAAAACGAAAGTTCAACTGTCTCTTTAATCCAATCAATGAAATTTA

TCTTTTCGTGCAGAGGCGGAGATAAATATATAAGACGAGAAGACCCTATGGAGCTTTAAA

CACACTATAGTATAAGTTTTGGGTTGGGGCGACCGCGGAGCAAAAAAATCCTCCGAGATA

AGAATAACACTTCGAAAATTAAAACATTTTGATCCACTAAGTGACCAACGAACCAAGTTA

CCCTAGGGATAACAGCGCAATCCTTTCTAAGAGTCCATATCGACGAATGGGTTTACGACC

TCGATGTTGGATCAGGACACCCAAATGGTGCAGCCGCTGTTAAGGTTCGTTTGTTCAACG

ATTAAAGTCCTACATGAACCCATATGCACTCTCAATTCTATTATCAAGCCTGGCAGTAGG

AACAATTACAACTCTCTCAAGCTCCCACTGATTTCTCGCATGACTAGGCTTAGAAATTAA

TACACTAGCAATCATCCCCCTGATAACAAAAATACACCACCCACGAGCAACAGAATCAGC

CACAAAATACTTCCTAACGCAAGCCACCGCATCAGCCCTAATCTTATTTTCTACAATCAC

AAACGCATGGGCCACAGGAGAATGAACAATTATCACCATAAATGACAACACATCAACACT

TATCTTAACAATCGCCCTAGCCACCAAACTGGGAATCGCCCCATTTCATCTGTGATTACC

AGACGTCATACAAGGACTAAACCTAGCAACATGTTTAATCCTTACAACATGGCAAAAACT

AGCCCCAATATCATTAATAATCATAACTAGCCATCAATTAAACACAAACCTACTAATTAC

AATAGCCATACTATCAACAGTAATTGGGGGGTGGGGGGGCCTTAACCAAACACAAATACG

AAAAATAATAGCATATTCATCTATCGCCCACCTCGGATGAATAACACTAATCCTATCTTT

CTCCCCAAACTTAACGACCCTAAACTTAATAATTTACCTAATACTAACCTCATCAATATT

TTTAATAATAATAACCATAACCTCAACAAATATAAACAAACTTTCAACATCTTGACTGAA

AACGCCACTACTAGCATCATCAATAATAGTTACACTCATAGCCCTTGGAGGCCTACCCCC

AACCTCAGGCTTCTTACCAAAATGACTAATCCTACAAGAAATAACTAAACAACACCTAGT

AGCCGTATCTACACTAATAGCTATATCTGCCCTACTAAGCCTTTTCTTCTATTTACGACT

ATCATATGCGATCTCACTAACCACTGCGCCAAACACCTCAAACTCCAACATAACCTGACG

AGTAGCCCACAACCAAATACAAGTCCTCCCAACAATAATCACACTAACTACAATAATACT

ACCAATCACACCAACACTACTAGCAACAGGACAAGGCAGCTTGGCGTACCCAGGTTTGAG

GACCCACGGGACTTTGGAGAGCATAGGTGGGCCTAAAGGCAGTTCAAGAGGAGGGTTGCC

CTCACTAGCAGACACTTTTGAACAAGTCATTGAGGAGATGCTTGAAGATGAGCAGAGCAT

GAGGCCCAGTGAGGAGAACAAGGATGCCGACATGTTTACATCTCGCGTAATGCTGAGCAG

TCAAGTGCCTTTGGAGCCTCCTCTTCTCTTTCTGCTGGAGGAGTACAAAAACTACTTGGA

TGCTGCAAATATGTCCATGAGGGTCCGACGTCACTCTGACCCCGCCCGCCGCGGGGAGCT

GAGTGTGTGTGACAGTATTAGTGAGTGGGTGACGGCATCTGACAAAAAGACTGCCGTGGA

CATGTCGGGGCAGACGGTAACTGTTCTAGAAAAAGTTCCAGTACCCAAAGGCCAACTGAA

GCAATACTTCTACGAGACCAAATGCAACCCTATGGGTTACATGAAGGAGGGCTGCAGAGG

CATTGACAAGAGGTACTGGAACTCCCAGTGCCGAACTACTCAGTCGTATGTGCGCGCGCT

CACCATGGATAACAAGAAGAAAGTGGGCTGGCGGTTTATCAGAATAGACACTTCATGTGT

ATGTACATTGACCATTGTTTGCTTGGGTTGCAGACCGAAGGCTGTTGTTCTACAAATATG

TCTACAAGCGATATAGAGCTGGGAAGCAAAGGGGTATGATTATAGAAACCGAAGGTGACA

GGCCTTCTTCAAAAGCTGACATTGAAATGGATGGAAAGATGGTCAACTCTCATACTGAAA

ATTTCATTGATGGCTCTCTAGTTCTGGAAGTCGATGAGAAAGATCAGGATGATGAAGAAG

CCAGGCGCGATATGGCCAGAATCCTAAAGGAGCTAAAGCAGAAACATCCTGACAAGGAAA

TAGAGCAACTGATTGAGTTAGCGAACTACCAGGTCTTGAGTCAACAGCAAAAAAGTCGAG

CATTCTATCGTATTCAAGCTACTCGCTTGATGACTGGGGCCGGAAATATTCTAAAGAGAC

ATGCAGCAGACCAGGCAAGGAAAGCTGTGAGCATGCAGGAAGTTAACTCTGAAGTGATCG

AAAATGAGCCCGTCAGTAAGATCTATTTCGAGCAAGC

>X74

CAACGGCCGCGGTATCATGACCGTGCAAAGGTAGCGTAATCACTTGTCTTTTAAATAAAG

ACCCGTATGAAAGGCAAAACGAAAGTTCAACTGTCTCTTTAATCCAATCAATGAAATTTA

TCTTTTCGTGCAGAGGCGGAGATAAACATATAAGACGAGAAGACCCTATGGAGCTTTAAA

CACACTATAATATAAGTTTTAGGTTGGGGCGACCACGGAGCAAAAAAATCCTCCGAGATA

AGAATAACACTTCGAAAATTAAAACATTTTGATCCACTAAGTGACCAACGAACCAAGTTA

CCCTAGGGATAACAGCGCAATCCTTTCTAAGAGTCCATATCGACGAATGGGTTTACGACC

TCGATGTTGGATCAGGACACCCAAATGGTGCAGCCGCTGTTAAGGTTCGTTTGTTCAACG

ATTAAAGTCCTACATGAACCCATATGCACTCTCAATTCTATTATCAAGCCTAGCAGTAGG

AACAATTACAACTCTTTCAAGCTCCCACTGATTCCTCGCATGACTAGGCTTAGAAATTAA

CACATTAACAATCATCCCGCTGATAACAAAAATACACCACCCACGAGCAACAGAATCGGC

CACAAAATACTTTCTAACGCAAGCCACCGCATCAGCCCTAATCTTATTTTCTACAATCAC

AAACGCATGGGCCACAGGAGAATGAACAATTATCACCATAAATGACAACACATCAACACT

TATCTTAACAATTGCCCTAGCCATTAAACTGGGAATCGCCCCATTTCACCTGTGATTACC

AGACGTCATACAAGGGCTAAACCTAGCAACATGTTTAATCCTTACAACATGACAAAAACT

AGCCCCAATATCATTAATAATTTTAACTAGCCATCAATTAAACACAAACCTACTAATTAC

AATAGCCATACTATCAACAGTAATTGGGGGGTGGGGGGGCCTTAACCAAACACAAATACG

AAAAATAATAGCATATTCATCTATTGCCCACCTCGGATGAATAACGCTAATCCTGTCTTT

CTCCCCAAACTTAACAACCCTAAACCTAATAATTTACCTAATACTAACCTCATCAATATT

TTTAATAATAATAACCTTAACCTCAACAAATATAAACAAACTTTCAACATCTTGACTGAA

AACACCACTACTAGCATCATCAATAATAGTTACACTCATAGCCCTGGGAGGCCTACCCCC

AACCTCAGGCTTCTTACCAAAATGACTAATCCTACAAGAAATAGCTAAACAACACCTAGG

AGCCGTATCTACACTAATAGCTATATCTGCCCTACTAAGCCTATTCTTCTACCTACGACT

ATCATATGCAATCTCACTAACCACTGCACCAAACACCTCAAACTCCAACATAGCCTGACG

AGTAGCCCACAACCAAATACAAATTCTCCCAACAATAATTACACTAACTACAATAATATT

ACCAATCACACCAACACTACTAGCAACAGGACAAGGCAGCTTGGCGTACCCAGGTCTGAG

GACCCACGGGACTTTGGAGAGCATAGGTGGGCCTAAAGGCAGTTCAAGAGGAGGGTTGCC

CTCACTAGCAGACACTTTTGAACAAGTCATTGAGGAGATGCTTGAAGATGAGCAGAGCAT

GAGGCCCAGTGAGGAGAACAAGGATGCGGACATGTTTACATCTCGCGTAATGCTGAGCAG

TCAAGTGCCTTTGGAGCCTCCTCTTCTCTTTCTGCTGGAGGAGTACAAAAACTACTTGGA

TGCTGCAAATATGTCCATGAGGGTCCGACGTCACTCTGACCCCGCCCGCCGCGGGGAGCT

GAGTGTGTGTGACAGTATTAGTGAGTGGGTGACGGCATCTGACAAAAAGACTGCCGTGGA

CATGTCGGGGCAGACGGTAACTGTTCTAGAAAAAGTTCCAGTACCCAAAGGCCAACTGAA

GCAATACTTCTACGAGACCAAATGCAACCCTATGGGTTACATGAAGGAGGGCTGCAGAGG

CATTGACAAGAGGTACTGGAACTCCCAGTGCCGAACTACTCAGTCGTATGTGCGCGCGCT

CACCATGGATAACAAGAAGAAAGTGGGCTGGCGGTTTATCAGAATAGACACTTCATGTGT

ATGTACATTGACCATTGTTTGCTTGGGTTGCAGACCGAAGGCTGTTGTTCTACAAATATG

TCTACAAGCGATATAGAGCTGGGAAGCAAAGGGGTATGATTATAGAAACCGAAGGTGACA

GGCCTTCTTCAAAAGCTGACATTGAAATGGATGGAAAGATGGTCAACTCTCATACTGAAA

ATTTCATTGATGGCTCTCTAGTTCTGGAAGTCGATGAGAAAGATCAGGATGATGAAGAAG

CCAGGCGCGATATGGCCAGAATCCTAAAGGAGCTAAAGCAGAAACATCCTGACAAGGAAA

TAGAGCAACTGATTGAGTTAGCGAACTACCAGGTCTTGAGTCAACAGCAAAAAAGTCGAG

CATTCTATCGTATTCAAGCTACTCGCTTGATGACTGGGGCCGGAAATATTCTAAAGAGAC

ATGCAGCAGACCAGGCAAGGAAAGCTGTGAGCATGCAGGAAGTTAACTCTGAAGTGATCG

AAAATGAGCCCGTCAGTAAGATCTATTTCGAGCAAGC

>X75

CAACGGCCGCGGTATCATGACCGTGCAAAGGTAGCGTAATCACTTGTCTTTTAAATAAAG

ACCCGTATGAAAGGCAAAACGAAAGTTCAACTGTCTCTTTAATCCAATCAATGAAATTTA

TCTTTTCGTGCAGAGGCGGAGATAAACATATAAGACGAGAAGACCCTATGGAGCTTTAAA

CACACTATAATATAAGTTTTAGGTTGGGGCGACCACGGAGCAAAAAAATCCTCCGAGATA

AGAATAACACTTCGAAAATTAAAACATTTTGATCCACTAAGTGACCAACGAACCAAGTTA

CCCTAGGGATAACAGCGCAATCCTTTCTAAGAGTCCATATCGACGAATGGGTTTACGACC

TCGATGTTGGATCAGGACACCCAAATGGTGCAGCCGCTGTTAAGGTTCGTTTGTTCAACG

ATTAAAGTCCTACATGAACCCATATGCACTCTCAATTCTATTATCAAGCCTAGCAGTAGG

AACAATTACAACTCTTTCAAGCTCCCACTGATTCCTCGCATGACTAGGCTTAGAAATTAA

CACATTAGCAATCATCCCGCTGATAACAAAAATACACCACCCACGAGCAACAGAATCGGC

CACAAAATACTTCCTGACGCAAGCCACCGCATCAGCCCTAATCTTATTTTCTACAATCAC

AAACGCATGAGCCACAGGAGAATGAACAATTATCACCATAAATGACAACACATCAACACT

TATCTTAACAATTGCCCTAGCCATTAAACTTGGAATCGCCCCATTTCATCTGTGATTACC

AGACGTCATACAAGGACTAAACCTAGCAACATGTTTAATCCTTACAACATGACAAAAACT

AGCCCCAATATCATTAATAATTTTAACTAGCTATCAATTAAACACAAACCTACTAATTAC

AATAGCTATACTATCAACAGTAATTGGGGGGTGGGGGGGCCTTAACCAAACACAAATACG

AAAAGTAATAGCATATTCATCTATTGCCCACCTCGGATGAATAACGCTAATCCTGTCTTT

CTCCCCAAACTTAACAACCCTAAACCTAATAATTTACCTAATACTAACCTCATCAATATT

TTTAATAATAATAACCTTAACCTCAACAAATATAAACAAACTTTCAACATCTTGACTGAA

AACACCACTACTAGCATCATCAATAATAGTTACACTCATAGCCCTGGGAGGCCTACCCCC

AACCTCAGGCTTTTTACCAAAATGACTAATCCTACAAGAAATAACTAAACAACACCTAGG

AGCCGTATCTACACTAATAGCTATATCTGCCCTACTAAGCCTATTCTTCTACCTACGACT

ATCATATGCAATCTCACTAACCACTGCACCAAACACCTCAAACTCCAACATAGCCTGACG

AGTAACCCATAACCAAATACAAGTTCTCCCAACAATAATTACACTAACTACAATAATATT

ACCAATCACACCAACACTACTAGCAACAGGACAAGGCAGCTTGGCGTACCCAGGTCTGAG

GACCCACGGGACTTTGGAGAGCATAGGTGGGCCTAAAGGCAGTTCAAGAGGAGGGTTGCC

CTCACTAGCAGACACTTTTGAACAAGTCATTGAGGAGATGCTTGAAGATGAGCAGAGCAT

GAGGCCCAGTGAGGAGAACAAGGATGCGGACATGTTTACATCTCGCGTAATGCTGAGCAG

TCAAGTGCCTTTGGAGCCTCCTCTTCTCTTTCTGCTGGAGGAGTACAAAAACTACTTGGA

TGCTGCAAATATGTCCATGAGGGTCCGACGTCACTCTGACCCCGCCCGCCGCGGGGAGCT

GAGTGTGTGTGACAGTATTAGTGAGTGGGTGACGGCATCTGACAAAAAGACTGCCGTGGA

CATGTCGGGGCAGACGGTAACTGTTCTAGAAAAAGTTCCAGTACCCAAAGGCCAACTGAA

GCAATACTTCTACGAGACCAAATGCAACCCTATGGGTTACATGAAGGAGGGCTGCAGAGG

CATTGACAAGAGGTACTGGAACTCCCAGTGCCGAACTACTCAGTCGTATGTGCGCGCGCT

CACCATGGATAACAAGAAGAAAGTGGGCTGGCGGTTTATCAGAATAGACACTTCATGTGT

ATGTACATTGACCATTGTTTGCTTGGGTTGCAGACCGAAGGCTGTTGTTCTACAAATATG

TCTACAAGCGATATAGAGCTGGGAAGCAAAGGGGTATGATTATAGAAACCGAAGGTGACA

GGCCTTCTTCAAAAGCTGACATTGAAATGGATGGAAAGATGGTCAACTCTCATACTGAAA

ATTTCATTGATGGCTCTCTAGTTCTGGAAGTCGATGAGAAAGATCAGGATGATGAAGAAG

CCAGGCGCGATATGGCCAGAATCCTAAAGGAGCTAAAGCAGAAACATCCTGACAAGGAAA

TAGAGCAACTGATTGAGTTAGCGAACTACCAGGTCTTGAGTCAACAGCAAAAAAGTCGAG

CATTCTATCGTATTCAAGCTACTCGCTTGATGACTGGGGCCGGAAATATTCTAAAGAGAC

ATGCAGCAGACCAGGCAAGGAAAGCTGTGAGCATGCAGGAAGTTAACTCTGAAGTGATCG

AAAATGAGCCCGTCAGTAAGATCTATTTCAAGCAAGC

>X78

CAACGGCCGCGGTATCATGACCGTGCAAAGGTAGCGTAATCACTTGTCTTTTAAATAAAG

ACCCGTATGAAAGGCAAAACGAAAGTTCGACTGTCTCTTTAATCCAATCAATGAAATTTA

TCTTTTCGTGCAGAGGCGGAGATAAATATATAAGACGAGAAGACCCTATGGAGCTTTAAA

CACACTATAATATAAGTTTTGGGTTGGGGCGACCACGGAGCAAAAAAATCCTCCGAGATA

AGAATAACACTTCGAAAATTAAAACATTTTGATCCACTAAGTGACCAACGAACCAAGTTA

CCCTAGGGATAACAGCGCAATCCTTTCTAAGAGTCCATATCGACGAATGGGTTTACGACC

TCGATGTTGGATCAGGACACCCAAATGGTGCAGCCGCTGTTAAGGTTCGTTTGTTCAACG

ATTAAAGTCCTACATGAACCCATATGCACTCTCAATTCTATTATCAAGCCTGGCAGTAGG

AACAATTACAACTCTTTCAAGCTCCCACTGATTTCTCGCATGACTAGGCTTAGAAATAAA

TACACTAGCGATCATCCCCTTAATAACAAAAATACATCACCCACGAGCAACAGAATCAGC

CACAAAATACTTCCTGACGCAAGCCGCCGCATCAGCCCTAATCTTATTTTCTACAATCAC

AAACGCATGGGCCACAGGAGAATGAACAATTATCACCATAAATGACAACACATCAACACT

TATCTTAACAATCGCCCTAGCCATTAAACTGGGAATCGCCCCATTTCACCTGTGATTACC

AGACGTCATACAAGGACTAAACCTAGCAACATGTTTAATCCTTACAACATGACAAAAACT

AGCCCCAATATCATTAATAATCATAACTAGCCATCAATTAAACGCAAACCTACTAATTAC

AATAGCCATACTATCAACAGTAATTGGGGGGTGGGGGGGCCTTAACCAAACACAAATCCG

AAAAATAATAGCATATTCATCTATCGCCCACCTCGGATGAATAACACTAATCCTGTCTTT

CTCCCCAAACTTAACGACCCTAAACTTAATAATTTACCTAATACTAACCTCATCAATATT

TTTAATAATAATAACCCTAACCTCAACAAATATAAACAAACTTTCAACATCTTGACTGAA

AACGCCACTACTAGCATCATCAATAATAATTACACTCATAGCCCTGGGAGGCCTACCCCC

AACCTCAGGCTTCTTACCAAAATGACTGATCCTACAAGAAATAACTAAACAACACCTAGG

AGCCGTGTCTACACTAATAGCTATATCTGCCCTACTAAGCCTATTCTTCTATTTACGACT

ATCATATGCAATCTCACTAACCACTCCGCCAAACACCTCAAACTCCAACATAACCTGACG

AGTAGCCCACAACCAAATACAAGTCCTCCCAACAATAATCACACTAACTACAATAATACT

ACCAATCACACCAGCACTATTAGCAACAGGACAAGGCAGCTTGGCGTACCCAGGTCTGAG

GACCCACGGGACTTTGGAGAGCATAGGTGGGCCTAAAGGCAGTTCAAGAGGAGGGTTGCC

CTCACTAGCAGACACTTTTGAACAAGTCATTGAGGAGATGCTTGAAGATGAGCAGAGCAT

GAGGCCCAGTGAGGAGAACAAGGATGCGGACATGTTTACATCTCGCGTAATGCTGAGCAG

TCAAGTGCCTTTGGAGCCTCCTCTTCTCTTTCTGCTGGAGGAGTACAAAAACTACTTGGA

TGCTGCAAATATGTCCATGAGGGTCCGACGTCACTCTGACCCCGCCCGCCGCGGGGAGCT

GAGTGTGTGTGACAGTATTAGTGAGTGGGTGACGGCATCTGACAAAAAGACTGCCGTGGA

CATGTCGGGGCAGACGGTAACTGTTCTAGAAAAAGTTCCAGTACCCAAAGGCCAACTGAA

GCAATACTTCTACGAGACCAAATGCAACCCTATGGGTTACATGAAGGAGGGCTGCAGAGG

CATTGACAAGAGGTACTGGAACTCCCAGTGCCGAACTACTCAGTCGTATGTGCGCGCGCT

CACCATGGATAACAAGAAGAAAGTGGGCTGGCGGTTTATCAGAATAGACACTTCATGTGT

ATGTACATTGACCATTGTTTGCTTGGGTTGCAGACCGAAGGCTGTTGTTCTACAAATATG

TCTACAAGCGATATAGAGCTGGGAAGCAAAGGGGTATGATTATAGAAACCGAAGGTGACA

GGCCTTCTTCAAAAGCTGACATTGAAATGGATGGAAAGATGGTCAACTCTCATACTGAAA

ATTTCATTGATGGCTCTCTAGTTCTGGAAGTCGATGAGAAAGATCAGGATGATGAAGAAG

CCAGGCGCGATATGGCCAGAATCCTAAAGGAGCTAAAGCAGAAACATCCTGACAAGGAAA

TAGAGCAACTGATTGAGTTAGCGAACTACCAGGTCTTGAGTCAACAGCAAAAAAGTCGAG

CATTCTATCGTATTCAAGCTACTCGCTTGATGACTGGGGCCGGAAATATTCTAAAGAGAC

ATGCAGCAGACCAGGCAAGGAAAGCTGTGAGCATGCAGGAAGTTAACTCTGAAGTGATCG

AAAATGAGCCCGTCAGTAAGATCTATTTCGAGCAAGC

>X79

CAACGGCCGCGGTATCATGACCGTGCAAAGGTAGCGTAATCACTTGTCTTTTAAATAAAG

ACCCGTATGAAAGGCAAAACGAAAGTTCGACTGTCTCTTTAATCCAATCAATGAAATTTA

TCTTTTCGTGCAGAGGCGGAGATAAATATATAAGACGAGAAGACCCTATGGAGCTTTAAA

CACACTATAATATAAGTTTTGGGTTGGGGCGACCACGGAGCAAAAAAATCCTCCGAGATA

AGAATAACACTTCGAAAATTAAAACATTTTGATCCACTAAGTGACCAACGAACCAAGTTA

CCCTAGGGATAACAGCGCAATCCTTTCTAAGAGTCCATATCGACGAATGGGTTTACGACC

TCGATGTTGGATCAGGACACCCAAATGGTGCAGCCGCTGTTAAGGTTCGTTTGTTCAACG

ATTAAAGTCCTACATGAACCCATATGCACTCTCAATTCTATTATCAAGCCTGGCAGTAGG

AACAATTACAACTCTTTCAAGCTCCCACTGATTTCTCGCATGACTAGGCTTAGAAATAAA

TACACTAGCGATCATCCCCTTAATAACAAAAATACATCACCCACGAGCAACAGAATCAGC

CACAAAATACTTCCTGACGCAAGCCGCCGCATCAGCCCTAATCTTATTTTCTACAATCAC

AAACGCATGGGCCACAGGAGAATGAACAATTATCACCATAAATGACAACACATCAACACT

TATCTTAACAATCGCCCTAGCCATTAAACTGGGAATCGCCCCATTTCACCTGTGATTACC

AGACGTCATACAAGGACTAAACCTATCAACATGTTTAATCCTTACAACATGACAAAAACT

AGCCCCAATATCATTAATAATCATAACTAGCCATCAATTAAACGCAAACCTACTAATTAC

AATAGCCATACTATCAACAGTAATTGGGGGGTGGGGGGGCCTTAACCAAACACAAATCCG

AAAAATAATAGCATATTCATCTATCGCCCACCTCGGATGAATAACACTAATCCTGTCTTT

CTCCCCAAACTTAACGACCCTAAACTTAATAATTTACCTAATACTAACCTCATCAATATT

TTTAATAATAATAACCCTAACCTCAACAAATATAAACAAACTTTCAACATCTTGACTGAA

AACGCCACTACTAGCATCATCAATAATAATTACACTCATAGCCCTGGGAGGCCTACCCCC

AACCTCAGGCTTCTTACCAAAATGACTGATCCTACAAGAAATAACTAAACAACACCTAGG

AGCCGTGTCTACACTAATAGCTATATCTGCCCTACTAAGCCTATTCTTCTATTTACGACT

ATCATATGCAATCTCACTAACCACTCCGCCAAACACCTCAAACTCCAACATAACCTGACG

AGTAGCCCACAACCAAATACAAGTCCTCCCAACAATAATCACACTAACTACAATAATACT

ACCAATCACACCAGCACTATTAGCAACAGGACAAGGCAGCTTGGCGTACCCAGGTCTGAG

GACCCACGGGACTTTGGAGAGCATAGGTGGGCCTAAAGGCAGTTCAAGAGGAGGGTTGCC

CTCACTAGCAGACACTTTTGAACAAGTCATTGAGGAGATGCTTGAAGATGAGCAGAGCAT

GAGGCCCAGTGAGGAGAACAAGGATGCGGACATGTTTACATCTCGCGTAATGCTGAGCAG

TCAAGTGCCTTTGGAGCCTCCTCTTCTCTTTCTGCTGGAGGAGTACAAAAACTACTTGGA

TGCTGCAAATATGTCCATGAGGGTCCGACGTCACTCTGACCCCGCCCGCCGCGGGGAGCT

GAGTGTGTGTGACAGTATTAGTGAGTGGGTGACGGCATCTGACAAAAAGACTGCCGTGGA

CATGTCGGGGCAGACGGTAACTGTTCTAGAAAAAGTTCCAGTACCCAAAGGCCAACTGAA

GCAATACTTCTACGAGACCAAATGCAACCCTATGGGTTACATGAAGGAGGGCTGCAGAGG

CATTGACAAGAGGTACTGGAACTCCCAGTGCCGAACTACTCAGTCGTATGTGCGCGCGCT

CACCATGGATAACAAGAAGAAAGTGGGCTGGCGGTTTATCAGAATAGACACTTCATGTGT

ATGTACATTGACCATTGTTTGCTTGGGTTGCAGACCGAAGGCTGTTGTTCTACAAATATG

TCTACAAGCGATATAGAGCTGGGAAGCAAAGGGGTATGATTATAGAAACCGAAGGTGACA

GGCCTTCTTCAAAAGCTGACATTGAAATGGATGGAAAGATGGTCAACTCTCATACTGAAA

ATTTCATTGATGGCTCTCTAGTTCTGGAAGTCGATGAGAAAGATCAGGATGATGAAGAAG

CCAGGCGCGATATGGCCAGAATCCTAAAGGAGCTAAAGCAGAAACATCCTGACAAGGAAA

TAGAGCAACTGATTGAGTTAGCGAACTACCAGGTCTTGAGTCAACAGCAAAAAAGTCGAG

CATTCTATCGTATTCAAGCTACTCGCTTGATGACTGGGGCCGGAAATATTCTAAAGAGAC

ATGCAGCAGACCAGGCAAGGAAAGCTGTGAGCATGCAGGAAGTTAACTCTGAAGTGATCG

AAAATGAGCCCGTCAGTAAGATCTATTTCGAGCAAGC

>X80

CAACGGCCGCGGTATCATGACCGTGCAAAGGTAGCGTAATCACTTGTCTTTTAAATAAAG

ACCCGTATGAAAGGCAAAACGAAAGTTCAACTGTCTCTTTAATCCAATCAATGAAATTTA

TCTTTTCGTGCAGAGGCGGAAATAAATATATAAGACGAGAAGACCCTATGGAGCTTTAAA

CACACTATAATACAAGTTTTGGGTTGGGGCGACCACGGAGCAAAAAAATCCTCCGAGATA

AGAATAACACTTCAAAAATTAAAACATTTTGATCCACTAAGTGACCAACGAACCAAGTTA

CCCTAGGGATAACAGCGCAATCCTTTCTAAGAGTCCATATCGACGAATGGGTTTACGACC

TCGATGTTGGATCAGGACACCCAAATGGTGCAGCCGCTGTTAAGGTTCGTTTGTTCAACG

ATTAAAGTCCTACATGAACCCATATGCACTATCAATTCTATTATCAAGCCTGGCAGTAGG

GACAATTACAACTCTTTCAAGCTCCCACTGATTCCTCGCATGACTAGGCTTAGAAATTAA

TACATTAGCAATCATCCCCCTGATAACAAAAATACACCACCCACGAGCGACAGAATCAGC

CACAAAATACTTCCTGACGCAAGCTACCGCATCGGCCCTAATCTTATTTTCTACAATCAC

AAACGCATGGGCCACAGGAGAATGAACAATTATTACCATAAATGACAACACATCAACACT

TATCTTAACAATCGCCCTAGCCATTAAACTAGGAATCGCCCCATTTCACCTGTGATTACC

AGACGTCATACAAGGACTAAACCTAGCAACATGTTTAATCCTTACAACATGACAAAAACT

GGCCCCAATATCACTAATAATCATAACTAGCCATCAATTAAACACAAACCTACTAGTTAC

AATAGCCATACTATCAACGGTAATTGGGGGGTGGGGGGGTCTTAACCAAACACAAATACG

AAAAATAATAGCATATTCATCTATTGCCCACCTCGGATGAATAACACTAATCCTGTCTTT

CTCCCCAAACTTAACAACCCTAAACTTAATAATTTACCTAATACTAACCTCATCAATATT

TTTAATAATAATAACCCTAACCTCAACAAATATAAACAAACTTTCAACATCTTGACTGAA

AACACCACTTCTAGCATCATCAATAATAATTACACTCATAGCCCTAGGAGGCCTACCCCC

AACCTCAGGCTTCTTACCAAAATGACTGATCCTACAAGAAATAACTAAACAACACCTAGG

AGCCGTATCTACACTAATAGCCATATCTGCCCTACTAAGCCTATTCTTCTATTTACGACT

ATCATATGCGATCTCACTAACCACTGCACCAAACACCTCAAACTCCAACATAACCTGACG

AGTGGCCCACAACCAAATACAAGTCCTCCCAACAATAATTACACTGACTACAGTAATACT

ACCAATCACACCAACACTACTAGCAACAGGACAAGGCAGCTTGGCGTACCCAGGTCTGAG

GACCCACGGGACTTTGGAGAGCATAGGTGGGCCTAAAGGCAGTTCAAGAGGAGGGTTGCC

CTCACTAGCAGACACTTTTGAACAAGTCATTGAGGAGATGCTTGAAGATGAGCAGAGCAT

GAGGCCCAGTGAGGAGAACAAGGATGCGGACATGTTTACATCTCGCGTAATGCTGAGCAG

TCAAGTGCCTTTGGAGCCTCCTCTTCTCTTTCTGCTGGAGGAGTACAAAAACTACTTGGA

TGCTGCAAATATGTCCATGAGGGTCCGACGTCACTCTGACCCCGCCCGCCGCGGGGAGCT

GAGTGTGTGTGACAGTATTAGTGAGTGGGTGACGGCATCTGACAAAAAGACTGCCGTGGA

CATGTCGGGGCAGACGGTAACTGTTCTAGAAAAAGTTCCAGTACCCAAAGGCCAACTGAA

GCAATACTTCTACGAGACCAAATGCAACCCTATGGGTTACATGAAGGAGGGCTGCAGAGG

CATTGACAAGAGGTACTGGAACTCCCAGTGCCGAACTACTCAGTCGTATGTGCGCGCGCT

CACCATGGATAACAAGAAGAAAGTGGGCTGGCGGTTTATCAGAATAGACACTTCATGTGT

ATGTACATTGACCATTGTTTGCTTGGGTTGCAGACCGAAGGCTGTTGTTCTACAAATATG

TCTACAAGCGATATAGAGCTGGGAAGCAAAGGGGTATGATTATAGAAACCGAAGGTGACA

GGCCTTCTTCAAAAGCTGACATTGAAATGGATGGAAAGATGGTCAACTCTCATACTGAAA

ATTTCATTGATGGCTCTCTAGTTCTGGAAGTCGATGAGAAAGATCAGGATGATGAAGAAG

CCAGGCGCGATATGGCCAGAATCCTAAAGGAGCTAAAGCAGAAACATCCTGACAAGGAAA

TAGAGCAACTGATTGAGTTAGCGAACTACCAGGTCTTGAGTCAACAGCAAAAAAGTCGAG

CATTCTATCGTATTCAAGCTACTCGCTTGATGACTGGGGCCGGAAATATTCTAAAGAGAC

ATGCAGCAGACCAGGCAAGGAAAGCTGTGAGCATGCAGGAAGTTAACTCTGAAGTGATCG

AAAATGAGCCCGTCAGTAAGATCTATTTCGAGCAAGC

>X81

CAACGGCCGCGGTATCATGACCGTGCAAAGGTAGCGTAATCACTTGTCTTTTAAATAAAG

ACCCGTATGAAAGGCAAAACGAAAGTTCAACTGTCTCTTTAATCCAATCAATGAAATTTA

TCTTTTCGTGCAGAGGCGGAAATAAATATATAAGACGAGAAGACCCTATGGAGCTTTAAA

CACACTATAATACAAGTTTTGGGTTGGGGCGACCACGGAGCAAAAAAATCCTCCGAGATA

AGAATAACACTTCAAAAATTAAAACATTTTGATCCACTAAGTGACCAACGAACCAAGTTA

CCCTAGGGATAACAGCGCAATCCTTTCTAAGAGTCCATATCGACGAATGGGTTTACGACC

TCGATGTTGGATCAGGACACCCAAATGGTGCAGCCGCTGTTAAGGTTCGTTTGTTCAACG

ATTAAAGTCCTACATGAACCCATATGCACTATCAATTCTATTATCAAGCCTGGCAGTAGG

GACAATTACAACTCTTTCAAGCTCCCACTGATTCCTCGCATGACTAGGCTTAGAAATTAA

TACATTAACAATCATCCCCCTGATAACAAAAATACACCACCCACGAGCGACAGAATCAGC

CACAAAATACTTCCTGACGCAAGCTACCGCATCGGCCCTAATCTTATTTTCTACAATCAC

AAACGCATGGGCCACAGGAGAATGAACAATTATTACCATAAATGACAACACATCAACACT

TATCTTAACAATCGCCCTAGCCATTAAACTAGGAATCGCCCCATTTCACCTGTGATTACC

AGACGTCATACAAGGACTAAACCTAGCAACATGTTTAATCCTTACAACATGACAAAAACT

GGCCCCAATATCACTAATAATCATAACTAGCCATCAATTAAACACAAACCTACTAGTTAC

AATAGCCATACTATCAACGGTAATTGGGGGGTGGGGGGGTCTTAACCAAACACAAATACG

AAAAATAATAGCATATTCATCTATTGCCCACCTCGGATGAATAACACTAATCCTGTCTTT

CTCCCCAAACTTAACAACCCTAAACTTAATAATTTACCTAATACTAACCTCATCAATATT

TTTAATAATAATAACCCTAACCTCAACAAATATAAACAAACTTTCAACATCTTGACTGAA

AACACCACTTCTAGCATCATCAATAATAATTACACTCATAGCCCTAGGAGGCCTACCCCC

AACCTCAGGCTTCTTACCAAAATGACTGATCCTACAAGAAATAACTAAACAACACCTAGG

AGCCGTATCTACACTAATAGCCATATCTGCCCTACTAAGCCTATTCTTCTATTTACGACT

ATCATATGCGATCTCACTAACCACTGCACCAAACACCTCAAACTCCAACATAACCTGACG

AGTGGCCCACAACCAAATACAAGTCCTCCCAACAATAATTACACTGACTACAGTAATACT

ACCAATCACACCAACACTACTAGCAACAGGACAAGGCAGCTTGGCGTACCCAGGTCTGAG

GACCCACGGGACTTTGGAGAGCATAGGTGGGCCTAAAGGCAGTTCAAGAGGAGGGTTGCC

CTCACTAGCAGACACTTTTGAACAAGTCATTGAGGAGATGCTTGAAGATGAGCAGAGCAT

GAGGCCCAGTGAGGAGAACAAGGATGCGGACATGTTTACATCTCGCGTAATGCTGAGCAG

TCAAGTGCCTTTGGAGCCTCCTCTTCTCTTTCTGCTGGAGGAGTACAAAAACTACTTGGA

TGCTGCAAATATGTCCATGAGGGTCCGACGTCACTCTGACCCCGCCCGCCGCGGGGAGCT

GAGTGTGTGTGACAGTATTAGTGAGTGGGTGACGGCATCTGACAAAAAGACTGCCGTGGA

CATGTCGGGGCAGACGGTAACTGTTCTAGAAAAAGTTCCAGTACCCAAAGGCCAACTGAA

GCAATACTTCTACGAGACCAAATGCAACCCTATGGGTTACATGAAGGAGGGCTGCAGAGG

CATTGACAAGAGGTACTGGAACTCCCAGTGCCGAACTACTCAGTCGTATGTGCGCGCGCT

CACCATGGATAACAAGAAGAAAGTGGGCTGGCGGTTTATCAGAATAGACACTTCATGTGT

ATGTACATTGACCATTGTTTGCTTGGGTTGCAGACCGAAGGCTGTTGTTCTACAAATATG

TCTACAAGCGATATAGAGCTGGGAAGCAAAGGGGTATGATTATAGAAACCGAAGGTGACA

GGCCTTCTTCAAAAGCTGACATTGAAATGGATGGAAAGATGGTCAACTCTCATACTGAAA

ATTTCATTGATGGCTCTCTAGTTCTGGAAGTCGATGAGAAAGATCAGGATGATGAAGAAG

CCAGGCGCGATATGGCCAGAATCCTAAAGGAGCTAAAGCAGAAACATCCTGACAAGGAAA

TAGAGCAACTGATTGAGTTAGCGAACTACCAGGTCTTGAGTCAACAGCAAAAAAGTCGAG

CATTCTATCGTATTCAAGCTACTCGCTTGATGACTGGGGCCGGAAATATTCTAAAGAGAC

ATGCAGCAGACCAGGCAAGGAAAGCTGTGAGCATGCAGGAAGTTAACTCTGAAGTGATCG

AAAATGAGCCCGTCAGTAAGATCTATTTCGAGCAAGC

>X82

CAACGGCCGCGGTATCATGACCGTGCAAAGGTAGCGTAATCACTTGTCTTTTAAATAAAG

ACCCGTATGAAAGGCAAAACGAAAGTTCAACTGTCTCTTTAATCCAATCAATGAAATTTA

TCTTTTCGTGCAGAGGCGGAGATAAATGTATAAGACGAGAAGACCCTATGGAGCTTTAAA

CACACTATAATATAAGTTTTGGGTTGGGGCGACCACGGAGCAAAAAAATCCTCCGAGATA

AGAATAACACTTCGAAAATTAAAACATTTTGATCCACTAAGTGACCAACGAACCAAGTTA

CCCTAGGGATAACAGCGCAATCCTTTCTAAGAGTCCATATCGACGAATGGGTTTACGACC

TCGATGTTGGATCAGGACACCCAAATGGTGCAGCCGCTGTTAAGGTTCGTTTGTTCAACG

ATTAAAGTCCTACATGAACCCATATGCACTCTCAATCCTATTATCAAGCCTAGCAGTAGG

AACAATTACAACCCTTTCAAGCTTCCACTGATTCCTCGCATGACTAGGCTTAGAAATTAA

CACATTAGCAATCATCCCCCTGATAACAAAAATACACCACCCACGAGCAACAGAATCGGC

CACAAAATACTTCCTGACGCAAGCCACCGCATCAGCCCTAATCTTATTTTCTACAATCAC

AAACGCATGGGCCACAGGAGAATGAACAATTATCACCATAAATGACAACACATCAACACT

TATCTTAACAATCGCCCTAGCCATTAAGCTTGGAATCGCCCCATTTCACCTGTGATTACC

AGACGTCATACAAGGGCTAAACCTAGCAACATGTTTAATCCTTACAACATGACAAAAACT

AGCCCCAATATCATTAATAATTATAACTAGCCATCAATTAAACACAAACCTACTAATTAC

AATAGCCATATTATCAACAGTAATTGGGGGGTGGGGGGGCCTTAACCAAACACAAATACG

AAAAATAATAGCATATTCATCTATTGCCCACCTCGGATGAATAACACTAATCTTGTCTTT

CTCCCCAAACTTAACAACCCTGAACCTAATAATTTACCTAATACTAACCTCATCGATATT

TTTAATAATAATAACCTTGACCTCGACAAATATAAACAAACTTTCAACATCTTGACTGAA

AACGCCACTACTAGCATCATCAATAATAGTTACACTCATAGCCCTGGGAGGCCTACCCCC

AACCTCAGGCTTCTTACCAAAATGACTAATCCTACAAGAAATAACTAAACAACACCTAGG

AGCCGTATCTACACTAATAACTATATCTGCCCTACTAAGCCTATTCTTCTACCTACGACT

ATCATATGCAATCTCACTAACCACTGCGCCAAACACCTCAAACTCCAACATAACCTGACG

AGTAGCCCACAGCCAAATACAAGTCCTCCCAACAATAATTACACTAACTACAATAATACT

ACCAATCACACCAACACTACTAGCAACAGGACAAGGCAGCTTGGCGTACCCAGGTCTGAG

GACCCACGGGACTTTGGAGAGCATAGGTGGGCCTAAAGGCAGTTCAAGAGGAGGGTTGCC

CTCACTAGCAGACACTTTTGAACAAGTCATTGAGGAGATGCTTGAAGATGAGCAGAGCAT

GAGGCCCAGTGAGGAGAACAAGGATGCGGACATGTTTACATCTCGCGTAATGCTGAGCAG

TCAAGTGCCTTTGGAGCCTCCTCTTCTCTTTCTGCTGGAGGAGTACAAAAACTACTTGGA

TGCTGCAAATATGTCCATGAGGGTCCGACGTCACTCTGACCCCGCCCGCCGCGGGGAGCT

GAGTGTGTGTGACAGTATTAGTGAGTGGGTGACGGCATCTGACAAAAAGACTGCCGTGGA

CATGTCGGGGCAGACGGTAACTGTTCTAGAAAAAGTTCCAGTACCCAAAGGCCAACTGAA

GCAATACTTCTACGAGACCAAATGCAACCCTATGGGTTACATGAAGGAGGGCTGCAGAGG

CATTGACAAGAGGTACTGGAACTCCCAGTGCCGAACTACTCAGTCGTATGTGCGCGCGCT

CACCATGGATAACAAGAAGAAAGTGGGCTGGCGGTTTATCAGAATAGACACTTCATGTGT

ATGTACATTGACCATTGTTTGCTTGGGTTGCAGACCGAAGGCTGTTGTTCTACAAATATG

TCTACAAGCGATATAGAGCTGGGAAGCAAAGGGGTATGATTATAGAAACCGAAGGTGACA

GGCCTTCTTCAAAAGCTGACATTGAAATGGATGGAAAGATGGTCAACTCTCATACTGAAA

ATTTCATTGATGGCTCTCTAGTTCTGGAAGTCGATGAGAAAGATCAGGATGATGAAGAAG

CCAGGCGCGATATGGCCAGAATCCTAAAGGAGCTAAAGCAGAAACATCCTGACAAGGAAA

TAGAGCAACTGATTGAGTTAGCGAACTACCAGGTCTTGAGTCAACAGCAAAAAAGTCGAG

CATTCTATCGTATTCAAGCTACTCGCTTGATGACTGGGGCCGGAAATATTCTAAAGAGAC

ATGCAGCAGACCAGGCAAGGAAAGCTGTGAGCATGCAGGAAGTTAACTCTGAAGTGATCG

AAAATGAGCCCGTCAGTAAGATCTATTTCGAGCAAGC

>X83

CAACGGCCGCGGTATCATGACCGTGCAAAGGTAGCGTAATCACTTGTCTTTTAAATAAAG

ACCCGTATGAAAGGCAAAACGAAAGTTCAACTGTCTCTTTAATCCAATCAATGAAATTTA

TCTTTTCGTGCAGAGGCGGAGATAAATATATAAGACGAGAAGACCCTATGGAGCTTTAAA

CACACTATAATATAAGTTTTGGGTTGGGGCGACCACGGAGCAAAAAAATCCTCCGAGATA

AGAATAACACTTCGAAAATTAAAACATTTTGATCCACTAAGTGACCAACGAACCAAGTTA

CCCTAGGGATAACAGCGCAATCCTTTCTAAGAGTCCATATCGACGAATGGGTTTACGACC

TCGATGTTGGATCAGGACACCCAAATGGTGCAGCCGCTGTTAAGGTTCGTTTGTTCAACG

ATTAAAGTCCTACATGAACCCATATGCACTCTCAATCCTATTATCAAGCCTAGCAGTAGG

AACAATTACAACCCTTTCAAGCTTCCACTGATTCCTCGCATGACTAGGCTTAGAAATTAA

CACATTAGCAATCATCCCCCTGATAACAAAAATACACCACCCACGAGCAACAGAATCGGC

CACAAAATACTTCCTGACGCAAGCCACCGCATCAGCCCTAATCTTATTTTCTACAATCAC

AAACGCATGGGCCACAGGAGAATGAACAATTATCACCATAAATGACAACACATCAACACT

TATCTTAACAGTCGCCCTAGCCATTAAGCTTGGAATCGCCCCATTTCACCTGTGATTACC

AGACGTCATACAAGGGCTAAACCTAGCAACATGTTTAATCCTTACAACATGACAAAAACT

AGCCCCAATATCATTAATAATTATAACTAGCCATCAATTAAACACAAACCTACTAATTAC

AATAGCCATATTATCAACAGTAATTGGGGGGTGGGGGGGCCTTAACCAAACACAAATACG

AAAAATAATAGCATATTCATCTATTGCCCACCTCGGATGAATAACACTAATCTTGTCTTT

CTCCCCAAACTTAACAACCCTGAACCTAATAATTTACCTAATACTAACCTCATCGATATT

TTTAATAATAATAACCTTGACCTCGACAAATATAAACAAACTTTCAACATCTTGACTGAA

AACGCCACTACTAGCATCATCAATAATAGTTACACTCATAGCCCTGGGAGGCCTACCCCC

AACCTCAGGCTTCTTACCAAAATGACTAATCCTACAAGAAATAACTAAACAACACCTAGG

AGCCGTATCTACACTAATAGCTATATCTGCCCTACTAAGCCTATTCTTCTACCTACGACT

ATCATATGCAATCTCACTAACCACTGCGCCAAACACCTCAAACTCCAACATAACCTGACG

AGTAGCCCACAGCCAAATACAAGTCCTCCCAACAATAATTACACTAACTACAATAATACT

ACCAATCACACCAACACTACTAGCAACAGGACAAGGCAGCTTGGCGTACCCAGGTCTGAG

GACCCACGGGACTTTGGAGAGCATAGGTGGGCCTAAAGGCAGTTCAAGAGGAGGGTTGCC

CTCACTAGCAGACACTTTTGAACAAGTCATTGAGGAGATGCTTGAAGATGAGCAGAGCAT

GAGGCCCAGTGAGGAGAACAAGGATGCGGACATGTTTACATCTCGCGTAATGCTGAGCAG

TCAAGTGCCTTTGGAGCCTCCTCTTCTCTTTCTGCTGGAGGAGTACAAAAACTACTTGGA

TGCTGCAAATATGTCCATGAGGGTCCGACGTCACTCTGACCCCGCCCGCCGCGGGGAGCT

GAGTGTGTGTGACAGTATTAGTGAGTGGGTGACGGCATCTGACAAAAAGACTGCCGTGGA

CATGTCGGGGCAGACGGTAACTGTTCTAGAAAAAGTTCCAGTACCCAAAGGCCAACTGAA

GCAATACTTCTACGAGACCAAATGCAACCCTATGGGTTACATGAAGGAGGGCTGCAGAGG

CATTGACAAGAGGTACTGGAACTCCCAGTGCCGAACTACTCAGTCGTATGTGCGCGCGCT

CACCATGGATAACAAGAAGAAAGTGGGCTGGCGGTTTATCAGAATAGACACTTCATGTGT

ATGTACATTGACCATTGTTTGCTTGGGTTGCAGACCGAAGGCTGTTGTTCTACAAATATG

TCTACAAGCGATATAGAGCTGGGAAGCAAAGGGGTATGATTATAGAAACCGAAGGTGACA

GGCCTTCTTCAAAAGCTGACATTGAAATGGATGGAAAGATGGTCAACTCTCATACTGAAA

ATTTCATTGATGGCTCTCTAGTTCTGGAAGTCGATGAGAAAGATCAGGATGATGAAGAAG

CCAGGCGCGATATGGCCAGAATCCTAAAGGAGCTAAAGCAGAAACATCCTGACAAGGAAA

TAGAGCAACTGATTGAGTTAGCGAACTACCAGGTCTTGAGTCAACAGCAAAAAAGTCGAG

CATTCTATCGTATTCAAGCTACTCGCTTGATGACTGGGGCCGGAAATATTCTAAAGAGAC

ATGCAGCAGACCAGGCAAGGAAAGCTGTGAGCATGCAGGAAGTTAACTCTGAAGTGATCG

AAAATGAGCCCGTCAGTAAGATCTATTTCGAGCAAGC

>X84

CAACGGCCGCGGTATCATGACCGTGCAAAGGTAGCGTAATCACTTGTCTTTTAAATAAAG

ACCCGTATGAAAGGCAAAACGAAAGTTCAACTGTCTCTTTAATCCAATCAATGAAATTTA

TCTTTTCGTGCAGAGGCGGAGATAAATATATAAGACGAGAAGACCCTATGGAGCTTTAAA

CACACTATAATATAAGTTTTGGGTTGGGGCGACCACGGAGCAAAAAAATCCTCCGAGATA

AGAATAACACTTCGAAAATTAAAACATTTTGATCCACTAAGTGACCAACGAACCAAGTTA

CCCTAGGGATAACAGCGCAATCCTTTCTAAGAGTCCATATCGACGAATGGGTTTACGACC

TCGATGTTGGATCAGGACACCCAAATGGTGCAGCCGCTGTTAAGGTTCGTTTGTTCAACG

ATTAAAGTCCTACATGAACCCATATGCACTCTCAATCCTATTATCAAGCCTAGCAGTAGG

AACAATTACAACCCTTTCAAGCTTCCACTGATTCCTCGCATGACTAGGCTTAGAAATTAA

CACATTAGCAATCATCCCCCTGATAACAAAAATACACCACCCACGAGCAACAGAATCGGC

CACAAAATACTTCCTGACGCAAGCCACCGCATCAGCCCTAATCTTATTTTCTACAATCAC

AAACGCATGGGCCACAGGAGAATGAACAATTATCACCATAAATGACAACACATCAACACT

TATCTTAACAATCGCCCTAGCCATTAAGCTTGGAATCGCCCCATTTCACCTGTGATTACC

AGACGTCATACAAGGGCTAAACCTAGCAACATGTTTAATCCTTACAACATGACAAAAACT

AGCCCCAATATCATTAATAATTATAACTAGCCATCAATTAAACACAAACCTACTAATTAC

AATAGCCATATTATCAACAGTAATTGGGGGGTGGGGGGGCCTTAACCAAACACAAATACG

AAAAATAATAGCATATTCATCTATTGCCCACCTCGGATGAATAACACTAATCTTGTCTTT

CTCCCCAAACTTAACAACCCTGAACCTAATAATTTACCTAATACTAACCTCATCGATATT

TTTAATAATAATAACCTTGACCTCGACAAATATAAACAAACTTTCAACATCTTGACTGAA

AACGCCACTACTAGCATCATCAATAATAGTTACACTCATAGCCCTGGGAGGCCTACCCCC

AACCTCAGGCTTCTTACCAAAATGACTAATCCTACAAGAAATAACTAAACAACACCTAGG

AGCCGTATCTACACTAATAGCTATATCTGCCCTACTAAGCCTATTCTTCTACCTACGACT

ATCATATGCAATCTCACTAACCACTGCGCCAAACACCTCAAACTCCAACATAACCTGACG

AGTAGCCCACAGCCAAATACAAGTCCTCCCAACAATAATTACACTAACTACAATAATACT

ACCAATCACACCAACACTACTAGCAACAGGACAAGGCAGCTTGGCGTACCCAGGTCTGAG

GACCCACGGGACTTTGGAGAGCATAGGTGGGCCTAAAGGCAGTTCAAGAGGAGGGTTGCC

CTCACTAGCAGACACTTTTGAACAAGTCATTGAGGAGATGCTTGAAGATGAGCAGAGCAT

GAGGCCCAGTGAGGAGAACAAGGATGCGGACATGTTTACATCTCGCGTAATGCTGAGCAG

TCAAGTGCCTTTGGAGCCTCCTCTTCTCTTTCTGCTGGAGGAGTACAAAAACTACTTGGA

TGCTGCAAATATGTCCATGAGGGTCCGACGTCACTCTGACCCCGCCCGCCGCGGGGAGCT

GAGTGTGTGTGACAGTATTAGTGAGTGGGTGACGGCATCTGACAAAAAGACTGCCGTGGA

CATGTCGGGGCAGACGGTAACTGTTCTAGAAAAAGTTCCAGTACCCAAAGGCCAACTGAA

GCAATACTTCTACGAGACCAAATGCAACCCTATGGGTTACATGAAGGAGGGCTGCAGAGG

CATTGACAAGAGGTACTGGAACTCCCAGTGCCGAACTACTCAGTCGTATGTGCGCGCGCT

CACCATGGATAACAAGAAGAAAGTGGGCTGGCGGTTTATCAGAATAGACACTTCATGTGT

ATGTACATTGACCATTGTTTGCTTGGGTTGCAGACCGAAGGCTGTTGTTCTACAAATATG

TCTACAAGCGATATAGAGCTGGGAAGCAAAGGGGTATGATTATAGAAACCGAAGGTGACA

GGCCTTCTTCAAAAGCTGACATTGAAATGGATGGAAAGATGGTCAACTCTCATACTGAAA

ATTTCATTGATGGCTCTCTAGTTCTGGAAGTCGATGAGAAAGATCAGGATGATGAAGAAG

CCAGGCGCGATATGGCCAGAATCCTAAAGGAGCTAAAGCAGAAACATCCTGACAAGGAAA

TAGAGCAACTGATTGAGTTAGCGAACTACCAGGTCTTGAGTCAACAGCAAAAAAGTCGAG

CATTCTATCGTATTCAAGCTACTCGCTTGATGACTGGGGCCGGAAATATTCTAAAGAGAC

ATGCAGCAGACCAGGCAAGGAAAGCTGTGAGCATGCAGGAAGTTAACTCTGAAGTGATCG

AAAATGAGCCCGTCAGTAAGATCTATTTCGAGCAAGC

>X86

CAACGGCCGCGGTATCATGACCGTGCAAAGGTAGCGTAATCACTTGTCTTTTAAATAAAG

ACCCGTATGAAAGGCAAAACGAAAGTTCAACTGTCTCTTTAATCCAATCAATGAAATTTA

TCTTTTCGTGCAGAGGCGGAGATAAATATATAAGACGAGAAGACCCTATGGAGCTTTAAA

CACACTATAATATAAGTTTTGGGTTGGGGCGACCACGGAGCAAAAAAATCCTCCGAGATA

AGAATAACACTTCGAAAATTAAAACATTTTGATCCACTAAGTGACCAACGAACCAAGTTA

CCCTAGGGATAACAGCGCAATCCTTTCTAAGAGTCCATATCGACGAATGGGTTTACGACC

TCGATGTTGGATCAGGACACCCAAATGGTGCAGCCGCTGTTAAGGTTCGTTTGTTCAACG

ATTAAAGTCCTACATGAACCCATATGCACTCTCAATTCTATTATCAAGCCTAGCAGTAGG

AACAATTACAACCCTTTCAAGCTTCCACTGATTCCTCGCATGACTAGGCTTAGAAATTAA

CACATTAGCAATCATCCCCCTGATAACAAAAATACACCACCCACGAGCAACAGAATCGGC

CACAAAATACTTCCTGACGCAAGCCACCGCATCAGCCCTAATCTTATTTTCTACAATCAC

AAACGCATGGGCCACAGGAGAATGAACAATTATCACCATAAATGACAACACATCAACACT

TATCTTAACAATCGCCCTAGCCATTAAGCTTGGAATCGCCCCATTTCACCTGTGATTACC

AGACGTCATACAAGGGCTAAACCTAGCAACATGTTTAATCCTTACAACATGACAAAAACT

AGCCCCAATATCATTAATAATTATAACTAGCCATCAATTAAACACAAACCTACTAATTAC

AATAGCCATATTATCAACAGTAATTGGGGGGTGGGGGGGCCTTAACCAAACACAAATACG

AAAAATAATAGCATATTCATCTATTGCCCACCTCGGATGAATAACACTAATCTTGTCTTT

CTCCCCAAACTTAACAACCCTGAACCTAATAATTTACCTAATACTAACCTCATCGATATT

TTTAATAATAATAACCTTGACCTCGACAAATATAAACAAACTTTCAACATCTTGACTGAA

AACGCCACTACTAGCATCATCAATAATAGTTACACTCATAGCCCTGGGAGGCCTACCCCC

AACCTCAGGCTTCTTACCAAAATGACTAATCCTACAAGAAATAACTAAACAACACCTAGG

AGCCGTATCTACACTAATAACTATATCTGCCCTACTAAGCCTATTCTTCTACCTACGACT

ATCATATGCAATCTCACTAACCACTGCGCCAAACACCTCAAACTCCAACATAACCTGACG

AGTAGCCCACAGCCAAATACAAGTCCTCCCAACAATAATTACACTAACTACAATAATACT

ACCAATCACACCAACACTACTAGCAACAGGACAAGGCAGCTTGGCGTACCCAGGTCTGAG

GACCCACGGGACTTTGGAGAGCATAGGTGGGCCTAAAGGCAGTTCAAGAGGAGGGTTGCC

CTCACTAGCAGACACTTTTGAACAAGTCATTGAGGAGATGCTTGAAGATGAGCAGAGCAT

GAGGCCCAGTGAGGAGAACAAGGATGCGGACATGTTTACATCTCGCGTAATGCTGAGCAG

TCAAGTGCCTTTGGAGCCTCCTCTTCTCTTTCTGCTGGAGGAGTACAAAAACTACTTGGA

TGCTGCAAATATGTCCATGAGGGTCCGACGTCACTCTGACCCCGCCCGCCGCGGGGAGCT

GAGTGTGTGTGACAGTATTAGTGAGTGGGTGACGGCATCTGACAAAAAGACTGCCGTGGA

CATGTCGGGGCAGACGGTAACTGTTCTAGAAAAAGTTCCAGTACCCAAAGGCCAACTGAA

GCAATACTTCTACGAGACCAAATGCAACCCTATGGGTTACATGAAGGAGGGCTGCAGAGG

CATTGACAAGAGGTACTGGAACTCCCAGTGCCGAACTACTCAGTCGTATGTGCGCGCGCT

CACCATGGATAACAAGAAGAAAGTGGGCTGGCGGTTTATCAGAATAGACACTTCATGTGT

ATGTACATTGACCATTGTTTGCTTGGGTTGCAGACCGAAGGCTGTTGTTCTACAAATATG

TCTACAAGCGATATAGAGCTGGGAAGCAAAGGGGTATGATTATAGAAACCGAAGGTGACA

GGCCTTCTTCAAAAGCTGACATTGAAATGGATGGAAAGATGGTCAACTCTCATACTGAAA

ATTTCATTGATGGCTCTCTAGTTCTGGAAGTCGATGAGAAAGATCAGGATGATGAAGAAG

CCAGGCGCGATATGGCCAGAATCCTAAAGGAGCTAAAGCAGAAACATCCTGACAAGGAAA

TAGAGCAACTGATTGAGTTAGCGAACTACCAGGTCTTGAGTCAACAGCAAAAAAGTCGAG

CATTCTATCGTATTCAAGCTACTCGCTTGATGACTGGGGCCGGAAATATTCTAAAGAGAC

ATGCAGCAGACCAGGCAAGGAAAGCTGTGAGCATGCAGGAAGTTAACTCTGAAGTGATCG

AAAATGAGCCCGTCAGTAAGATCTATTTCGAGCAAGC

>X91

CAACGGCCGCGGTATCATGACCGTGCAAAGGTAGCGTAATCACTTGTCTTTTAAATAAAG

ACCCGTATGAAAGGCAAAACGAAAGTTCGACTGTCTCTTTAATCCAATCAATGAAATTTA

TCTTTTCGTGCAGAGGCGGAGATAAATATATAAGACGAGAAGACCCTATGGAGCTTTAAA

CACACTATAATATAAGTTTTAGGTTGGGGCGACCACGGAGCAAAAAAATCCTCCGAGATA

AGAATAACACTTCGAAAATTAAAACATTTTGATCCACTAAGTGACCAACGAACCAAGTTA

CCCTAGGGATAACAGCGCAATCCTTTCTAAGAGTCCATATCGACGAATGGGTTTACGACC

TCGATGTTGGATCAGGACACCCAAATGGTGCAGCCGCTGTTAAGGTTCGTTTGTTCAACG

ATTAAAGTCCTACATGAACCCATATGCACTCTCAATTCTATTATCAAGCCTGGCAGTAGG

AACAATTACAACTCTTTCAAGCTCCCACTGATTTCTCGCATGACTAGGCTTAGAAATAAA

TACACTAACAATCATCCCCCTAATAACAAAAATACATCACCCACGAGCAACAGAATCAGC

CACAAAATACTTCCTAACGCAAGCCGCCGCATCAGCCCTAATCTTATTTTCTACAATCAC

AAACGCATGGGCCACAGGAGAATGAACAATTATCACCATAAATGACAACACATCAACACT

TATCTTAACAATCGCCCTAGCCATTAAACTGGGAATCGCCCCATTTCACCTGTGATTACC

AGACGTCATACAAGGGCTAAACCTAGCAACATGTTTAATCCTTACAACATGACAAAAACT

AGCCCCAATATCATTAATAATCATAACTAGCCATCAATTAAACGCAAACCTACTAATTAC

AATAGCCATACTATCAACAGTAATTGGGGGGTGGGGGGGCCTTAACCAAACACAAATCCG

AAAAATAATAGCATATTCATCTATCGCTCACCTCGGATGAATAACACTTATCCTGTCTTT

CTCCCCAAACTTAACGACCCTAAACTTAATAATTTACCTAATACTAACCTCATCAATATT

TTTAATAATAATAACCCTAACCTCAACAAATATAAACAAACTTTCAACATCTTGACTGAA

AACGCCACTACTAGCATCATCAATAATAATTACACTCATAGCCCTGGGAGGCCTACCCCC

AACCTCAGGCTTCTTACCAAAATGATTGATCCTACAAGAAATAACTAAACAACACCTAGG

AGCCGTATCTACACTAATAGCTATATCTGCCCTACTAAGCCTATTCTTCTATTTACGACT

ATCATATGCAATCTCACTAACCACTGCACCAAACACCTCAAACTCCAACATAACCTGACG

AGTAACCCACAACCAAATACAAGTCCTCCCAACAATAATCACACTAACTACAATTATACT

ACCAATCACACCAACACTATTAGCAACAGGACAAGGCAGCTTGGCGTACCCAGGTCTGAG

GACCCACGGGACTTTGGAGAGCATAGGTGGGCCTAAAGGCAGTTCAAGAGGAGGGTTGCC

CTCACTAGCAGACACTTTTGAACAAGTCATTGAGGAGATGCTTGAAGATGAGCAGAGCAT

GAGGCCCAGTGAGGAGAACAAGGATGCGGACATGTTTACATCTCGCGTAATGCTGAGCAG

TCAAGTGCCTTTGGAGCCTCCTCTTCTCTTTCTGCTGGAGGAGTACAAAAACTACTTGGA

TGCTGCAAATATGTCCATGAGGGTCCGACGTCACTCTGACCCCGCCCGCCGCGGGGAGCT

GAGTGTGTGTGACAGTATTAGTGAGTGGGTGACGGCATCTGACAAAAAGACTGCCGTGGA

CATGTCGGGGCAGACGGTAACTGTTCTAGAAAAAGTTCCAGTACCCAAAGGCCAACTGAA

GCAATACTTCTACGAGACCAAATGCAACCCTATGGGTTACATGAAGGAGGGCTGCAGAGG

CATTGACAAGAGGTACTGGAACTCCCAGTGCCGAACTACTCAGTCGTATGTGCGCGCGCT

CACCATGGATAACAAGAAGAAAGTGGGCTGGCGGTTTATCAGAATAGACACTTCATGTGT

ATGTACATTGACCATTGTTTGCTTGGGTTGCAGACCGAAGGCTGTTGTTCTACAAATATG

TCTACAAGCGATATAGAGCTGGGAAGCAAAGGGGTATGATTATAGAAACCGAAGGTGACA

GGCCTTCTTCAAAAGCTGACATTGAAATGGATGGAAAGATGGTCAACTCTCATACTGAAA

ATTTCATTGATGGCTCTCTAGTTCTGGAAGTCGATGAGAAAGATCAGGATGATGAAGAAG

CCAGGCGCGATATGGCCAGAATCCTAAAGGAGCTAAAGCAGAAACATCCTGACAAGGAAA

TAGAGCAACTGATTGAGTTAGCGAACTACCAGGTCTTGAGTCAACAGCAAAAAAGTCGAG

CATTCTATCGTATTCAAGCTACTCGCTTGATGACTGGGGCCGGAAATATTCTAAAGAGAC

ATGCAGCAGACCAGGCAAGGAAAGCTGTGAGCATGCAGGAAGTTAACTCTGAAGTGATCG

AAAATGAGCCCGTCAGTAAGATCTATTTCGAGCAAGC

>X92

CAACGGCCGCGGTATCATGACCGTGCAAAGGTAGCGTAATCACTTGTCTTTTAAATAAAG

ACCCGTATGAAAGGCAAAACGAAAGTTCAACTGTCTCTTTAATCCAATCAATGAAATTTA

TCTTTTCGTGCAGAGGCGGAGATAAATATATAAGACGAGAAGACCCTATGGAGCTTTAAA

CACACTATAATATAAGTTTTGGGTTGGGGCGACCACGGAGCAAAAAAATCCTCCGAGATA

AGAATAACACTTCGAAAATTAAAACATTTTGATCCACTAAGTGACCAACGAACCAAGTTA

CCCTAGGGATAACAGCGCAATCCTTTCTAAGAGTCCATATCGACGAATGGGTTTACGACC

TCGATGTTGGATCAGGACACCCAAATGGTGCAGCCGCTGTTAAGGTTCGTTTGTTCAACG

ATTAAAGTCCTACATGAACCCATATGCACTCTCAATCCTATTATCAAGCCTGGCAGTAGG

AACAATTACAACCCTTTCAAGCTCCCACTGATTCCTCGCATGACTAGGCTTAGAAATTAA

CACATTAGCAATCATCCCCCTGATAACAAAAATACACCACCCACGAGCAACAGAATCGGC

CACAAAATACTTCCTGACGCAAGCCACCGCATCAGCCCTAATCTTATTTTCTACAATCAC

AAACGCATGGGCCACAGGAGAATGAACAATTATCACCATAAATGACAACACATCAACACT

TATCTTAACAATCGCCCTAGCCATTAAGCTTGGAATCGCCCCATTTCACCTGTGATTACC

AGACGTCATACAAGGGCTAAACCTAGCAACATGTTTAATCCTTACAACATGACAAAAACT

AGCCCCAATATCATTAATAATTATAACTAGCCATCAATTAAACACAAACCTACTAATTAC

AATAGCCATATTATCAACAGTAATTGGGGGGTGGGGGGGCCTTAACCAAACACAAATACG

AAAAATAATAGCATATTCATCTATTGCCCACCTCGGATGAATAACACTAATCTTGTCTTT

CTCCCCAAACTTAACAACCCTGAACCTAATAATTTACCTAATACTAACCTCATCGATATT

TTTAATAATAATAACCTTGACCTCGACAAATATAAACAAACTTTCAACATCTTGACTGAA

AACGCCACTACTAGCGTCATCAATAATAGTTACACTCATAGCCCTGGGAGGCCTACCCCC

AACCTCAGGCTTCTTACCAAAATGACTAATCCTACAAGAAATAACTAAACAACACCTAGG

AGCCGTATCTACACTAATAGCTATATCTGCCCTACTAAGCCTATTCTTCTACCTACGACT

ATCATATGCAATCTCACTAACCACTGCGCCAAACACCTCAAACTCCAACATAACCTGACG

AGTAGCCCGCAGCCAAATACAAGTCCTCCCAACAATAATTACACTAACTACAATAATACT

ACCAATCACACCAACACTACTAGCAACAGGACAAGGCAGCTTGGCGTACCCAGGTCTGAG

GACCCACGGGACTTTGGAGAGCATAGGTGGGCCTAAAGGCAGTTCAAGAGGAGGGTTGCC

CTCACTAGCAGACACTTTTGAACAAGTCATTGAGGAGATGCTTGAAGATGAGCAGAGCAT

GAGGCCCAGTGAGGAGAACAAGGATGCGGACATGTTTACATCTCGCGTAATGCTGAGCAG

TCAAGTGCCTTTGGAGCCTCCTCTTCTCTTTCTGCTGGAGGAGTACAAAAACTACTTGGA

TGCTGCAAATATGTCCATGAGGGTCCGACGTCACTCTGACCCCGCCCGCCGCGGGGAGCT

GAGTGTGTGTGACAGTATTAGTGAGTGGGTGACGGCATCTGACAAAAAGACTGCCGTGGA

CATGTCGGGGCAGACGGTAACTGTTCTAGAAAAAGTTCCAGTACCCAAAGGCCAACTGAA

GCAATACTTCTACGAGACCAAATGCAACCCTATGGGTTACATGAAGGAGGGCTGCAGAGG

CATTGACAAGAGGTACTGGAACTCCCAGTGCCGAACTACTCAGTCGTATGTGCGCGCGCT

CACCATGGATAACAAGAAGAAAGTGGGCTGGCGGTTTATCAGAATAGACACTTCATGTGT

ATGTACATTGACCATTGTTTGCTTGGGTTGCAGACCGAAGGCTGTTGTTCTACAAATATG

TCTACAAGCGATATAGAGCTGGGAAGCAAAGGGGTATGATTATAGAAACCGAAGGTGACA

GGCCTTCTTCAAAAGCTGACATTGAAATGGATGGAAAGATGGTCAACTCTCATACTGAAA

ATTTCATTGATGGCTCTCTAGTTCTGGAAGTCGATGAGAAAGATCAGGATGATGAAGAAG

CCAGGCGCGATATGGCCAGAATCCTAAAGGAGCTAAAGCAGAAACATCCTGACAAGGAAA

TAGAGCAACTGATTGAGTTAGCGAACTACCAGGTCTTGAGTCAACAGCAAAAAAGTCGAG

CATTCTATCGTATTCAAGCTACTCGCTTGATGACTGGGGCCGGAAATATTCTAAAGAGAC

ATGCAGCAGACCAGGCAAGGAAAGCTGTGAGCATGCAGGAAGTTAACTCTGAAGTGATCG

AAAATGAGCCCGTCAGTAAGATCTATTTCGAGCAAGC

>X93

CAACGGCCGCGGTATCATGACCGTGCAAAGGTAGCGTAATCACTTGTCTTTTAAATAAAG

ACCTGTATGAAAGGCAAAACGAAAGTTCAACTGTCTCTTTAATCCAATCAATGAAATTTA

TCTTTTCGTGCAGAAGCGGAAATGAATATATAAGACGAGAAGACCCTATGGAGCTTTAAA

CACACTATAATACAAGTTTTTGGTTGGGGCGACCGCGGAGAAAAGAAACCCTCCGAGATA

AGAATAACACTTCGAAAATTAAAAAATTTTGATCCACTAAGTGACCAACGAACCAAGTTA

CCCTAGGGATAACAGCGCAATCCTTTCTAAGAGTCCATATCGACGAATGGGTTTACGACC

TCGATGTTGGATCAGGACACCCAAATGGTGCAGCCGCTATTAAGGTTCGTTTGTTCAACG

ATTAAAGTCCTACATGAACCCGTATGCACTATCAACCCTGTTGTCGAGCCTAGCAGTAGG

AACAATTACAACTCTCTCAAGCTCCCATTGATTTCTGGCATGAGTAGGACTAGAAATTAA

TACACTAGCAATAATCCCCCTAATAACAAAAATACACCACCCACGATCAACAGAATCAGC

CACAAAATACTTCCTAACGCAAGCCGCCGCATCAGCCTTAATCTTATTTTCTACAATCAT

AAACGCATGGGCTACAGGAGAATGAACAATTATCACCATAAATGATAACTCATCAACATT

TATATTAACAGTTGCCCTAGCTATTAAACTTGGAATCGCCCCTTTTCATCTGTGACTACC

AGACGTCATACAAGGGTTAAACCTAACAACATGCCTAATCCTTGCAACATGACAAAAATT

AGCCCCAATATCCTTAATAATTATAACCAATCACCAATTAAACACAAACCTGCTAATTAC

AATAGCCGTATTATCAGCAGTAATTGGAGGATGAGGGGGACTTAACCAAACACAAATCCG

AAAAATAATAGCATATTCATCTATCGCCCACCTTGGGTGAATAACACTAGTCCTCTCTTT

CTCCCCAAATTTAACAACCCTTAACCTAATAATCTACCTAATATTAACATCATCAATGTT

CTTAATAATAACAACCCTAAACTCAACAAATATTAACAAACTTTCAACATCTTGACTGAA

AACCCCACTACTAGCATCATCAATAATAGTTACACTCATGGCCCTAGGAGGCCTACCCCC

AACATCAGGATTCCTACCAAAATGACTTATCCTACAAGAAATAACCAAACAGCACCTAGG

GGCCGTGTCCACACTAATAGCTATATCTGCACTACTAAGCCTATTCTTCTACTTACGACT

ATCATATGCAATCTCACTAACTACCGCACGAAACACATCAAACTCCAACATAACCTGACG

AATAACCCACAACCAAATACAAATCTTACCAACAATAATTACACTAACTACAATAATACT

ACCGATCACACCAACACTACTAGCAATAGGACAAGGCAGCTTGGCGTACCCAGGTCTGAG

GACCCACGGGACTTTGGAGAGCATAGGTGGGCCTAAAGGCAGTTCAAGAGGAGGGTTGCC

CTCACTAGCAGACACTTTTGAACAAGTCATTGAGGAGATGCTTGAAGATGAGCAGAGCAT

GAGGCCCAGTGAGGAGAACAAGGATGCGGACATGTTTACATCTCGCGTAATGCTGAGCAG

TCAAGTGCCTTTGGAGCCTCCTCTTCTCTTTCTGCTGGAGGAGTACAAAAACTACTTGGA

TGCTGCAAATATGTCCATGAGGGTCCGACGTCACTCTGACCCCGCCCGCCGCGGGGAGCT

GAGTGTGTGTGACAGTATTAGTGAGTGGGTGACGGCATCTGACAAAAAGACTGCCGTGGA

CATGTCGGGGCAGACGGTAACTGTTCTAGAAAAAGTTCCAGTACCCAAAGGCCAACTGAA

GCAATACTTCTACGAGACCAAATGCAACCCTATGGGTTACATGAAGGAGGGCTGCAGAGG

CATTGACAAGAGGTACTGGAACTCCCAGTGCCGAACTACTCAGTCGTATGTGCGCGCGCT

CACCATGGATAACAAGAAGAAAGTGGGCTGGCGATTTATCAGAATAGACACTTCATGTGT

ATGTACATTGACCATTGTTTGCTTGGGTTGCAGACCGAAGGCTGTTGTTCTACAAATATG

TCTACAAGCGATATAGAGCTGGGAAGCAAAGGGGTATGATTATAGAAACCGAAGGTGACA

GGCCTTCTTCAAAAGCTGACATTGAAATGGATGGAAAGATGGTCAACTCTCATACTGAAA

ATTTCATTGATGGCTCTCTAGTTCTGGAAGTCGATGAGAAAGATCAGGATGATGAAGAAG

CCAGGCGCGATATGGCCAGAATCCTAAAGGAGCTAAAGCAGAAACATCCTGACAAAGAAA

TAGAGCAACTGATTGAGTTAGCGAACTACCAGGTCTTGAGTCAACAGCAAAAAAGTCGAG

CATTCTATCGTATTCAAGCTACTCGCTTGATGACTGGGGCCGGAAATATTCTAAAGAGAC

ATGCAGCAGACCAGGCAAGGAAAGCTGTGAGCATGCAGGAAGTTAACTCTGAAGTGATCG

AAAATGAGCCCGTCAGTAAGATCTATTTCGAGCAAGC

>X94

CAACGGCCGCGGTATCATGACCGTGCAAAGGTAGCGTAATCACTTGTCTTTTAAATAAAG

ACCTGTATGAAAGGCAAAACGAAAGTTCAACTGTCTCTTTAATCCAATCAATGAAATTTA

TCTTTTCGTGCAGAAGCGGAAATGAATATATAAGACGAGAAGACCCTATGGAGCTTTAAA

CACACTATAATACAAGTTTTTGGTTGGGGCGACCGCGGAGAAAAGAAACCCTCCGAGATA

AGAATAACACTTCGAAAATTAAAAAATTTTGATCCACTAAGTGACCAACGAACCAAGTTA

CCCTAGGGATAACAGCGCAATCCTTTCTAAGAGTCCATATCGACGAATGGGTTTACGACC

TCGATGTTGGATCAGGACACCCAAATGGTGCAGCCGCTATTAAGGTTCGTTTGTTCAACG

ATTAAAGTCCTACATGAACCCGTATGCACTATCAACCCTGTTGTCGAGCCTAGCAGTAGG

AACAATTACAACTCTCTCAAGCTCCCATTGATTTCTGGCATGAGTAGGACTAGAAATTAA

TACACTAGCAATAATCCCCCTAATAACAAAAATACACCACCCACGATCAACAGAATCAGC

CACAAAATACTTCCTAACGCAAGCCGCCGCATCAGCCTTAATCTTATTTTCTACAATCAT

AAACGCATGGGCTACAGGAGAATGAACAATTATCACCATAAATGATAACTCATCAACATT

TATATTAACAGTTGCCCTAGCTATTAAACTTGGAATCGCCCCTTTTCATCTGTGACTACC

AGACGTCATACAAGGGTTAAACCTAACAACATGCCTAATCCTTGCAACATGACAAAAATT

AGCCCCAATATCCTTAATAATTATAACCAATCACCAATTAAACACAAACCTGCTAATTAC

AATAGCCGTATTATCAGCAGTAATTGGAGGATGAGGGGGACTTAACCAAACACAAATCCG

AAAAATAATAGCATATTCATCTATCGCCCACCTTGGGTGAATAACACTAGTCCTCTCTTT

CTCCCCAAATTTAACAACCCTTAACCTAATAATCTACCTAATATTAACATCATCAATGTT

CTTAATAATAACAACCCTAAACTCAACAAATATTAACAAACTTTCAACATCTTGACTGAA

AACCCCACTACTAGCATCATCAATAATAGTTACACTCATGGCCCTAGGAGGCCTACCCCC

AACATCAGGATTCCTACCAAAATGACTTATCCTACAAGAAATAACCAAACAGCACCTAGG

GGCCGTGTCCACACTAATAGCTATATCTGCACTACTAAGCCTATTCTTCTACTTACGACT

ATCATATGCAATCTCACTAACTACCGCACCAAACACATCAAACTCCAACATAACCTGACG

AATAACCCACAACCAAATACAAATCTTACCAACAATAATTACACTAACTACAATAATACT

ACCGATCACACCAACACTACTAGCAATAGGACAAGGCAGCTTGGCGTACCCAGGTCTGAG

GACCCACGGGACTTTGGAGAGCATAGGTGGGCCTAAAGGCAGTTCAAGAGGAGGGTTGCC

CTCACTAGCAGACACTTTTGAACAAGTCATTGAGGAGATGCTTGAAGATGAGCAGAGCAT

GAGGCCCAGTGAGGAGAACAAGGATGCGGACATGTTTACATCTCGCGTAATGCTGAGCAG

TCAAGTGCCTTTGGAGCCTCCTCTTCTCTTTCTGCTGGAGGAGTACAAAAACTACTTGGA

TGCTGCAAATATGTCCATGAGGGTCCGACGTCACTCTGACCCCGCCCGCCGCGGGGAGCT

GAGTGTGTGTGACAGTATTAGTGAGTGGGTGACGGCATCTGACAAAAAGACTGCCGTGGA

CATGTCGGGGCAGACGGTAACTGTTCTAGAAAAAGTTCCAGTACCCAAAGGCCAACTGAA

GCAATACTTCTACGAGACCAAATGCAACCCTATGGGTTACATGAAGGAGGGCTGCAGAGG

CATTGACAAGAGGTACTGGAACTCCCAGTGCCGAACTACTCAGTCGTATGTGCGCGCGCT

CACCATGGATAACAAGAAGAAAGTGGGCTGGCGGTTTATCAGAATAGACACTTCATGTGT

ATGTACATTGACCATTGTTTGCTTGGGTTGCAGACCGAAGGCTGTTGTTCTACAAATATG

TCTACAAGCGATATAGAGCTGGGAAGCAAAGGGGTATGATTATAGAAACCGAAGGTGACA

GGCCTTCTTCAAAAGCTGACATTGAAATGGATGGAAAGATGGTCAACTCTCATACTGAAA

ATTTCATTGATGGCTCTCTAGTTCTGGAAGTCGATGAGAAAGATCAGGATGATGAAGAAG

CCAGGCGCGATATGGCCAGAATCCTAAAGGAGCTAAAGCAGAAACATCCTGACAAAGAAA

TAGAGCAACTGATTGAGTTAGCGAACTACCAGGTCTTGAGTCAACAGCAAAAAAGTCGAG

CATTCTATCGTATTCAAGCTACTCGCTTGATGACTGGGGCCGGAAATATTCTAAAGAGAC

ATGCAGCAGACCAGGCAAGGAAAGCTGTGAGCATGCAGGAAGTTAACTCTGAAGTGATCG

AAAATGAGCCCGTCAGTAAGATCTATTTCGAGCAAGC

>X95

CAACGGCCGCGGTATCATGACCGTGCAAAGGTAGCGTAATCACTTGTCTTTTAAATAAAG

ACCTGTATGAAAGGCAAAACGAAAGTTCAACTGTCTCTTTAATCCAATCAATGAAATTTA

TCTTTTCGTGCAGAAGCGGAAATGAATATATAAGACGAGAAGACCCTATGGAGCTTTAAA

CACACTATAATACAAGTTTTTGGTTGGGGCGACCGCGGAGAAAAGAAACCCTCCGAGATA

AGAATAACACTTCGAAAATTAAAAAATTTTGATCCACTAAGTGACCAACGAACCAAGTTA

CCCTAGGGATAACAGCGCAATCCTTTCTAAGAGTCCATATCGACGAATGGGTTTACGACC

TCGATGTTGGATCAGGACACCCAAATGGTGCAGCCGCTATTAAGGTTCGTTTGTTCAACG

ATTAAAGTCCTACATGAACCCGTATGCACTATCAATCCTGTTGTCGAGCCTAGCAGTAGG

AACAATTACAACTCTCTCAAGCTCCCATTGATTTCTGGCATGAGTAGGACTAGAAATTAA

TACACTAGCAATAATCCCCCTAATAACAAAAATACACCACCCACGATCAACAGAATCAGC

CACAAAATACTTCCTAACGCAAGCCGCCGCATCAGCCTTAATCTTATTTTCTACAATCAT

AAACGCATGGGCTACAGGAGAATGAACAATTATCACCATAAATGATAACTCATCAACATT

TATATTAACAGTTGCCCTAGCTATTAAACTTGGAATCGCCCCTTTTCATCTGTGACTACC

AGACGTCATACAAGGGTTAAACCTAACAACATGCCTAATCCTTGCAACATGACAAAAATT

AGCCCCAATATCCTTAATAATTATAACCAATCACCAATTAAACACAAACCTGCTAATTAC

AATAGCCGTATTATCAGCAGTAATTGGAGGATGAGGGGGACTTAACCAAACACAAATCCG

AAAAATAATAGCATATTCATCTATCGCCCACCTTGGGTGAATAACACTAGTCCTCTCTTT

CTCCCCAAATTTAACAACCCTTAACCTAATAATCTACCTAATATTAACATCATCAATGTT

CTTAATAATAACAACCCTAAACTCAACAAATATTAACAAACTTTCAACATCTTGACTGAA

AACCCCACTACTAGCATCATCAATAATAGTTACACTCATGGCCCTAGGAGGCCTACCCCC

AACATCAGGATTCCTACCAAAATGACTTATCCTACAAGAAATAACCAAACAGCACCTAGG

GGCCGTGTCCACACTAATAGCTATATCTGCACTACTAAGCCTATTCTTCTACTTACGACT

ATCATATGCAATCTCACTAACTACCGCACCAAACACATCAAACTCCAACATAACCTGACG

AATAACCCACAACCAAATACAAATCTTACCAACAATAATTACACTAACTACAATAATACT

ACCGATCACACCAACACTACTAGCAATAGGACAAGGCAGCTTGGCGTACCCCGGTCTGAG

GACCCACGGGACTTTGGAGAGCATAGGTGGGCCTAAAGGCAGTTCAAGAGGAGGGTTGCC

CTCACTAGCAGACACTTTTGAACAAGTCATTGAGGAGATGCTTGAAGATGAGCAGAGCAT

GAGGCCCAGTGAGGAGAACAAGGATGCGGACATGTTTACATCTCGCGTAATGCTGAGCAG

TCAAGTGCCTTTGGAGCCTCCTCTTCTCTTTCTGCTGGAGGAGTACAAAAACTACTTGGA

TGCTGCAAATATGTCCATGAGGGTCCGACGTCACTCTGACCCCGCCCGCCGCGGGGAGCT

GAGTGTGTGTGACAGTATTAGTGAGTGGGTGACGGCATCTGACAAAAAGACTGCCGTGGA

CATGTCGGGGCAGACGGTAACTGTTCTAGAAAAAGTTCCAGTACCCAAAGGCCAACTGAA

GCAATACTTCTACGAGACCAAATGCAACCCTATGGGTTACATGAAGGAGGGCTGCAGAGG

CATTGACAAGAGGTACTGGAACTCCCAGTGCCGAACTACTCAGTCGTATGTGCGCGCGCT

CACCATGGATAACAAGAAGAAAGTGGGCTGGCGGTTTATCAGAATAGACACTTCATGTGT

ATGTACATTGACCATTGTTTGCTTGGGTTGCAGACCGAAGGCTGTTGTTCTACAAATATG

TCTACAAGCGATATAGAGCTGGGAAGCAAAGGGGTATGATTATAGAAACCGAAGGTGACA

GGCCTTCTTCAAAAGCTGACATTGAAATGGATGGAAAGATGGTCAACTCTCATACTGAAA

ATTTCATTGATGGCTCTCTAGTTCTGGAAGTCGATGAGAAAGATCAGGATGATGAAGAAG

CCAGGCGCGATATGGCCAGAATCCTAAAGGAGCTAAAGCAGAAACATCCTGACAAAGAAA

TAGAGCAACTGATTGAGTTAGCGAACTACCAGGTCTTGAGTCAACAGCAAAAAAGTCGAG

CATTCTATCGTATTCAAGCTACTCGCTTGATGACTGGGGCCGGAAATATTCTAAAGAGAC

ATGCAGCAGACCAGGCAAGGAAAGCTGTGAGCATGCAGGAAGTTAACTCTGAAGTGATCG

AAAATGAGCCCGTCAGTAAGATCTATTTCGAGCAAGC

>X96

CAACGGCCGCGGTATCATGACCGTGCAAAGGTAGCGTAATCACTTGTCTTTTAAATAAAG

ACCTGTATGAAAGGCAAAACGAAAGTTCAACTGTCTCTTTAATCCAATCAATGAAATTTA

TCTTTTCGTGCAGAAGCGGAAATGAATATATAAGACGAGAAGACCCTATGGAGCTTTAAA

CACACTATAATACAAGTTTTTGGTTGGGGCGACCGCGGAGAAAAGAAACCCTCCGAGATA

AGAATAACACTTCGAAAATTAAAAAATTTTGATCCACTAAGTGACCAACGAACCAAGTTA

CCCTAGGGATAACAGCGCAATCCTTTCTAAGAGTCCATATCGACGAATGGGTTTACGACC

TCGATGTTGGATCAGGACACCCAAATGGTGCAGCCGCTATTAAGGTTCGTTTGTTCAACG

ATTAAAGTCCTACATGAACCCGTATGCACTATCAATCCTGTTGTCGAGCCTAGCAGTAGG

AACAATTACAACTCTCTCAAGCTCCCATTGATTTCTGGCATGAGTAGGACTAGAAATTAA

TACACTAGCAATAATCCCCCTAATAACAAAAATACACCACCCACGATCAACAGAATCAGC

CACAAAATACTTCCTAACGCAAGCCGCCGCATCAGCCTTAATCTTATTTTCTACAATCAT

AAACGCATGGGCTACAGGAGAATGAACAATTATCACCATAAATGATAACTCATCAACATT

TATATTAACAGTTGCCCTAGCTATTAAACTTGGAATCGCCCCTTTTCATCTGTGACTACC

AGACGTCATACAAGGGTTAAACCTAACAACATGCCTAATCCTTGCAACATGACAAAAATT

AGCCCCAATATCCTTAATAATTATAACCAATCACCAATTAAACACAAACCTGCTAATTAC

AATAGCCGTATTATCAGCAGTAATTGGAGGATGAGGGGGACTTAACCAAACACAAATCCG

AAAAATAATAGCATATTCATCTATCGCCCACCTTGGGTGAATAACACTAGTCCTCTCTTT

CTCCCCAAATTTAACAACCCTTAACCTAATAATCTACCTAATATTAACATCATCAATGTT

CTTAATAATAACAACCCTAAACTCAACAAATATTAACAAACTTTCAACATCTTGACTGAA

AACCCCACTACTAGCATCATCAATAATAGTTACACTCATGGCCCTAGGAGGCCTACCCCC

AACATCAGGATTCCTACCAAAATGACTTATCCTACAAGAAATAACCAAACAGCACCTAGG

GGCCGTGTCCGCACTAATAGCTATATCTGCACTACTAAGCCTATTCTTCTACTTACGACT

ATCATATGCAATCTCACTAACTACCGCACCAAACACATCAAACTCCAACATAACCTGACG

AATAACCCACAACCAAATACAAATCTTACAAACAATAATTACACTAACTACAATAATACT

ACCGATCACACCAACACTACTAGCAATAGGACAAGGCAGCTTGGCGTACCCAGGTCTGAG

GACCCACGGGACTTTGGAGAGCATAGGTGGGCCTAAAGGCAGTTCAAGAGGAGGGTTGCC

CTCACTAGCAGACACTTTTGAACAAGTCATTGAGGAGATGCTTGAAGATGAGCAGAGCAT

GAGGCCCAGTGAGGAGAACAAGGATGCGGACATGTTTACATCTCGCGTAATGCTGAGCAG

TCAAGTGCCTTTGGAGCCTCCTCTTCTCTTTCTGCTGGAGGAGTACAAAAACTACTTGGA

TGCTGCAAATATGTCCATGAGGGTCCGACGTCACTCTGACCCCGCCCGCCGCGGGGAGCT

GAGTGTGTGTGACAGTATTAGTGAGTGGGTGACGGCATCTGACAAAAAGACTGCCGTGGA

CATGTCGGGGCAGACGGTAACTGTTCTAGAAAAAGTTCCAGTACCCAAAGGCCAACTGAA

GCAATACTTCTACGAGACCAAATGCAACCCTATGGGTTACATGAAGGAGGGCTGCAGAGG

CATTGACAAGAGGTACTGGAACTCCCAGTGCCGAACTACTCAGTCGTATGTGCGCGCGCT

CACCATGGATAACAAGAAGAAAGTGGGCTGGCGGTTTATCAGAATAGACACTTCATGTGT

ATGTACATTGACCATTGTTTGCTTGGGTTGCAGACCGAAGGCTGTTGTTCTACAAATATG

TCTACAAGCGATATAGAGCTGGGAAGCAAAGGGGTATGATTATAGAAACCGAAGGTGACA

GGCCTTCTTCAAAAGCTGACATTGAAATGGATGGAAAGATGGTCAACTCTCATACTGAAA

ATTTCATTGATGGCTCTCTAGTTCTGGAAGTCGATGAGAAAGATCAGGATGATGAAGAAG

CCAGGCGCGATATGGCCAGAATCCTAAAGGAGCTAAAGCAGAAACATCCTGACAAAGAAA

TAGAGCAACTGATTGAGTTAGCGAACTACCAGGTCTTGAGTCAACAGCAAAAAAGTCGAG

CATTCTATCGTATTCAAGCTACTCGCTTGATGACTGGGGCCGGAAATATTCTAAAGAGAC

ATGCAGCAGACCAGGCAAGGAAAGCTGTGAGCATGCAGGAAGTTAACTCTGAAGTGATCG

AAAATGAGCCCGTCAGTAAGATCTATTTCGAGCAAGC

>X97

CAACGGCCGCGGTATCATGACCGTGCAAAGGTAGCGTAATCACTTGTCTTTTAAATAAAG

ACCTGTATGAAAGGCAAAACGAAAGTTCAACTGTCTCTTTAATCCAATCAATGAAATTTA

TCTTTTCGTGCAGAAGCGGAAATGAATATATAAGACGAGAAGACCCTATGGAGCTTTAAA

CACACTATAATACAAGTTTTTGGTTGGGGCGACCGCGGAGAAAAGAAACCCTCCGAGATA

AGAATAACACTTCGAAAATTAAAAAATTTTGATCCACTAAGTGACCAACGAACCAAGTTA

CCCTAGGGATAACAGCGCAATCCTTTCTAAGAGTCCATATCGACGAATGGGTTTACGACC

TCGATGTTGGATCAGGACACCCAAATGGTGCAGCCGCTATTAAGGTTCGTTTGTTCAACG

ATTAAAGTCCTACATGAACCCGTATGCACTATCAATCCTGTTGTCGAGCCTAGCAGTAGG

AACAATTACAACTCTCTCAAGCTCCCATTGATTTCTGGCATGAGTAGGACTAGAAATTAA

TACACTAGCAATAATCCCCCTAATAACAAAAATACACCACCCACGATCAACAGAATCAGC

CACAAAATACTTCCTAACGCAAGCCGCCGCATCAGCCTTAATCTTATTTTCTACAATCAT

AAACGCATGGGCTACAGGAGAATGAACAATTATCACCATAAATGATAACTCATCAACATT

TATATTAACAGTTGCCCTAGCTATTAAACTTGGAATCGCCCCTTTTCATCTGTGACTACC

AGACGTCATACAAGGGTTAAACCTAACAACATGCCTAATCCTTGCAACATGACAAAAATT

AGCCCCAATATCCTTAATAATTATAACCAATCACCAATTAAACACAAACCTGCTAATTAC

AATAGCCGTATTATCAGCAGTAATTGGAGGATGAGGGGGACTTAACCAAACACAAATCCG

AAAAATAATAGCATATTCATCTATCGCCCACCTTGGGTGAATAACACTAGTCCTCTCTTT

CTCCCCAAATTTAACAACCCTTAACCTAATAATCTACCTAATATTAACATCATCAATGTT

CTTAATAATAACAACCCTAAACTCAACAAATATTAACAAACTTTCAACATCTTGACTGAA

AACCCCACTACTAGCATCATCAATAATAGTTACACTCATGGCCCTAGGAGGCCTACCCCC

AACATCAGGATTCCTACCAAAATGACTTATCCTACAAGAAATAACCAAACAGCACCTAGG

GGCCGTGTCCGCACTAATAGCTATATCTGCACTACTAAGCCTATTCTTCTACTTACGACT

ATCATATGCAATCTCACTAACTACCGCACCAAACACATCAAACTCCAACATAACCTGACG

AATAACCCACAACCAAATACAAATCTTACAAACAATAATTACACTAACTACAATAATACT

ACCGATCACACCAACACTACTAGCAATAGGACAAGGCAGCTTGGCGTACCCAGGTCTGAG

GACCCACGGGACTTTGGAGAGCATAGGTGGGCCTAAAGGCAGTTCAAGAGGAGGGTTGCC

CTCACTAGCAGACACTTTTGAACAAGTCATTGAGGAGATGCTTGAAGATGAGCAGAGCAT

GAGGCCCAGTGAGGAGAACAAGGATGCGGACATGTTTACATCTCGCGTAATGCTGAGCAG

TCAAGTGCCTTTGGAGCCTCCTCTTCTCTTTCTGCTGGAGGAGTACAAAAACTACTTGGA

TGCTGCAAATATGTCCATGAGGGTCCGACGTCACTCTGACCCCGCCCGCCGCGGGGAGCT

GAGTGTGTGTGACAGTATTAGTGAGTGGGTGACTGCATCTGACAAAAAGACTGCCGTGGA

CATGTCGGGGCAGACGGTAACTGTTCTAGAAAAAGTTCCAGTACCCAAAGGCCAACTGAA

GCAATACTTCTACGAGACCAAATGCAACCCTATGGGTTACATGAAGGAGGGCTGCAGAGG

CATTGACAAGAGGTACTGGAACTCCCAGTGCCGAACTACTCAGTCGTATGTGCGCGCGCT

CACCATGGATAACAAGAAGAAAGTGGGCTGGCGATTTATCAGAATAGACACTTCATGTGT

ATGTACATTGACCATTGTTTGCTTGGGTTGCAGACCGAAGGCTGTTGTTCTACAAATATG

TCTACAAGCGATATAGAGCTGGGAAGCAAAGGGGTATGATTATAGAAACCGAAGGTGACA

GGCCTTCTTCAAAAGCTGACATTGAAATGGATGGAAAGATGGTCAACTCTCATACTGAAA

ATTTCATTGATGGCTCTCTAGTTCTGGAAGTCGATGAGAAAGATCAGGATGATGAAGAAG

CCAGGCGCGATATGGCCAGAATCCTAAAGGAGCTAAAGCAGAAACATCCTGACAAAGAAA

TAGAGCAACTGATTGAGTTAGCGAACTACCAGGTCTTGAGTCAACAGCAAAAAAGTCGAG

CATTCTATCGTATTCAAGCTACTCGCTTGATGACTGGGGCCGGAAATATTCTAAAGAGAC

ATGCAGCAGACCAGGCAAGGAAAGCTGTGAGCATGCAGGAAGTTAACTCTGAAGTGATCG

AAAATGAGCCCGTCAGTAAGATCTATTTCGAGCAAGC

>X98

CAACGGCCGCGGTATCATGACCGTGCAAAGGTAGCGTAATCACTTGTCTTTTAAATAAAG

ACCTGTATGAAAGGCAAAACGAAAGTTCAACTGTCTCTTTAATCCAATCAATGAAATTTA

TCTTTTCGTGCAGAAGCGGAAATGAATATATAAGACGAGAAGACCCTATGGAGCTTTAAA

CACACTATAATACAAGTTTTTGGTTGGGGCGACCGCGGAGAAAAGAAACCCTCCGAGATA

AGAATAACACTTCGAAAATTAAAAAATTTTGATCCACTAAGTGACCAACGAACCAAGTTA

CCCTAGGGATAACAGCGCAATCCTTTCTAAGAGTCCATATCGACGAATGGGTTTACGACC

TCGATGTTGGATCAGGACACCCAAATGGTGCAGCCGCTATTAAGGTTCGTTTGTTCAACG

ATTAAAGTCCTACATGAACCCGTATGCACTATCAACCCTGTTGTCGAGCCTAGCAGTAGG

AACAATTACAACTCTCTCAAGCTCCCATTGATTTCTGGCATGAGTAGGACTAGAAATTAA

TACACTAGCAATAATCCCCCTAATAACAAAAATACACCACCCACGATCAACAGAATCAGC

CACAAAATACTTCCTAACGCAAGCCGCCGCATCAGCCTTAATCTTATTTTCTACAATCAT

AAACGCATGGGCTACAGGAGAATGAACAATTATCACCATAAATGATAACTCATCAACATT

TATATTAACAGTTGCCCTAGCTATTAAACTTGGAATCGCCCCTTTTCATCTGTGACTACC

AGACGTCATACAAGGGTTAAACCTAACAACATGCCTAATCCTTGCAACATGACAAAAATT

AGCCCCAATATCCTTAATAATTATAACCAATCACCAATTAAACACAAACCTGCTAATTAC

AATAGCCGTATTATCAGCAGTAATTGGAGGATGAGGGGGACTTAACCAAACACAAATCCG

AAAAATAATAGCATATTCATCTATCGCCCACCTTGGGTGAATAACACTAGTCCTCTCTTT

CTCCCCAAATTTAACAACCCTTAACCTAATAATCTACCTAATATTAACATCATCAATGTT

CTTAATAATAACAACCCTAAACTCAACAAATATTAACAAACTTTCAACATCTTGACTGAA

AACCCCACTACTAGCATCATCAATAATAGTTACACTCATGGCCCTAGGAGGCCTACCCCC

AACATCAGGATTCCTACCAAAATGACTTATCCTACAAGAAATAACCAAACAGCACCTAGG

GGCCGTGTCCACACTAATAGCTATATCTGCACTACTAAGCCTATTCTTCTACTTACGACT

ATCATATGCAATCTCACTAACTACCGCACCAAACACATCAAACTCCAACATAACCTGACG

AATAACCCACAACCAAATACAAATCTTACCAACAATAATTACACTAACTACAATAATACT

ACCGATCACACCAACACTACTAGCAATAGGACAAGGCAGCTTGGCGTACCCCGGTCTGAG

GACCCACGGGACTTTGGAGAGCATAGGTGGGCCTAAAGGCAGTTCAAGAGGAGGGTTGCC

CTCACTAGCAAACACTTTTGAACAAGTCATTGAGGAGATGCTTGAAGATGAGCAGAGCAT

GAGGCCCAGTGAGGAGAACAAGGATGCGGACATGTTTACATCTCGCGTAATGCTGAGCAG

TCAAGTGCCTTTGGAGCCTCCTCTTCTCTTTCTGCTGGAGGAGTACAAAAACTACTTGGA

TGCTGCAAATATGTCCATGAGGGTCCGACGTCACTCTGACCCCGCCCGCCGCGGGGAGCT

GAGTGTGTGTGACAGTATTAGTGAGTGGGTGACGGCATCTGACAAAAAGACTGCCGTGGA

CATGTCGGGGCAGACGGTAACTGTTCTAGAAAAAGTTCCAGTACCCAAAGGCCAACTGAA

GCAATACTTCTACGAGACCAAATGCAACCCTATGGGTTACATGAAGGAGGGCTGCAGAGG

CATTGACAAGAGGTACTGGAACTCCCAGTGCCGAACTACTCAGTCGTATGTGCGCGCGCT

CACCATGGATAACAAGAAGAAAGTGGGCTGGCGGTTTATCAGAATAGACACTTCATGTGT

ATGTACATTGACCATTGTTTGCTTGGGTTGCAGACCGAAGGCTGTTGTTCTACAAATATG

TCTACAAGCGATATAGAGCTGGGAAGCAAAGGGGTATGATTATAGAAACCGAAGGTGACA

GGCCTTCTTCAAAAGCTGACATTGAAATGGATGGAAAGATGGTCAACTCTCATACTGAAA

ATTTCATTGATGGCTCTCTAGTTCTGGAAGTCGATGAGAAAGATCAGGATGATGAAGAAG

CCAGGCGCGATATGGCCAGAATCCTAAAGGAGCTAAAGCAGAAACATCCTGACAAAGAAA

TAGAGCAACTGATTGAGTTAGCGAACTACCAGGTCTTGAGTCAACAGCAAAAAAGTCGAG

CATTCTATCGTATTCAAGCTACTCGCTTGATGACTGGGGCCGGAAATATTCTAAAGAGAC

ATGCAGCAGACCAGGCAAGGAAAGCTGTGAGCATGCAGGAAGTTAACTCTGAAGTGATCG

AAAATGAGCCCGTCAGTAAGATCTATTTCGAGCAAGC

>X108

CAACGGCCGCGGTATCATGACCGTGCAAAGGTAGCGTAATCACTTGTCTTTTAAATAAAG

ACCCGTATGAAAGGCAAAACGAAAGTTCAACTGTCTCTTTAATCCAATCAATGAAATTTA

TCTTTTCGTGCAGAGGCGGAGATAAATATATAAGACGAGAAGACCCTATGGAGCTTCAAA

CACACTATAATATAAGTTTTAGGTTGGGGCGACCACGGAGCAAAAAAATCCTCCGAGATA

AGAATAACACTTCAAAAATTAAAACATTTTGATCCACTAAGTGACCAACGAACCAAGTTA

CCCTAGGGATAACAGCGCAATCCTTTCTAAGAGTCCATATCGACGAATGGGTTTACGACC

TCGATGTTGGATCAGGACACCCAAATGGTGCAGCCGCTGTTAAGGTTCGTTTGTTCAACG

ATTAAAGTCCTACATGAACCCATATGCACTCTCAATTCTATTATCAAGCCTAGCAGTGGG

AACAATTACAACTCTTTCAAGCTCCCACTGATTCCTGGCATGACTAGGCTTAGAAATTAA

CACATTAGCAATCATCCCACTAATAACAAAAATACACCACCCACGAGCAACGGAATCGGC

CACAAAATACTTCTTAACGCAAGCCACCGCATCAGCCCTAATCTTATTTTCTACAATCAC

AAACGCATGGACCACAGGAGAATGAACAATTATTACCATAAATGACAACACATCAACACT

TATCTTAACAATCGCCCTAGCCATTAAACTGGGAATCGCCCCATTTCACCTGTGATTACC

AGATGTCATACAAGGACTAAACCTAGCAACATGTTTAATCCTTACAACATGACAAAAACT

AGCCCCAATATCATTAATAATTATAACCAGCCATCAATTAAACACAAACCTACTAATTAC

AATAGCCATATTATCAACAGTAATTGGGGGGTGGGGGGGCCTGAACCAAACGCAAATACG

AAAAATAATAGCATATTCATCTATTGCCCACCTCGGATGAATAACACTTATCCTGTCTTT

CTCCCCAAACTTAACAACCCTAAACCTAATAATTTACCTAATACTAACCTCATCAATATT

TTTAATAATAATAACCTTAACCTCAACAAATATAAACAAACTTTCAACATCTTGACTGAA

AACGCCACTACTAGCATCATCAATAATAATTACACTCATAGCCCTGGGAGGCCTACCCCC

AACCTCAGGCTTCTTACCAAAATGACTAATCCTACAAGAAATAACTAAACAACACCTAGG

AGCCGCATCTACACTAATAGCTATATCTGCCCTACTAAGCCTATTCTTCTACCTACGACT

ATCATATACAATCTCACTAACCACTGCACCAAACACCTCAAACTCCAACATAACCTGACG

AGTAGCCCACAACCAAATACAAGTCCTCCCAGCAATAATTACACTAACTATAATAATACT

ACCAATCACACCAACACTACTAGCAACAGGACAAGGCAGCTTGGCGTACCCAGGTCTGAG

GACCCACGGGACTTTGGAGAGCATAGGTGGGCCTAAAGGCAGTTCAAGAGGAGGGTTGCC

CTCACTAGCAGACACTTTTGAACAAGTCATTGAGGAGATGCTTGAAGATGAGCAGAGCAT

GAGGCCCAGTGAGGAGAACAAGGATGCGGACATGTTTACATCTCGCGTAATGCTGAGCAG

TCAAGTGCCTTTGGAGCCTCCTCTTCTCTTTCTGCTGGAGGAGTACAAAAACTACTTGGA

TGCTGCAAATATGTCCATGAGGGTCCGACGTCACTCTGACCCCGCCCGCCGCGGGGAGCT

GAGTGTGTGTGACAGTATTAGTGAGTGGGTGACGGCATCTGACAAAAAGACTGCCGTGGA

CATGTCGGGGCAGACGGTAACTGTTCTAGAAAAAGTTCCAGTACCCAAAGGCCAACTGAA

GCAATACTTCTACGAGACCAAATGCAACCCTATGGGTTACATGAAGGAGGGCTGCAGAGG

CATTGACAAGAGGTACTGGAACTCCCAGTGCCGAACTACTCAGTCGTATGTGCGCGCGCT

CACCATGGATAACAAGAAGAAAGTGGGCTGGCGGTTTATCAGAATAGACACTTCATGTGT

ATGTACATTGACCATTGTTTGCTTGGGTTGCAGACCGAAGGCTGTTGTTCTACAAATATG

TCTACAAGCGATATAGAGCTGGGAAGCAAAGGGGTATGATTATAGAAACCGAAGGTGACA

GGCCTTCTTCAAAAGCTGACATTGAAATGGATGGAAAGATGGTCAACTCTCATACTGAAA

ATTTCATTGATGGCTCTCTAGTTCTGGAAGTCGATGAGAAAGATCAGGATGATGAAGAAG

CCAGGCGCGATATGGCCAGAATCCTAAAGGAGCTAAAGCAGAAACATCCTGACAAGGAAA

TAGAGCAACTGATTGAGTTAGCGAACTACCAGGTCTTGAGTCAACAGCAAAAAAGTCGAG

CATTCTATCGTATTCAAGCTACTCGCTTGATGACTGGGGCCGGAAATATTCTAAAGAGAC

ATGCAGCAGACCAGGCAAGGAAAGCTGTGAGCATGCAGGAAGTTAACTCTGAAGTGATCG

AAAATGAGCCCGTCAGTAAGATCTATTTCGAGCAAGC

>X109

CAACGGCCGCGGTATCATGACCGTGCAAAGGTAGCGTAATCACTTGTCTTTTAAATAAAG

ACCCGTATGAAAGGCAAAACGAAAGTTCAACTGTCTCTTTAATCCAATCAATGAAATTTA

TCTTTTCGTGCAGAGGCGGAGATAAATATATAAGACGAGAAGACCCTATGGAGCTTCAAA

CACACTATAATATAAGTTTTAGGTTGGGGCGACCACGGAGCAAAAAAATCCTCCGAGATA

AGAATAACACTTCAAAAATTAAAACATTTTGATCCACTAAGTGACCAACGAACCAAGTTA

CCCTAGGGATAACAGCGCAATCCTTTCTAAGAGTCCATATCGACGAATGGGTTTACGACC

TCGATGTTGGATCAGGACACCCAAATGGTGCAGCCGCTGTTAAGGTTCGTTTGTTCAACG

ATTAAAGTCCTACATGAACCCATATGCACTCTCAATTCTATTATCAAGCCTAGCAGTGGG

AACAATTACAACTCTTTCAAGCTCCCACTGATTCCTGGCATGACTAGGCTTAGAAATTAA

CACATTAGCAATCATCCCACTAATAACAAAAATACACCACCCACGAGCAACGGAATCGGC

CACAAAATACTTCTTAACGCAAGCCACCGCATCAGCCCTAATCTTATTTTCTACAATCAC

AAACGCATGGACCACAGGAGAATGAACAATTATTACCATAAATGACAACACATCAACACT

TATCTTAACAATCGCCCTAGCCATTAAACTGGGAATCGCCCCATTTCACCTGTGATTACC

AGATGTCATACAAGGACTAAACCTAGCAACATGTTTAATCCTTACAACATGACAAAAACT

AGCCCCAATATCATTAATAATTATAACCAGCCATCAATTAAACACAAACCTACTAATTAC

AATAGCCATATTATCAACAGTAATTGGGGGGTGGGGGGGCCTGAACCAAACGCAAATACG

AAAAATAATAGCATATTCATCTATTGCCCACCTCGGATGAATAACACTTATCCTGTCTTT

CTCCCCAAACTTAACAACCCTAAACCTAATAATTTACCTAATACTAACCTCATCAATATT

TTTAATAATAATAACCTTAACCTCAACAAATATAAACAAACTTTCAACATCTTGACTGAA

AACGCCACTACTAGCATCATCAATAATAATTACACTCATAGCCCTGGGAGGCCTACCCCC

AACCTCAGGCTTCTTACCAAAATGACTAATCCTACAAGAAATAACTAAACAACACCTAGG

AGCCGCATCTACACTAATAGCTATATCTGCCCTACTAAGCCTATTCTTTGACCTACGACT

ATCATATACAATCTCACTAACCACTGCACCAAACACCTCAAACTCCAACATAACCTGACG

AGTAGCCCACAACCAAATACAAGTCCTCCCAGCAATAATTACACTAACTATAATAATACT

ACCAATCACACCAACACTACTAGCAACAGGACAAGGCAGCTTGGCGTACCCAGGTCTGAG

GACCCACGGGACTTTGGAGAGCATAGGTGGGCCTAAAGGCAGTTCAAGAGGAGGGTTGCC

CTCACTAGCAGACACTTTTGAACAAGTCATTGAGGAGATGCTTGAAGATGAGCAGAGCAT

GAGGCCCAGTGAGGAGAACAAGGATGCGGACATGTTTACATCTCGTGTAATGCTGAGCAG

TCAAGTGCCTTTGGAGCCTCCTCTTCTCTTTCTGCTGGAGGAGTACAAAAACTACTTGGA

TGCTGCAAATATGTCCATGAGGGTCCGACGTCACTCTGACCCCGCCCGCCGCGGGGAGCT

GAGTGTGTGTGACAGTATTAGTGAGTGGGTGACGGCATCTGACAAAAAGACTGCCGTGGA

CATGTCGGGGCAGACGGTAACTGTTCTAGAAAAAGTTCCAGTACCCAAAGGCCAACTGAA

GCAATACTTCTACGAGACCAAATGCAACCCTATGGGTTACATGAAGGAGGGCTGCAGAGG

CATTGACAAGAGGTACTGGAACTCCCAGTGCCGAACTACTCAGTCGTATGTGCGCGCGCT

CACCATGGATAACAAGAAGAAAGTGGGCTGGCGGTTTATCAGAATAGACACTTCATGTGT

ATGTACATTGACCATTGTTTGCTTGGGTTGCAGACCGAAGGCTGTTGTTCTACAAATATG

TCTACAAGCGATATAGAGCTGGGAAGCAAAGGGGTATGATTATAGAAACCGAAGGTGACA

GGCCTTCTTCAAAAGCTGACATTGAAATGGATGGAAAGATGGTCAACTCTCATACTGAAA

ATTTCATTGATGGCTCTCTAGTTCTGGAAGTCGATGAGAAAGATCAGGATGATGAAGAAG

CCAGGCGCGATATGGCCAGAATCCTAAAGGAGCTAAAGCAGAAACATCCTGACAAGGAAA

TAGAGCAACTGATTGAGTTAGCGAACTACCAGGTCTTGAGTCAACAGCAAAAAAGTCGAG

CATTCTATCGTATTCAAGCTACTCGCTTGATGACTGGGGCCGGAAATATTCTAAAGAGAC

ATGCAGCAGACCAGGCAAGGAAAGCTGTGAGCATGCAGGAAGTTAACTCTGAAGTGATCG

AAAATGAGCCCGTCAGTAAGATCTATTTCGAGCAAGC

>X110

CAACGGCCGCGGTATCATGACCGTGCAAAGGTAGCGTAATCACTTGTCTTTTAAATAAAG

ACCCGTATGAAAGGCAAAACGAAAGTTCAACTGTCTCTTTAATCCAATCAATGAAATTTA

TCTTTTCGTGCAGAGGCGGAGATAAATATATAAGACGAGAAGACCCTATGGAGCTTCAAA

CACACTATAATATAAGTTTTAGGTTGGGGCGACCACGGAGCAAAAAAATCCTCCGAGATA

AGAATAACACTTCAAAAATTAAAACATTTTGATCCACTAAGTGACCAACGAACCAAGTTA

CCCTAGGGATAACAGCGCAATCCTTTCTAAGAGTCCATATCGACGAATGGGTTTACGACC

TCGATGTTGGATCAGGACACCCAAATGGTGCAGCCGCTGTTAAGGTTCGTTTGTTCAACG

ATTAAAGTCCTACATGAACCCATATGCACTCTCAATTCTATTATCAAGCCTAGCAGTGGG

AACAATTACAACTCTTTCAAGCTCCCACTGATTCCTGGCATGACTAGGCTTAGAAATTAA

CACATTAGCAATCATCCCACTAATAACAAAAATACACCACCCACGAGCAACGGAATCGGC

CACAAAATACTTCTTAACGCAAGCCACCGCATCAGCCCTAATCTTATTTTCTACAATCAC

AAACGCATGGACCACAGGAGAATGAACAATTATTACCATAAATGACAACACATCAACACT

TATCTTAACAATCGCCCTAGCCATTAAACTGGGAATCGCCCCATTTCACCTGTGATTACC

AGATGTCATACAAGGACTAAACCTAGCAACATGTTTAATCCTTACAACATGACAAAAACT

AGCCCCAATATCATTAATAATTATAACCAGCCATCAATTAAACACAAACCTACTAATTAC

AATAGCCATATTATCAACAGTAATTGGGGGGTGGGGGGGCCTGAACCAAACGCAAATACG

AAAAATAATAGCATATTCATCTATTGCCCACCTCGGATGAATAACACTTATCCTGTCTTT

CTCCCCAAACTTAACAACCCTAAACCTAATAATTTACCTAATACTAACCTCATCAATATT

TTTAATAATAATAACCTTAACCTCAACAAATATAAACAAACTTTCAACATCTTGACTGAA

AACGCCACTACTAGCATCATCAATAATAATTACACTCATAGCCCTGGGAGGCCTACCCCC

AACCTCAGGCTTCTTACCAAAATGACTAATCCTACAAGAAATAACTAAACAACACCTAGG

AGCCGCATCTACACTAATAGCTATATCTGCCCTACTAAGCCTATTCTTTGACCTACGACT

ATCATATACAATCTCACTAACCACTGCACCAAACACCTCAAACTCCAACATAACCTGACG

AGTAGCCCACAACCAAATACAAGTCCTCCCAGCAATAATTACACTAACTATAATAATACT

ACCAATCACACCAACACTACTAGCAACAGGACAAGGCAGCTTGGCGTACCCAGGTCTGAG

GACCCACGGGACTTTGGAGAGCATAGGTGGGCCTAAAGGCAGTTCAAGAGGAGGGTTGCC

CTCACTAGCAGACACTTTTGAACAAGTCATTGAGGAGATGCTTGAAGATGAGCAGAGCAT

GAGGCCCAGTGAGGAGAACAAGGATGCGGACATGTTTACATCTCGCGTAATGCTGAGCAG

TCAAGTGCCTTTGGAGCCTCCTCTTCTCTTTCTGCTGGAGGAGTACAAAAACTACTTGGA

TGCTGCAAATATGTCCATGAGGGTCCGACGTCACTCTGACCCCGCCCGCCGCGGGGAGCT

GAGTGTGTGTGACAGTATTAGTGAGTGGGTGACGGCATCTGACAAAAAGACTGCCGTGGA

CATGTCGGGGCAGACGGTAACTGTTCTAGAAAAAGTTCCAGTACCCAAAGGCCAACTGAA

GCAATACTTCTACGAGACCAAATGCAACCCTATGGGTTACATGAAGGAGGGCTGCAGAGG

CATTGACAAGAGGTACTGGAACTCCCAGTGCCGAACTACTCAGTCGTATGTGCGCGCGCT

CACCATGGATAACAAGAAGAAAGTGGGCTGGCGGTTTATCAGAATAGACACTTCATGTGT

ATGTACATTGACCATTGTTTGCTTGGGTTGCAGACCGAAGGCTGTTGTTCTACAAATATG

TCTACAAGCGATATAGAGCTGGGAAGCAAAGGGGTATGATTATAGAAACCGAAGGTGACA

GGCCTTCTTCAAAAGCTGACATTGAAATGGATGGAAAGATGGTCAACTCTCATACTGAAA

ATTTCATTGATGGCTCTCTAGTTCTGGAAGTCGATGAGAAAGATCAGGATGATGAAGAAG

CCAGGCGCGATATGGCCAGAATCCTAAAGGAGCTAAAGCAGAAACATCCTGACAAGGAAA

TAGAGCAACTGATTGAGTTAGCGAACTACCAGGTCTTGAGTCAACAGCAAAAAAGTCGAG

CATTCTATCGTATTCAAGCTACTCGCTTGATGACTGGGGCCGGAAATATTCTAAAGAGAC

ATGCAGCAGACCAGGCAAGGAAAGCTGTGAGCATGCAGGAAGTTAACTCTGAAGTGATCG

AAAATGAGCCCGTCAGTAAGATCTATTTCGAGCAAGC

>JA246

CAACGGCCGCGGTATCATGACCGTGCAAAGGTAGCGTAATCACTTGTCTTTTAAATAAAG

ACCTGTATGAAAGGCAAAACGAAAGTTCAACTGTCTCTTTAATCCAATCAATGAAATTTA

TCTTTTCGTGCAGAAGCGAAAATAAATATATAAGACGAGAAGACCCTATGGAGCTTTAAA

CACACTATAATATAAGTTTTAGGTTGGGGCGACCACGGAGAAAAGAAATCCTCCGAGATA

AGAGCAACACCTCGAAAATTAAAACATTTTGACCCACTAAGTGACCAACGAACCAAGTTA

CCCTAGGGATAACAGCGCAATCCTTTCTAAGAGTCCATATCGACGAATGGGTTTACGACC

TCGATGTTGGATCAGGACACCCAAATGGTGCAGCCGCTATTAAGGTTCGTTTGTTCAACG

ATTAAAGTCCTACATGAACCCATATGCACTATCAATCCTGTTGTCGGGCCTAGCACTAGG

AACAATCACAACTCTCTCAAGCTCCCATTGATTCCTAGCATGAGTAGGACTAGAAATTAA

TACACTAGCAATAATTCCACTAATAACAAAAATACACCACCCACGGTCAACAGAATCAGC

CACAAAATACTTCCTAACGCAAGCCACCGCATCGGCCTTAATCCTATTTTCTACAATCAC

AAACGCATGAGCCACAGGAGAATGAACAACTACCACCATAAATGACAACACATCAACACT

TATATTAACAATTGCCCTAGCTATTAAACTTGGAATCGCCCCTTTTCATCTATGACTACC

AGACGTCATACAAGGGTTAAATATAACAACATGCCTAATCCTTGCAACATGACAAAAATT

AGCCCCAATGTCTCTAATAATTATAACCAACCACCAATTAAACACAAACATGCTAATTAC

AATAGCCGTGTTGTCAACAGTAATTGGGGGGTGGGGGGGCCTTAACCAAACACAAACCCG

AAAAATAATAGCATACTCATCTATCGCCCACCTCGGATGAATAACACTAGTCCTATCTTT

CTCCCCAGATTTAACAACCCTTAACCTAATAATCTACCTAATACTAACATCATCAATATT

CTTAATAATAACAACCCTAAACTCAACAAATATTAACAAACTTTCAATATCTTGACTGAA

AGCCCCCCTACTAACATCATCAATAATAATCACACTAATGGCCCTAGGAGGCCTGCCCCC

AACATCAGGGTTCCTACCAAAATGGCTTATCCTACAAGAAATAACTAAACAACACCTAGC

AGCCGTATCCACACTAATAGCTATATCTGCACTACTAAGCCTATTCTTCTACCTACGACT

ATCATATGCAATCTCACTAACTACCACACCAAACACCTCAAACTCCAACATAACCTGACG

AATAACCCACAACCAAATACAAACCCTACCAACAATAATTACACTAACTACAATAATACT

ACCAATCACACCAACACTACTAGCAATAGGACAAGGCAGCTTGGCGTACCCAGGTCTGAG

GACCCACGGGACTTTGGAGAGCATAGGTGGGCCTAAAGGCAGTTCAAGAGGAGGGTTGCC

CTCACTAGCAGACACTTTTGAACAAGTCATTGAGGAGATGCTTGAAGATGAGCAGAGCAT

GAGGCCCAGTGAGGAGAACAAGGATGCGGACATGTTTACATCTCGCGTAATGCTGAGCAG

TCAAGTGCCTTTGGAGCCTCCTCTTCTCTTTCTGCTGGAGGAGTACAAAAACTACTTGGA

TGCTGCAAATATGTCCATGAGGGTCCGACGTCACTCTGACCCCGCCCGCCGCGGGGAGCT

CAGTGTGTGTGACAGTATTAGTGAGTGGGTGACGGCATCTGACAAAAAGACTGCCGTGGA

CATGTCGGGGCAGACGGTAACTGTTCTAGAAAAAGTTCCAGTACCCAAAGGCCAACTGAA

GCAATACTTCTACGAGACCAAATGCAACCCTATGGGTTACATGAAGGAGGGCTGCAGAGG

CATTGACAAGAGGTACTGGAACTCCCAGTGCCGAACTACTCAGTCGTATGTGCGCGCGCT

CACCATGGATAACAAGAAGAAAGTGGGCTGGCGGTTTATCAGAATAGACACTTCATGTGT

ATGTACATTGACCATTGTTTGCTTGGGTTGCAGACCGAAGGCTGTTGTTCTACAAATATG

TCTACAAGCGATATAGAGCTGGGAAGCAAAGGGGTATGATTATAGAAACCGAAGGTGACA

GGCCTTCTTCAAAAGCTGACATTGAAATGGATGGAAAGATGGTCAACTCTCATACTGAAA

ATTTCATTGATGGCTCTCTAGTTCTGGAAGTCGATGAGAAAGATCAGGATGATGAAGAAG

CCAGGCGCGATATGGCCAGAATCCTAAAGGAGCTAAAGCAGAAACATCCTGACAAGGAAA

TAGAGCAACTGATTGAGTTAGCGAACTACCAGGTCTTGAGTCAACAGCAAAAAAGTCGAG

CATTCTATCGTATTCAAGCTACTCGCTTGATGACTGGGGCCGGAAATATTCTAAAGAGAC

ATGCAGCAGACCAGGCAAGGAAAGCTGTGAGCATGCAGGAAGTTAACTCTGAAGTGATCG

AAAATGAGCCCGTCAGTAAGATCTATTTCGAGCAAGC

>JA284

CAACGGCCGCGGTATCATGACCGTGCAAAGGTAGCGTAATCACTTGTCTTTTAAATAAAG

ACCTGTATGAAAGGCAAAACGAAAGTTCAACTGTCTCTTTAATCCAATCAATGAAATTTA

TCTTTTCGTGCAGAAGCGAAAATAAATATATAAGACGAGAAGACCCTATGGAGCTTTAAA

CACACTATAATATAAGTTTTAGGTTGGGGCGACCACGGAGAAAAGAAATCCTCCGAGATA

AGAGCAACACCTCGAAAATTAAAACATTTTGACCCACTAAGTGACCAACGAACCAAGTTA

CCCTAGGGATAACAGCGCAATCCTTTCTAAGAGTCCATATCGACGAATGGGTTTACGACC

TCGATGTTGGATCAGGACACCCAAATGGTGCAGCCGCTATTAAGGTTCGTTTGTTCAACG

ATTAAAGTCCTACATGAACCCATATGCACTATCAATCCTGTTGTCGGGCCTAGCACTAGG

AACAATCACAACTCTCTCAAGCTCCCATTGATTCCTAGCATGAGTAGGACTAGAAATTAA

TACACTAGCAATAATTCCACTAATAACAAAAATACACCACCCACGGTCAACAGAATCAGC

CACAAAATACTTCCTAACGCAAGCCACCGCATCGGCCTTAATCCTATTTTCTACAATCAC

AAACGCATGAGCCACAGGAGAATGAACAACTACCACCATAAATGACAACACATCAACACT

TATATTAACAATTGCCCTAGCTATTAAACTTGGAATCGCCCCTTTTCATCTATGACTACC

AGACGTCATACAAGGGTTAAATATAACAACATGCCTAATCCTTGCAACATGACAAAAATT

AGCCCCAATGTCTCTAATAATTATAACCAACCACCAATTAAACACAAACATGCTAATTAC

AATAGCCGTGTTGTCAACAGTAATTGGGGGGTGAGGGGGCCTTAACCAAACACAAACCCG

AAAAATAATAGCATACTCATCTATCGCCCACCTCGGATGAATAACACTAGTCCTATCTTT

CTCCCCAGATTTAACAACCCTTAACCTAATAATCTACCTAATACTAACATCATCAATATT

CTTAATAATAACAACCCTAAACTCAACAAATATTAACAAACTTTCAATATCTTGACTGAA

AGCCCCCCTACTAACATCATCAATAATAATCACACTAATGGCCCTAGGAGGCCTGCCCCC

AACATCAGGGTTCCTACCAAAATGGCTTATCCTACAAGAAATAACTAAACAACACCTAGC

AGCCGTATCCACACTAATAGCTATATCTGCACTACTAAGCCTATTCTTCTACCTACGACT

ATCATATGCAATCTCACTAACTACCACACCAAACACCTCAAACTCCAACATAACCTGACG

AATAACCCACAACCAAATACAAACCCTACCAACAATAATTACACTAACTACAATAATACT

ACCAATCACACCAACACTACTAGCAATAGGACAAGGCAGCTTGGCGTACCCAGGTCTGAG

GACCCACGGGACTTTGGAGAGCATAGGTGGGCCTAAAGGCAGTTCAAGAGGAGGGTTGCC

CTCACTAGCAGACACTTTTGAACAAGTCATTGAGGAGATGCTTGAAGATGAGCAGAGCAT

GAGGCCCAGTGAGGAGAACAAGGATGCGGACATGTTTACATCTCGCGTAATGCTGAGCAG

TCAAGTGCCTTTGGAGCCTCCTCTTCTCTTTCTGCTGGAGGAGTACAAAAACTACTTGGA

TGCTGCAAATATGTCCATGAGGGTCCGACGTCACTCTGACCCCGCCCGCCGCGGGGAGCT

CAGTGTGTGTGACAGTATTAGTGAGTGGGTGACGGCATCTGACAAAAAGACTGCCGTGGA

CATGTCGGGGCAGACGGTAACTGTTCTAGAAAAAGTTCCAGTACCCAAAGGCCAACTGAA

GCAATACTTCTACGAGACCAAATGCAACCCTATGGGTTACATGAAGGAGGGCTGCAGAGG

CATTGACAAGAGGTACTGGAACTCCCAGTGCCGAACTACTCAGTCGTATGTGCGCGCGCT

CACCATGGATAACAAGAAGAAAGTGGGCTGGCGGTTTATCAGAATAGACACTTCATGTGT

ATGTACATTGACCATTGTTTGCTTGGGTTGCAGACCGAAGGCTGTTGTTCTACAAATATG

TCTACAAGCGATATAGAGCTGGGAAGCAAAGGGGTATGATTATAGAAACCGAAGGTGACA

GGCCTTCTTCAAAAGCTGACATTGAAATGGATGGAAAGATGGTCAACTCTCATACTGAAA

ATTTCATTGATGGCTCTCTAGTTCTGGAAGTCGATGAGAAAGATCAGGATGATGAAGAAG

CCAGGCGCGATATGGCCAGAATCCTAAAGGAGCTAAAGCAGAAACATCCTGACAAGGAAA

TAGAGCAACTGATTGAGTTAGCGAACTACCAGGTCTTGAGTCAACAGCAAAAAAGTCGAG

CATTCTATCGTATTCAAGCTACTCGCTTGATGACTGGGGCCGGAAATATTCTAAAGAGAC

ATGCAGCAGACCAGGCAAGGAAAGCTGTGAGCATGCAGGAAGTTAACTCTGAAGTGATCG

AAAATGAGCCCGTCAGTAAGATCTATTTCGAGCAAGC

>JA285

CAACGGCCGCGGTATCATGACCGTGCAAAGGTAGCGTAATCACTTGTCTTTTAAATAAAG

ACCTGTATGAAAGGCAAAACGAAAGTTCAACTGTCTCTTTAATCCAATCAATGAAATTTA

TCTTTTCGTGCAGAAGCGAAAATAAATATATAAGACGAGAAGACCCTATGGAGCTTTAAA

CACACTATAATATAAGTTTTAGGTTGGGGCGACCACGGAGAAAAGAAATCCTCCGAGATA

AGAGCAACACCTCGAAAATTAAAACATTTTGACCCACTAAGTGACCAACGAACCAAGTTA

CCCTAGGGATAACAGCGCAATCCTTTCTAAGAGTCCATATCGACGAATGGGTTTACGACC

TCGATGTTGGATCAGGACACCCAAATGGTGCAGCCGCTATTAAGGTTCGTTTGTTCAACG

ATTAAAGTCCTACATGAACCCATATGCACTATCAATCCTGTTGTCGGGCCTAGCACTAGG

AACAATCACAACTCTCTCAAGCTCCCATTGATTCCTAGCATGAGTAGGACTAGAAATTAA

TACACTAGCAATAATTCCACTAATAACAAAAATACACCACCCACGGTCAACAGAATCAGC

CACAAAATACTTCCTAACGCAAGCCACCGCATCGGCCTTAATCCTATTTTCTACAATCAC

AAACGCATGAGCCACAGGAGAATGAACAACTACCACCATAAATGACAACACATCAACACT

TATATTAACAATTGCCCTAGCTATTAAACTTGGAATCGCCCCTTTTCATCTATGACTACC

AGACGTCATACAAGGGTTAAATATAACAACATGCCTAATCCTTGCAACATGACAAAAATT

AGCCCCAATGTCTCTAATAATTATAACCAACCACCAATTAAACACAAACATGCTAATTAC

AATAGCCGTGTTGTCAACAGTAATTGGGGGGTGAGGGGGCCTTAACCAAACACAAACCCG

AAAAATAATAGCATACTCATCTATCGCCCACCTCGGATGAATAACACTAGTCCTATCTTT

CTCCCCAGATTTAACAACCCTTAACCTAATAATCTACCTAATACTAACATCATCAATATT

CTTAATAATAACAACCCTAAACTCAACAAATATTAACAAACTTTCAATATCTTGACTGAA

AGCCCCCCTACTAACATCATCAATAATAATCACACTAATGGCCCTAGGAGGCCTGCCCCC

AACATCAGGGTTCCTACCAAAATGGCTTATCCTACAAGAAATAACTAAACAACACCTAGC

AGCCGTATCCACACTAATAGCTATATCTGCACTACTAAGCCTATTCTTCTACCTACGACT

ATCATATGCAATCTCACTAACTACCACACCAAACACCTCAAACTCCAACATAACCTGACG

AATAACCCACAACCAAATACAAACCCTACCAACAATAATTACACTAACTACAATAATACT

ACCAATCACACCAACACTACTAGCAATAGGACAAGGCAGCTTGGCGTACCCAGGTCTGAG

GACCCACGGGACTTTGGAGAGCATAGGTGGGCCTAAAGGCAGTTCAAGAGGAGGGTTGCC

CTCACTAGCAGACACTTTTGAACAAGTCATTGAGGAGATGCTTGAAGATGAGCAGAGCAT

GAGGCCCAGTGAGGAGAACAAGGATGCGGACATGTTTACATCTCGCGTAATGCTGAGCAG

TCAAGTGCCTTTGGAGCCTCCTCTTCTCTTTCTGCTGGAGGAGTACAAAAACTACTTGGA

TGCTGCAAATATGTCCATGAGGGTCCGACGTCACTCTGACCCCGCCCGCCGCGGGGAGCT

CAGTGTGTGTGACAGTATTAGTGAGTGGGTGACGGCATCTGACAAAAAGACTGCCGTGGA

CATGTCGGGGCAGACGGTAACTGTTCTAGAAAAAGTTCCAGTACCCAAAGGCCAACTGAA

GCAATACTTCTACGAGACCAAATGCAACCCTATGGGTTACATGAAGGAGGGCTGCAGAGG

CATTGACAAGAGGTACTGGAACTCCCAGTGCCGAACTACTCAGTCGTATGTGCGCGCGCT

CACCATGGATAACAAGAAGAAAGTGGGCTGGCGGTTTATCAGAATAGACACTTCATGTGT

ATGTACATTGACCATTGTTTGCTTGGGTTGCAGACCGAAGGCTGTTGTTCTACAAATATG

TCTACAAGCGATATAGAGCTGGGAAGCAAAGGGGTATGATTATAGAAACCGAAGGTGACA

GGCCTTCTTCAAAAGCTGACATTGAAATGGATGGAAAGATGGTCAACTCTCATACTGAAA

ATTTCATTGATGGCTCTCTAGTTCTGGAAGTCGATGAGAAAGATCAGGATGATGAAGAAG

CCAGGCGCGATATGGCCAGAATCCTAAAGGAGCTAAAGCAGAAACATCCTGACAAGGAAA

TAGAGCAACTGATTGAGTTAGCGAACTACCAGGTCTTGAGTCAACAGCAAAAAAGTCGAG

CATTCTATCGTATTCAAGCTACTCGCTTGATGACTGGGGCCGGAAATATTCTAAAGAGAC

ATGCAGCAGACCAGGCAAGGAAAGCTGTGAGCATGCAGGAAGTTAACTCTGAAGTGATCG

AAAATGAGCCCGTCAGTAAGATCTATTTCGAGCAAGC

>LXX652

CAACGGCCGCGGTATCATGACCGTGCAAAGGTAGCGTAATCACTTGTCTTTTAAATAAAG

ACCTGTATGAAAGGCAAAACGAAAGTTCAACTGTCTCTTTAATCCAATCAATGAAATTTA

TCTTTTCGTGCAGAAGCGGAAATGAATATATAAGACGAGAAGACCCTATGGAGCTTTAAA

CACGCTATAATACAAGTTTTTGGTTGGGGCGACCGCGGAGAAAAGAAATCCTCCGAGATA

AGAATAACACTTCAAAAATTAAAAAATTTTGATCCACTAAGTGACCAACGAACCAAGTTA

CCCTAGGGATAACAGCGCAATCCTTTCTAAGAGTCCATATCGACGAATGGGTTTACGACC

TCGATGTTGGATCAGGACACCCAAATGGTGCAGCCGCTATTAAGGTTCGTTTGTTCAACG

ATTAAAGTCCTACATGAACCCGTATGCACTATCAATCCTGTTGTCGAGCCTAGCAGTAGG

AACAATTACAACTCTCTCAAGCTCCCATTGATTTCTGGCATGAGTAGGACTAGAAATTAA

CACACTAGCAATAATTCCCCTAATAACAAAAATACACCACCCACGATCAACAGAATCAGC

CACAAAATACTTCCTAACGCAAGCCACCGCATCGGCCTTAATCTTATTTTCTACAATCAT

AAACGCATGGACTACAGGAGAATGAACAATTATCACCATAAATGATAACTCATCAACATT

TATATTAACAGTTGCCCTAGCTATTAAACTTGGAATCGCCCCTTTTCATCTGTGACTACC

AGACGTCATACAAGGGTTAAACCTAACAACATGCCTAATCCTTGCAACATGACAAAAATT

AGCCCCAATATCCTTAATAATTATAACCAATCACCAATTAAACACAAACCTGCTAATTAC

AATAGCCGTATTATCAGCAGTAATTGGAGGATGAGGGGGACTTAACCAAACACAAATCCG

AAAAATAATAGCATATTCATCTATCGCCCACCTTGGGTGAATAACACTAGTCCTCTCTTA

CTCCCCAAATTTAACAGCCCTTAACCTAATAATCTACCTAATACTAACATCATCAATGTT

CTTAATAATAACAACCCTAAACTCAACAAATATTAACAAACTTTCAACATCTTGACTGAA

AACCCCACTACTAGCATCATCAATAATAGTCACACTCATGGCCCTAGGAGGCCTACCCCC

AACATCAGGATTCCTACCAAAATGACTTATCCTACAAGAAATAACCAAACAGCACCTAGG

GGCCGTGTCCACACTAATAGCTATATCTGCACTACTAAGCCTATTCTTCTACTTACGACT

ATCATATGCAATCTCACTAACTACCGCACCAAACACATCAAACTCCAACATAACCTGACG

AATAACCCACAACCAAATACAAATCTTACCAACAATAATTATACTAACTACAATAATACT

ACCAATCACACCAACACTACTAGCAATAGGACAAGGCAGCTTGGCGTACCCAGGTCTGAG

GACCCACGGGACTCTGGAGAGCATAGGTGGGCCTAAAGGCAATTCAAGAGGAGGGTTGCC

CTCACTAGCAGACACTTTTGAACAAGTCATTGAGGAGATGCTTGAAGATGAGCAGAGCAT

GAGGCCCAGTGAGGAGAACAAGGATGCGGACATGTTTACATCTCGCGTAATGCTGAGCAG

TCAAGTGCCTTTGGAGCCTCCTCTTCTCTTTCTGCTGGAGGAGTACAAAAACTACTTGGA

TGCTGCAAATATGTCCATGAGGGTCCGACGTCACTCTGACCCCGCACGCCGCGGGGAGCT

GAGTGTGTGTGACAGTATTAGTGAGTGGGTGACGGCATCTGACAAAAAGACTGCCGTGGA

CATGTCGGGGCAGACGGTAACTGTTCTAGAAAAAGTTCCAGTACCCAAAGGCCAACTGAA

GCAATACTTCTACGAGACCAAATGCAACCCTATGGGTTACATGAAGGAGGGCTGCAGAGG

CATTGACAAGAGGTACTGGAACTCCCAGTGCCGAACTACTCAGTCGTATGTGCGCGCGCT

CACCATGGATAACAAGAAGAAAGTGGGCTGGCGGTTTATCAGAATAGACACTTCATGTGT

ATGTACATTGACCATTGTTTGCTTGGGTTGCAGACCGAAGGCTGTTGTTCTACAAATATG

TCTACAAGCGATATAGAGCTGGGAAGCAAAGGGGTATGATTATAGAAACCGAAGGTGACA

GGCCTTCTTCAAAAGCTGACATTGAAATGGATGGAAAGATGGTCAACTCTCATACTGAAA

ATTTCATTGATGGCTCTCTAGTTCTGGAAGTCGATGAGAAAGATCAGGATGATGAAGAAG

CCAGGCGCGATATGGCCAGAATCCTAAAGGAGCTAAAGCAGAAACATCCTGACAAGGAAA

TAGAGCAACTGATTGAGTTAGCGAACTACCAGGTCTTGAGTCAACAGCAAAAAAGTCGAG

CATTCTATCGTATTCAAGCTACTCGCTTGATGACTGGGGCCGGAAATATTCTAAAGAGAC

ATGCAGCAGACCAGGCAAGGAAAGCTGTGAGCATGCAGGAAGTTAACTCTGAAGTGATCG

AAAATGAGCCCGTCAGTAAGATCTATTTCGAGCAAGC

>LXX653

CAACGGCCGCGGTATCATGACCGTGCAAAGGTAGCGTAATCACTTGTCTTTTAAATAAAG

ACCTGTATGAAAGGCAAAACGAAAGTTCAACTGTCTCTTTAATCCAATCAATGAAATTTA

TCTTTTCGTGCAGAAGCGGAAATGAATATATAAGACGAGAAGACCCTATGGAGCTTTAAA

CACGCTATAATACAAGTTTTTGGTTGGGGCGACCGCGGAGAAAAGAAATCCTCCGAGATA

AGAATAACACTTCGAAAATTAAAAAATTTTGATCCACTAAGTGACCAACGAACCAAGTTA

CCCTAGGGATAACAGCGCAATCCTTTCTAAGAGTCCATATCGACGAATGGGTTTACGACC

TCGATGTTGGATCAGGACACCCAAATGGTGCAGCCGCTATTAAGGTTCGTTTGTTCAACG

ATTAAAGTCCTACATGAACCCGTATGCACTATCAATCCTGTTGTCGAGCCTAGCAGTAGG

AACAATTACAACTCTCTCAAGCTCCCATTGATTTCTGGCATGAGTAGGACTAGAAATTAA

TACACTAGCAATAATCCCCCTAATAACAAAAATACACCACCCACGATCAACAGAATCAGC

CACAAAATACTTCCTAACGCAAGCCACCGCATCGGCCTTAATCTTATTTTCTACAATCAT

AAACGCATGGACTACAGGAGAATGAACAATTATCACCATAAATGATAACTCATCAACATT

TATATTAACAGTTGCCCTAGCTATTAAACTTGGAATCGCCCCTTTTCATCTGTGACTACC

AGACGTCATACAAGGGTTAAACCTAACAACATGCCTAATCCTTGCAACATGACAAAAATT

AGCCCCAATATCCTTAATAATTATAACCAATCACCAATTAAACACAAACCTGCTAATTAC

AATAGCCGTATTATCAGCAGTAATTGGAGGATGAGGGGGACTTAACCAAACACAAATCCG

AAAAATAATAGCATATTCATCTATCGCCCACCTTGGGTGAATAACACTAGTCCTCTCTTT

CTCCCCAAATTTAACAACCCTTAACCTAATAATCTACCTAATACTAACATCATCAATGTT

CTTAATAATAACAACCCTAAACTCAACAAATATTAACAAACTTTCAACATCTTGACTGAA

AACCCCACTACTAGCATCATCAATAATAGTCACACTCATGGCCCTAGGAGGCCTACCCCC

AACATCAGGATTCCTACCAAAATGACTCATCCTACAAGAAATAACCAAACAGCACCTAGG

GGCCGTGTCCACACTAATAGCTATATCTGCACTACTAAGCCTATTCTTCTACTTACGACT

ATCATATGCAATCTCACTAACTACCGCACCAAACACATCAAACTCCAACATAACCTGACG

AATAACCCACAACCAAATACAAATCTTACCAACAATAATTATACTAACTACAATAATACT

ACCAATCACACCAACACTACTAGCAATAGGACAAGGCAGCTTGGCGTACCCAGGTCTGAG

GACCCACGGGACTCTGGAGAGCATAGGTGGGCCTAAAGGCAGTTCAAGAGGAGGGTTGCC

CTCACTAGCAGACACTTTTGAACAAGTCATTGAGGAGATGCTTGAAGATGAGCAGAGCAT

GAGGCCCAGTGAGGAGAACAAGGATGCGGACATGTTTACATCTCGCGTAATGCTGAGCAG

TCAAGTGCCTTTGGAGCCTCCTCTTCTCTTTCTGCTGGAGGAGTACAAAAACTACTTGGA

TGCTGCAAATATGTCCATGAGGGTCCGACGTCACTCTGACCCCGCCCGCCGCGGGGAGCT

GAGTGTGTGTGACAGTATTAGTGAGTGGGTGACGGCATCTGACAAAAAGACTGCCGTGGA

CATGTCGGGGCAGACGGTAACTGTTCTAGAAAAAGTTCCAGTACCCAAAGGCCAACTGAA

GCAATACTTCTACGAGACCAAATGCAACCCTATGGGTTACATGAAGGAGGGCTGCAGAGG

CATTGACAAGAGGTACTGGAACTCCCAGTGCCGAACTACTCAGTCGTATGTGCGCGCGCT

CACCATGGATAACAAGAAGAAAGTGGGCTGGCGGTTTATCAGAATAGACACTTCATGTGT

ATGTACATTGACCATTGTTTGCTTGGGTTGCAGACCGAAGGCTGTTGTTCTACAAATATG

TCTACAAGCGATATAGAGCTGGGAAGCAAAGGGGTATGATTATAGAAACCGAAGGTGACA

GGCCTTCTTCAAAAGCTGACATTGAAATGGATGGAAAGATGGTCAACTCTCATACTGAAA

ATTTCATTGATGGCTCTCTAGTTCTGGAAGTCGATGAGAAAGATCAGGATGATGAAGAAG

CCAGGCGCGATATGGCCAGAATCCTAAAGGAGCTAAAGCAGAAACATCCTGACAAGGAAA

TAGAGCAACTGATTGAGTTAGCGAACTACCAGGTCTTGAGTCAACAGCAAAAAAGTCGAG

CATTCTATCGTATTCAAGCTACTCGCTTGATGACTGGGGCCGGAAATATTCTAAAGAGAC

ATGCAGCAGACCAGGCAAGGAAAGCTGTGAGCATGCAGGAAGTTAACTCTGAAGTGATCG

AAAATGAGCCCGTCAGTAAGATCTATTTCGAGCAAGC

>LXX654

CAACGGCCGCGGTATCATGACCGTGCAAAGGTAGCGTAATCACTTGTCTTTTAAATAAAG

ACCTGTATGAAAGGCAAAACGAAAGTTCAACTGTCTCTTTAATCCAATCAATGAAATTTA

TCTTTTCGTGCAGAAGCGGAAATGAATATATAAGACGAGAAGACCCTATGGAGCTTTAAA

CACGCTATAATACAAGTTTTTGGTTGGGGCGACCGCGGAGAAAAGAAATCCTCCGAGATA

AGAATAACACTTCGAAAATTAAAAAATTTTGATCCACTAAGTGACCAACGAACCAAGTTA

CCCTAGGGATAACAGCGCAATCCTTTCTAAGAGTCCATATCGACGAATGGGTTTACGACC

TCGATGTTGGATCAGGACACCCAAATGGTGCAGCCGCTATTAAGGTTCGTTTGTTCAACG

ATTAAAGTCCTACATGAACCCGTATGCATTATCAATCCTGTTGTCGAGCCTAGCAGTAGG

AACAATTACAACTCTCTCAAGCTCCCATTGATTTCTGGCATGAGTAGGACTAGAAATTAA

TACACTAGCAATAATCCCCCTAATAACAAAAATACACCACCCACGATCAACAGAATCAGC

CACAAAATACTTCCTAACGCAAGCCACCGCATCGGCCTTAATCTTATTTTCTATAATCAT

AAACGCATGGACTACAGGAGAATGAACAATTATCACCATAAATGATAACTCATCAACATT

TATATTAACAGTTGCCCTAGCTATTAAACTTGGAATCGCCCCTTTTCATCTGTGACTACC

AGACGTCATACAAGGGTTAAACCTAACAACATGCCTAATCCTTGCAACATGACAAAAATT

AGCCCCAATATCCTTAATAATTATAACCAATCACCAATTAAACACAAACCTGCTAATTAC

AATAGCCGTATTATCAGCAGTAATTGGAGGATGAGGGGGACTTAACCAAACACAAATCCG

AAAAATAATAGCATATTCATCTATCGCCCACCTTGGGTGAATAACACTAGTCCTCTCTTT

CTCCCCAAATTTAACAACCCTTAACCTAATAATCTACCTAATACTAACATCATCAATGTT

CTTAATAATAACAACCCTAAACTCAACAAATATTAACAAACTTTCAACATCTTGACTGAA

AACCCCACTACTAGCATCATCAATAATAGTCACACTCATAGCCCTAGGAGGCCTACCCCC

AACATCAGGATTCCTACCAAAATGACTTATCCTACAAGAAATAACCAAACAGCACCTAGG

GGCCGTGTCCACACTAATAGCTATATCTGCACTACTAAGCCTATTCTTCTACTTACGACT

ATCATATGCAATCTCACTAACTACCGCACCAAACACATCAAACTCCAACATAACCTGACG

AATAACCCATAACCAAATACAAATCTTACCAACAATAATTATACTAACTACAATAATACT

ACCAATCACACCAACACTACTAGCAATAGGACAAGGCAGCTTGGCGTACCCAGGTCTGAG

GACCCACGGGACTTTGGAGAGCATAGGTGGGCCTAAAGGCAGTTCAAGAGGAGGGTTGCC

CTCACTAGCAGACACTTTTGAACAAGTCATTGAGGAGATGCTTGAAGATGAGCAGAGCAT

GAGGCCCAGTGAGGAGAACAAGGATGCGGACATGTTTACATCTCGCGTAATGCTGAGCAG

TCAAGTGCCTTTGGAGCCTCCTCTTCTCTTTCTGCTGGAGGAGTACAAAAACTACTTGGA

TGCTGCAAATATGTCCATGAGGGTCCGACGTCACTCTGACCCCGCCCGCCGCGGGGAGCT

GAGTGTGTGTGACAGTATTAGTGAGTGGGTGACGGCATCTGACAAAAAGACTGCCGTGGA

CATGTCGGGGCAGACGGTAACTGTTCTAGAAAAAGTTCCAGTACCCAAAGGCCAACTGAA

GCAATACTTCTACGAGACCAAATGCAACCCTATGGGTTACATGAAGGAGGGCTGCAGAGG

CATTGACAAGAGGTACTGGAACTCCCAGTGCCGAACTACTCAGTCGTATGTGCGCGCGCT

CACCATGGATAACAAGAAGAAAGTGGGCTGGCGGTTTATCAGAATAGACACTTCATGTGT

ATGTACATTGACCATTGTTTGCTTGGGTTGCAGACCGAAGGCTGTTGTTCTACAAATATG

TCTACAAGCGATATAGAGCTGGGAAGCAAAGGGGTATGATTATAGAAACCGAAGGTGACA

GGCCTTCTTCAAAAGCTGACATTGAAATGGATGGAAAGATGGTCAACTCTCATACTGAAA

ATTTCATTGATGGCTCTCTAGTTCTGGAAGTCGATGAGAAAGATCAGGATGATGAAGAAG

CCAGGCGCGATATGGCCAGAATCCTAAAGGAGCTAAAGCAGAAACATCCTGACAAGGAAA

TAGAGCAACTGATTGAGTTAGCGAACTACCAGGTCTTGAGTCAACAGCAAAAAAGTCGAG

CATTCTATCGTATTCAAGCTACTCGCTTGATGACTGGGGCCGGAAATATTCTAAAGAGAC

ATGCAGCAGACCAGGCAAGGAAAGCTGTGAGCATGCAGGAAGTTAACTCTGAAGTGATCG

AAAATGAGCCCGTCAGTAAGATCTATTTCGAGCAAGC

>LXX656

CAACGGCCGCGGTATCATGACCGTGCAAAGGTAGCGTAATCACTTGTCTTTTAAATAAAG

ACCTGTATGAAAGGCAAAACGAAAGTTCAACTGTCTCTTTAATCCAATCAATGAAATTTA

TCTTTTCGTGCAGAAGCGGAAATGAATATATAAGACGAGAAGACCCTATGGAGCTTTAAA

CACGCTATAATACAAGTTTTTGGTTGGGGCGACCGCGGAGAAAAGAAATCCTCCGAGATA

AGAATAACACTTCGAAAATTAAAAAATTTTGATCCACTAAGTGACCAACGAACCAAGTTA

CCCTAGGGATAACAGCGCAATCCTTTCTAAGAGTCCATATCGACGAATGGGTTTACGACC

TCGATGTTGGATCAGGACACCCAAATGGTGCAGCCGCTATTAAGGTTCGTTTGTTCAACG

ATTAAAGTCCTACATGAACCCGTATGCACTATCAATCCTGTTGTCGAGCCTAGCAGTAGG

AACAATTACAACTCTCTCAAGCTCCCATTGATTTCTGGCATGAGTAGGACTAGAAATTAA

TACACTAGCAATAATCCCCCTAATAACAAAAATACACCACCCACGATCAACAGAATCAGC

CACAAAATACTTCCTAACGCAAGCCACCGCATCGGCCTTAATCTTATTTTCTACAATCAT

AAACGCATGGACTACAGGAGAATGAACAATTATCACCATAAATGATAACTCATCAACATT

TATATTAACAGTTGCCCTAGCTATTAAACTTGGAATCGCCCCTTTTCATCTGTGACTACC

AGACGTCATACAAGGGTTAAACCTAACAACATGCCTAATCCTTGCAACATGACAAAAATT

AGCCCCAATATCCTTAATAATTATAACCAATCACCAATTAAACACAAACCTGCTAATTAC

AATAGCCGTATTATCAGCAGTAATTGGAGGATGAGGGGGACTTAACCAAACACAAATCCG

AAAAATAATAGCATATTCATCTATCGCCCACCTTGGGTGAATAACACTAGTCCTCTCTTT

CTCCCCAAATTTAACAACCCTTAGCCTAATAATCTACCTAATACTAACATCATCAATGTT

CTTAATAATAACAACCCTAAACTCAACAAATATTAACAAACTTTCAACATCTTGACTGAA

AACCCCACTACTAGCATCATCAATAATAGTCACACTCATGGCCCTAGGAGGCCTACCCCC

AACATCAGGATTCCTACCAAAATGACTTATCCTACAAGAAATAACCAAACAGCACCTAGG

GGCCGTGTCCACACTAATAGCTATATCTGCACTACTAAGCCTATTCTTCTACTTACGACT

ATCATATGCAATCTCACTAACTACCGCACCAAACACATCAAACTCCAACATAACCTGACG

AATAACCCACAACCAAATACAAATCTTACCAACAATAATTATACTAACTACAATAATACT

ACCAATCACACCAACACTACTAGCAATAGGACAAGGCAGCTTGGCGTACCCAGGTCTGAG

GACCCACGGGACTTTGGAGAGCATAGGTGGGCCTAAAGGCAGTTCAAGAGGAGGGTTGCC

CTCACTAGCAGACACTTTTGAACAAGTCATTGAGGAGATGCTTGAAGATGAGCAGAGCAT

GAGGCCCAGTGAGGAGAACAAGGATGCGGACATGTTTACATCTCGCGTAATGCTGAGCAG

TCAAGTGCCTTTGGAGCCTCCTCTTCTCTTTCTGCTGGAGGAGTACAAAAACTACTTGGA

TGCTGCAAATATGTCCATGAGGGTCCGACGTCACTCTGACCCCGCCCGCCGCGGGGAGCT

GAGTGTGTGTGACAGTATTAGTGAGTGGGTGACGGCATCTGACAAAAAGACTGCCGTGGA

CATGTCGGGGCAGACGGTAACTGTTCTAGAAAAAGTTCCAGTACCCAAAGGCCAACTGAA

GCAATACTTCTACGAGACCAAATGCAACCCTATGGGTTACATGAAGGAGGGCTGCAGAGG

CATTGACAAGAGGTACTGGAACTCCCAGTGCCGAACTACTCAGTCGTATGTGCGCGCGCT

CACCATGGATAACAAGAAGAAAGTGGGCTGGCGGTTTATCAGAATAGACACTTCATGTGT

ATGTACATTGACCATTGTTTGCTTGGGTTGCAGACCGAAGGCTGTTGTTCTACAAATATG

TCTACAAGCGATATAGAGCTGGGAAGCAAAGGGGTATGATTATAGAAACCGAAGGTGACA

GGCCTTCTTCAAAAGCTGACATTGAAATGGATGGAAAGATGGTCAACTCTCATACTGAAA

ATTTCATTGATGGCTCTCTAGTTCTGGAAGTCGATGAGAAAGATCAGGATGATGAAGAAG

CCAGGCGCGATATGGCCAGAATCCTAAAGGAGCTAAAGCAGAAACATCCTGACAAGGAAA

TAGAGCAACTGATTGAGTTAGCGAACTACCAGGTCTTGAGTCAACAGCAAAAAAGTCGAG

CATTCTATCGTATTCAAGCTACTCGCTTGATGACTGGGGCCGGAAATATTCTAAAGAGAC

ATGCAGCAGACCAGGCAAGGAAAGCTGTGAGCATGCAGGAAGTTAACTCTGAAGTGATCG

AAAATGAGCCCGTCAGTAAGATCTATTTCGAGCAAGC

>NN3

CAACGGCCGCGGTATCATGACCGTGCAAAGGTAGCGTAATCACTTGTCTTTTAAATAAAG

ACCTGTATGAAAGGCAAAACGAAAGTTCAACTGTCTCTTTAATCCAATCAATGAAATTTA

TCTTTTCGTGCAGAAGCGGAAATAAATATATAAGACGAGAAGACCCTATGGAGCTTTAAA

CACACTATAATACAAGTTTTGGGTTGGGGCGACCGCGGAGAAAAAAAATCCTCCGAGATA

AGAATAACACTTCGAAAATTAAAATATTTTGATCCACTAAGTGATCAACGAACCAAGTTA

CCCTAGGGATAACAGCGCAATCCTTTCTAAGAGTCCATATCGACGAATGGGTTTACGACC

TCGATGTTGGATCAGGACACCCAAATGGTGCAGCCGCTATTAAGGTTCGTTTGTTCAACG

ATTAAAGTCCTACATGAACCCACATGCGCTATCAATCCTACTATCGAGCCTGGCAGTAGG

AACAATTACAACTCTCTCAAGCTCCCATTGATTCTTAGCATGAGTGGGCCTAGAAATTAA

TACACTAGCAATTATCCCACTAATAACAAAAATACACCACCCACGAGCAACAGAATCAGC

CACAAAATACTTCCTAACGCAGGCCACCGCATCAGCCTTAATCTTATTTTCTACCATCAC

AAACGCATGGGCTACAGGAGAATGAACAATTATTACCATAAATGACAACACATCAACATT

TATATTAACAATTGCCCTAGCCATTAAACTTGGAATCGCCCCATTTCATCTATGACTACC

AGACGTCATACAAGGGTTAAGCCTAACAACATGCCTAATCCTTGCAACATGACAAAAATT

AGCCCCAATATCCTTAATAATTATAACTAGCCATCAGTTAAACACAAACCTGCTAATTAC

AATAGCCGCGTTATCAACAGTTGTTGGGGGGTGGGGGGGCCTTAACCAAACACAAATGCG

AAAAATAATAGCATACTCATCTATCGCCCACCTCGGATGAATAACACTAATCCTATCTTT

CTCCCCAAATTTAACAACCCTAAACCTAATAATTTACCTAATATTAACATCATCAATATT

CTTAATGATAACAACCCTAAATTCAACAAATATTAACAAACTTTCAACATCTTGACTAAA

AACCCCCCTACTAGCATCATCAATAATAATTACACTTATAGCCCTAGGAGGGCTGCCCCC

AACATCAGGATTCCTACCAAAATGACTCATCCTACAAGAAATAACCAAACAACACCTAGG

AGCCGTATCCACACTAATAGCTATATCTGCCCTACTAAGCCTATTCTTCTACCTACGACT

ATCATATGCAATCTCACTAACTACTACACCAAACACTTCAAACTCCAACATAACCTGACG

AATAACCCACAACCAAATACAAATCCTACCAACAATAATTACACTAACTACAATAATATT

ACCAATTACACCAACACTACTAGCAATAGGACAAGGCAGCTTGGCGTACCCAGGTCTGAG

GACCCACGGGACTTTGGAGAGCATAGGTGGGCCTAAAGGCAGTTCAAGAGGAGGGTTGCC

CTCACTAGCAGACACTTTTGAACAAGTCATTGAGGAGATGCTTGAAGATGAGCAGAGCAT

GAGGCCCAGTGAGGAGAACAAGGATGCGGACATGTTTACATCTCGCGTAATGCTGAGCAG

TCAAGTGCCTTTGGAGCCTCCTCTTCTCTTTCTGCTGGAGGAGTACAAAAACTACTTGGA

TGCTGCAAATATGTCCATGAGGGTCCGACGTCACTCTGACCCCGCCCGCCGCGGGGAGCT

GAGTGTGTGTGACAGTATTAGTGAGTGGGTGACGGCATCTGACAAAAAGACTGCCGTGGA

CATGTCGGGGCAGACGGTAACTGTTCTAGAAAAAGTTCCAGTACCCAAAGGCCAACTGAA

GCAATACTTCTACGAGACCAAATGCAACCCTATGGGTTACATGAAGGAGGGCTGCAGAGG

CATTGACAAGAGGTACTGGAACTCCCAGTGCCGAACTACTCAGTCGTATGTGCGCGCGCT

CACCATGGATAACAAGAAGAAAGTGGGCTGGCGGTTTATCAGAATAGACACTTCATGTGT

ATGTACATTGACCATTGTTTGCTTGGGTTGCAGACCGAAGGCTGTTGTTCTACAAATATG

TCTACAAGCGATATAGAGCTGGGAAGCAAAGGGGTATGATTATAGAAACCGAAGGTGACA

GGCCTTCTTCAAAAGCTGACATTGAAATGGATGGAAAGATGGTCAACTCTCATACTGAAA

ATTTCATTGATGGCTCTCTAGTTCTGGAAGTCGATGAGAAAGATCAGGATGATGAAGAAG

CCAGGCGCGATATGGCCAGAATCCTAAAGGAGCTAAAGCAGAAACATCCTGACAAGGAAA

TAGAGCAACTGATTGAGTTAGCGAACTACCAGGTCTTGAGTCAACAGCAAAAAAGTCGAG

CATTCTATCGTATTCAAGCTACTCGGTTGATGACTGGGGCCGGAAATATTCTAAAGAGAC

ATGCAGCAGACCAGGCAAGGAAAGCTGTGAGCATGCAGGAAGTTAACTCTGAAGTGATCG

AAAATGAGCCCGTCAGTAAGATCTATTTCGAGCAAGC

>NN7

CAACGGCCGCGGTATCATGACCGTGCAAAGGTAGCGTAATCACTTGTCTTTTAAATAAAG

ACCTGTATGAAAGGCAAAACGAAAGTTCAACTGTCTCTTTAATCCAATCAATGAAATTTA

TCTTTTCGTGCAGAAGCGGAAATAAATATATAAGACGAGAAGACCCTATGGAGCTTTAAA

CACACTATAATACAAGTTTTGGGTTGGGGCGACCGCGGAGAAAAAAAATCCTCCGAGATA

AGAATAACACTTCGAAAATTAAAATATTTTGATCCACTAAGTGATCAACGAACCAAGTTA

CCCTAGGGATAACAGCGCAATCCTTTCTAAGAGTCCATATCGACGAATGGGTTTACGACC

TCGATGTTGGATCAGGACACCCAAATGGTGCAGCCGCTATTAAGGTTCGTTTGTTCAACG

ATTAAAGTCCTACATGAACCCACATGCGCTATCAATCCTACTATCGAGCCTGGCAGTAGG

AACAATTACAACTCTCTCAAGCTCCCATTGATTCTTAGCATGAGTGGGCCTAGAAATTAA

TACACTAGCAATTATCCCACTAATAACAAAAATACACCACCCACGAGCAACAGAATCAGC

CACAAAATACTTCCTAACGCAGGCCACCGCATCAGCCTTAATCTTATTTTCTACCATCAC

AAACGCATGGGCTACAGGAGAATGAACAATTATTACCATAAATGACAACACATCAACATT

TATATTAACAATTGCCCTAGCCATTAAACTTGGAATCGCCCCATTTCATCTATGATTACC

AGACGTCATACAAGGGTTAAGCCTAACAACATGCCTAATCCTTGCAACATGACAAAAATT

AGCCCCAATATCCTTAATAATTATAACTGGCCATCAGTTAAACACAAACCTGCTAATTAC

AATAGCCGCGTTATCAACAGTTGTTGGGGGGTGGGGGGGCCTTAACCAAACACAAATGCG

AAAAATAATAGCATACTCATCTATCGCCCACCTCGGATGAATAACACTAATCCTATCTTT

CTCCCCAAATTTAACAACCCTAAACCTAATAATTTACCTAATATTAACATCATCAATATT

CTTAATAATAACAACCCTAAATTCAACAAATATTAACAAACTTTCAACATCTTGACTAAA

AACCCCCCTACTAACATCATCAATAATAATTACACTTATAGCCCTAGGAGGGCTGCCCCC

AACATCAGGATTCCTACCAAAATGACTCATCCTACAAGAAATAACCAAACAACACCTAGG

AGCCGTATCCACACTAATAGCTATATCTGCTTTACTAAGCCTATTCTTCTACCTACGACT

ATCATATGCAATCTCACTAACTACTACACCAAACACTTCAAACTCCAACATAACCTGACG

AATAGCCCACAACCAAATACAAATCCTACCAACAATAATTACACTAACTACAATAATATT

ACCAATTACACCAACACTACTAGCAATAGGACAAGGCAGCTTGGCGTACCCAGGTCTGAG

GACCCACGGGACTTTGGAGAGCATAGGTGGGCCTAAAGGCAGTTCAAGAGGAGGGTTGCC

CTCACTAGCAGACACTTTTGAACAAGTCATTGAGGAGATGCTTGAAGATGAGCAGAGCAT

GAGGCCCAGTGAGGAGAACAAGGATGCGGACATGTTTACATCTCGCGTAATGCTGAGCAG

TCAAGTGCCTTTGGAGCCTCCTCTTCTCTTTCTGCTGGAGGAGTACAAAAACTACTTGGA

TGCTGCAAATATGTCCATGAGGGTCCGACGTCACTCTGACCCCGCCCGCCGCGGGGAGCT

GAGTGTGTGTGACAGTATTAGTGAGTGGGTGACGGCATCTGACAAAAAGACTGCCGTGGA

CATGTCGGGGCAGACGGTAACTGTTCTAGAAAAAGTTCCAGTACCCAAAGGCCAACTGAA

GCAATACTTCTACGAGACCAAATGCAACCCTATGGGTTACATGAAGGAGGGCTGCAGAGG

CATTGACAAGAGGTACTGGAACTCCCAGTGCCGAACTACTCAGTCGTATGTGCGCGCGCT

CACCATGGATAACAAGAAGAAAGTGGGCTGGCGGTTTATCAGAATAGACACTTCATGTGT

ATGTACATTGACCATTGTTTGCTTGGGTTGCAGACCGAAGGCTGTTGTTCTACAAATATG

TCTACAAGCGATATAGAGCTGGGAAGCAAAGGGGTATGATTATAGAAACCGAAGGTGACA

GGCCTTCTTCAAAAGCTGACATTGAAATGGATGGAAAGATGGTCAACTCTCATACTGAAA

ATTTCATTGATGGCTCTCTAGTTCTGGAAGTCGATGAGAAAGATCAGGATGATGAAGAAG

CCAGGCGCGATATGGCCAGAATCCTAAAGGAGCTAAAGCAGAAACATCCTGACAAGGAAA

TAGAGCAACTGATTGAGTTAGCGAACTACCAGGTCTTGAGTCAACAGCAAAAAAGTCGAG

CATTCTATCGTATTCAAGCTACTCGCTTGATGACTGGGGCCGGAAATATTCTAAAGAGAC

ATGCAGCAGACCAGGCAAGGAAAGCTGTGAGCATGCAGGAAGTTAACTCTGAAGTGATCG

AAAATGAGCCCGTCAGTAAGATCTATTTCGAGCAAGC

>NN12

CAACGGCCGCGGTATCATGACCGTGCAAAGGTAGCGTAATCACTTGTCTTTTAAATAAAG

ACCTGTATGAAAGGCAAAACGAAAGTTCAACTGTCTCTTTAATCCAATCAATGAAATTTA

TCTTTTCGTGCAGAAGCGGAAATAAATATATAAGACGAGAAGACCCTATGGAGCTTTAAA

CACACTATAATACAAGTTTTGGGTTGGGGCGACCGCGGAGAAAAAAAATCCTCCGAGATA

AGAATAACACTTCGAAAATTAAAATATTTTGATCCACTAAGTGATCAACGAACCAAGTTA

CCCTAGGGATAACAGCGCAATCCTTTCTAAGAGTCCATATCGACGAATGGGTTTACGACC

TCGATGTTGGATCAGGACACCCAAATGGTGCAGCCGCTATTAAGGTTCGTTTGTTCAACG

ATTAAAGTCCTACATGAACCCACATGCGCTATCAATCCTACTATCGAGCCTGGCAGTAGG

AACAATTACAACTCTCTCAAGCTCCCATTGATTCTTAGCATGAGTGGGCCTAGAAATTAA

TACACTAGCAATTATCCCACTAATAACAAAAATACACCACCCACGAGCAACAGAATCAGC

CACAAAATACTTCCTAACGCAGGCCACCGCATCAGCCTTAATCTTATTTTCTACCATCAC

AAACGCATGGGCTACAGGAGAATGAACAATTATTACCATAAATGACAACACATCAACATT

TATATTAACAATTGCCCTAGCCATTAAACTTGGAATCGCCCCATTTCATCTATGATTACC

AGACGTCATACAAGGGTTAAGCCTAACAACATGCCTAATCCTTGCAACATGACAAAAATT

AGCCCCAATATCCTTAATAATTATAACTGGCCATCAGTTAAACACAAACCTGCTAATTAC

AATAGCCGCGTTATCAACAGTTGTTGGGGGGTGGGGGGGCCTTAACCAAACACAAATGCG

AAAAATAATAGCATACTCATCTATCGCCCACCTCGGATGAATAACACTAATCCTATCTTT

CTCCCCAAATTTAACAACCCTAAACCTAATAATTTACCTAATATTAACATCATCAATATT

CTTAATAATAACAACCCTAAATTCAACAAATATTAACAAACTTTCAACATCTTGACTAAA

AACCCCCCTACTAGCATCATCAATAATAATTACACTTATAGCCCTAGGAGGGCTGCCCCC

AACATCAGGATTCCTACCAAAATGACTCATCCTACAAGAAATAACCAAACAACACCTAGG

AGCCGTATCCACACTAATAGCTATATCTGCTTTACTAAGCCTATTCTTCTACCTACGACT

ATCATATGCAATCTCACTAACTACTACACCAAACACTTCAAACTCCAACATAACCTGACG

AATAGCCCACAACCAAATACAAATCCTACCAACAATAATTACACTAACTACAATAATATT

ACCAATTACACCAACACTACTAGCAATAGGACAAGGCAGCTTGGCGTACCCAGGTCTGAG

GACCCACGGGACTTTGGAGAGCATAGGTGGGCCTAAAGGCAGTTCAAGAGGAGGGTTGCC

CTCACTAGCAGACACTTTTGAACAAGTCATTGAGGAGATGCTTGAAGATGAGCAGAGCAT

GAGGCCCAGTGAGGAGAACAAGGATGCGGACATGTTTACATCTCGCGTAATGCTGAGCAG

TCAAGTGCCTTTGGAGCCTCCTCTTCTCTTTCTGCTGGAGGAGTACAAAAACTACTTGGA

TGCTGCAAATATGTCCATGAGGGTCCGACGTCACTCTGACCCCGCCCGCCGCGGGGAGCT

GAGTGTGTGTGACAGTATTAGTGAGTGGGTGACGGCATCTGACAAAAAGACTGCCGTGGA

CATGTCGGGGCAGACGGTAACTGTTCTAGAAAAAGTTCCAGTACCCAAAGGCCAACTGAA

GCAATACTTCTACGAGACCAAATGCAACCCTATGGGTTACATGAAGGAGGGCTGCAGAGG

CATTGACAAGAGGTACTGGAACTCCCAGTGCCGAACTACTCAGTCGTATGTGCGCGCGCT

CACCATGGATAACAAGAAGAAAGTGGGCTGGCGGTTTATCAGAATAGACACTTCATGTGT

ATGTACATTGACCATTGTTTGCTTGGGTTGCAGACCGAAGGCTGTTGTTCTACAAATATG

TCTACAAGCGATATAGAGCTGGGAAGCAAAGGGGTATGATTATAGAAACCGAAGGTGACA

GGCCTTCTTCAAAAGCTGACATTGAAATGGATGGAAAGATGGTCAACTCTCATACTGAAA

ATTTCATTGATGGCTCTCTAGTTCTGGAAGTCGATGAGAAAGATCAGGATGATGAAGAAG

CCAGGCGCGATATGGCCAGAATCCTAAAGGAGCTAAAGCAGAAACATCCTGACAAGGAAA

TAGAGCAACTGATTGAGTTAGCGAACTACCAGGTCTTGAGTCAACAGCAAAAAAGTCGAG

CATTCTATCGTATTCAAGCTACTCGGTTGATGACTGGGGCCGGAAATATTCTAAAGAGAC

ATGCAGCAGACCAGGCAAGGAAAGCTGTGAGCATGCAGGAAGTTAACTCTGAAGTGATCG

AAAATGAGCCCGTCAGTAAGATCTATTTCGAGCAAGC

>qp1080

CAACGGCCGCGGTATCATGACCGTGCAAAGGTAGCGTAATCACTTGTCTTTTAAATAAAG

ACCCGTATGAAAGGCAAAACGAAAGTTCAACTGTCTCTTTAATCCAATCAATGAAATTTA

TCTTTTCGTGCAGAGGCGGAAATAAATATATAAGACGAGAAGACCCTATGGAGCTTCAAA

CACACTATAATATAAGTTTTGGGTTGGGGCGACCACGGAGCAAAAAAATCCTCCGAGATA

AGAGTAACACTTCGAAAATTAAAACATTTTGATCCACTAAGTGACCAACGAACCAAGTTA

CCCTAGGGATAACAGCGCAATCCTTTCTAAGAGTCCATATCGACGAATGGGTTTACGACC

TCGATGTTGGATCAGGACACCCAAATGGTGCAGCCGCTGTTAAGGTTCGTTTGTTCAACG

ATTAAAGTCCTACATGAACCCATATGCACTCTCAATTCTACTATCAAGCCTAGCAGTAGG

AACAATTACAACTCTTTCAAGCTCCCACTGATTCCTCGCATGACTAGGCTTAGAAATTAA

CACATTAGCAATCATCCCGCTGATAACAAAAATACACCACCCGCGAGCAACAGAATCGGC

CACAAAATACTTCCTGACGCAAGCCACCGCATCAGCCCTAATCTTATTTTCTACAATCAC

AAACGCATGAGCCACAGGAGAATGAACAATTATCACCATAAATGACAACACATCAACACT

TATCTTAACAATCGCCCTAGCCATTAAACTGGGAATCGCCCCATTTCACCTGTGATTACC

AGACGTCATACAAGGACTAAACCTAGCAACATGTTTAATCCTTACAACATGACAAAAACT

AGCCCCAATATCATTAATGATTATAACTAGCCATCAATTAAACACAAACCTACTAATTAC

AATAGCCATACTGTCAACAGTAATTGGGGGGTGGGGGGGCCTTAACCAAACACAAATACG

AAAAATAATAGCATATTCATCTATTGCCCACCTCGGATGAATAACACTTATCCTATCTTT

CTCCCCAAACTTAACAACCCTAAACCTAATAATTTACCTAATACTAACCTCATCAATATT

TTTAATAATAATAACCTTAACCTCAACAAATATAAACAAACTTTCAACATCTTGACTGAA

AACACCAATACTAGCATCATCAATAATAGTTACACTCATAGCCCTAGGAGGCCTACCCCC

AACCTCAGGCTTCTTACCAAAATGACTAATCCTACAAGAAATAACTAAACAACACCTAGG

AGCCGTATCTACACTAATAGCTATATCTGCCCTACTAAGCCTATTCTTCTACCTACGACT

ATCATATGCAATCTCACTAACCACTGCACCAAACACCTCAAACTCCAACATAACCTGACG

AGTAGACCACAACCAAATACAAATTCTCCCAACAATAATTACACTAACTACAATAATACT

ACCAATCACACCAACACTACTAGCAACAGGACAAGGCAGCTTGGCGTACCCAGGTCTGAG

GACCCACGGGACTTTGGAGAGCATAGGTGGGCCTAAAGGCAGTTCAAGAGGAGGGTTGCC

CTCACTAGCAGACACTTTTGAACAAGTCATTGAGGAGATGCTTGAAGATGAGCAGAGCAT

GAGGCCCAGTGAGGAGAACAAGGATGCGGACATGTTTACATCTCGCGTAATGCTGAGCAG

TCAAGTGCCTTTGGAGCCTCCTCTTCTCTTTCTGCTGGAGGAGTACAAAAACTACTTGGA

TGCTGCAAATATGTCCATGAGGGTCCGACGTCACTCTGACCCCGCCCGCCGCGGGGAGCT

GAGTGTGTGTGACAGTATTAGTGAGTGGGTGACGGCATCTGACAAAAAGACTGCCGTGGA

CATGTCGGGGCAGACGGTAACTGTTCTAGAAAAAGTTCCAGTACCCAAAGGCCAACTGAA

GCAATACTTCTACGAGACCAAATGCAACCCTATGGGTTACATGAAGGAGGGCTGCAGAGG

CATTGACAAGAGGTACTGGAACTCCCAGTGCCGAACTACTCAGTCGTATGTGCGCGCGCT

CACCATGGATAACAAGAAGAAAGTGGGCTGGCGGTTTATCAGAATAGACACTTCATGTGT

ATGTACATTGACCATTGTTTGCTTGGGTTGCAGACCGAAGGCTGTTGTTCTACAAATATG

TCTACAAGCGATATAGAGCTGGGAAGCAAAGGGGTATGATTATAGAAACCGAAGGTGACA

GGCCTTCTTCAAAAGCTGACATTGAAATGGATGGAAAGATGGTCAACTCTCATACTGAAA

ATTTCATTGATGGCTCTCTAGTTCTGGAAGTCGATGAGAAAGATCAGGATGATGAAGAAG

CCAGGCGCGATATGGCCAGAATCCTAAAGGAGCTAAAGCAGAAACATCCTGACAAGGAAA

TAGAGCAACTGATTGAGTTAGCGAACTACCAGGTCTTGAGTCAACAGCAAAAAAGTCGAG

CATTCTATCGTATTCAAGCTACTCGCTTGATGACTGGGGCCGGAAATATTCTAAAGAGAC

ATGCAGCAGACCAGGCAAGGAAAGCTGTGAGCATGCAGGAAGTTAACTCTGAAGTGATCG

AAAATGAGCCCGTCAGTAAGATCTATTTCGAGCAAGC

>qp1081

CAACGGCCGCGGTATCATGACCGTGCAAAGGTAGCGTAATCACTTGTCTTTTAAATAAAG

ACCCGTATGAAAGGCAAAACGAAAGTTCAACTGTCTCTTTAATCCAATCAATGAAATTTA

TCTTTTCGTGCAGAGGCGGAAATAAATATATAAGACGAGAAGACCCTATGGAGCTTCAAA

CACACTATAATATAAGTTTTAGGTTGGGGCGACCACGGAGCAAAAAAATCCTCCGAGATA

AGAGTAACACTTCGAAAATTAAAACATTTTGATCCACTAAGTGACCAACGAACCAAGTTA

CCCTAGGGATAACAGCGCAATCCTTTCTAAGAGTCCATATCGACGAATGGGTTTACGACC

TCGATGTTGGATCAGGACACCCAAATGGTGCAGCCGCTGTTAAGGTTCGTTTGTTCAACG

ATTAAAGTCCTACATGAACCCATATGCACTCTCAATTCTACTATCAAGCCTAGCAGTAGG

AACAATTACAACTCTTTCAAGCTCCCACTGATTCCTCGCATGACTAGGCTTAGAAATTAA

CACATTAGCAATCATCCCGCTGATAACAAAAATACACCACCCGCGAGCAACAGAATCGGC

CACAAAATACTTCCTGACGCAAGCCACCGCATCAGCCCTAATCTTATTTTCTACAATCAC

AAACGCATGAGCCACAGGAGAATGAACAATTATCACCATAAATGACAACACATCAACACT

TATCTTAACAATCGCCCTAGCCATTAAACTGGGAATCGCCCCATTTCACCTGTGATTACC

AGACGTCATACAAGGACTAAACCTAGCAACATGTTTAATCCTTACAACATGACAAAAACT

AGCCCCAATATCATTAATGATTATAACTAGCCATCAATTAAACACAAACCTACTAATTAC

AATAGCCATACTGTCAACAGTAATTGGGGGGTGGGGGGGCCTTAACCAAACACAAATACG

AAAAATAATAGCATATTCATCTATTGCCCACCTCGGATGAATAACACTTATCCTATCTTT

CTCCCCAAACTTAACAACCCTAAACCTAATAATTTACCTAATACTAACCTCATCAATATT

TTTAATAATAATAACCTTAACCTCAACAAATATAAACAAACTTTCAACATCTTGACTAAA

AACACCAATACTAGCATCATCAATAATAGTTACACTCATAGCCCTAGGAGGCCTACCCCC

AACCTCAGGCTTCTTACCAAAATGACTAATCCTACAAGAAATAACTAAACAACACCTAGG

AGCCGTATCTACACTAATAGCTATATCTGCCCTACTAAGCCTATTCTTCTACCTACGACT

ATCATATGCAATCTCACTAACCACTGCACCAAACACCTCAAACTCCAACATAACCTGACG

AGTAGACCACAACCAAATACAAATTCTCCCAACAATAATTACACTAACTACAATAATACT

ACCAATCACACCAACACTACTAGCAACAGGACAAGGCAGCTTGGCGTACCCAGGTCTGAG

GACCCACGGGACTTTGGAGAGCATAGGTGGGCCTAAAGGCAGTTCAAGAGGAGGGTTGCC

CTCACTAGCAGACACTTTTGAACAAGTCATTGAGGAGATGCTTGAAGATGAGCAGAGCAT

GAGGCCCAGTGAGGAGAACAAGGATGCGGACATGTTTACATCTCGCGTAATGCTGAGCAG

TCAAGTGCCTTTGGAGCCTCCTCTTCTCTTTCTGCTGGAGGAGTACAAAAACTACTTGGA

TGCTGCAAATATGTCCATGAGGGTCCGACGTCACTCTGACCCCGCCCGCCGCGGGGAGCT

GAGTGTGTGTGACAGTATTAGTGAGTGGGTGACGGCATCTGACAAAAAGACTGCCGTGGA

CATGTCGGGGCAGACGGTAACTGTTCTAGAAAAAGTTCCAGTACCCAAAGGCCAACTGAA

GCAATACTTCTACGAGACCAAATGCAACCCTATGGGTTACATGAAGGAGGGCTGCAGAGG

CATTGACAAGAGGTACTGGAACTCCCAGTGCCGAACTACTCAGTCGTATGTGCGCGCGCT

CACCATGGATAACAAGAAGAAAGTGGGCTGGCGGTTTATCAGAATAGACACTTCATGTGT

ATGTACATTGACCATTGTTTGCTTGGGTTGCAGACCGAAGGCTGTTGTTCTACAAATATG

TCTACAAGCGATATAGAGCTGGGAAGCAAAGGGGTATGATTATAGAAACCGAAGGTGACA

GGCCTTCTTCAAAAGCTGACATTGAAATGGATGGAAAGATGGTCAACTCTCATACTGAAA

ATTTCATTGATGGCTCTCTAGTTCTGGAAGTCGATGAGAAAGATCAGGATGATGAAGAAG

CCAGGCGCGATATGGCCAGAATCCTAAAGGAGCTAAAGCAGAAACATCCTGACAAGGAAA

TAGAGCAACTGATTGAGTTAGCGAACTACCAGGTCTTGAGTCAACAGCAAAAAAGTCGAG

CATTCTATCGTATTCAAGCTACTCGCTTGATGACTGGGGCCGGAAATATTCTAAAGAGAC

ATGCAGCAGACCAGGCAAGGAAAGCTGTGAGCATGCAGGAAGTTAACTCTGAAGTGATCG

AAAATGAGCCCGTCAGTAAGATCTATTTCGAGCAAGC

>qp1082

CAACGGCCGCGGTATCATGACCGTGCAAAGGTAGCGTAATCACTTGTCTTTTAAATAAAG

ACCCGTATGAAAGGCAAAACGAAAGTTCAACTGTCTCTTTAATCCAATCAATGAAATTTA

TCTTTTCGTGCAGAGGCGGAAATAAATATATAAGACGAGAAGACCCTATGGAGCTTCAAA

CACACTATAATATAAGTTTTAGGTTGGGGCGACCACGGAGCAAAAAAATCCTCCGAGATA

AGAGTAACACTTCGAAAATTAAAACATTTTGATCCACTAAGTGACCAACGAACCAAGTTA

CCCTAGGGATAACAGCGCAATCCTTTCTAAGAGTCCATATCGACGAATGGGTTTACGACC

TCGATGTTGGATCAGGACACCCAAATGGTGCAGCCGCTGTTAAGGTTCGTTTGTTCAACG

ATTAAAGTCCTACATGAACCCATATGCACTCTCAATTCTACTATCAAGCCTAGCAGTAGG

AACAATTACAACTCTTTCAAGCTCCCACTGATTCCTCGCATGACTAGGCTTAGAAATTAA

CACATTAGCAATCATCCCGCTGATAACAAAAATACACCACCCGCGAGCAACAGAATCGGC

CACAAAATACTTCCTGACGCAAGCCACCGCATCAGCCCTAATCTTATTTTCTACAATCAC

AAACGCATGAGCCACAGGAGAATGAACAATTATCACCATAAATGACAACACATCAACACT

TATCTTAACAATCGCCCTAGCCATTAAACTGGGAATCGCCCCATTTCACCTGTGATTACC

AGACGTCATACAAGGACTAAACCTAGCAACATGTTTAATCCTTACAACATGACAAAAACT

AGCCCCAATATCATTAATGATTATAACTAGCCATCAATTAAACACAAACCTACTAATTAC

AATAGCCATACTGTCAACAGTAATTGGGGGGTGGGGGGGCCTTAACCAAACACAAATACG

AAAAATAATAGCATATTCATCTATTGCCCACCTCGGATGAATAACACTTATCCTATCTTT

CTCCCCAAACTTAACAACCCTAAACCTAATAATTTACCTAATACTAACCTCATCAATATT

TTTAATAATAATAACCTTAACCTCAACAAATATAAACAAACTTTCAACATCTTGACTAAA

AACACCAATACTAGCATCATCAATAATAGTTACACTCATAGCCCTAGGAGGCCTACCCCC

AACCTCAGGCTTCTTACCAAAATGACTAATCCTACAAGAAATAACTAAACAACACCTAGG

AGCCGTATCTACACTAATAGCTATATCTGCCCTACTAAGCCTATTCTTCTACCTACGACT

ATCATATGCAATCTCACTAACCACTGCACCAAACACCTCAAACTCCAACATAACCTGACG

AGTAGACCACAACCAAATACAAATTCTCCCAACAATAATTACACTAACTACAATAATACT

ACCAATCACACCAACACTACTAGCAACAGGACAAGGCAGCTTGGCGTACCCAGGTCTGAG

GACCCACGGGACTTTGGAGAGCATAGGTGGGCCTAAAGGCAGTTCAAGAGGAGGGTTGCC

CTCACTAGCAGACACTTTTGAACAAGTCATTGAGGAGATGCTTGAAGATGAGCAGAGCAT

GAGGCCCAGTGAGGAGAACAAGGATGCGGACATGTTTACATCTCGCGTAATGCTGAGCAG

TCAAGTGCCTTTGGAGCCTCCTCTTCTCTTTCTGCTGGAGGAGTACAAAAACTACTTGGA

TGCTGCAAATATGTCCATGAGGGTCCGACGTCACTCTGACCCCGCCCGCCGCGGGGAGCT

GAGTGTGTGTGACAGTATTAGTGAGTGGGTGACGGCATCTGACAAAAAGACTGCCGTGGA

CATGTCGGGGCAGACGGTAACTGTTCTAGAAAAAGTTCCAGTACCCAAAGGCCAACTGAA

GCAATACTTCTACGAGACCAAATGCAACCCTATGGGTTACATGAAGGAGGGCTGCAGAGG

CATTGACAAGAGGTACTGGAACTCCCAGTGCCGAACTACTCAGTCGTATGTGCGCGCGCT

CACCATGGATAACAAGAAGAAAGTGGGCTGGCGGTTTATCAGAATAGACACTTCATGTGT

ATGTACATTGACCATTGTTTGCTTGGGTTGCAGACCGAAGGCTGTTGTTCTACAAATATG

TCTACAAGCGATATAGAGCTGGGAAGCAAAGGGGTATGATTATAGAAACCGAAGGTGACA

GGCCTTCTTCAAAAGCTGACATTGAAATGGATGGAAAGATGGTCAACTCTCATACTGAAA

ATTTCATTGATGGCTCTCTAGTTCTGGAAGTCGATGAGAAAGATCAGGATGATGAAGAAG

CCAGGCGCGATATGGCCAGAATCCTAAAGGAGCTAAAGCAGAAACATCCTGACAAGGAAA

TAGAGCAACTGATTGAGTTAGCGAACTACCAGGTCTTGAGTCAACAGCAAAAAAGTCGAG

CATTCTATCGTATTCAAGCTACTCGCTTGATGACTGGGGCCGGAAATATTCTAAAGAGAC

ATGCAGCAGACCAGGCAAGGAAAGCTGTGAGCATGCAGGAAGTTAACTCTGAAGTGATCG

AAAATGAGCCCGTCAGTAAGATCTATTTCGAGCAAGC

>qp1086

CAACGGCCGCGGTATCATGACCGTGCAAAGGTAGCGTAATCACTTGTCTTTTAAATAAAG

ACCCGTATGAAAGGCAAAACGAAAGTTCAACTGTCTCTTTAATCCAATCAATGAAATTTA

TCTTTTCGTGCAGAGGCGGAAATAAATATATAAGACGAGAAGACCCTATGGAGCTTCAAA

CACACTATAATATAAGTTTTAGGTTGGGGCGACCACGGAGCAAAAAAATCCTCCGAGATA

AGAGTAACACTTCGAAAATTAAAACATTTTGATCCACTAAGTGACCAACGAACCAAGTTA

CCCTAGGGATAACAGCGCAATCCTTTCTAAGAGTCCATATCGACGAATGGGTTTACGACC

TCGATGTTGGATCAGGACACCCAAATGGTGCAGCCGCTGTTAAGGTTCGTTTGTTCAACG

ATTAAAGTCCTACATGAACCCATATGCACTCTCAATTCTACTATCAAGCCTAGCAGTAGG

AACAATTACAACTCTTTCAAGCTCCCACTGATTCCTCGCATGACTAGGCTTAGAAATTAA

CACATTAGCAATCATCCCGCTGATAACAAAAATACACCACCCGCGAGCAACAGAATCGGC

CACAAAATACTTCCTGACGCAAGCCACCGCATCAGCCCTAATCTTATTTTCTACAATCAC

AAACGCATGAGCCACAGGAGAATGAACAATTATCACCATAAATGACAACACATCAACACT

TATCTTAACAATCGCCCTAGCCATTAAACTGGGAATCGCCCCATTTCACCTGTGATTACC

AGACGTCATACAAGGACTAAACCTAGCAACATGTTTAATCCTTACAACATGACAAAAACT

AGCCCCAATATCATTAATGATTATAACTAGCCATCAATTAAACACAAACCTACTAATTAC

AATAGCCATACTGTCAACAGTAATTGGGGGGTGGGGGGGCCTTAACCAAACACAAATACG

AAAAATAATAGCATATTCATCTATTGCCCACCTCGGATGAATAACACTTATCCTATCTTT

CTCCCCAAACTTAACAACCCTAAACCTAATAATTTACCTAATACTAACCTCATCAATATT

TTTAATAATAATAACCTTAACCTCAACAAATATAAACAAACTTTCAACATCTTGACTAAA

AACACCAATACTAGCATCATCAATAATAGTTACACTCATAGCCCTAGGAGGCCTACCCCC

AACCTCAGGCTTCTTACCAAAATGACTAATCCTACAAGAAATAACTAAACAACACCTAGG

AGCCGTATCTACACTAATAGCTATATCTGCCCTACTAAGCCTATTCTTCTACCTACGACT

ATCATATGCAATCTCACTAACCACTGCACCAAACACCTCAAACTCCAACATAACCTGACG

AGTAGACCACAACCAAATACAAATTCTCCCAACAATAATTACACTAACTACAATAATACT

ACCAATCACACCAACACTACTAGCAACAGGACAAGGCAGCTTGGCGTACCCAGGTCTGAG

GACCCACGGGACTTTGGAGAGCATAGGTGGGCCTAAAGGCAGTTCAAGAGGAGGGTTGCC

CTCACTAGCAGACACTTTTGAACAAGTCATTGAGGAGATGCTTGAAGATGAGCAGAGCAT

GAGGCCCAGTGAGGAGAACAAGGATGCGGACATGTTTACATCTCGCGTAATGCTGAGCAG

TCAAGTGCCTTTGGAGCCTCCTCTTCTCTTTCTGCTGGAGGAGTACAAAAACTACTTGGA

TGCTGCAAATATGTCCATGAGGGTCCGACGTCACTCTGACCCCGCCCGCCGCGGGGAGCT

GAGTGTGTGTGACAGTATTAGTGAGTGGGTGACGGCATCTGACAAAAAGACTGCCGTGGA

CATGTCGGGGCAGACGGTAACTGTTCTAGAAAAAGTTCCAGTACCCAAAGGCCAACTGAA

GCAATACTTCTACGAGACCAAATGCAACCCTATGGGTTACATGAAGGAGGGCTGCAGAGG

CATTGACAAGAGGTACTGGAACTCCCAGTGCCGAACTACTCAGTCGTATGTGCGCGCGCT

CACCATGGATAACAAGAAGAAAGTGGGCTGGCGGTTTATCAGAATAGACACTTCATGTGT

ATGTACATTGACCATTGTTTGCTTGGGTTGCAGACCGAAGGCTGTTGTTCTACAAATATG

TCTACAAGCGATATAGAGCTGGGAAGCAAAGGGGTATGATTATAGAAACCGAAGGTGACA

GGCCTTCTTCAAAAGCTGACATTGAAATGGATGGAAAGATGGTCAACTCTCATACTGAAA

ATTTCATTGATGGCTCTCTAGTTCTGGAAGTCGATGAGAAAGATCAGGATGATGAAGAAG

CCAGGCGCGATATGGCCAGAATCCTAAAGGAGCTAAAGCAGAAACATCCTGACAAGGAAA

TAGAGCAACTGATTGAGTTAGCGAACTACCAGGTCTTGAGTCAACAGCAAAAAAGTCGAG

CATTCTATCGTATTCAAGCTACTCGCTTGATGACTGGGGCCGGAAATATTCTAAAGAGAC

ATGCAGCAGACCAGGCAAGGAAAGCTGTGAGCATGCAGGAAGTTAACTCTGAAGTGATCG

AAAATGAGCCCGTCAGTAAGATCTATTTCGAGCAAGC

>qp1088

CAACGGCCGCGGTATCATGACCGTGCAAAGGTAGCGTAATCACTTGTCTTTTAAATAAAG

ACCCGTATGAAAGGCAAAACGAAAGTTCAACTGTCTCTTTAATCCAATCAATGAAATTTA

TCTTTTCGTGCAGAGGCGGAGATAAATATATAAGACGAGAAGACCCTATGGAGCTTTAAA

CACACTATAATATAAGTTTTAGGTTGGGGCGACCACGGAGCAAAAAAATCCTCCGAGATA

AGAATAACACTTCGAAAATTAAAACATTTTGATCCACTAAGTGACCAACGAACCAAGTTA

CCCTAGGGATAACAGCGCAATCCTTTCTAAGAGTCCATATCGACGAATGGGTTTACGACC

TCGATGTTGGATCAGGACACCCAAATGGTGCAGCCGCTGTTAAGGTTCGTTTGTTCAACG

ATTAAAGTCCTACATGAACCCATATGCACTCTCAATTCTATTATCAAGCCTGGCAGTAGG

AACAATTACAACTCTTTCAAGCTCCCACTGATTTCTCGCATGACTAGGCTTAGAAATAAA

TACACTAGCAATCATCCCCCTAATAACAAAAATACATCACCCACGAGCAACAGAATCAGC

CACAAAATACTTCCTAACGCAAGCCGCCGCATCAGCCCTAATCTTATTTTCTACAATCAC

AAACGCATGGGCCACAGGAGAATGAACAATTATCACCATAAATGACAACACATCAACACT

TATCTTAACAATCGCCCTAGCCATTAAACTGGGAATCGCCCCATTTCACCTGTGATTACC

AGACGTCATACAAGGGCTAAACCTAGCAACATGTTTAATCCTTACAACATGACAAAAACT

AGCCCCAATATCATTAATAATCATAACTAGCCATCAATTAAACGCAAACCTACTAATTAC

AATAGCCATACTATCAACAGTAATTGGGGGGTGGGGGGGCCTTAACCAAACACAAATCCG

AAAAATAATAGCATATTCATCTATCGCTCACCTCGGATGAATAACACTTATCCTGTCTTT

CTCCCCAAACTTAACGACCCTAAACTTAATAATTTACCTAATACTAACCTCATCAATATT

TTTAATAATAATAACCCTAACCTCAACAAATATAAACAAACTTTCAACATCTTGACTGAA

AACGCCACTACTAGCATCATCAATAATAATTACACTCATAGCCCTGGGAGGCCTACCCCC

AACCTCAGGCTTCTTACCAAAATGATTGATCCTACAAGAAATAACTAAACAACACCTAGG

AGCCGTATCTACACTAATAGCTATATCTGCCCTACTAAGCCTATTCTTCTATTTACGACT

ATCATATGCAATCTCACTAACCACTGCACCAAACACCTCAAACTCCAACATAACCTGACG

AGTAACCCACAACCAAATACAAGTCCTCCCAACAATAATCACACTAACTACAATTATACT

ACCAATCACACCAACACTATTAGCAACAGGACAAGGCAGCTTGGCGTACCCAGGTCTGAG

GACCCACGGGACTTTGGAGAGCATAGGTGGGCCTAAAGGCAGTTCAAGAGGAGGGTTGCC

CTCACTAGCAGACACTTTTGAACAAGTCATTGAGGAGATGCTTGAAGATGAGCAGAGCAT

GAGGCCCAGTGAGGAGAACAAGGATGCGGACATGTTTACATCTCGCGTAATGCTGAGCAG

TCAAGTGCCTTTGGAGCCTCCTCTTCTCTTTCTGCTGGAGGAGTACAAAAACTACTTGGA

TGCTGCAAATATGTCCATGAGGGTCCGACGTCACTCTGACCCCGCCCGCCGCGGGGAGCT

GAGTGTGTGTGACAGTATTAGTGAGTGGGTGACGGCATCTGACAAAAAGACTGCCGTGGA

CATGTCGGGGCAGACGGTAACTGTTCTAGAAAAAGTTCCAGTACCCAAAGGCCAACTGAA

GCAATACTTCTACGAGACCAAATGCAACCCTATGGGTTACATGAAGGAGGGCTGCAGAGG

CATTGACAAGAGGTACTGGAACTCCCAGTGCCGAACTACTCAGTCGTATGTGCGCGCGCT

CACCATGGATAACAAGAAGAAAGTGGGCTGGCGGTTTATCAGAATAGACACTTCATGTGT

ATGTACATTGACCATTGTTTGCTTGGGTTGCAGACCGAAGGCTGTTGTTCTACAAATATG

TCTACAAGCGATATAGAGCTGGGAAGCAAAGGGGTATGATTATAGAAACCGAAGGTGACA

GGCCTTCTTCAAAAGCTGACATTGAAATGGATGGAAAGATGGTCAACTCTCATACTGAAA

ATTTCATTGATGGCTCTCTAGTTCTGGAAGTCGATGAGAAAGATCAGGATGATGAAGAAG

CCAGGCGCGATATGGCCAGAATCCTAAAGGAGCTAAAGCAGAAACATCCTGACAAGGAAA

TAGAGCAACTGATTGAGTTAGCGAACTACCAGGTCTTGAGTCAACAGCAAAAAAGTCGAG

CATTCTATCGTATTCAAGCTACTCGCTTGATGACTGGGGCCGGAAATATTCTAAAGAGAC

ATGCAGCAGACCAGGCAAGGAAAGCTGTGAGCATGCAGGAAGTTAACTCTGAAGTGATCG

AAAATGAGCCCGTCAGTAAGATCTATTTCGAGCAAGC

>qp1089

CAACGGCCGCGGTATCATGACCGTGCAAAGGTAGCGTAATCACTTGTCTTTTAAATAAAG

ACCCGTATGAAAGGCAAAACGAAAGTTCGACTGTCTCTTTAATCCAATCAATGAAATTTA

TCTTTTCGTGCAGAGGCGGAGATAAATATATAAGACGAGAAGACCCTATGGAGCTTTAAA

CACACTATAATATAAGTTTTGGGTTGGGGCGACCACGGAGCAAAAAAATCCTCCGAGATA

AGAATAACACTTCGAAAATTAAAACATTTTGATCCACTAAGTGACCAACGAACCAAGTTA

CCCTAGGGATAACAGCGCAATCCTTTCTAAGAGTCCATATCGACGAATGGGTTTACGACC

TCGATGTTGGATCAGGACACCCAAATGGTGCAGCCGCTGTTAAGGTTCGTTTGTTCAACG

ATTAAAGTCCTACATGAACCCATATGCACTCTCAATTCTATTATCAAGCCTGGCAGTAGG

AACAATTACAACTCTTTCAAGCTCCCACTGATTTCTCGCATGACTAGGCTTAGAAATAAA

TACACTAGCAATCATCCCCCTAATAACAAAAATACATCACCCACGAGCAACAGAATCAGC

CACAAAATACTTCCTAACGCAAGCCGCCGCATCAGCCCTAATCTTATTTTCTACAATCAC

AAACGCATGGGCCACAGGAGAATGAACAATTATCACCATAAATGACAACACATCAACACT

TATCTTAACAATCGCCCTAGCCATTAAACTGGGAATCGCCCCATTTCACCTGTGATTACC

AGACGTCATACAAGGGCTAAACCTAGCAACATGTTTAATCCTTACAACATGACAAAAACT

AGCCCCAATATCATTAATAATCATAACTAGCCATCAATTAAACGCAAACCTACTAATTAC

AATAGCCATACTATCAACAGTAATTGGGGGGTGGGGGGGCCTTAACCAAACACAAATCCG

AAAAATAATAGCATATTCATCTATCGCTCACCTCGGATGAATAACACTTATCCTGTCTTT

CTCCCCAAACTTAACGACCCTAAACTTAATAATTTACCTAATACTAACCTCATCAATATT

TTTAATAATAATAACCCTAACCTCAACAAATATAAACAAACTTTCAACATCTTGACTGAA

AACGCCACTACTAGCATCATCAATAATAATTACACTCATAGCCCTGGGAGGCCTACCCCC

AACCTCAGGCTTCTTACCAAAATGATTGATCCTACAAGAAATAACTAAACAACACCTAGG

AGCCGTATCTACACTAATAGCTATATCTGCCCTACTAAGCCTATTCTTCTATTTACGACT

ATCATATGCAATCTCACTAACCACTGCACCAAACACCTCAAACTCCAACATAACCTGACG

AGTAACCCACAACCAAATACAAGTCCTCCCAACAATAATCACACTAACTACAATTATACT

ACCAATCACACCAACACTATTAGCAACAGGACAAGGCAGCTTGGCGTACCCAGGTCTGAG

GACCCACGGGACTTTGGAGAGCATAGGTGGGCCTAAAGGCAGTTCAAGAGGAGGGTTGCC

CTCACTAGCAGACACTTTTGAACAAGTCATTGAGGAGATGCTTGAAGATGAGCAGAGCAT

GAGGCCCAGTGAGGAGAACAAGGATGCGGACATGTTTACATCTCGCGTAATGCTGAGCAG

TCAAGTGCCTTTGGAGCCTCCTCTTCTCTTTCTGCTGGAGGAGTACAAAAACTACTTGGA

TGCTGCAAATATGTCCATGAGGGTCCGACGTCACTCTGACCCCGCCCGCCGCGGGGAGCT

GAGTGTGTGTGACAGTATTAGTGAGTGGGTGACGGCATCTGACAAAAAGACTGCCGTGGA

CATGTCGGGGCAGACGGTAACTGTTCTAGAAAAAGTTCCAGTACCCAAAGGCCAACTGAA

GCAATACTTCTACGAGACCAAATGCAACCCTATGGGTTACATGAAGGAGGGCTGCAGAGG

CATTGACAAGAGGTACTGGAACTCCCAGTGCCGAACTACTCAGTCGTATGTGCGCGCGCT

CACCATGGATAACAAGAAGAAAGTGGGCTGGCGGTTTATCAGAATAGACACTTCATGTGT

ATGTACATTGACCATTGTTTGCTTGGGTTGCAGACCGAAGGCTGTTGTTCTACAAATATG

TCTACAAGCGATATAGAGCTGGGAAGCAAAGGGGTATGATTATAGAAACCGAAGGTGACA

GGCCTTCTTCAAAAGCTGACATTGAAATGGATGGAAAGATGGTCAACTCTCATACTGAAA

ATTTCATTGATGGCTCTCTAGTTCTGGAAGTCGATGAGAAAGATCAGGATGATGAAGAAG

CCAGGCGCGATATGGCCAGAATCCTAAAGGAGCTAAAGCAGAAACATCCTGACAAGGAAA

TAGAGCAACTGATTGAGTTAGCGAACTACCAGGTCTTGAGTCAACAGCAAAAAAGTCGAG

CATTCTATCGTATTCAAGCTACTCGCTTGATGACTGGGGCCGGAAATATTCTAAAGAGAC

ATGCAGCAGACCAGGCAAGGAAAGCTGTGAGCATGCAGGAAGTTAACTCTGAAGTGATCG

AAAATGAGCCCGTCAGTAAGATCTATTTCGAGCAAGC

>qp1091

CAACGGCCGCGGTATCATGACCGTGCAAAGGTAGCGTAATCACTTGTCTTTTAAATAAAG

ACCCGTATGAAAGGCAAAACGAAAGTTCAACTGTCTCTTTAATCCAGTCAATGAAATTTA

TCTTTTCGTGCAGAGGCGGAGATAAGCATATAAGACGAGAAGACCCTATGGAGCTTTAAA

CACACCATAATACAAGTTTTGGGTTGGGGCGACCGCGGAGGAAAAAAATCCTCCGAGATA

AGAATAACACTTCAAAAATTAAAACATTTTGATCCACTAAGTGACCAACGAACCAAGTTA

CCCTAGGGATAACAGCGCAATCCTTTCTAAGAGTCCATATCGACGAATGGGTTTACGACC

TCGATGTTGGATCAGGACACCCAAATGGTGCAGCCGCTGTTAAGGTTCGTTTGTTCAACG

ATTAAAGTCCTACATGAACCCATATGCACTGTCAATTCTATTATCAAGCCTAGCAATAGG

AACAATTACAACCCTTTCAAGCTCCCATTGATTCCTCGCATGAGTGGGCCTAGAAATTAA

CACACTAGCAATTATCCCGCTAATAACAAAAATACACCACCCACGAGCAACAGAATCAGC

CACAAAATATTTCCTAACGCAAGCCGCCGCATCAGCCTTAATCTTATTTTCTACAATCAC

AAACGCATGGGCCACAGGAGAATGAACAATTACTACCATAAATGATAACACATCAACACT

TATCTTAACAATTGCCTTAGCCATTAAACTAGGAATCGCCCCATTTCACCTATGACTACC

AGACGTCATACAAGGATTAAACTTAACGACATGTTTAATCCTTACAACATGACAAAAACT

AGCCCCAATATCACTAATAATCATAACCAGCCATCAATTGAACACAAATCTGCTAATCAC

AATAGCCATACTATCAACAGTAATTGGGGGATGAGGCGGCCTTAACCAAACACAAATACG

AAAAATAATAGCATACTCATCTATCGCCCACCTCGGATGAATGACACTAATCCTATCTTT

CTCCCCAAACTTAACAGCCCTAAACCTAATAATTTACCTCATACTAACCTCATCAATATT

TTTAATAATAATAACCCTAACCTCAACAAATATAAACAAGCTTTCAACATCTTGACTAAA

AACACCTCTACTAGCATCATCAATAATAATTACACTTATAGCCCTGGGGGGCCTACCCCC

AACCTCAGGCTTCTTACCAAAATGACTAATCCTACAAGAAATAACCAAACAACACCTAGG

AGCCGTATCCACACTAATAGCTATATCTGCCCTACTAAGCCTATTCTTCTACCTACGACT

ATCATATGCAATCTCATTAACTACAGCGCCAAACACCTCAAACTCCAACATAACCTGACG

AGTATCAAACAACCAAATACAAGCCCTCCCAACAATAATTACACTAACTACAATTATACT

ACCGATCACACCAACACTACTAGCAATAGGACAAGGCAGCTTGGCGTACCCAGGTCTGAG

GACCCACGGGACTTTGGAGAGCATAGGTGGGCCTAAAGGCAGTTCAAGAGGAGGGTTGCC

CTCACTAGCAGACACTTTTGAACAAGTCATTGAGGAGATGCTTGAAGATGAGCAGAGCAT

GAGGCCCAGTGAGGAGAACAAGGATGCGGACATGTTTACATCGCGCGTAATGCTGAGCAG

TCAAGTGCCTTTGGAGCCTCCTCTTCTCTTTCTGCTGGAGGAGTACAAAAACTACTTGGA

TGCTGCAAATATGTCCATGAGGGTCCGACGTCACTCTGACCCCGCCCGCCGCGGGGAGCT

GAGTGTGTGTGACAGTATTAGTGAGTGGGTGACGGCATCTGACAAAAAGACTGCCGTGGA

CATGTCGGGGCAGACGGTAACTGTTCTAGAAAAAGTTCCAGTACCCAAAGGCCAACTGAA

GCAATACTTCTACGAGACCAAATGCAACCCTATGGGTTACATGAAGGAGGGCTGCAGAGG

CATTGACAAGAGGTACTGGAACTCCCAGTGCCGAACTACTCAGTCGTATGTGCGCGCGCT

CACCATGGATAACAAGAAGAAAGTGGGCTGGCGGTTTATCAGAATAGACACTTCATGTGT

ATGTACATTGACCATTGTTTGCTTGGGTTGCAGACCGAAGGCTGTTGTTCTACAAATATG

TCTACAAGCGATATAGAGCTGGGAAGCAAAGGGGTATGATTATAGAAACCGAAGGTGACA

GGCCTTCTTCAAAAGCTGACATTGAAATGGATGGAAAGATGGTCAACTCTCATACTGAAA

ATTTCATTGATGGCTCTCTAGTTCTGGAAGTCGATGAGAAAGATCAGGATGATGAAGAAG

CCAGGCGCGATATGGCCAGAATCCTAAAGGAGCTAAAGCAGAAACATCCTGACAAGGAAA

TAGAGCAACTGATTGAGTTAGCGAACTACCAGGTCTTGAGTCAACAGCAAAAAAGTCGAG

CATTCTATCGTATTCAAGCTACTCGCTTGATGACTGGGGCCGGAAATATTCTAAAGAGAC

ATGCAGCAGACCAGGCAAGGAAAGCTGTGAGCATGCAGGAAGTTAACTCTGAAGTGATCG

AAAATGAGCCCGTCAGTAAGATCTATTTCGAGCAAGC

>TY01

CAACGGCCGCGGTATCATGACCGTGCAAAGGTAGCGTAATCACTTGTCTTTTAAATAAAG

ACCCGTATGAAAGGCAAAACGAAAGTTCAACTGTCTCTTTAATCCAATCAATGAAATTTA

TCTTTTCGTGCAGAGGCGGAGATAAACATATAAGACGAGAAGACCCTATGGAGCTTCAAA

CACACTGTAATATAAGTTTTAGGTTGGGGCGACCACGGAGCAAAAAAATCCTCCGAGATA

AGAGTAACACTTCGAAAATTAAAACATTTTGATCCACTAAGTGACCAACGAACCAAGTTA

CCCTAGGGATAACAGCGCAATCCTTTCTAAGAGTCCATATCGACGAATGGGTTTACGACC

TCGATGTTGGATCAGGACACCCAAATGGTGCAGCCGCTGTTAAGGTTCGTTTGTTCAACG

ATTAAAGTCCTACATGAACCCATATGCACTCTCAATTCTACTATCAAGCCTAGCAGTAGG

AACAATTACAACTCTTTCAAGCTCCCACTGATTCCTCGCATGACTAGGCTTAGAAATTAA

TACATTAGCAATCATCCCACTGATAACAAAAATACACCACCCACGAGCAACAGAATCGGC

CACAAAATACTTCCTGACGCAAGCCACCGCATCAGCCCTAATCTTATTTTCTACAATCAC

AAACGCATGAGCTACAGGAGAATGAACAATTATTACCATAAATGACAACACATCAACACT

TATCCTAACAATCGCTCTAGCCATTAAACTGGGAATCGCCCCATTTCACCTGTGGTTACC

AGACGTCATACAAGGACTAAACCTAGCAACATGTTTAATCCTTACAACATGACAAAAACT

AGCCCCAATATCACTAATGATTGTAACTAGCCATCAATTAAACACAAACCTACTAATCAC

AATAGCCATACTATCAACAGTAATTGGGGGGTGGGGGGGCCTTAACCAAACACAAATACG

AAAAATAATAGCATATTCATCTATTGCCCACCTCGGGTGAATAACACTTATCCTATCTTT

CTCCCCAAACTTAACAACCCTAAACCTAATAATTTACCTAATACTAACCTCATCAATATT

TTTGATAATAATAACCTTAGCCTCAACAAATATAAACAAACTTTCAACATCTTGACTGAA

AACACCACTACTAGCATCATCAATAATAGTTACACTCATAGCCCTGGGAGGCCTACCCCC

AACATCAGGCTTCTTACCAAAGTGACTAATCCTACAAGAAATAACTAAACAACACCTAGG

AACCGTATCTACACTAATAGCTATATCTGCCCTACTAAGCCTATTCTTCTACCTACGACT

ATCATATGCAATCTCACTAACCACTGCGCCAAACACCTCAAACTCCAACATAACCTGACG

AGTAGCCCACAACCAAATACAGGTCCTCCCAACAATAATTACACTAACTACAATAATACT

ACCAATCACACCAACACTACTAGCAACAGGACAAGGCAGCTTGGCGTACCCAGGTCTGAG

GACCCACGGGACTTTGGAGAGCATAGGTGGGCCTAAAGGCAGTTCAAGAGGAGGGTTGCC

CTCACTAGCAGACACTTTTGAACAAGTCATTGAGGAGATGCTTGAAGATGAGCAGAGCAT

GAGGCCCAGTGAGGAGAACAAGGATGCGGACATGTTTACATCTCGTGTAATGCTGAGCAG

TCAAGTGCCTTTGGAGCCTCCTCTTCTCTTTCTGCTGGAGGAGTACAAAAACTACTTGGA

TGCTGCAAATATGTCCATGAGGGTCCGACGTCACTCTGACCCCGCCCGCCGCGGGGAGCT

GAGTGTGTGTGACAGTATTAGTGAGTGGGTGACGGCATCTGACAAAAAGACTGCCGTGGA

CATGTCGGGGCAGACGGTAACTGTTCTAGAAAAAGTTCCAGTACCCAAAGGCCAACTGAA

GCAATACTTCTACGAGACCAAATGCAACCCTATGGGTTACATGAAGGAGGGCTGCAGAGG

CATTGACAAGAGGTACTGGAACTCCCAGTGCCGAACTACTCAGTCGTATGTGCGCGCGCT

CACCATGGATAACAAGAAGAAAGTGGGCTGGCGGTTTATCAGAATAGACACTTCATGTGT

ATGTACATTGACCATTGTTTGCTTGGGTTGCAGACCGAAGGCTGTTGTTCTACAAATATG

TCTACAAGCGATATAGAGCTGGGAAGCAAAGGGGTATGATTATAGAAACCGAAGGTGACA

GGCCTTCTTCAAAAGCTGACATTGAAATGGATGGAAAGATGGTCAACTCTCATACTGAAA

ATTTCATTGATGGCTCTCTAGTTCTGGAAGTCGATGAGAAAGATCAGGATGATGAAGAAG

CCAGGCGCGATATGGCCAGAATCCTAAAGGAGCTAAAGCAGAAACATCCTGACAAGGAAA

TAGAGCAACTGATTGAGTTAGCGAACTACCAGGTCTTGAGTCAACAGCAAAAAAGTCGAG

CATTCTATCGTATTCAAGCTACTCGCTTGATGACTGGGGCCGGAAATATTCTAAAGAGAC

ATGCAGCAGACCAGGCAAGGAAAGCTGTGAGCATGCAGGAAGTTAACTCTGAAGTGATCG

AAAATGAGCCCGTCAGTAAGATCTATTTCGAGCAAGC

>TY03

CAACGGCCGCGGTATCATGACCGTGCAAAGGTAGCGTAATCACTTGTCTTTTAAATAAAG

ACCCGTATGAAAGGCAAAACGAAAGTTCAACTGTCTCTTTAATCCAATCAATGAAATTTA

TCTTTTCGTGCAGAGGCGGAGATAAACATATAAGACGAGAAGACCCTATGGAGCTTCAAA

CACACTGTAATATAAGTTTTAGGTTGGGGCGACCACGGAGCAAAAAAATCCTCCGAGATA

AGAGTAACACTTCGAAAATTAAAACATTTTGATCCACTAAGTGACCAACGAACCAAGTTA

CCCTAGGGATAACAGCGCAATCCTTTCTAAGAGTCCATATCGACGAATGGGTTTACGACC

TCGATGTTGGATCAGGACACCCAAATGGTGCAGCCGCTGTTAAGGTTCGTTTGTTCAACG

ATTAAAGTCCTACATGAACCCATATGCACTCTCAATTCTACTATCAAGCCTAGCAGTAGG

AACAATTACAACTCTTTCAAGCTCCCACTGATTCCTCGCATGACTAGGCTTAGAAATTAA

TACATTAGCAATCATCCCACTGATAACAAAAATACACCACCCACGAGCAACAGAATCGGC

CACAAAATACTTCCTGACGCAAGCCACCGCATCAGCCCTAATCTTATTTTCTACAATCAC

AAACGCATGAGCTACAGGAGAATGAACAATTATTACCATAAATGACAACACATCAACACT

TATCCTAACAATCGCTCTAGCCATTAAACTGGGAATCGCCCCATTTCACCTGTGGTTACC

AGACGTCATACAAGGACTAAACCTAGCAACATGTTTAATCCTTACAACATGACAAAAACT

AGCCCCAATATCACTAATGATTGTAACTAGCCATCAATTAAACACAAACCTACTAATCAC

AATAGCCATACTATCAACAGTAATTGGGGGGTGGGGGGGCCTTAACCAAACACAAATACG

AAAAATAATAGCATATTCATCTATTGCCCACCTCGGGTGAATAACACTTATCCTATCTTT

CTCCCCAAACTTAACAACCCTAAACCTAATAATTTACCTAATACTAACCTCATCAATATT

TTTGATAATAATAACCTTAGCCTCAACAAATATAAACAAACTTTCAACATCTTGACTGAA

AACACCACTACTAGCATCATCAATAATAGTTACACTCATAGCCCTGGGAGGCCTACCCCC

AACATCAAGCTTCTTACCAAAGTGACTAATCCTACAAGAAATAACTAAACAACACCTAGG

AACCGTATCTACACTAATAGCTATATCTGCCCTACTAAGCCTATTCTTCTACCTACGACT

ATCATATGCAATCTCACTAACCACTGCGCCAAACACCTCAAACTCCAACATAACCTGACG

AGTAGCCCACAACCAAATACAGGTCCTCCCAACAATAATTACACTAACTACAATAATACT

ACCAATCACACCAACACTACTAGCAACAGGACAAGGCAGCTTGGCGTACCCAGGTCTGAG

GACCCACGGGACTTTGGAGAGCATAGGTGGGCCTAAAGGCAGTTCAAGAGGAGGGTTGCC

CTCACTAGCAGACACTTTTGAACAAGTCATTGAGGAGATGCTTGAAGATGAGCAGAGCAT

GAGGCCCAGTGAGGAGAACAAGGATGCGGACATGTTTACATCTCGCGTAATGCTGAGCAG

TCAAGTGCCTTTGGAGCCTCCTCTTCTCTTTCTGCTGGAGGAGTACAAAAACTACTTGGA

TGCTGCAAATATGTCCATGAGGGTCCGACGTCACTCTGACCCCGCCCGCCGCGGGGAGCT

GAGTGTGTGTGACAGTATTAGTGAGTGGGTGACGGCATCTGACAAAAAGACTGCCGTGGA

CATGTCGGGGCAGACGGTAACTGTTCTAGAAAAAGTTCCAGTACCCAAAGGCCAACTGAA

GCAATACTTCTACGAGACCAAATGCAACCCTATGGGTTACATGAAGGAGGGCTGCAGAGG

CATTGACAAGAGGTACTGGAACTCCCAGTGCCGAACTACTCAGTCGTATGTGCGCGCGCT

CACCATGGATAACAAGAAGAAAGTGGGCTGGCGGTTTATCAGAATAGACACTTCATGTGT

ATGTACATTGACCATTGTTTGCTTGGGTTGCAGACCGAAGGCTGTTGTTCTACAAATATG

TCTACAAGCGATATAGAGCTGGGAAGCAAAGGGGTATGATTATAGAAACCGAAGGTGACA

GGCCTTCTTCAAAAGCTGACATTGAAATGGATGGAAAGATGGTCAACTCTCATACTGAAA

ATTTCATTGATGGCTCTCTAGTTCTGGAAGTCGATGAGAAAGATCAGGATGATGAAGAAG

CCAGGCGCGATATGGCCAGAATCCTAAAGGAGCTAAAGCAGAAACATCCTGACAAGGAAA

TAGAGCAACTGATTGAGTTAGCGAACTACCAGGTCTTGAGTCAACAGCAAAAAAGTCGAG

CATTCTATCGTATTCAAGCTACTCGCTTGATGACTGGGGCCGGAAATATTCTAAAGAGAC

ATGCAGCAGACCAGGCAAGGAAAGCTGTGAGCATGCAGGAAGTTAACTCTGAAGTGATCG

AAAATGAGCCCGTCAGTAAGATCTATTTCGAGCAAGC

>TY05

CAACGGCCGCGGTATCATGACCGTGCAAAGGTAGCGTAATCACTTGTCTTTTAAATAAAG

ACCCGTATGAAAGGCAAAACGAAAGTTCAACTGTCTCTTTAATCCAATCAATGAAATTTA

TCTTTTCGTGCAGAGGCGGAGATAAACATATAAGACGAAAAGACCCTATGGAGCTTCAAA

CACACTGTAATATAAGTTTTAGGTTGGGGCGACCACGGAGCAAAAAAATCCTCCGAGATA

AGAGTAACACTTCGAAAATTAAAACATTTTGATCCACTAAGTGACCAACGAACCAAGTTA

CCCTAGGGATAACAGCGCAATCCTTTCTAAGAGTCCATATCGACGAATGGGTTTACGACC

TCGATGTTGGATCAGGACACCCAAATGGTGCAGCCGCTGTTAAGGTTCGTTTGTTCAACG

ATTAAAGTCCTACATGAACCCATATGCACTCTCAATTCTACTATCAAGCCTAGCAGTAGG

AACAATTACAACTCTTTCAAGCTCCCACTGATTCCTCGCATGACTAGGCTTAGAAATTAA

TACATTAGCAATCATCCCACTGATAACAAAAATACACCACCCACGAGCAACAGAATCGGC

CACAAAATACTTCCTGACGCAAGCCACCGCATCAGCCCTAATCTTATTTTCTACAATCAC

AAACGCATGAGCTACAGGAGAATGAGCAATTATTACCATAAATGACAACACATCAACACT

TATCCTAACAATCGCTCTAGCCATTAAACTGGGAATCGCCCCATTTCACCTGTGGTTACC

AGACGTCATACAAGGACTAAACCTAGCAACATGTTTAATCCTTACAACATGACAAAAACT

AGCCCCAATATCACTAATGATTGTAACTAGCCATCAATTAAACACAAACCTACTAATCAC

AATAGCCATACTATCAACAGTAATTGGGGGGTGGGGGGGCCTTAACCAAACACAAATACG

AAAAATAATAGCATATTCATCTATTGCCCACCTCGGGTGAATAACACTTATCCTATCTTT

CTCCCCAAACTTAACAACCCTAAACCTAATAATTTACCTAATACTAACCTCATCAATATT

TTTGATAATAATAACCTTAGCCTCAACAAATATAAACAAACTTTCAACATCTTGACTGAA

AACACCACTACTAGCATCATCAATAATAGTTACACTCATAGCCCTGGGAGGCCTACCCCC

AACCTCAGGCTTCTTACCAAAGTGACTAATCCTACAAGAAATAACTAAACAACACCTAGG

AACCGTATCTACACTAATAGCTATATCTGCCCTACTAAGCCTATTCTTCTACCTACGACT

ATCATATGCAATCTCACTAACCACTGCGCCAAACACCTCAAACTCCAACATAACCTGACG

AGTAGCCCACAACCAAATACAGGTCCTCCCAACAATAATTACACTAACTACAATAATACT

ACCAATCACACCAACACTACTAGCAACAGGACAAGGCAGCTTGGCGTACCCAGGTCTGAG

GACCCACGGGACTTTGGAGAGCATAGGTGGGCCTAAAGGCAGTTCAAGAGGAGGGTTGCC

CTCACTAGCAGACACTTTTGAACAAGTCATTGAGGAGATGCTTGAAGATGAGCAGAGCAT

GAGGCCCAGTGAGGAGAACAAGGATGCGGACATGTTTACATCTCGTGTAATGCTGAGCAG

TCAAGTGCCTTTGGAGCCTCCTCTTCTCTTTCTGCTGGAGGAGTACAAAAACTACTTGGA

TGCTGCAAATATGTCCATGAGGGTCCGACGTCACTCTGACCCCGCCCGCCGCGGGGAGCT

GAGTGTGTGTGACAGTATTAGTGAGTGGGTGACGGCATCTGACAAAAAGACTGCCGTGGA

CATGTCGGGGCAGACGGTAACTGTTCTAGAAAAAGTTCCAGTACCCAAAGGCCAACTGAA

GCAATACTTCTACGAGACCAAATGCAACCCTATGGGTTACATGAAGGAGGGCTGCAGAGG

CATTGACAAGAGGTACTGGAACTCCCAGTGCCGAACTACTCAGTCGTATGTGCGCGCGCT

CACCATGGATAACAAGAAGAAAGTGGGCTGGCGGTTTATCAGAATAGACACTTCATGTGT

ATGTACATTGACCATTGTTTGCTTGGGTTGCAGACCGAAGGCTGTTGTTCTACAAATATG

TCTACAAGCGATATAGAGCTGGGAAGCAAAGGGGTATGATTATAGAAACCGAAGGTGACA

GGCCTTCTTCAAAAGCTGACATTGAAATGGATGGAAAGATGGTCAACTCTCATACTGAAA

ATTTCATTGATGGCTCTCTAGTTCTGGAAGTCGATGAGAAAGATCAGGATGATGAAGAAG

CCAGGCGCGATATGGCCAGAATCCTAAAGGAGCTAAAGCAGAAACATCCTGACAAGGAAA

TAGAGCAACTGATTGAGTTAGCGAACTACCAGGTCTTGAGTCAACAGCAAAAAAGTCGAG

CATTCTATCGTATTCAAGCTACTCGCTTGATGACTGGGGCCGGAAATATTCTAAAGAGAC

ATGCAGCAGACCAGGCAAGGAAAGCTGTGAGCATGCAGGAAGTTAACTCTGAAGTGATCG

AAAATGAGCCCGTCAGTAAGATCTATTTCGAGCAAGC

>TY11

CAACGGCCGCGGTATCATGACCGTGCAAAGGTAGCGTAATCACTTGTCTTTTAAATAAAG

ACCTGTATGAAAGGCAAAACGAAAGTTCAACTGTCTCTTTAATCCAATCAATGAAATTTA

TCTTTTCGTGCAGAAGCGGAAATAAATATATAAGACGAGAAGACCCTATGGAGCTTTAAA

CACACTATAATATAAGTTTTGGGTTGGGGCGACCACGGAGAAAAGAAATCCTCCGAGATA

AGAATAACAATTCGAAAATTAAAACATTTTGATCCACTAAGTGACCAACGAACCAAGTTA

CCCTAGGGATAACAGCGCAATCCTTTCTAAGAGTCCATATCGACGAATGGGTTTACGACC

TCGATGTTGGATCAGGACACCCAAATGGTGCAGCCGCTATTAAGGTTCGTTTGTTCAACG

ATTAAAGTCCTACATGAACCCATATGCACTATCAATTCTATTATCTAGCCTAGCAGTAGG

AACTATCACAACCCTTTCAAGCTCCCATTGATTCCTCGCATGAGTAGGCCTAGAAATCAA

CACACTAGCAATCATCCCACTAATAACAAAAATACACCACCCACGAGCAACAGAATCAGC

AACAAAATACTTCCTAACGCAAGCCACCGCATCAGCCTTAATCTTATTTTCCACAATCAC

AAATGCATGAGCCACAGGAGAATGAACAATTATTACCATAAATGACAACACATCAACATT

TATCTTAACAATTGCCCTGGCCATTAAACTGGGAATCGCCCCATTTCACCTCTGATTGCC

AGACGTCATACAAGGATTAACCCTAACGACATGTCTTATCCTTACAACATGACAAAAATT

AGCCCCAATATCCTTAATAATTATAACCAGCCATCAATTAAACACAAACCTATTAATTAC

AATGGCCATATTATCAACAGTACTTGGGGGGTGGGGGGGCCTTAACCAAACACAAATACG

AAAAATAATAGCATATTCATCTATTGCCCACCTCGGATGAATAACACTAATCCTATCCTT

CTCCCCAAAATTAACAACATTAAACCTAATAATTTACCTAATACTAACCTCATCAATATT

CTTAATAATAATAACAATAACCTCAACAAATATAAATAAACTTTCAACATCTTGACTAAA

GACCCCCATACTGGCATCATCAATAATAATTACACTTATAGCCCTGGGGGGCCTACCCCC

AACCTCAGGCTTCTTGCCAAAATGACTAATTCTACAAGAAATAACCAAACAACACCTGGG

AGCTGTATCCACACTGATAGCTATATCTGCACTACTAAGCCTATTCTTCTACCTACGACT

ATCATATGCAGTCTCACTAACCACTGCACCAAACACCTCAAACTCCAACATGGCCTGACG

AATAACCCACAACCAAATACAAATACACCCCACAATAATTACACTAACTACAATAATACT

ACCAATCACACCAACACTACTAACGATAGGACAAGGCAGCTTGGCGTACCCAGGTCTGAG

GACCCACGGGACTTTGGAGAGCATAGGTGGGCCTAAAGGCAGTTCAAGAGGAGGGTTGCC

CTCACTAGCAGACACTTTTGAACAAGTCATTGAGGAGATGCTTGAAGATGAGCAGAGCAT

GAGGCCCAGTGAGGAGAACAAGGATGCGGACATGTTTACATCTCGCGTAATGCTGAGCAG

TCAAGTGCCTTTGGAGCCTCCTCTTCTCTTTCTGCTGGAGGAGTACAAAAACTACTTGGA

TGCTGCAAATATGTCCATGAGGGTCCGACGTCACTCTGACCCCGCCCGCCGCGGGGAGCT

GAGTGTGTGTGACAGTATTAGTGAGTGGGTGACGGCATCTGACAAAAAGACTGCCGTGGA

CATGTCGGGGCAGACGGTAACTGTTCTAGAAAAAGTTCCAGTACCCAAAGGCCAACTGAA

GCAATACTTCTACGAGACCAAATGCAACCCTATGGGTTACATGAAGGAGGGCTGCAGAGG

CATTGACAAGAGGTACTGGAACTCCCAGTGCCGAACTACTCAGTCGTATGTGCGCGCGCT

CACCATGGATAACAAGAAGAAAGTGGGCTGGCGGTTTATCAGAATAGACACTTCATGTGT

ATGTACATTGACCATTGTTTGCTTGGGTTGCAGACCGAAGGCTGTTGTTCTACAAATATG

TCTACAAGCGATATAGAGCTGGGAAGCAAAGGGGTATGATTATAGAAACCGAAGGTGACA

GGCCTTCTTCAAAAGCTGACATTGAAATGGATGGAAAGATGGTCAACTCTCATACTGAAA

ATTTCATTGATGGCTCTCTAGTTCTGGAAGTCGATGAGAAAGATCAGGATGATGAAGAAG

CCAGGCGCGATATGGCCAGAATCCTAAAGGAGCTAAAGCAGAAACATCCTGACAAGGAAA

TAGAGCAACTGATTGAGTTAGCGAACTACCAGGTCTTGAGTCAACAGCAAAAAAGTCGAG

CATTCTATCGTATTCAAGCTACTCGCTTGATGACTGGGGCCGGAAATATTCTAAAGAGAC

ATGCAGCAGACCAGGCAAGGAAAGCTGTGAGCATGCAGGAAGTTAACTCTGAAGTGATCG

AAAATGAGCCCGTCAGTAAGATCTATTTCGAGCAAGC

>TY12

CAACGGCCGCGGTATCATGACCGTGCAAAGGTAGCGTAATCACTTGTCTTTTAAATAAAG

ACCTGTATGAAAGGCAAAACGAAAGTTCAACTGTCTCTTTAATCCAATCAATGAAATTTA

TCTTTTCGTGCAGAAGCGGAAATAAATATATAAGACGAGAAGACCCTATGGAGCTTTAAA

CACACTATAATATAAGTTTTGGGTTGGGGCGACCACGGAGAAAAGAAATCCTCCGAGATA

AGAATAACAATTCGAAAATTAAAACATTTTGATCCACTAAGTGACCAACGAACCAAGTTA

CCCTAGGGATAACAGCGCAATCCTTTCTAAGAGTCCATATCGACGAATGGGTTTACGACC

TCGATGTTGGATCAGGACACCCAAATGGTGCAGCCGCTATTAAGGTTCGTTTGTTCAACG

ATTAAAGTCCTACATGAACCCATATGCTCTATCAATTCTATTATCTAGCCTAGCAGTAGG

AACTATCACAACCCTTTCAAGCTCCCATTGATTCCTCGCATGAGTAGGCCTAGAAATCAA

CACACTAGCAATCATCCCACTAATAACAAAAATACACCACCCACGAGCAACAGAATCAGC

AACAAAATACTTCCTAACGCAAGCCACCGCATCAGCCTTAATCTTATTTTCCACAATCAC

AAATGCATGAGCCACAGGAGAATGAACAATTATTACCATAAATGACAACACATCAACATT

TATCTTAACAATTGCCCTGGCCATTAAACTGGGAATCGCCCCATTTCACCTCTGATTGCC

AGACGTCATACAAGGATTAACCCTAACGACATGTCTTATCCTTACAACATGACAAAAATT

AGCCCCAATATCCTTAATAATTATAACCAGCCATCAATTAAACACAAACCTATTAATTAC

AATGGCCATATTATCAACAGTACTTGGGGGGTGGGGGGGCCTTAACCAAACACAAATACG

AAAAATAATAGCATATTCATCTATTGCCCACCTCGGATGAATAACACTAATCCTATCCTT

CTCCCCAAAATTAACAACATTAAACCTAATAATTTACCTAATACTAACCTCATCAATATT

CTTAATAATAATAACAATAACCTCAACAAATATAAATAAACTTTCAACATCTTGACTAAA

AACCCCCATACTGGCATCATCAATAATAATTACACTTATAGCCCTGGGGGGCCTACCCCC

AACCTCAGGCTTCTTGCCAAAATGACTAATTCTACAAGAAATAACCAAACAGCACCTGGG

AGCTGTATCCACACTGATAGCTATATCTGCACTACTAAGCCTATTCTTCTACCTACGACT

ATCATATGCAGTCTCACTAACCACTGCACCAAACACCTCAAACTCCAACATGGCCTGACG

AATAACCCACAACCAAATACAAATACACCCCACAATAATTACACTAACTACAATAATACT

ACCAATCACACCAACACTACTAACGATAGGACAAGGCAGCTTGGCGTACCCAGGTCTGAG

GACCCACGGGACTTTGGAGAGCATAGGTGGGCCTAAAGGCAGTTCAAGAGGAGGGTTGCC

CTCACTAGCAGACACTTTTGAACAAGTCATTGAGGAGATGCTTGAAGATGAGCAGAGCAT

GAGGCCCAGTGAGGAGAACAAGGATGCGGACATGTTTACATCTCGCGTAATGCTGAGCAG

TCAAGTGCCTTTGGAGCCTCCTCTTCTCTTTCTGCTGGAGGAGTACAAAAACTACTTGGA

TGCTGCAAATATGTCCATGAGGGTCCGACGTCACTCTGACCCCGCCCGCCGCGGGGAGCT

GAGTGTGTGTGACAGTATTAGTGAGTGGGTGACGGCATCTGACAAAAAGACTGCCGTGGA

CATGTCGGGGCAGACGGTAACTGTTCTAGAAAAAGTTCCAGTACCCAAAGGCCAACTGAA

GCAATACTTCTACGAGACCAAATGCAACCCTATGGGTTACATGAAGGAGGGCTGCAGAGG

CATTGACAAGAGGTACTGGAACTCCCAGTGCCGAACTACTCAGTCGTATGTGCGCGCGCT

CACCATGGATAACAAGAAGAAAGTGGGCTGGCGGTTTATCAGAATAGACACTTCATGTGT

ATGTACATTGACCATTGTTTGCTTGGGTTGCAGACCGAAGGCTGTTGTTCTACAAATATG

TCTACAAGCGATATAGAGCTGGGAAGCAAAGGGGTATGATTATAGAAACCGAAGGTGACA

GGCCTTCTTCAAAAGCTGACATTGAAATGGATGGAAAGATGGTCAACTCTCATACTGAAA

ATTTCATTGATGGCTCTCTAGTTCTGGAAGTCGATGAGAAAGATCAGGATGATGAAGAAG

CCAGGCGCGATATGGCCAGAATCCTAAAGGAGCTAAAGCAGAAACATCCTGACAAGGAAA

TAGAGCAACTGATTGAGTTAGCGAACTACCAGGTCTTGAGTCAACAGCAAAAAAGTCGAG

CATTCTATCGTATTCAAGCTACTCGCTTGATGACTGGGGCCGGAAATATTCTAAAGAGAC

ATGCAGCAGACCAGGCAAGGAAAGCTGTGAGCATGCAGGAAGTTAACTCTGAAGTGATCG

AAAATGAGCCCGTCAGTAAGATCTATTTCGAGCAAGC

>TY13

CAACGGCCGCGGTATCATGACCGTGCAAAGGTAGCGTAATCACTTGTCTTTTAAATAAAG

ACCTGTATGAAAGGCAAAACGAAAGTTCAACTGTCTCTTTAATCCAATCAATGAAATTTA

TCTTTTCGTGCAGAAGCGGAAATAAACATATAAGACGAGAAGACCCTATGGAGCTTTAAG

CACACTGTAATATAAGCTTTAGGTTGGGGCGACCGCGGAGAAAAAAAATCCTCCGAGATA

AGAACAACACTTCGAAAATTAAAACATTTTGATCCACTAAGTGACCAACGAACCAAGTTA

CCCTAGGGATAACAGCGCAATCCTTTCTAAGAGTCCATATCGACGAACGGGTTTACGACC

TCGATGTTGGATCAGGACACCCAAATGGTGCAGCCGCTATTAAGGTTCGTTTGTTCAACG

ATTAAAGTCCTACATGAACCCGTATGCGCTGTCAATTTTATTGTCGAGCCTAGCAGTAGG

AACAATCACAACTCTTTCAAGCTCCCACTGATTCCTCGCATGAGTGGGCCTAGAAATCAA

TACACTAGCAATCATCCCGCTAATAACAAAAATACACCACCCACGAGCAACAGAATCCGC

TACAAAATACTTCCTAACACAAGCCGCCGCATCAGCCCTAATCTTATTTTCTACAATCAC

AAACGCATGGACCACAGGAGAATGAACAATTACCACCATAAATAACAATATATCAACATC

TACCCTAACAATTGCCCTAGCCATAAAACTGGGAATCGCCCCATTTCACCTGTGGCTACC

AGACGTTATACAAGGACTTAACCTAACAACATGCTTAATCCTTACAACATGACAAAAATT

AGCCCCCATGTCATTAATAATTATAACTGGCCATCAACTAAGCACAAACCTATTAATTAC

AATAGCCACACTATCAACAGTAATTGGGGGGTGAGGCGGCCTTAACCAAACGCACATACG

AAAAATAATAGCATATTCATCTGTCGCCCACCTCGGGTGAATAACACTAGTCCTGTCTTT

CTCACCAAACTTAACAACCCTAAACCTAATAATCTACCTAATATTAACCTCATCAATATT

CTTAATAATAATGGCCCTGGCCTCAACAAGTATAAACAAACTTTCAACATCTTGACTGAA

AACGCCTCTACTAGCGTCATCAATAATAATTACACTTATGGCCTTAGGGGGGCTGCCCCC

AACCTCCGGGTTCTTACCAAAATGACTTATCCTGCAAGAAATAACCAAACAACACCTTGG

AACCGTATCCACACTAATAGCTTTATCTGCACTACTAAGCCTGTTCTTCTACTTACGACT

GTCATATGCAATCTCATTAACTACTGCACCAAACATCTCAAACTCCAACATGACATGACG

AATGCCCCCCAACCAAATACAAATCCTCCCCACAATAATCACACTAACTACAATAATACT

ACCAATCACACCAACACTACTAACGATAGGACAAGGCAGCTTGGCGTACCCAGGTCTGAG

GACCCACGGGACTTTGGAGAGCATAGGTGGGCCTAAAGGCAGTTCAAGAGGAGGGTTGCC

CTCACTAGCAGACACTTTTGAACAAGTCATTGAGGAGATGCTTGAAGATGAGCAGAGCAT

GAGGCCCAGTGAGGAGAACAAGGATGCGGACATGTTTACATCTCGCGTAATGCTGAGCAG

TCAAGTGCCTTTGGAGCCTCCTCTTCTCTTTCTGCTGGAGGAGTACAAAAACTACTTGGA

TGCTGCAAATATGTCCATGAGGGTCCGACGTCACTCTGACCCCGCCCGCCGCGGGGAGCT

GAGTGTGTGTGACAGTATTAGTGAGTGGGTGACGGCATCTGACAAAAAGACTGCCGTGGA

CATGTCGGGGCAGACGGTAACTGTTCTAGAAAAAGTTCCAGTACCCAAAGGCCAACTGAA

GCAATACTTCTACGAGACCAAATGCAACCCTATGGGTTACATGAAGGAGGGCTGCAGAGG

CATTGACAAGAGGTACTGGAACTCCCAGTGCCGAACTACTCAGTCGTATGTGCGCGCGCT

CACCATGGATAACAAGAAGAAAGTGGGCTGGCGGTTTATCAGAATAGACACTTCATGTGT

ATGTACATTGACCATTGTTTGCTTGGGTTGCAGACCGAAGGCTGTTGTTCTACAAATATG

TCTACAAGCGATATAGAGCTGGGAAGCAAAGGGGTATGATTATAGAAACCGAAGGTGACA

GGCCTTCTTCAAAAGCTGACATTGAAATGGATGGAAAGATGGTCAACTCTCATACTGAAA

ATTTCATTGATGGCTCTCTAGTTCTGGAAGTCGATGAGAAAGATCAGGATGATGAAGAAG

CCAGGCGCGATATGGCCAGAATCCTAAAGGAGCTAAAGCAGAAACATCCTGACAAGGAAA

TAGAGCAACTGATTGAGTTAGCGAACTACCAGGTCTTGAGTCAACAGCAAAAAAGTCGAG

CATTCTATCGTATTCAAGCTACTCGCTTGATGACTGGGGCCGGAAATATTCTAAAGAGAC

ATGCAGCAGACCAGGCAAGGAAAGCTGTGAGCATGCAGGAAGTTAACTCTGAAGTGATCG

AAAATGAGCCCGTCAGTAAGATCTATTTCGAGCAAGC

>TY14

CAACGGCCGCGGTATCATGACCGTGCAAAGGTAGCGTAATCACTTGTCTTTTAAATAAAG

ACCTGTATGAAAGGCAAAACGAAAGTTCAACTGTCTCTTTAATCCAATCAATGAAATTTA

TCTTTTCGTGCAGAAGCGGAAATAAACATATAAGACGAGAAGACCCTATGGAGCTTTAAG

CACACTGTAATATAAGCTTTAGGTTGGGGCGACCGCGGAGAAAAAAAATCCTCCGAGATA

AGAACAACACTTCGAAAATTAAAACATTTTGATCCACTAAGTGACCAACGAACCAAGTTA

CCCTAGGGATAACAGCGCAATCCTTTCTAAGAGTCCATATCGACGAACGGGTTTACGACC

TCGATGTTGGATCAGGACACCCAAATGGTGCAGCCGCTATTAAGGTTCGTTTGTTCAACG

ATTAAAGTCCTACATGAACCCGTATGCGCTGTCAATTTTATTGTCGAGCCTAGCAGTAGG

AACAATCACAACTCTTTCAAGCTCCCACTGATTCCTCGCATGAGTGGGCCTAGAAATCAA

TACACTAGCAATCATCCCGCTAATAACAAAAATACACCACCCACGAGCAACAGAATCCGC

TACAAAATACTTCCTAACACAAGCCGCCGCATCAGCCCTAATCTTATTTTCTACAATCAC

AAACGCATGGACCACAGGAGAATGAACAATTACCACCATAAATAACAATATATCAACATC

TACCCTAACAATTGCCCTAGCCATAAAACTGGGAATCGCCCCATTTCACCTGTGGCTACC

AGACGTTATACAAGGACTTAACCTAACAACATGCTTAATCCTTACAACATGACAAAAATT

AGCCCCCATGTCATTAATAATTATAACTGGCCATCAACTAAGCACAAACCTATTAATTAC

AATAGCCACACTATCAACAGTAATTGGGGGGTGAGGCGGCCTTAACCAAACGCACATACG

AAAAATAATAGCATATTCATCTGTCGCCCACCTCGGGTGAATAACACTAGTCCTGTCTTT

CTCACCAAACTTAACAACCCTAAACCTAATAATCTACCTAATATTAACCTCATCAATATT

CTTAATAATAATGGCCCTGGCCTCAACAAGTATAAACAAACTTTCAACATCTTGACTGAA

AACGCCTCTACTAGCGTCATCAATAATAATTACACTTATGGCCTTAGGGGGGCTGCCCCC

AACCTCCGGGTTCTTACCAAAATGACTTATCCTGCAAGAAATAACCAAACAACACCTTGG

AACCGTATCCACACTAATAGCTTTATCTGCACTACTAAGCCTGTTCTTCTACTTACGACT

GTCATATGCAATCTCATTAACTACTGCACCAAACATCTCAAACTCCAACATGACATGACG

AATGCCCCCCAACCAAATACAAATCCTCCCCACAATAATCACACTAACTACAATAATACT

ACCAATCACACCAACACTACTAACGATAGGACAAGGCAGCTTGGCGTACCCAGGTCTGAG

GACCCACGGGACTTTGGAGAGCATAGGTGGGCCTAAAGGCAGTTCAAGAGGAGGGTTGCC

CTCACTAGCAGACACTTTTGAACAAGTCATTGAGGAGATGCTTGAAGATGAGCAGAGCAT

GAGGCCCAGTGAGGAGAACAAGGATGCGGACATGTTTACATCTCGCGTAATGCTGAGCAG

TCAAGTGCCTTTGGAGCCTCCTCTTCTCTTTCTGCTGGAGGAGTACAAAAACTACTTGGA

TGCTGCAAATATGTCCATGAGGGTCCGACGTCACTCTGACCCCGCCCGCCGCGGGGAGCT

GAGTGTGTGTGACAGTATTAGTGAGTGGGTGACGGCATCTGACAAAAAGACTGCCGTGGA

CATGTCGGGGCAGACGGTAACTGTTCTAGAAAAAGTTCCAGTACCCAAAGGCCAACTGAA

GCAATACTTCTACGAGACCAAATGCAACCCTATGGGTTACATGAAGGAGGGCTGCAGAGG

CATTGACAAGAGGTACTGGAACTCCCAGTGCCGAACTACTCAGTCGTATGTGCGCGCGCT

CACCATGGATAACAAGAAGAAAGTGGGCTGGCGGTATATCAGAATAGACACTTCATGTGT

ATGTACATTGACCATTGTTTGCTTGGGTTGCAGACCGAAGGCTGTTGTTCTACAAATATG

TCTACAAGCGATATAGAGCTGGGAAGCAAAGGGGTATGATTATAGAAACCGAAGGTGACA

GGCCTTCTTCAAAAGCTGACATTGAAATGGATGGAAAGATGGTCAACTCTCATACTGAAA

ATTTCATTGATGGCTCTCTAGTTCTGGAAGTCGATGAGAAAGATCAGGATGATGAAGAAG

CCAGGCGCGATATGGCCAGAATCCTAAAGGAGCTAAAGCAGAAACATCCTGACAAGGAAA

TAGAGCAACTGATTGAGTTAGCGAACTACCAGGTCTTGAGTCAACAGCAAAAAAGTCGAG

CATTCTATCGTATTCAAGCTACTCGCTTGATGACTGGGGCCGGAAATATTCTAAAGAGAC

ATGCAGCAGACCAGGCAAGGAAAGCTGTGAGCATGCAGGAAGTTAACTCTGAAGTGATCG

AAAATGAGCCCGTCAGTAAGATCTATTTCGAGCAAGC

>TY15

CAACGGCCGCGGTATCATGACCGTGCAAAGGTAGCGTAATCACTTGTCTTTTAAATAAAG

ACCTGTATGAAAGGCAAAACGAAAGTTCAACTGTCTCTTTAATCCAATCAATGAAATTTA

TCTTTTCGTGCAGAAGCGGAAATAAACATATAAGACGAGAAGACCCTATGGAGCTTTAAG

CACACTGTAATATAAGCTTTAGGTTGGGGCGACCGCGGAGAAAAAAAATCCTCCGAGATA

AGAACAACACTTCGAAAATTAAAACATTTTGATCCACTAAGTGACCAACGAACCAAGTTA

CCCTAGGGATAACAGCGCAATCCTTTCTAAGAGTCCATATCGACGAACGGGTTTACGACC

TCGATGTTGGATCAGGACACCCAAATGGTGCAGCCGCTATTAAGGTTCGTTTGTTCAACG

ATTAAAGTCCTACATGAACCCGTATGCGCTGTCAATTTTATTGTCGAGCCTAGCAGTAGG

AACAATCACAACTCTTTCAGGCTCCCACTGATTCCTCGCATGAGTGGGCCTAGAAATCAA

TACACTAGCAATCATCCCACTAATAACAAAAATACACCACCCACGAGCAACAGAATCCGC

TACAAAATACTTCCTAACACAAGCCGCCGCATCAGCCCTAATCTTATTTTCTACAATCAC

AAACGCATGGACCACAGGAGAATGAACAATTACCACCATAAATAACAATATATCAACATC

TACCCTAACAATTGCCCTAGCCATAAAACTGGGAATCGCCCCATTTCACCTGTGGCTACC

AGACGTTATACAAGGACTTAACCTAACAACATGCTTAATCCTTACAACATGACAAAAATT

AGCACCCATGTCATTAATAATTATAACTGGCCATCAACTAAGCACAAACCTATTAATTAC

AATAGCCACACTATCAACAGTAATTGGGGGGTGAGGCGGCCTTAACCAAACGCACATACG

AAAAATAATAGCATATTCATCTGTCGCCCACCTCGGGTGAATAACACTAGTCCTGTCTTT

CTCACCAAACTTAACAACCCTAAACCTAATAATCTACCTAATATTAACCTCATCAATATT

CTTAATAATAATGGCCCTGGCCTCAACAAGTATAAACAAACTTTCAACATCTTGACTGAA

AACGCCTCTACTAGCATCATCAATAATAATTACACTTATGGCCTTAGGGGGGCTGCCCCC

AACCTCCGGGTTCTTACCAAAATGACTTATCCTGCAAGAAATAACCAAACAACACCTTGG

AACCGTATCCACACTAATAGCTTTATCTGCACTACTAAGCCTGTTCTTCTACTTACGACT

GTCATATGCAATCTCATTAACTACTGCACCAAACATCTCAAACTCCAACATGACATGACG

AATGCCCCCCAACCAAATACAAATCCTCCCCACAATAATTACACTAACTACAATAATACT

ACCAATCACACCAACACTACTAACGATAGGACAAGGCAGCTTGGCGTACCCAGGTCTGAG

GACCCACGGGACTTTGGAGAGCATAGGTGGGCCTAAAGGCAGTTCAAGAGGAGGGTTGCC

CTCACTAGCAGACACTTTTGAACAAGTCATTGAGGAGATGCTTGAAGATGAGCAGAGCAT

GAGGCCCAGTGAGGAGAACAAGGATGCGGACATGTTTACATCTCGCGTAATGCTGAGCAG

TCAAGTGCCTTTGGAGCCTCCTCTTCTCTTTCTGCTGGAGGAGTACAAAAACTACTTGGA

TGCTGCAAATATGTCCATGAGGGTCCGACGTCACTCTGACCCCGCCCGCCGCGGGGAGCT

GAGTGTGTGTGACAGTATTAGTGAGTGGGTGACGGCATCTGACAAAAAGACTGCCGTGGA

CATGTCGGGGCAGACGGTAACTGTTCTAGAAAAAGTTCCAGTACCCAAAGGCCAACTGAA

GCAATACTTCTACGAGACCAAATGCAACCCTATGGGTTACATGAAGGAGGGCTGCAGAGG

CATTGACAAGAGGTACTGGAACTCCCAGTGCCGAACTACTCAGTCGTATGTGCGCGCGCT

CACCATGGATAACAAGAAGAAAGTGGGCTGGCGGTTTATCAGAATAGACACTTCATGTGT

ATGTACATTGACCATTGTTTGCTTGGGTTGCAGACCGAAGGCTGTTGTTCTACAAATATG

TCTACAAGCGATATAGAGCTGGGAAGCAAAGGGGTATGATTATAGAAACCGAAGGTGACA

GGCCTTCTTCAAAAGCTGACATTGAAATGGATGGAAAGATGGTCAACTCTCATACTGAAA

ATTTCATTGATGGCTCTCTAGTTCTGGAAGTCGATGAGAAAGATCAGGATGATGAAGAAG

CCAGGCGCGATATGGCCAGAATCCTAAAGGAGCTAAAGCAGAAACATCCTGACAAGGAAA

TAGAGCAACTGATTGAGTTAGCGAACTACCAGGTCTTGAGTCAACAGCAAAAAAGTCGAG

CATTCTATCGTATTCAAGCTACTCGCTTGATGACTGGGGCCGGAAATATTCTAAAGAGAC

ATGCAGCAGACCAGGCAAGGAAAGCTGTGAGCATGCAGGAAGTTAACTCTGAAGTGATCG

AAAATGAGCCCGTCAGTAAGATCTATTTCGAGCAAGC

>TY17

CAACGGCCGCGGTATCATGACCGTGCAAAGGTAGCGTAATCACTTGTCTTTTAAATAAAG

ACCCGTATGAAAGGCAAAACGAAAGTTCAACTGTCTCTTTAATCCAATCAATGAAATTTA

TCTTTTCGTGCAGAGGCGGAGATAAATGTATAAGACGAGAAGACCCTATGGAGCTTTAAA

CACACTATAATATAAGTTTTGGGTTGGGGCGACCACGGAGCAAAAAAATCCTCCGAGATA

AGAATAACACTTCGAAAATTAAAACATTTTGATCCACTAAGTGACCAACGAACCAAGTTA

CCCTAGGGATAACAGCGCAATCCTTTCTAAGAGTCCATATCGACGAATGGGTTTACGACC

TCGATGTTGGATCAGGACACCCAAATGGTGCAGCCGCTGTTAAGGTTCGTTTGTTCAACG

ATTAAAGTCCTACATGAACCCATATGCACTGTCAATTCTATTATCAAGCCTAGCAATAGG

AACAATTACAACCCTTTCAAGCTCCCATTGATTCCTCGCATGAGTGGGCCTAGAAATTAA

CACACTAGCAATTATCCCGCTAATAACAAAAATACACCACCCACGAGCAACAGAATCAGC

CACAAAATATTTCCTAACGCAAGCCGCCGCATCAGCCTTAATCTTATTTTCTACAATCAC

AAACGCATGGGCCACAGGAGAATGAACAATTACTACCATAAATGATAACACATCAACACT

TATCTTAACAATTGCCTTAGCCATTAAACTAGGAATCGCCCCATTTCACCTATGACTACC

AGACGTCATACAAGGATTAAACTTAACGACATGTTTAATCCTTACAACATGACAAAAACT

AGCCCCAATATCACTAATAATCATAACCAGCCATCAATTGAACACAAATCTGCTAATCAC

AATAGCCATACTATCAACAGTAATTGGGGGATGAGGCGGCCTTAACCAAACACAAATACG

AAAAATAATAGCATACTCATCTATCGCCCACCTCGGATGAATGACACTAATCCTATCTTT

CTCCCCAAACTTAACAGCCCTAAACCTAATAATTTACCTCATACTAACCTCATCAATATT

TTTAATAATAATAACCCTAACCTCAACAAATATAAACAAGCTTTCAACATCTTGACTAAA

AACACCTCTACTAGCATCATCAATAATAATTACACTTATAGCCCTGGGGGGCCTACCCCC

AACCTCAGGCTTCTTACCAAAATGACTAATCCTACAAGAAATAACCAAACAACACCTAGG

AGCCGTATCCACACTAATAGCTATATCTGCCCTACTAAGCCTATTCTTCTACCTACGACT

ATCATATGCAATCTCATTAACTACAGCGCCAAACACCTCAAACTCCAACATAACCTGACG

AGTATCAAACAACCAAATACAAGCCCTCCCAACAATAATTACACTAACTACAATTATACT

ACCAATCACACCAACACTACTAGCAATAGGACAAGGCAGCTTGGCGTACCCAGGTCTGAG

GACCCACGGGACTTTGGAGAGCATAGGTGGGCCTAAAGGCAGTTCAAGAGGAGGGTTGCC

CTCACTAGCAGACACTTTTGAACAAGTCATTGAGGAGATGCTTGAAGATGAGCAGAGCAT

GAGGCCCAGTGAGGAGAACAAGGATGCGGACATGTTTACATCGCGCGTAATGCTGAGCAG

TCAAGTGCCTTTGGAGCCTCCTCTTCTCTTTCTGCTGGAGGAGTACAAAAACTACTTGGA

TGCTGCAAATATGTCCATGAGGGTCCGACGTCACTCTGACCCCGCCCGCCGCGGGGAGCT

GAGTGTGTGTGACAGTATTAGTGAGTGGGTGACGGCATCTGACAAAAAGACTGCCGTGGA

CATGTCGGGGCAGACGGTAACTGTTCTAGAAAAAGTTCCAGTACCCAAAGGCCAACTGAA

GCAATACTTCTACGAGACCAAATGCAACCCTATGGGTTACATGAAGGAGGGCTGCAGAGG

CATTGACAAGAGGTACTGGAACTCCCAGTGCCGAACTACTCAGTCGTATGTGCGCGCGCT

CACCATGGATAACAAGAAGAAAGTGGGCTGGCGGTTTATCAGAATAGACACTTCATGTGT

ATGTACATTGACCATTGTTTGCTTGGGTTGCAGACCGAAGGCTGTTGTTCTACAAATATG

TCTACAAGCGATATAGAGCTGGGAAGCAAAGGGGTATGATTATAGAAACCGAAGGTGACA

GGCCTTCTTCAAAAGCTGACATTGAAATGGATGGAAAGATGGTCAACTCTCATACTGAAA

ATTTCATTGATGGCTCTCTAGTTCTGGAAGTCGATGAGAAAGATCAGGATGATGAAGAAG

CCAGGCGCGATATGGCCAGAATCCTAAAGGAGCTAAAGCAGAAACATCCTGACAAGGAAA

TAGAGCAACTGATTGAGTTAGCGAACTACCAGGTCTTGAGTCAACAGCAAAAAAGTCGAG

CATTCTATCGTATTCAAGCTACTCGCTTGATGACTGGGGCCGGAAATATTCTAAAGAGAC

ATGCAGCAGACCAGGCAAGGAAAGCTGTGAGCATGCAGGAAGTTAACTCTGAAGTGATCG

AAAATGAGCCCGTCAGTAAGATCTATTTCGAGCAAGC

>TY20

CAACGGCCGCGGTATCATGACCGTGCAAAGGTAGCGTAATCACTTGTCTTTTAAATAAAG

ACCTGTATGAAAGGCAAAACGAAAGTTCAACTGTCTCTTTAATCCAATCAATAAAATTTA

TCTCTCCGTGCAGAAGCGGAAATAAATATATAAGACGAGAAGACCCTATGGAGCTTTAAA

CACACTATAATATAAGTTTTAGGTTGGGGCGACCGCGGAAGAAAAAAATCCTCCGAGATA

AGAATAACACTTCGAAAATTAAAACATTTTGATCCACTAAGTGACCAACGAACCAAGTTA

CCCTAGGGATAACAGCGCAATCCTTTCTAAGAGTCCGTATCGACGAATGGGTTTACGACC

TCGATGTTGGATCAGGACACCCAAATGGTGCAGCCGCTATTAAGGTTCGTTTGTTCAACG

ATTAAAGTCCTACATGAACCCATATGCGCTGTCAATTCTGTTGTCGAGCCTAGCAGTAGG

AACAATCACAACCCTTTCAAGCTCCCATTGATTCCTCGCATGAATAGGCCTAGAAATTAA

CACACTAGCAATCATCCCACTAATAACAAAAATACACCACCCACGAGCAACAGAATCAGC

CACAAAATACTTCCTAACGCAAGCCACCGCATCAGCCTTAATCTTATTTTCTACAATCAC

AAACGCATGGGCCACAGGAGAATGAACAATTATCACCATAAATGACAACACATCAACATT

TATCTTAACAATTGCCCTAGCCATTAAACTGGGAATCGCCCCATTTCACCTTTGATTACC

AGACGTCATACAAGGATTAACCCTAACAACATGCCTAATCCTTACAACATGACAAAAATT

AGCCCCAATATCCCTAATAATTATAGCCAGCCATCAATTAAACACAAACCTGCTAATTAC

AATGGCCGTATTATCAACAGTACTTGGGGGATGGGGCGGCCTTAACCAAACACAAATACG

AAAAATAATAGCATACTCATCTATTGCCCACCTCGGATGAATAACACTAATCCTATCCTT

CTCCCCAAGCCTAACAACATTAAACCTAATAATTTACCTAATACTAACCTCATCAATATT

CTTAATAATAATAACAATAACCTCAACAAATATAAATAAACTTTCAACATCTTGACTGAA

GGCTCCCCTACTGGCATCATCAATAATAATTACACTTATAGCCCTAGGAGGCCTACCCCC

AACCTCAGGCTTCTTGCCAAAATGACTAATCCTACAAGAAATAATCAAACAACATCTGGA

AGCTGTATCCACACTAATGGCCATATCTGCACTACTAAGCCTATTCTTCTACCTACGACT

ATCATATGCGATCTCATTAACTACTGCACCAAACACCTCAAACTCTAACATAACCTGACG

AATAAGCCACAACCAAATACAAATCCACCCCCAAATAATTACACTAACTACAATAATACT

ACCAATCCCGCCAACACTACTAGCAATAGGACAAGGCAGCTTGGCGTACCCAGGTCTGAG

GACCCACGGGACTTTGGAGAGCATAGGTGGGCCTAAAGGCAGTTCAAGAGGAGGGTTGCC

CTCACTAGCAGACACTTTTGAACAAGTCATTGAGGAGATGCTTGAAGATGAGCAGAGCAT

GAGGCCCAGTGAGGAGAACAAGGATGCGGACATGTTTACATCTCGCGTAATGCTGAGCAG

TCAAGTGCCTTTGGAGCCTCCTCTTCTCTTTCTGCTGGAGGAGTACAAAAACTACTTGGA

TGCTGCAAATATGTCCATGAGGGTCCGACGTCACTCTGACCCCGCCCGCCGCGGGGAGCT

GAGTGTGTGTGACAGTATTAGTGAGTGGGTGACGGCATCTGACAAAAAGACTGCCGTGGA

CATGTCGGGGCAGACGGTAACTGTTCTAGAAAAAGTTCCAGTACCCAAAGGCCAACTGAA

GCAATACTTCTACGAGACCAAATGCAACCCTATGGGTTACATGAAGGAGGGCTGCAGAGG

CATTGACAAGAGGTACTGGAACTCCCAGTGCCGAACTACTCAGTCGTATGTGCGCGCGCT

CACCATGGATAACAAGAAGAAAGTGGGCTGGCGGTATATCAGAATAGACACTTCATGTGT

ATGTACATTGACCATTGTTTGCTTGGGTTGCAGACCGAAGGCTGTTGTTCTACAAATATG

TCTACAAGCGATATAGAGCTGGGAAGCAAAGGGGTATGATTATAGAAACCGAAGGTGACA

GGCCTTCTTCAAAAGCTGACATTGAAATGGATGGAAAGATGGTCAACTCTCATACTGAAA

ATTTCATTGATGGCTCTCTAGTTCTGGAAGTCGATGAGAAAGATCAGGATGATGAAGAAG

CCAGGCGCGATATGGCCAGAATCCTAAAGGAGCTAAAGCAGAAACATCCTGACAAGGAAA

TAGAGCAACTGATTGAGTTAGCGAACTACCAGGTCTTGAGTCAACAGCAAAAAAGTCGAG

CATTCTATCGTATTCAAGCTACTCGCTTGATGACTGGGGCCGGAAATATTCTAAAGAGAC

ATGCAGCAGACCAGGCAAGGAAAGCTGTGAGCATGCAGGAAGTTAACTCTGAAGTGATCG

AAAATGAGCCCGTCAGTAAGATCTATTTCGAGCAAGC

>TY21

CAACGGCCGCGGTATCATGACCGTGCAAAGGTAGCGTAATCACTTGTCTTTTAAATAAAG

ACCTGTATGAAAGGCAAAACGAAAGTTCAACTGTCTCTTTAATCCAATCAATAAAATTTA

TCTCTCCGTGCAGAAGCGGAAATAAATATATAAGACGAGAAGACCCTATGGAGCTTTAAA

CACACTATAATATAAGTTTTAGGTTGGGGCGACCGCGGAAGAAAAAAATCCTCCGAGATA

AGAATAACACTTCGAAAATTAAAACATTTTGATCCACTAAGTGACCAACGAACCAAGTTA

CCCTAGGGATAACAGCGCAATCCTTTCTAAGAGTCCGTATCGACGAATGGGTTTACGACC

TCGATGTTGGATCAGGACACCCAAATGGTGCAGCCGCTATTAAGGTTCGTTTGTTCAACG

ATTAAAGTCCTACATGAACCCATATGCGCTGTCAATTCTGTTGTCGAGCCTAGCAGTAGG

AACAATCACAACCCTTTCAAGCTCCCATTGATTCCTCGCATGAGTAGGCCTAGAAATTAA

CACACTAGCAATCATCCCACTAATAACAAAAATACACCACCCACGAGCAACAGAATCAGC

CACAAAATACTTCCTAACGCAAGCCACCGCATCAGCCTTAATCTTATTTTCTACAATCAC

AAACGCATGGGCCACAGGAGAATGAACAATTATCACCATAAATGACAACACATCAACATT

TATCTTAACAATTGCCCTAGCCATTAAACTGGGAATCGCCCCATTTCACCTTTGATTACC

AGACGTCATACAAGGATTAACCCTAACAACATGCCTAATCCTTACAACATGACAAAAATT

AGCCCCAATATCCCTAATAATTATAGCCAGCCATCAATTAAACACAAACCTGCTAATTAC

AATGGCCGTATTATCAACAGTACTTGGGGGATGGGGCGGCCTTAACCAAACACAAATACG

AAAAATAATAGCATACTCATCTATTGCCCACCTCGGATGAATAACACTAATCCTATCCTT

CTCCCCAAGCCTAACAACATTAAACCTAATAATTTACCTAATACTAACCTCATCAATATT

CTTAATAATAATAACAATAACCTCAACAAATATAAATAAACTTTCAACATCTTGACTGAA

GGCTCCCCTACTGGCATCATCAATAATAATTACACTTATAGCCCTAGGAGGCCTACCCCC

AACCTCAGGCTTCTTGCCAAAATGACTAATCCTACAAGAAATAATCAAACAACATCTGGA

AGCTGTATCCACACTAATGGCCATATCTGCACTACTAAGCCTATTCTTCTACCTAGGACT

ATCATATGCGATCTCATTAACTACTGCACCAAACACCTCAAACTCTAACATAAGCTGACG

AATAAGCCACAACCAAATACAAATCCACCCCCAAATAATTACACTAACTACAATAATACT

ACCAATCCCGCCAACACTACTAGCAATAGGACAAGGCAGCTTGGCGTACCCAGGTCTGAG

GACCCACGGGACTTTGGAGAGCATAGGTGGGCCTAAAGGCAGTTCAAGAGGAGGGTTGCC

CTCACTAGCAGACACTTTTGAACAAGTCATTGAGGAGATGCTTGAAGATGAGCAGAGCAT

GAGGCCCAGTGAGGAGAACAAGGATGCGGACATGTTTACATCTCGCGTAATGCTGAGCAG

TCAAGTGCCTTTGGAGCCTCCTCTTCTCTTTCTGCTGGAGGAGTACAAAAACTACTTGGA

TGCTGCAAATATGTCCATGAGGGTCCGACGTCACTCTGACCCCGCCCGCCGCGGGGAGCT

GAGTGTGTGTGACAGTATTAGTGAGTGGGTGACGGCATCTGACAAAAAGACTGCCGTGGA

CATGTCGGGGCAGACGGTAACTGTTCTAGAAAAAGTTCCAGTACCCAAAGGCCAACTGAA

GCAATACTTCTACGAGACCAAATGCAACCCTATGGGTTACATGAAGGAGGGCTGCAGAGG

CATTGACAAGAGGTACTGGAACTCCCAGTGCCGAACTACTCAGTCGTATGTGCGCGCGCT

CACCATGGATAACAAGAAGAAAGTGGGCTGGCGGTATATCAGAATAGACACTTCATGTGT

ATGTACATTGACCATTGTTTGCTTGGGTTGCAGACCGAAGGCTGTTGTTCTACAAATATG

TCTACAAGCGATATAGAGCTGGGAAGCAAAGGGGTATGATTATAGAAACCGAAGGTGACA

GGCCTTCTTCAAAAGCTGACATTGAAATGGATGGAAAGATGGTCAACTCTCATACTGAAA

ATTTCATTGATGGCTCTCTAGTTCTGGAAGTCGATGAGAAAGATCAGGATGATGAAGAAG

CCAGGCGCGATATGGCCAGAATCCTAAAGGAGCTAAAGCAGAAACATCCTGACAAGGAAA

TAGAGCAACTGATTGAGTTAGCGAACTACCAGGTCTTGAGTCAACAGCAAAAAAGTCGAG

CATTCTATCGTATTCAAGCTACTCGCTTGATGACTGGGGCCGGAAATATTCTAAAGAGAC

ATGCAGCAGACCAGGCAAGGAAAGCTGTGAGCATGCAGGAAGTTAACTCTGAAGTGATCG

AAAATGAACCCGTCAGTAAGATCTATTTCGAGCAAGC

>TY22

CAACGGCCGCGGTATCATGACCGTGCAAAGGTAGCGTAATCACTTGTCTTTTAAATAAAG

ACCTGTATGAAAGGCAAAACGAAAGTTCAACTGTCTCTTTAATCCAATCAATAAAATTTA

TCTCTCCGTGCAGAAGCGGAAATAAATATATAAGACGAGAAGACCCTATGGAGCTTTAAA

CACACTATAATATAAGTTTTAGGTTGGGGCGACCGCGGAAGAAAAAAATCCTCCGAGATA

AGAATAACACTTCGAAAATTAAAACATTTTGATCCACTAAGTGACCAACGAACCAAGTTA

CCCTAGGGATAACAGCGCAATCCTTTCTAAGAGTCCGTATCGACGAATGGGTTTACGACC

TCGATGTTGGATCAGGACACCCAAATGGTGCAGCCGCTATTAAGGTTCGTTTGTTCAACG

ATTAAAGTCCTACATGAACCCATATGCGCTGTCAATTCTGTTGTCGAGCCTAGCAGTAGG

AACAATCACAACCCTTTCAAGCTCCCATTGATTCCTCGCATGAATAGGCCTAGAAATTAA

CACACTAGCAATCATCCCACTAATAACAAAAATACACCACCCACGAGCAACAGAATCAGC

CACAAAATACTTCCTAACGCAAGCCACCGCATCAGCCTTAATCTTATTTTCTACAATCAC

AAACGCATGGGCCACAGGAGAATGAACAATTATCACCATAAATGACAACACATCAACATT

TATCTTAACAATTGCCCTAGCCATTAAACTGGGAATCGCCCCATTTCACCTTTGATTACC

AGACGTCATACAAGGATTAACCCTAACAACATGCCTAATCCTTACAACATGACAAAAATT

AGCCCCAATATCCCTAATAATTATAGCCAGCCATCAATTAAACACAAACCTGCTAATTAC

AATGGCCGTATTATCAACAGTACTTGGGGGATGGGGCGGCCTTAACCAAACACAAATACG

AAAAATAATAGCATACTCATCTATTGCCCACCTCGGATGAATAACACTAATCCTATCCTT

CTCCCCAAGCCTAACAACATTAAACCTAATAATTTACCTAATACTAACCTCATCAATATT

CTTAATAATAATAACAATAACCTCAACAAATATAAATAAACTTTCAACATCTTGACTGAA

GGCTCCCCTACTGGCATCATCAATAATAATTACACTTATAGCCCTAGGAGGCCTACCCCC

AACCTCAGGCTTCTTGCCAAAATGACTAATCCTACAAGAAATAATCAAACAACATCTGGA

AGCTGTATCCACACTAATGGCCATATCTGCACTACTAAGCCTATTCTTCTACCTACGACT

ATCATATGCGATCTCATTAACTACTGCACCAAACACCTCAAACTCTAACATAAGCTGACG

AATAACCCACAACCAAATACAAATCCACCCCCAAATAATTACACTAACTACAATAATACT

ACCAATCCCGCCAACACTACTAGCAATAGGACAAGGCAGCTTGGCGTACCCAGGTCTGAG

GACCCACGGGACTTTGGAGAGCATAGGTGGGCCTAAAGGCAGTTCAAGAGGAGGGTTGCC

CTCACTAGCAGACACTTTTGAACAAGTCATTGAGGAGATGCTTGAAGATGAGCAGAGCAT

GAGGCCCAGTGAGGAGAACAAGGATGCGGACATGTTTACATCTCGCGTAATGCTGAGCAG

TCAAGTGCCTTTGGAGCCTCCTCTTCTCTTTCTGCTGGAGGAGTACAAAAACTACTTGGA

TGCTGCAAATATGTCCATGAGGGTCCGACGTCACTCTGACCCCGCCCGCCGCGGGGAGCT

GAGTGTGTGTGACAGTATTAGTGAGTGGGTGACGGCATCTGACAAAAAGACTGCCGTGGA

CATGTCGGGGCAGACGGTAACTGTTCTAGAAAAAGTTCCAGTACCCAAAGGCCAACTGAA

GCAATACTTCTACGAGACCAAATGCAACCCTATGGGTTACATGAAGGAGGGCTGCAGAGG

CATTGACAAGAGGTACTGGAACTCCCAGTGCCGAACTACTCAGTCGTATGTGCGCGCGCT

CACCATGGATAACAAGAAGAAAGTGGGCTGGCGGTATATCAGAATAGACACTTCATGTGT

ATGTACATTGACCATTGTTTGCTTGGGTTGCAGACCGAAGGCTGTTGTTCTACAAATATG

TCTACAAGCGATATAGAGCTGGGAAGCAAAGGGGTATGATTATAGAAACCGAAGGTGACA

GGCCTTCTTCAAAAGCTGACATTGAAATGGATGGAAAGATGGTCAACTCTCATACTGAAA

ATTTCATTGATGGCTCTCTAGTTCTGGAAGTCGATGAGAAAGATCAGGATGATGAAGAAG

CCAGGCGCGATATGGCCAGAATCCTAAAGGAGCTAAAGCAGAAACATCCTGACAAGGAAA

TAGAGCAACTGATTGAGTTAGCGAACTACCAGGTCTTGAGTCAACAGCAAAAAAGTCGAG

CATTCTATCGTATTCAAGCTACTCGCTTGATGACTGGGGCCGGAAATATTCTAAAGAGAC

ATGCAGCAGACCAGGCAAGGAAAGCTGTGAGCATGCAGGAAGTTAACTCTGAAGTGATCG

AAAATGAACCCGTCAGTAAGATCTATTTCGAGCAAGC

>TY23

CAACGGCCGCGGTATCATGACCGTGCAAAGGTAGCGTAATCACTTGTCTTTTAAATAAAG

ACCTGTATGAAAGGCAAAACGAAAGTTCAACTGTCTCTTTAATCCAATCAATAAAATTTA

TCTCTCCGTGCAGAAGCGGAAATAAATATATAAGACGAGAAGACCCTATGGAGCTTTAAA

CACACTATAATATAAGTTTTAGGTTGGGGCGACCGCGGAAGAAAAAAATCCTCCGAGATA

AGAATAACACTTCGAAAATTAAAACATTTTGATCCACTAAGTGACCAACGAACCAAGTTA

CCCTAGGGATAACAGCGCAATCCTTTCTAAGAGTCCGTATCGACGAATGGGTTTACGACC

TCGATGTTGGATCAGGACACCCAAATGGTGCAGCCGCTATTAAGGTTCGTTTGTTCAACG

ATTAAAGTCCTACATGAACCCATATGCGCTGTCAATTCTGTTGTCGAGCCTAGCAGTAGG

AACAATCACAACCCTTTCAAGCTCCCATTGATTCCTCGCATGAATAGGCCTAGAAATTAA

CACACTAGCAATCATCCCACTAATAACAAAAATACACCACCCACGAGCAACAGAATCAGC

CACAAAATACTTCCTAACGCAAGCCACCGCATCAGCCTTAATCTTATTTTCTACAATCAC

AAACGCATGGGCCACAGGAGAATGAACAATTATCACCATAAATGACAACACATCAACATT

TATCTTAACAATTGCCCTAGCCATTAAACTGGGAATCGCCCCATTTCACCTTTGATTACC

AGACGTCATACAAGGATTAACCCTAACAACATGCCTAATCCTTACAACATGACAAAAATT

AGCCCCAATATCCCTAATAATTATAGCCAGCCATCAATTAAACACAAACCTGCTAATTAC

AATGGCCGTATTATCAACAGTACTTGGGGGATGGGGCGGCCTTAACCAAACACAAATACG

AAAAATAATAGCATACTCATCTATTGCCCACCTCGGATGAATAACACTAATCCTATCCTT

CTCCCCAAGCCTAACAACATTAAACCTAATAATTTACCTAATACTAACCTCATCAATATT

CTTAATAATAATAACAATAACCTCAACAAATATAAATAAACTTTCAACATCTTGACTGAA

GGCTCCCCTACTGGCATCATCAATAATAATTACACTTATAGCCCTAGGAGGCCTACCCCC

AACCTCAGGCTTCTTGCCAAAATGACTAATCCTACAAGAAATAATCAAACAACATCTGGA

AGCTGTATCCACACTAATGGCCATATCTGCACTACTAAGCCTATTCTTCTACCTACGACT

ATCATATGCGATCTCATTAACTACTGCACCAAACACCTCAAACTCTAACATAAGCTGACG

AATAAGCCACAACCAAATACAAATCCACCCCCAAATAATTACACTAACTACAATAATACT

ACCAATCCCGCCAACACTACTAGCAATAGGACAAGGCAGCTTGGCGTACCCAGGTCTGAG

GACCCACGGGACTTTGGAGAGCATAGGTGGGCCTAAAGGCAGTTCAAGAGGAGGGTTGCC

CTCACTAGCAGACACTTTTGAACAAGTCATTGAGGAGATGCTTGAAGATGAGCAGAGCAT

GAGGCCCAGTGAGGAGAACAAGGATGCGGACATGTTTACATCTCGCGTAATGCTGAGCAG

TCAAGTGCCTTTGGAGCCTCCTCTTCTCTTTCTGCTGGAGGAGTACAAAAACTACTTGGA

TGCTGCAAATATGTCCATGAGGGTCCGACGTCACTCTGACCCCGCCCGCCGCGGGGAGCT

GAGTGTGTGTGACAGTATTAGTGAGTGGGTGACGGCATCTGACAAAAAGACTGCCGTGGA

CATGTCGGGGCAGACGGTAACTGTTCTAGAAAAAGTTCCAGTACCCAAAGGCCAACTGAA

GCAATACTTCTACGAGACCAAATGCAACCCTATGGGTTACATGAAGGAGGGCTGCAGAGG

CATTGACAAGAGGTACTGGAACTCCCAGTGCCGAACTACTCAGTCGTATGTGCGCGCGCT

CACCATGGATAACAAGAAGAAAGTGGGCTGGCGGTATATCAGAATAGACACTTCATGTGT

ATGTACATTGACCATTGTTTGCTTGGGTTGCAGACCGAAGGCTGTTGTTCTACAAATATG

TCTACAAGCGATATAGAGCTGGGAAGCAAAGGGGTATGATTATAGAAACCGAAGGTGACA

GGCCTTCTTCAAAAGCTGACATTGAAATGGATGGAAAGATGGTCAACTCTCATACTGAAA

ATTTCATTGATGGCTCTCTAGTTCTGGAAGTCGATGAGAAAGATCAGGATGATGAAGAAG

CCAGGCGCGATATGGCCAGAATCCTAAAGGAGCTAAAGCAGAAACATCCTGACAAGGAAA

TAGAGCAACTGATTGAGTTAGCGAACTACCAGGTCTTGAGTCAACAGCAAAAAAGTCGAG

CATTCTATCGTATTCAAGCTACTCGCTTGATGACTGGGGCCGGAAATATTCTAAAGAGAC

ATGCAGCAGACCAGGCAAGGAAAGCTGTGAGCATGCAGGAAGTTAACTCTGAAGTGATCG

AAAATGAACCCGTCAGTAAGATCTATTTCGAGCAAGC

>TY29

CAACGGCCGCGGTATCATGACCGTGCAAAGGTAGCGTAATCACTTGTCTTTTAAATAAAG

ACCTGTATGAAAGGCAAAACGAAAGTTCAACTGTCTCTTTAATCCAATCAATGAAATTTA

TCTTTTCGTGCAGAAGCGGAAATAAATATATAAGACGAGAAGACCCTATGGAGCTTTAAA

CACGCTATAATACAAGTTTTTGGTTGGGGCGACCGCGGAGAAAAGAAATCCTCCGAGATA

AGAATAACACTTCAAAAATTAAAACATTTTGATCCACTAAGTGACCAACGAACCAAGTTA

CCCTAGGGATAACAGCGCAATCCTTTCTAAGAGTCCATATCGACGAATGGGTTTACGACC

TCGATGTTGGATCAGGACACCCAAATGGTGCAGCCGCTATTAAGGTTCGTTTGTTCAACG

ATTAAAGTCCTACATGAACCCGTATGCACTATCAATCCTGTTGTCGAGCCTAGCAGTAGG

AACAATTACAACTCTCTCAAGCTCCCATTGATTTCTGGCATGAGTAGGACTAGAAATTAA

TACACTAGCAATAATCCCGCTAATAACAAAAATACACCACCCACGATCAACAGAATCAGC

CACAAAATACTTCCTAACGCAAGCCGCCGCATCAGCCTTAATCTTATTTTCTACAATCAT

AAACGCATGGGCTACAGGAGAATGAACAATTATCACCATAAATGACAACTCATCAACATT

TATATTAACAGTTGCCCTAGCTATTAAACTTGGAATCGCCCCTTTTCATCTATGACTACC

AGACGTCATACAAGGGTTAAACCTAACAACATGCCTAATCCTTGCAACATGACAAAAATT

AGCCCCAATATCCTTAATAATTATAGCCAATCACCAACTAAACACAAACCTGCTAATTAC

AATAGCCGTATTATCAGCAGCAATTGGGGGGTGGGGGGGACTTAACCAAACACAAATCCG

AAAAATAATAGCATATTCATCTATCGCCCACCTTGGGTGAATAACACTAGTCCTCTCTTT

CTCCCCAAATTTAACAACCCTTAACCTAATAATTTACCTAATACTAACATCATCAATGTT

CTTAATAATAACAACCCTAAACTCAACAAATATTAACAAACTTTCAACATCTTGACTAAA

AACCCCACTACTAGCATCATCAATAATAATTACACTCATAGCCCTAGGAGGCCTACCCCC

CACATCAGGATTCCTACCAAAATGACTTATCCTACAAGAAATAACCAAACAACACCTAGG

AGCCGTGTCCACACTAATAGCTATATCTGCACTACTAAGCCTATTCTTCTACTTACGACT

ATCATATGCAATCTCACTAACTACCGCACCAAACACATCAAACTCCAACATAACCTGACG

AATAGCCCACAACCAAATACAAATCCTACCAACAATAATCACACTAACTACAATAATACT

ACCGATCACACCAACACTACTAGCAATAGGACAAGGCAGCTTGGCGTACCCAGGTCTGAG

GACCCACGGGACTTTGGAGAGCATAGGTGGGCCTAAAGGCAGTTCAAGAGGAGGGTTGCC

CTCACTAGCAGACACTTTTGAACAAGTCATTGAGGAGATGCTTGAAGATGAGCAGAGCAT

GAGGCCCAGTGAGGAGAACAAGGATGCGGACATGTTTACATCTCGCGTAATGCTGAGCAG

TCAAGTGCCTTTGGAGCCTCCTCTTCTCTTTCTGCTGGAGGAGTACAAAAACTACTTGGA

TGCTGCAAATATGTCCATGAGGGTCCGACGTCACTCTGACCCCGCCCGCCGCGGGGAGCT

GAGTGTGTGTGACAGTATTAGTGAGTGGGTGACGGCATCTGACAAAAAGACTGCCGTGGA

CATGTCGGGGCAGACGGTAACTGTTCTAGAAAAAGTTCCAGTACCCAAAGGCCAACTGAA

GCAATACTTCTACGAGACCAAATGCAACCCTATGGGTTACATGAAGGAGGGCTGCAGAGG

CATTGACAAGAGGTACTGGAACTCCCAGTGCCGAACTACTCAGTCGTATGTGCGCGCGCT

CACCATGGATAACAAGAAGAAAGTGGGCTGGCGGTTTATCAGAATAGACACTTCATGTGT

ATGTACATTGACCATTGTTTGCTTGGGTTGCAGACCGAAGGCTGTTGTTCTACAAATATG

TCTACAAGCGATATAGAGCTGGGAAGCAAAGGGGTATGATTATAGAAACCGAAGGTGACA

GGCCTTCTTCAAAAGCTGACATTGAAATGGATGGAAAGATGGTCAACTCTCATACTGAAA

ATTTCATTGATGGCTCTCTAGTTCTGGAAGTTGATGAGAAAGATCAGGATGATGAAGAAG

CCAGGCGCGATATGGCCAGAATCCTAAAGGAGCTAAAGCAGAAACATCCTGACAAGGAAA

TAGAGCAACTGATTGAGTTAGCGAACTACCAGGTCTTGAGTCAACAGCAAAAAAGTCGAG

CATTCTATCGTATTCAAGCTACTCGCTTGATGACTGGGGCCGGAAATATTCTAAAGAGAC

ATGCAGCAGACCAGGCAAGGAAAGCTGTGAGCATGCAGGAAGTTAACTCTGAAGTGATCG

AAAATGAGCCCGTCAGTAAGATCTATTTCGAGCAAGC

>TY30

CAACGGCCGCGGTATCATGACCGTGCAAAGGTAGCGTAATCACTTGTCTTTTAAATAAAG

ACCCGTATGAAAGGCAAAACGAAAGTTCAACTGTCTCTTTAATCCAATCAATGAAATTTA

TCTTTTCGTGCAGAGGCGGAGATAAATATATAAGACGAGAAGACCCTATGGAGCTTTAAA

CACACTATAATACAAGTTTTAGGTTGGGGCGACCACGGAGCAAAAAAATCCTCCGAGATA

AGAATAACACTTCGAAAATTAAAACATTTTGATCCACTAAGTGACCAACGAACCAAGTTA

CCCTAGGGATAACAGCGCAATCCTTTCTAAGAGTCCATATCGACGAATGGGTTTACGACC

TCGATGTTGGATCAGGACACCCAAATGGTGCAGCCGCTATTAAGGTTCGTTTGTTCAACG

ATTAAAGTCCTACATGAACCCATATGCCCTGTCAATTCTATTATCGAGCCTGGCAATAGG

AACAATTACAACTCTTTCAAGCTCCCACTGATTCCTCGCATGAGTTGGCCTAGAAATTAA

CACGCTGGCAATCATCCCACTAATAACAAAAATACACCACCCACGAGCAACAGAATCAGC

CACAAAATACTTCCTAACACAAGCCACCGCATCAGCCTTAATCTTATTTTCTGCAATCAC

AAACGCATGGGCCACAGGAGAATGAACAATTATTACCATGAACGATAACACATCAACATT

TATCTTAACAATCGCCCTATCCATTAAGCTGGGAATCGCCCCATTTCACCTATGACTACC

AGACGTCATACAAGGGCTAAACCTAACAACATGTTTAATCCTTACAACATGACAAAAACT

AGCCCCAATATCACTAATAATCATAACCAGCCATCAATTAAACACAAGCCTACTAATTAC

AATAGCCATATTATCAACAGTAGTTGGGGGGTGGGGGGGCCTTAACCAAACACAAATACG

AAAAATAATAGCATACTCATCCATCGCCCACCTCGGATGAATAACACTAGTCCTATCTTT

CTCCCCAAGCTTAACGGCCCTGAACCTCATAATTTACCTTATACTAACCTCATCAATATT

TTTAATAATAATAACCCTGACCGCAACAAATATAAACAAACTTTCAACATCTTGACTAAA

AACACCACTACTAGCATCATTAATAATAATTACACTTATAGCCCTAGGAGGCCTACCCCC

GACCTCAGGCTTCTTACCAAAATGACTAATTCTACAAGAAATAACTAAACAGCACCTAGA

GACCGTATCCACACTAATGGCTATATCTGCCCTACTAAGCCTGTTCTTCTACCTACGACT

GTCGTATGCAATCTCATTAACTACTGCACCAAACACCTCAAACTCCAACATAACCTGACG

AGTATCTCACAACCAAATACAAACTCTCCCAACAATAATCACACTAACCACAATAATATT

ACCGATCACACCAACACTACTAGCAATAGGACAAGGCAGCTTGGCGTACCCAGGTCTGAG

GACCCACGGGACTTTGGAGAGCATAGGTGGGCCTAAAGGCAGTTCAAGAGGAGGGTTGCC

CTCACTAGCAGACACTTTTGAACAAGTCATTGAGGAGATGCTTGAAGATGAGCAGAGCAT

GAGGCCCAGTGAGGAGAACAAGGATGCGGACATGTTTACATCGCGCGTAATGCTGAGCAG

TCAAGTGCCTTTGGAGCCTCCTCTTCTCTTTCTGCTGGAGGAGTACAAAAACTACTTGGA

TGCTGCAAATATGTCCATGAGGGTCCGACGTCACTCTGACCCCGCCCGCCGCGGGGAGCT

GAGTGTGTGTGACAGTATTAGTGAGTGGGTGACGGCATCTGACAAAAAGACTGCCGTGGA

CATGTCGGGGCAGACGGTAACTGTTCTAGAAAAAGTTCCAGTACCCAAAGGCCAACTGAA

GCAATACTTCTACGAGACCAAATGCAACCCTATGGGTTACATGAAGGAGGGCTGCAGAGG

CATTGACAAGAGGTACTGGAACTCCCAGTGCCGAACTACTCAGTCGTATGTGCGCGCGCT

CACCATGGATAACAAGAAGAAAGTGGGCTGGCGGTTTATCAGAATAGACACTTCATGTGT

ATGTACATTGACCATTGTTTGCTTGGGTTGCAGACCGAAGGCTGTTGTTCTACAAATATG

TCTACAAGCGATATAGAGCTGGGAAGCAAAGGGGTATGATTATAGAAACCGAAGGTGACA

GGCCTTCTTCAAAAGCTGACATTGAAATGGATGGAAAGATGGTCAACTCTCATACTGAAA

ATTTCATTGATGGCTCTCTAGTTCTGGAAGTCGATGAGAAAGATCAGGATGATGAAGAAG

CCAGGCGCGATATGGCCAGAATCCTAAAGGAGCTAAAGCAGAAACATCCTGACAAGGAAA

TAGAGCAACTGATTGAGTTAGCGAACTACCAGGTCTTGAGTCAACAGCAAAAAAGTCGAG

CATTCTATCGTATTCAAGCTACTCGCTTGATGACTGGGGCCGGAAATATTCTAAAGAGAC

ATGCAGCAGACCAGGCAAGGAAAGCTGTGAGCATGCAGGAAGTTAACTCTGAAGTGATCG

AAAACGAGCCCGTCAGTAAGATCTATTTCGAGCAAGC

>TY31

CAACGGCCGCGGTATCATGACCGTGCAAAGGTAGCGTAATCACTTGTCTTTTAAATAAAG

ACCCGTATGAAAGGCAAAACGAAAGTTCAACTGTCTCTTTAATCCAATCAATGAAATTTA

TCTTTTCGTGCAGAGGCGGAGATAAATATATAAGACGAGAAGACCCTATGGAGCTTTAAA

CACACTATAATACAAGTTTTAGGTTGGGGCGACCACGGAGCAAAAAAATCCTCCGAGATA

AGAATAACACTTCGAAAATTAAAACATTTTGATCCACTAAGTGACCAACGAACCAAGTTA

CCCTAGGGATAACAGCGCAATCCTTTCTAAGAGTCCATATCGACGAATGGGTTTACGACC

TCGATGTTGGATCAGGACACCCAAATGGTGCAGCCGCTATTAAGGTTCGTTTGTTCAACG

ATTAAAGTCCTACATGAACCCATATGCCCTGTCAATTCTATTATCGAGCCTGGCAATAGG

AACAATTACAACTCTTTCAAGCTCCCACTGATTCCTCGCATGAGTTGGCCTAGAAATTAA

CACGCTGGCAATCATCCCACTAATAACAAAAATACACCACCCACGAGCAACAGAATCAGC

CACAAAATACTTCCTAACACAAGCCACCGCATCAGCCTTAATCTTATTTTCTGCAATCAC

AAACGCATGGGCCACAGGAGAATGAACAATTATTACCATGAACGATAACACATCAACATT

TATCTTAACAATCGCCCTATCCATTAAGCTGGGAATCGCCCCATTTCACCTATGACTACC

AGACGTCATACAAGGGCTAAACCTAACAACATGTTTAATCCTTACAACATGACAAAAACT

AGCCCCAATATCACTAATAATCATAACCAGCCATCAATTAAACACAAGCCTACTAATTAC

AATAGCCATATTATCAACAGTAGTTGGGGGGTGGGGGGGCCTTAACCAAACACAAATACG

AAAAATAATAGCATACTCATCCATCGCCCACCTCGGATGAATAACACTAGTCCTATCTTT

CTCCCCAAGCTTAACGGCCCTGAACCTCATAATTTACCTTATACTAACCTCATCAATATT

TTTAATAATAATAACCCTGACCGCAACAAATATAAACAAACTTTCAACATCTTGACTAAA

AACACCACTACTAGCATCATTAATAATAATTACACTTATAGCCCTAGGAGGCCTACCCCC

GACCTCAGGCTTCTTACCAAAATGACTAATTCTACAAGAAATAACTAAACAGCACCTAGA

GACCGTATCCACACTAATGGCTATATCTGCCCTACTAAGCCTGTTCTTCTACCTACGACT

GTCGTATGCAATCTCATTAACTACTGCACCAAACACCTCAAACTCCAACATAACCTGACG

AGTATCTCACAACCAAATACAAACTCTCCCAACAATAATCACACTAACCACAATAATATT

ACCGATCACACCAACACTACTAGCAATAGGACAAGGCAGCTTGGCGTACCCAGGTCTGAG

GACCCACGGGACTTTGGAGAGCATAGGTGGGCCTAAAGGCAGTTCAAGAGGAGGGTTGCC

CTCACTAGCAGACACTTTTGAACAAGTCATTGAGGAGATGCTTGAAGATGAGCAGAGCAT

GAGGCCCAGTGAGGAGAACAAGGATGCGGACATGTTTACATCGCGCGTAATGCTGAGCAG

TCAAGTGCCTTTGGAGCCTCCTCTTCTCTTTCTGCTGGAGGAGTACAAAAACTACTTGGA

TGCTGCAAATATGTCCATGAGGGTCCGACGTCACTCTGACCCCGCCCGCCGCGGGGAGCT

GAGTGTGTGTGACAGTATTAGTGAGTGGGTGACGGCATCTGACAAAAAGACTGCCGTGGA

CATGTCGGGGCAGACGGTAACTGTTCTAGAAAAAGTTCCAGTACCCAAAGGCCAACTGAA

GCAATACTTCTACGAGACCAAATGCAACCCTATGGGTTACATGAAGGAGGGCTGCAGAGG

CATTGACAAGAGGTACTGGAACTCCCAGTGCCGAACTACTCAGTCGTATGTGCGCGCGCT

CACCATGGATAACAAGAAGAAAGTGGGCTGGCGGTTTATCAGAATAGACACTTCATGTGT

ATGTACATTGACCATTGTTTGCTTGGGTTGCAAACCGAAGGCTGTTGTTCTACAAATATG

TCTACAAGCGATATAGAGCTGGGAAGCAAAGGGGTATGATTATAGAAACCGAAGGTGACA

GGCCTTCTTCAAAAGCTGACATTGAAATGGATGGAAAGATGGTCAACTCTCATACTGAAA

ATTTCATTGATGGCTCTCTAGTTCTGGAAGTCGATGAGAAAGATCAGGATGATGAAGAAG

CCAGGCGCGATATGGCCAGAATCCTAAAGGAGCTAAAGCAGAAACATCCTGACAAGGAAA

TAGAGCAACTGATTGAGTTAGCGAACTACCAGGTCTTGAGTCAACAGCAAAAAAGTCGAG

CATTCTATCGTATTCAAGCTACTCGCTTGATGACTGGGGCCGGAAATATTCTAAAGAGAC

ATGCAGCAGACCAGGCAAGGAAAGCTGTGAGCATGCAGGAAGTTAACTCTGAAGTGATCG

AAAATGAGCCCGTCAGTAAGATCTATTTCGAGCAAGC

>TY32

????????????????????????????????????????????????????????????

????????????????????????????????????????????????????????????

????????????????????????????????????????????????????????????

????????????????????????????????????????????????????????????

????????????????????????????????????????????????????????????

????????????????????????????????????????????????????????????

????????????????????????????????????????????????????????????

?????????????ATGAGCCCATATGCACTATCAATTCTATTATCGAGCCTAGCAGTAGG

AACAATCACAACCCTTTCAAGCTCCCATTGATTCCTCGCATGAATGGGACTAGAAATTAA

CACACTAGCAATCATCCCACTAATAACAAAAATACACCACCCACGAGCAACAGAATCAGC

TACAAAATACTTCCTAACGCAAGCCACCGCATCAGCTTTAATCTTGTTTTCTACAATCAC

AAACGCATGAGCCACAGGAGAATGAACAATTATTACCATAAATGACAACACATCAACATT

TATCTTAACAATTGCCCTGGCCATCAAACTGGGAATCGCCCCATTTCACCTCTGATTACC

AGACGTCATACAAGGGTTAACCCTAACAACATGTCTAATCCTTACAACATGACAAAAATT

AGCCCCAATATCCTTAATAATTATAACCGGCCATCAATTAAACACAAACCTACTGATTAC

AATGGCCATACTATCAACAGTGTTAGGGGGGTGGGGGGGCCTTAACCAAACACAAATACG

AAAAATAATAGCATATTCATCTATTGCCCACCTCGGATGAATAACACTAATCCTATCCTT

CTCCCCAGACTTAACAACATTAAACCTAATAATTTACCTAATACTAACCTCATCTATATT

CTTAATAATAATAACAATAACCTCAACAAACATAAATAAACTTTCAACATCTTGACTAAA

AACCCCACTACTTGCATCATCAATGATAATTACACTTATAGCCCTAGGAGGCTTACCCCC

AACCTCAGGCTTCTTGCCAAAATGGCTAATCCTACAAGAAATAACCAAACAACACCTGGG

AGCTGTATCCACACTAATAGCTATATCCGCCCTACTAAGCCTATTCTTCTACCTACGACT

ATCATATGCAATCTCATTAACCACTGCACCAAACACCTCAAACTCCAACATAGCCTGACG

AATAACCCACAGCCAAATACAAATCCACCCAACAATAATTACACTAACCACAATAATACT

ACCAATCACACCAACACTACTAGCAATAGGACAAGGCAGCTTGGCGTACCCAGGTCTGAG

GACCCACGGGACTCTGGAGAGCATAGGTGGGCCTAAAGGCAATTCAAGAGGAGGGTTGCC

CTCACTAGCAGACACTTTTGAACAAGTCATTGAGGAGATGCTTGAAGATGAGCAGAGCAT

GAGGCCCAGTGAGGAGAACAAGGATGCGGACATGTTTACATCTCGCGTAATGCTGAGCAG

TCAAGTGCCTTTGGAGCCTCCTCTTCTCTTTCTGCTGGAGGAGTACAAAAACTACTTGGA

TGCTGCAAATATGTCCATGAGGGTCCGACGTCACTCTGACCCCGCACGCCGCGGGGAGCT

GAGTGTGTGTGACAGTATTAGTGAGTGGGTGACGGCATCTGACAAAAAGACTGCCGTGGA

CATGTCGGGGCAGACGGTAACTGTTCTAGAAAAAGTTCCAGTACCCAAAGGCCAACTGAA

GCAATACTTCTACGAGACCAAATGCAACCCTATGGGTTACATGAAGGAGGGCTGCAGAGG

CATTGACAAGAGGTACTGGAACTCCCAGTGCCGAACTACTCAGTCGTATGTGCGCGCGCT

CACCATGGATAACAAGAAGAAAGTGGGCTGGCGGTTTATCAGAATAGACACTTCATGTGT

ATGTACATTGACCATTGTTTGCTTGGGTTGCAGACCGAAGGCTGTTGTTCTACAAATATG

TCTACAAGCGATATAGAGCTGGGAAGCAAAGGGGTATGATTATAGAAACCGAAGGTGACA

GGCCTTCTTCAAAAGCTGACATTGAAATGGATGGAAAGATGGTCAACTCTCATACTGAAA

ATTTCATTGATGGCTCTCTAGTTCTGGAAGTCGATGAGAAAGATCAGGATGATGAAGAAG

CCAGGCGCGATATGGCCAGAATCCTAAAGGAGCTAAAGCAGAAACATCCTGACAAGGAAA

TAGAGCAACTGATTGAGTTAGCGAACTACCAGGTCTTGAGTCAACAGCAAAAAAGTCGAG

CATTCTATCGTATTCAAGCTACTCGCTTGATGACTGGGGCCGGAAATATTCTAAAGAGAC

ATGCAGCAGACCAGGCAAGGAAAGCTGTGAGCATGCAGGAAGTTAACTCGGAAGTGATCG

AAAATGAACCCGTCAGTAAGATCTATTTCGAGCAAGC

>TY33

????????????????????????????????????????????????????????????

????????????????????????????????????????????????????????????

????????????????????????????????????????????????????????????

????????????????????????????????????????????????????????????

????????????????????????????????????????????????????????????

????????????????????????????????????????????????????????????

????????????????????????????????????????????????????????????

?????????????ATGAGCCCATATGCACTATCAATTCTATTATCGAGCCTAGCAGTAGG

AACAATCACAACCCTTTCAAGCTCCCATTGATTCCTCGCATGAATGGGACTAGAAATTAA

CACACTAGCAATCATCCCACTAATAACAAAAATACACCACCCACGAGCAACAGAATCAGC

TACAAAATACTTCCTAACGCAAGCCACCGCATCAGCTTTAATCTTGTTTTCTACAATCAC

AAACGCATGAGCCACAGGAGAATGAACAATTATTACCATAAATGACAACACATCAACATT

TATCTTAACAATTGCCCTGGCCATCAAACTGGGAATCGCCCCATTTCACCTCTGATTACC

AGACGTCATACAAGGGTTAACCCTAACAACATGTCTAATCCTTACAACATGACAAAAATT

AGCCCCAATATCCTTAATAATTATAACCGGCCATCAATTAAACACAAACCTACTGATTAC

AATGGCCATACTATCAACAGTGTTAGGGGGGTGGGGGGGCCTTAACCAAACACAAATACG

AAAAATAATAGCATATTCATCTATTGCCCACCTCGGATGAATAACACTAATCCTATCCTT

CTCCCCAGACTTAACAACATTAAACCTAATAATTTACCTAATACTAACCTCATCTATATT

CTTAATAATAATAACAATAACCTCAACAAACATAAATAAACTTTCAACATCTTGACTAAA

AACCCCACTACTTGCATCATCAATGATAATTACACTTATAGCCCTAGGAGGCTTACCCCC

AACCTCAGGCTTCTTGCCAAAATGGCTAATCCTACAAGAAATAACCAAACAACACCTGGG

AGCTGTATCCACACTAATAGCTATATCCGCCCTACTAAGCCTATTCTTCTACCTACGACT

ATCATATGCAATCTCATTAACCACTGCACCAAACACCTCAAACTCCAACATAGCCTGACG

AATAACCCACAGCCAAATACAAATCCACCCAACAATAATTACACTAACCACAATAATACT

ACCAATCACACCAACACTACTAGCAATAGGACAAGGCAGCTTGGCGTACCCAGGTCTGAG

GACCCACGGGACTCTGGAGAGCATAGGTGGGCCTAAAGGCAATTCAAGAGGAGGGTTGCC

CTCACTAGCAGACACTTTTGAACAAGTCATTGAGGAGATGCTTGAAGATGAGCAGAGCAT

GAGGCCCAGTGAGGAGAACAAGGATGCGGACATGTTTACATCTCGCGTAATGCTGAGCAG

TCAAGTGCCTTTGGAGCCTCCTCTTCTCTTTCTGCTGGAGGAGTACAAAAACTACTTGGA

TGCTGCAAATATGTCCATGAGGGTCCGACGTCACTCTGACCCCGCCCGCCGCGGGGAGCT

GAGTGTGTGTGACAGTATTAGTGAGTGGGTGACGGCATCTGACAAAAAGACTGCCGTGGA

CATGTCGGGGCAGACGGTAACTGTTCTAGAAAAAGTTCCAGTACCCAAAGGCCAACTGAA

GCAATACTTCTACGAGACCAAATGCAACCCTATGGGTTACATGAAGGAGGGCTGCAGAGG

CATTGACAAGAGGTACTGGAACTCCCAGTGCCGAACTACTCAGTCGTATGTGCGCGCGCT

CACCATGGATAACAAGAAGAAAGTGGGCTGGCGGTTTATCAGAATAGACACTTCATGTGT

ATGTACATTGACCATTGTTTGCTTGGGTTGCAGACCGAAGGCTGTTGTTCTACAAATATG

TCTACAAGCGATATAGAGCTGGGAAGCAAAGGGGTATGATTATAGAAACCGAAGGTGACA

GGCCTTCTTCAAAAGCTGACATTGAAATGGATGGAAAGATGGTCAACTCTCATACTGAAA

ATTTCATTGATGGCTCTCTAGTTCTGGAAGTCGATGAGAAAGATCAGGATGATGAAGAAG

CCAGGCGCGATATGGCCAGAATCCTAAAGGAGCTAAAGCAGAAACATCCTGACAAGGAAA

TAGAGCAACTGATTGAGTTAGCGAACTACCAGGTCTTGAGTCAACAGCAAAAAAGTCGAG

CATTCTATCGTATTCAAGCTACTCGCTTGATGACTGGGGCCGGAAATATTCTAAAGAGAC

ATGCAGCAGACCAGGCAAGGAAAGCTGTGAGCATGCAGGAAGTTAACTCGGAAGTGATCG

AAAATGAACCCGTCAGTAAGATCTATTTCGAGCAAGC

>TY34

CAACGGCCGCGGTATCATGACCGTGCAAAGGTAGCGTAATCACTTGTCTTTTAAATAAAG

ACCTGTATGAAAGGCAAAACGAAAGTTCAACTGTCTCTTTAATCCAATCAATAAAATTTA

TCTTTCCGTGCAGAAGCGGAAATAGACATATAAGACGAGAAGACCCTATGGAGCTTTAAA

CACACTATAATATAAGTTTTAGGTTGGGGCGACCGCGGAAGAAAAAAATCCTCCGAGATA

AGAATAACACTTCGAAAATTAAAACATTTTGATCCACTAAGTGACCAACGAACCAAGTTA

CCCTAGGGATAACAGCGCAATCCTTTCTAAGAGTCCATATCGACGAACGGGTTTACGACC

TCGATGTTGGATCAGGACACCCAAATGGTGCAGCCGCTATTAAGGTTCGTTTGTTCAACG

ATTAAAGTCCTACATGAACCCATATGCACTATCAATTCTATTGTCAAGCCTAGCAGTAGG

AACAATCACAACCCTTTCAAGCTCCCATTGATTCCTCGCATGAGTGGGCCTAGAAATTAA

CACACTAGCAATCATCCCGCTAATAACAAAAATACACCACCCACGAGCAACGGAATCAGC

CACAAAATACTTTCTAACGCAAGCCACCGCATCAGCCTTAATCTTATTTTCTACAATCAC

AAACGCATGGGCCACAGGAGAATGAACAATTATTACCATAAATGACAACACATCAACATT

TATCTTAACAATTGCCCTAGCCATCAAACTTGGAATCGCCCCATTTCACCTCTGATTACC

AGACGTTATACAAGGATTAACCCTAACAACATGTCTAATCCTTACAACATGACAAAAATT

AGCCCCAATATCCTTAATAATTATAACCGGCCATCAATTAAACACAAACCTACTAATTAC

AATGGCCATATTATCAACAGTACTTGGGGGGTGAGGGGGCCTTAACCAAACACAAATACG

AAAAATAATAGCATATTCATCTATTGCCCACCTCGGATGAATAACCCTAATCCTATCCTT

CTCCCCAGACTTAACAACATTAAACCTAGTAATTTACCTAATACTAACCTCATCAATATT

TTTAATAATAATAACAATAACCTCAACAAACATAAATAAACTTTCAACATCTTGACTGAA

GACCCCCCTACTAGCATCATCAATAATAATTACACTTATAGCCCTGGGGGGCCTACCCCC

AACCTCAGGCTTCTTGCCAAAATGACTTATCCTGCAAGAAATAACCAAACAACACCTGGG

AGCTGTATCCACACTAATAGCTATATCTGCCATACTAAGCCTATTCTTCTACCTACGACT

ATCATATGCAGTCTCATTAACCACTGCACCAAACACCTCAAACTCCAACATAGCCTGACG

AATACCCCGCAACCAAATACAAATCAACCCCACAATAATTATACTAACTACAATAATACT

ACCAATCACACCAACACTACTAATAATA????????????????????????????????

????????????????????????????????????????????????????????????

????????????????????????????????????????????????????????????

????????????????????????????????????????????????????????????

????????????????????????????????????????????????????????????

????????????????????????????????????????????????????????????

????????????????????????????????????????????????????????????

????????????????????????????????????????????????????????????

????????????????????????????????????????????????????????????

????????????????????????????????????????????????????????????

????????????????????????????????????????????????????????????

????????????????GTTTGCTTGGGTTGCAGACCGAAGGCTGTTGTTCTACAAATATG

TCTACAAGCGATATAGAGCTGGGAAGCAAAGGGGTATGATTATAGAAACCGAAGGTGACA

GGCCTTCTTCAAAAGCTGACATTGAAATGGATGGAAAGATGGTCAACTCTCATACTGAAA

ATTTCATTGATGGCTCTCTAGTTCTGGAAGTCGATGAGAAAGATCAGGATGATGAAGAAG

CCAGGCGCGATATGGCCAGAATCCTAAAGGAGCTAAAGCAGAAACATCCTGACAAGGAAA

TAGAGCAACTGATTGAGTTAGCGAACTACCAGGTCTTGAGTCAACAGCAAAAAAGTCGAG

CATTCTATCGTATTCAAGCTACTCGCTTGATGACTGGGGCCGGAAATATTCTAAAGAGAC

ATGCAGCAGACCAGGCAAGGAAAGCTGTGAGCATGCAGGAAGTTAACTCTGAAGTGATCG

AAAATGAGCCCGTCAGTAAGATCTATTTCGAGCAAGC

>TY35

????????????????????????????????????????????????????????????

????????????????????????????????????????????????????????????

????????????????????????????????????????????????????????????

????????????????????????????????????????????????????????????

????????????????????????????????????????????????????????????

????????????????????????????????????????????????????????????

????????????????????????????????????????????????????????????

?????????????ATGAACCCATATGCACTGTCAATTCTATTGTCGAGCCTAGCAGTAGG

AACAATCACAACCCTTTCAAGCTCCCATTGATTCCTCGCATGAGTGGGCCTAGAAATTAA

CACACTAGCAATCATCCCACTAATAACAAAAATACACCACCCACGAGCAACGGAATCAGC

CACAAAATACTTTCTAACGCAAGCCACCGCATCAGCCTTAATCTTATTTTCTACAATCAC

AAACGCATGAGCCACAGGAGAGTGAACAATTATTACCATAAATGACAACACGTCAACATT

TATCTTAACAATTGCCCTGGCCATCAAACTGGGAATCGCCCCATTTCACCTCTGATTACC

AGACGTTATACAAGGATTAACCCTAACAACATGTCTAATCCTTACAACATGACAAAAATT

AGCCCCAATATCCTTAATAATTATAACCGGCCATCAATTAAACACAAACCTACTAATTAC

AATGGCCATATTATCAACAATTCTTGGGGGGTGGGGAGGCCTTAACCAAACACAAATACG

AAAAATAATAGCATACTCATCTATTGCCCACCTCGGATGAATAACCCTAATCCTATCCTT

CTCCCCAGGCCTAACAGCATTAAACCTAGCAATTTACCTAATACTAACCTCATCAATATT

TTTAATAATAATAACAATAACCTCAACAAATATAAATAAACTTTCAACATCTTGACTGAA

GACCCCCCTACTGGCATCATCAATGATAATTACACTTATAGCCCTAGGGGGCCTACCCCC

AACCTCAGGCTTTTTGCCAAAATGACTAATCCTACAAGAAATAACCAAACAACACCTAGG

AGCTGTATCCACACTAATAGCTATATCTGCCCTACTAAGCCTATTCTTCTACCTACGACT

ATCATATGCCGTCTCATTAACCACTGCACCAAACACCTCAAACTCCAACATAGCCTGACG

AATACCCCACAACCAAATACAAATCAACCCAACAATAATTACACTAACTATAATAATACT

ACCAATCACACCAACACTACTAACAATA????????????????????????????????

????????????????????????????????????????????????????????????

????????????????????????????????????????????????????????????

????????????????????????????????????????????????????????????

????????????????????????????????????????????????????????????

????????????????????????????????????????????????????????????

????????????????????????????????????????????????????????????

????????????????????????????????????????????????????????????

????????????????????????????????????????????????????????????

????????????????????????????????????????????????????????????

????????????????????????????????????????????????????????????

????????????????GTTTGCTTGGGTTGCAGACCGAAGGCTGTTGTTCTACAAATATG

TCTACAAGCGATATAGAGCTGGGAAGCAAAGGGGTATGATTATAGAAACCGAAGGTGACA

GGCCTTCTTCAAAAGCTGACATTGAAATGGATGGAAAGATGGTCAACTCTCATACTGAAA

ATTTCATTGATGGCTCTCTAGTTCTGGAAGTCGATGAGAAAGATCAGGATGATGAAGAAG

CCAGGCGCGATATGGCCAGAATCCTAAAGGAGCTAAAGCAGAAACATCCTGACAAGGAAA

TAGAGCAACTGATTGAGTTAGCGAACTACCAGGTCTTGAGTCAACAGCAAAAAAGTCGAG

CATTCTATCGTATTCAAGCTACTCGCTTGATGACTGGGGCCGGAAATATTCTAAAGAGAC

ATGCAGCAGACCAGGCAAGGAAAGCTGTGAGCATGCAGGAAGTTAACTCTGAAGTGATCG

AAAATGAGCCCGTCAGTAAGATCTATTTCGAGCAAGC

>TY36

CAACGGCCGCGGTATCATGACCGTGCAAAGGTAGCGTAATCACTTGTCTTTTAAATAAAG

ACCTGTATGAAAGGCAAAACGAAAGTTCAACTGTCTCTTTAATCCAATCAATGAAATTTA

TCTTTTCGTGCAGAAGCGGAAATAAATATATAAGACGAGAAGACCCTATGGAGCTTTAAA

CACACTATAATATAAGTTTTGGGTTGGGGCGACCACGGAGAAAAGAAATCCTCCGAGATA

AGAGTAACACTTCAAAAATTAAAACATTTTGATCCACTAAGTGACCAACGAACCAAGTTA

CCCTAGGGATAACAGCGCAATCCTTTCTAAGAGTCCATATCGACGAATGGGTTTACGACC

TCGATGTTGGATCAGGACACCCAAATGGTGCAGCCGCTATTAAGGTTCGTTTGTTCAACG

ATTAAAGTCCTACATGAACCCATATGCACTATCAATCCTGTTGTCGAGCCTAGCAATAGG

AACAATTACAACCCTCTCAAGCTCCCATTGATTTCTGGCATGAATAGGGCTAGAAATTAA

TACACTAGCAATAATCCCGCTAATAACAAAAATACACCACCCACGATCAACAGAATCAGC

CACAAAATACTTCCTAACGCAAGCCGCCGCATCAGCCTTAATCCTATTTTCTACAATCAC

AAACGCATGGGCCACAGGAGAATGAACAATTATCACCATAAATGACAACCTATCAACATT

TATATTAACAATTGCCCTAGCCATTAAACTTGGAGTCGCCCCTTTTCATCTATGACTACC

AGACGTCATACAAGGGCTAAACCTAACAACATGCCTAATCCTTGCAACATGACAAAAATT

AGCCCCAATATCCTTAATAATTATAACCAGCCACCAATTAAACACAAACCTGCTAATTAC

AATAGCCGTATTATCAGCAGTAATTGGAGGATGAGGGGGACTTAACCAAACACAAATCCG

AAAGATAATAGCATATTCATCTATCGCCCACCTCGGATGAATAACACTAGTCCTATCTTT

CTCCCCAAATTTAACAACCCTTAACCTAATAATTTACCTAATACTAACATCATCAATGTT

CTTAATAATAACAACCCTAAACTCAACAAATATTAACAAACTTTCAACATCTTGACTGAA

GACCCCACTACTAGCATCATCAATAATAATTACACTCATGGCCCTAGGGGGCCTACCCCC

CACATCAGGATTCCTACCAAAATGACTTATCCTACAAGAAATAACCAAACAGCACCTAGG

AGCTGTTTCCACACTAATAGCTATATCTGCACTACTAAGCCTATTCTTCTACCTACGACT

ATCATATGCGATCTCACTAACTACCGCACCAAACACATCAAGCTCCAACATAACCTGACG

AATAGCCCACAACCAAATACAAATCCTACCAATAATAATTACACTAACTACAATAATACT

ACCGATCACACCAACACTACTAACAATAGGACAAGGCAGCTTGGCGTACCCAGGTCTGAG

GACCCACGGGACTTTGGAGAGCATAGGTGGGCCTAAAGGCAGTTCAAGAGGAGGGTTGCC

CTCACTAGCAGACACTTTTGAACAAGTCATTGAGGAGATGCTTGAAGATGAGCAGAGCAT

GAGGCCCAGTGAGGAGAACAAGGATGCGGACATGTTTACATCTCGCGTAATGCTGAGCAG

TCAAGTGCCTTTGGAGCCTCCTCTTCTCTTTCTGCTGGAGGAGTACAAAAACTACTTGGA

TGCTGCAAATATGTCCATGAGGGTCCGACGTCACTCTGACCCCGCCCGCCGCGGGGAGCT

GAGTGTGTGTGACAGTATTAGTGAGTGGGTGACGGCATCTGACAAAAAGACTGCCGTGGA

CATGTCGGGGCAGACGGTAACTGTTCTAGAAAAAGTTCCAGTACCCAAAGGCCAACTGAA

GCAATACTTCTACGAGACCAAATGCAACCCTATGGGTTACATGAAGGAGGGCTGCAGAGG

CATTGACAAGAGGTACTGGAACTCCCAGTGCCGAACTACTCAGTCGTATGTGCGCGCGCT

CACCATGGATAACAAGAAGAAAGTGGGCTGGCGGTTTATCAGAATAGACACTTCATGTGT

ATGTACATTGACCATTGTTTGCTTGGGTTGCAGACCGAAGGCTGTTGTTCTACAAATATG

TCTACAAGCGATATAGAGCTGGGAAGCAAAGGGGTATGATTATAGAAACCGAAGGTGACA

GGCCTTCTTCAAAAGCTGACATTGAAATGGATGGAAAGATGGTCAACTCTCATACTGAAA

ATTTCATTGATGGCTCTCTAGTTCTGGAAGTCGATGAGAAAGATCAGGATGATGAAGAAG

CCAGGCGCGATATGGCCAGAATCCTAAAGGAGCTAAAGCAGAAACATCCTGACAAGGAAA

TAGAGCAACTGATTGAGTTAGCGAACTACCAGGTCTTGAGTCAACAGCAAAAAAGTCGAG

CATTCTATCGTATTCAAGCTACTCGCTTGATGACTGGGGCCGGAAATATTCTAAAGAGAC

ATGCAGCAGACCAGGCAAGGAAAGCTGTGAGCATGCAGGAAGTTAACTCTGAAGTGATCG

AAAATGAGCCCGTCAGTAAGATCTATTTCGAGCAAGC

>TY38

????????????????????????????????????????????????????????????

????????????????????????????????????????????????????????????

????????????????????????????????????????????????????????????

????????????????????????????????????????????????????????????

????????????????????????????????????????????????????????????

????????????????????????????????????????????????????????????

????????????????????????????????????????????????????????????

?????????????ATGAACCCATATGCCCTGTCAATTCTATTATCGAGCCTAGCAGTAGG

AACTATCACAACCCTTTCAAGCTCCCATTGATTCCTCGCATGGGTGGGCCTAGAAATTAA

CACACTAGCAATCATCCCGCTAATAACAAAAATACACCACCCACGAGCAACAGAATCAGC

CACAAAATACTTCCTAACGCAAGCCACCGCATCAGCCTTAATCTTATTTTCTACAATCAC

AAATGCATGAGCCACAGGAGAATGAACAATTATTACCATAAACGACAACACATCAACATT

TATCTTAACAATTGCCCTAGCCATTAAACTGGGGATCGCCCCATTTCACCTCTGATTGCC

AGACGTCATACAAGGATTAACCCTAACGACATGTCTTATCCTTACAACATGACAAAAATT

AGCCCCAATATCCTTAATAATTATAGCCAGCCATCAATTAAACACAAACCTGCTAATTAC

AATGGCCATATTATCAACAGTTCTAGGAGGGTGGGGAGGCCTTAACCAAACACAAATACG

AAAAATAATAGCATATTCATCTGTTGCCCACCTCGGATGAATAACACTAATCCTATCCTT

CTCCCCAAGCTTAACAACATTAAACCTAATAATTTACCTAATACTAACCTCATCAATATT

CTTAATAATAATAACAATAACCTCAACAAATATAAATAAACTTTCAACATCTTGACTAAA

GACCCCCCTACTGGCATCATCAATAATAATTACACTAATAGCCCTGGGGGGCCTACCCCC

AACCTCAGGCTTCTTGCCAAAATGACTAATTCTACAAGAAATAACCAAACAACACCTGGG

AGCTGTATCCACACTAATAGCTATATCTGCACTACTAAGCCTATTCTTCTACCTACGACT

ATCATATGCAGTCTCATTAACCACTGCACCAAACACCTCAAACTCCAACATGGCCTGACG

AATAACCCACAACCAAATACAAATACACCCCACAATAATTACACTAACTACAATAATACT

ACCAATCACACCAACACTACTAACGATAGGACAAGGCAGCTTGGCGTACCCAGGTCTGAG

GACCCACGGGACTTTGGAGAGCATAGGTGGGCCTAAAGGCAGTTCAAGAGGAGGGTTGCC

CTCACTAGCAGACACTTTTGAACAAGTCATTGAGGAGATGCTTGAAGATGAGCAGAGCAT

GAGGCCCAGTGAGGAGAACAAGGATGCGGACATGTTTACATCTCGCGTAATGCTGAGCAG

TCAAGTGCCTTTGGAGCCTCCTCTTCTCTTTCTGCTGGAGGAGTACAAAAACTACTTGGA

TGCTGCAAATATGTCCATGAGGGTCCGACGTCACTCTGACCCCGCCCGCCGCGGGGAGCT

GAGTGTGTGTGACAGTATTAGTGAGTGGGTGACGGCATCTGACAAAAAGACTGCCGTGGA

CATGTCGGGGCAGACGGTAACTGTTCTAGAAAAAGTTCCAGTACCCAAAGGCCAACTGAA

GCAATACTTCTACGAGACCAAATGCAACCCTATGGGTTACATGAAGGAGGGCTGCAGAGG

CATTGACAAGAGGTACTGGAACTCCCAGTGCCGAACTACTCAGTCGTATGTGCGCGCGCT

CACCATGGATAACAAGAAGAAAGTGGGCTGGCGGTTTATCAGAATAGACACTTCATGTGT

ATGTACATTGACCATTGTTTGCTTGGGTTGCAGACCGAAGGCTGTTGTTCTACAAATATG

TCTACAAGCGATATAGAGCTGGGAAGCAAAGGGGTATGATTATAGAAACCGAAGGTGACA

GGCCTTCTTCAAAAGCTGACATTGAAATGGATGGAAAGATGGTCAACTCTCATACTGAAA

ATTTCATTGATGGCTCTCTAGTTCTGGAAGTCGATGAGAAAGATCAGGATGATGAAGAAG

CCAGGCGCGATATGGCCAGAATCCTAAAGGAGCTAAAGCAGAAACATCCTGACAAGGAAA

TAGAGCAACTGATTGAGTTAGCGAACTACCAGGTCTTGAGTCAACAGCAAAAAAGTCGAG

CATTCTATCGTATTCAAGCTACTCGCTTGATGACTGGGGCCGGAAATATTCTAAAGAGAC

ATGCAGCAGACCAGGCAAGGAAAGCTGTGAGCATGCAGGAAGTTAACTCTGAAGTGATCG

AAAATGAGCCCGTCAGTAAGATCTATTTCGAGCAAGC

>TY39

CAACGGCCGCGGTATCATGACCGTGCAAAGGTAGCGTAATCACTTGTCTTTTAAATAAAG

ACCTGTATGAAAGGCAAAACGAAAGTTCAACTGTCTCTTTAATCCAATCAATGAAATTTA

TCTTTTCGTGCAGAAGCGGAAATAAATATATAAGACGAGAAGACCCTATGGAGCTTTAAA

CACACTATAATATAAGTTTTGGGTTGGGGCGACCACGGAGAAAAGAAATCCTCCGAGATA

AGAATAACAATTCGAAAATTAAAACATTTTGATCCACTAAGTGACCAACGAACCAAGTTA

CCCTAGGGATAACAGCGCAATCCTTTCTAAGAGTCCATATCGACGAATGGGTTTACGACC

TCGATGTTGGATCAGGACACCCAAATGGTGCAGCCGCTATTAAGGTTCGTTTGTTCAACG

ATTAAAGTCCTACATGAACCCATATGCACTATCAATTCTATTATCTAGCCTAGCAGTAGG

AACTATCACAACCCTTTCAAGCTCCCATTGATTCCTCGCATGAGTAGGCCTAGAAATCAA

CACACTAGCAATCATCCCACTAATAACAAAAATACACCACCCACGAGCAACAGAATCAGC

AACAAAATACTTCCTAACGCAAGCCACCGCATCAGCCTTAATCTTATTTTCCACAATCAC

AAATGCATGAGCCACAGGAGAATGAACAATTATTACCATAAATGACAACACATCAACATT

TATCTTAACAATTGCCCTGGCCATTAAACTGGGAATCGCCCCATTTCACCTCTGATTGCC

AGACGTCATACAAGGATTAACCCTAACGACATGTCTTATCCTTACAACATGACAAAAATT

AGCCCCAATATCCTTAATAATTATAACCAGCCATCAATTAAACACAAACCTATTAATTAC

AATGGCCATATTATCAACAGTACTTGGGGGGTGGGGGGGCCTTAACCAAACACAAATACG

AAAAATAATAGCATATTCATCTATTGCCCACCTCGGATGAATAACACTAATCCTATCCTT

CTCCCCAAAATTAACAACATTAAACCTAATAATTTACCTAATACTAACCTCATCAATATT

CTTAATAATAATAACAATAACCTCAACAAATATAAATAAACTTTCAACATCTTGACTAAA

GACCCCCATACTGGCATCATCAATAATAATTACACTTATAGCCCTGGGGGGCCTACCCCC

AACCTCAGGCTTCTTGCCAAAATGACTAATTCTACAAGAAATAACCAAACAACACCTGGG

AGCTGTATCCACACTGATAGCTATATCTGCACTACTAAGCCTATTCTTCTACCTACGACT

ATCATATGCAGTCTCACTAACCACTGCACCAAACACCTCAAACTCCAACATGGCCTGACG

AATAACCCACAACCAAATACAAATACACCCCACAATAATTACACTAACTACAATAATACT

ACCAATCACACCAACACTACTAACGATAGGACAAGGCAGCTTGGCGTACCCAGGTCTGAG

GACCCACGGGACTTTGGAGAGCATAGGTGGGCCTAAAGGCAGTTCAAGAGGAGGGTTGCC

CTCACTAGCAGACACTTTTGAACAAGTCATTGAGGAGATGCTTGAAGATGAGCAGAGCAT

GAGGCCCAGTGAGGAGAACAAGGATGCGGACATGTTTACATCTCGCGTAATGCTGAGCAG

TCAAGTGCCTTTGGAGCCTCCTCTTCTCTTTCTGCTGGAGGAGTACAAAAACTACTTGGA

TGCTGCAAATATGTCCATGAGGGTCCGACGTCACTCTGACCCCGCCCGCCGCGGGGAGCT

GAGTGTGTGTGACAGTATTAGTGAGTGGGTGACGGCATCTGACAAAAAGACTGCCGTGGA

CATGTCGGGGCAGACGGTAACTGTTCTAGAAAAAGTTCCAGTACCCAAAGGCCAACTGAA

GCAATACTTCTACGAGACCAAATGCAACCCTATGGGTTACATGAAGGAGGGCTGCAGAGG

CATTGACAAGAGGTACTGGAACTCCCAGTGCCGAACTACTCAGTCGTATGTGCGCGCGCT

CACCATGGATAACAAGAAGAAAGTGGGCTGGCGGTTTATCAGAATAGACACTTCATGTGT

ATGTACATTGACCATTGTTTGCTTGGGTTGCAGACCGAAGGCTGTTGTTCTACAAATATG

TCTACAAGCGATATAGAGCTGGGAAGCAAAGGGGTATGATTATAGAAACCGAAGGTGACA

GGCCTTCTTCAAAAGCTGACATTGAAATGGATGGAAAGATGGTCAACTCTCATACTGAAA

ATTTCATTGATGGCTCTCTAGTTCTGGAAGTCGATGAGAAAGATCAGGATGATGAAGAAG

CCAGGCGCGATATGGCCAGAATCCTAAAGGAGCTAAAGCAGAAACATCCTGACAAGGAAA

TAGAGCAACTGATTGAGTTAGCGAACTACCAGGTCTTGAGTCAACAGCAAAAAAGTCGAG

CATTCTATCGTATTCAAGCTACTCGCTTGATGACTGGGGCCGGAAATATTCTAAAGAGAC

ATGCAGCAGACCAGGCAAGGAAAGCTGTGAGCATGCAGGAAGTTAACTCTGAAGTGATCG

AAAATGAGCCCGTCAGTAAGATCTATTTCGAGCAAGC

>TY40

CAACGGCCGCGGTATCATGACCGTGCAAAGGTAGCGTAATCACTTGTCTTTTAAATAAAG

ACCTGTATGAAAGGCAAAACGAAAGTTCAACTGTCTCTTTAATCCAATCAATGAAATTTA

TCTTTTCGTGCAGAAGCGGAAATAAATATATAAGACGAGAAGACCCTATGGAGCTTTAAA

CACGCTATAATACAAGTTTTTGGTTGGGGCGACCGCGGAGAAAAGAAATCCTCCGAGATA

AGAATAACACTTCAAAAATTAAAACATTTTGATCCACTAAGTGACCAACGAACCAAGTTA

CCCTAGGGATAACAGCGCAATCCTTTCTAAGAGTCCATATCGACGAATGGGTTTACGACC

TCGATGTTGGATCAGGACACCCAAATGGTGCAGCCGCTATTAAGGTTCGTTTGTTCAACG

ATTAAAGTCCTACATGAACCCGTATGCACTATCAATCCTGTTGTCGAGCCTAGCAGTAGG

AACAATTACAACTCTCTCAAGCTCCCATTGATTTCTGGCATGAGTAGGACTAGAAATTAA

TACACTAGCAATAATCCCGCTAATAACAAAAATACACCACCCACGATCAACAGAATCAGC

CACAAAATACTTCCTAACGCAAGCCGCCGCATCAGCCTTAATCTTATTTTCTACAATCAT

AAACGCATGGGCTACAGGAGAATGAACAATTATCACCATAAATGACAACTCATCAACATT

TATATTAACAGTTGCCCTAGCTATTAAACTTGGAATCGCCCCTTTTCATCTATGACTACC

AGACGTCATACAAGGGTTAAACCTAACAACATGCCTAATCCTTGCAACATGACAAAAATT

AGCCCCAATATCCTTAATAATTATAGCCAATCACCAACTAAACACAAACCTGCTAATTAC

AATAGCCGTATTATCAGCAGCAATTGGGGGGTGGGGGGGACTTAACCAAACACAAATCCG

AAAAATAATAGCATATTCATCTATCGCCCACCTTGGGTGAATAACACTAGTCCTCTCTTT

CTCCCCAAATTTAACAACCCTTAACCTAATAATTTACCTAATACTAACATCATCAATGTT

CTTAATAATAACAACCCTAAACTCAACAAATATTAACAAACTTTCAACATCTTGACTAAA

AACCCCACTACTAGCATCATCAATAATAATTACACTCATAGCCCTAGGAGGCCTACCCCC

CACATCAGGATTCCTACCAAAATGACTTATCCTACAAGAAATAACCAAACAACACCTAGG

AGCCGTGTCCACACTAATAGCTATATCTGCACTACTAAGCCTATTCTTCTACTTACGACT

ATCATATGCAATCTCACTAACTACCGCACCAAACACATCAAACTCCAACATAACCTGACG

AATAGCCCACAACCAAATACAAATCCTACCAACAATAATCACACTAACTACAATAATACT

ACCGATCACACCAACACTACTAGCAATAGGACAAGGCAGCTTGGCGTACCCAGGTCTGAG

GACCCACGGGACTTTGGAGAGCATAGGTGGGCCTAAAGGCAGTTCAAGAGGAGGGTTGCC

CTCACTAGCAGACACTTTTGAACAAGTCATTGAGGAGATGCTTGAAGATGAGCAGAGCAT

GAGGCCCAGTGAGGAGAACAAGGATGCGGACATGTTTACATCTCGCGTAATGCTGAGCAG

TCAAGTGCCTTTGGAGCCTCCTCTTCTCTTTCTGCTGGAGGAGTACAAAAACTACTTGGA

TGCTGCAAATATGTCCATGAGGGTCCGACGTCACTCTGACCCCGCCCGCCGCGGGGAGCT

GAGTGTGTGTGACAGTATTAGTGAGTGGGTGACGGCATCTGACAAAAAGACTGCCGTGGA

CATGTCGGGGCAGACGGTAACTGTTCTAGAAAAAGTTCCAGTACCCAAAGGCCAACTGAA

GCAATACTTCTACGAGACCAAATGCAACCCTATGGGTTACATGAAGGAGGGCTGCAGAGG

CATTGACAAGAGGTACTGGAACTCCCAGTGCCGAACTACTCAGTCGTATGTGCGCGCGCT

CACCATGGATAACAAGAAGAAAGTGGGCTGGCGGTTTATCAGAATAGACACTTCATGTGT

ATGTACATTGACCATTGTTTGCTTGGGTTGCAGACCGAAGGCTGTTGTTCTACAAATATG

TCTACAAGCGATATAGAGCTGGGAAGCAAAGGGGTATGATTATAGAAACCGAAGGTGACA

GGCCTTCTTCAAAAGCTGACATTGAAATGGATGGAAAGATGGTCAACTCTCATACTGAAA

ATTTCATTGATGGCTCTCTAGTTCTGGAAGTTGATGAGAAAGATCAGGATGATGAAGAAG

CCAGGCGCGATATGGCCAGAATCCTAAAGGAGCTAAAGCAGAAACATCCTGACAAGGAAA

TAGAGCAACTGATTGAGTTAGCGAACTACCAGGTCTTGAGTCAACAGCAAAAAAGTCGAG

CATTCTATCGTATTCAAGCTACTCGCTTGATGACTGGGGCCGGAAATATTCTAAAGAGAC

ATGCAGCAGACCAGGCAAGGAAAGCTGTGAGCATGCAGGAAGTTAACTCTGAAGTGATCG

AAAATGAGCCCGTCAGTAAGATCTATTTCGAGCAAGC

>LC017829

????????????????????????????????????????????????????????????

????????????????????????????????????????????????????????????

????????????????????????????????????????????????????????????

????????????????????????????????????????????????????????????

????????????????????????????????????????????????????????????

????????????????????????????????????????????????????????????

????????????????????????????????????????????????????????????

?????????????ATGAACCCATATGCACTATCAATCCTGTTGTCGAGCCTAGCAGTAGG

AACAATTACAACCCTCTCAAGCTCCCATTGATTTCTGGCATGAATAGGGCTAGAAATTAA

TACACTAGCAATAATCCCGCTAATAACAAAAATACACCACCCACGATCAACAGAATCAGC

CACAAAATACTTCCTAACGCAAGCCGCCGCATCAGCCTTAATCCTATTTTCTACAATCAC

AAACGCATGGGCCACAGGAGAATGAACAATTATTACCATAAATGACAACCTATCAACATT

TATATTAACAATTGCCCTAGCCATTAAACTTGGAGTCGCCCCTTTTCATCTATGACTACC

AGACGTCATACAAGGGCTAAACCTAACAACATGCCTAATCCTTGCAACATGACAAAAATT

AGCCCCAATATCCTTAATAATTATAACCAGCCACCAATTAAACACAAACCTACTAATTAC

AATAGCCGTATTATCAGCAGTAATTGGGGGGTGGGGGGGACTTAACCAAACACAAATCCG

AAAGATAATAGCATATTCATCTATCGCCCACCTCGGATGAATAACACTAGTCCTATCTTT

CTCCCCAAATTTAACAACCCTTAACCTAATAATTTACCTAATACTAACATCATCAATGTT

CTTAATAATAACAACCCTAAACTCAACAAATATTAACAAACTTTCAACATCTTGACTGAA

GACCCCACTACTAGCATCATCAATAATAATTACACTCATGGCCCTAGGGGGCCTACCCCC

CACATCAGGATTCCTACCAAAATGACTTATCCTACAAGAAATAACCAAACAGCACCTAGG

AGCTGTTTCCACACTAATAGCTATATCTGCACTACTAAGCCTATTCTTCTACCTACGACT

ATCATATACGATCTCACTAACTACCGCACCAAACACATCAAGCTCCAACATAACCTGACG

AATAGCCCACAACCAAATACAAATCCTACCAATAATAATTACACTAACTACAATAATACT

ACCGATCACACCAACACTACTAACAATA????????????????????????????????

????????????????????????????????????????????????????????????

????????????????????????????????????????????????????????????

????????????????????????????????????????????????????????????

????????????????????????????????????????????????????????????

????????????????????????????????????????????????????????????

????????????????????????????????????????????????????????????

????????????????????????????????????????????????????????????

????????????????????????????????????????????????????????????

????????????????????????????????????????????????????????????

????????????????????????????????????????????????????????????

????????????????????????????????????????????????????????????

????????????????????????????????????????????????????????????

????????????????????????????????????????????????????????????

????????????????????????????????????????????????????????????

????????????????????????????????????????????????????????????

????????????????????????????????????????????????????????????

????????????????????????????????????????????????????????????

????????????????????????????????????????????????????????????

?????????????????????????????????????

>LC017830

????????????????????????????????????????????????????????????

????????????????????????????????????????????????????????????

????????????????????????????????????????????????????????????

????????????????????????????????????????????????????????????

????????????????????????????????????????????????????????????

????????????????????????????????????????????????????????????

????????????????????????????????????????????????????????????

?????????????ATGAACCCATATGCACTATCAATCCTGTTGTCGAGCCTAGCAGTAGG

AACAATTACAACCCTCTCAAGCTCCCATTGATTTCTGGCATGAATAGGGCTAGAAATTAA

TACACTAGCAATAATCCCGCTAATAACAAAAATACACCACCCACGATCAACAGAATCAGC

CACAAAATACTTCCTAACGCAAGCCGCCGCATCAGCCTTAATCCTATTTTCTACAATCAC

AAACGCATGGGCCACAGGAGAATGAACAATTATCACCATAAATGACAACCTATCAACATT

TATATTAACAATTGCCCTAGCCATTAAACTTGGAGTCGCCCCTTTTCATCTATGACTACC

AGACGTCATACAAGGGCTAAACCTAACAACATGCCTAATCCTTGCAACATGACAAAAATT

AGCCCCAATATCCTTAATAATTATAACCAGCCACCAATTAAACACAAACCTACTAATTAC

AATAGCCGTATTATCAGCAGTAATTGGAGGATGAGGGGGACTTAACCAAACACAAATCCG

AAAGATAATAGCATATTCATCTATCGCCCACCTCGGATGAATAACACTAGTCCTATCTTT

CTCCCCAAATTTAACAACCCTTAACCTAATAATTTACCTAATACTAACATCATCAATGTT

CTTAATAATAACAACCCTAAACTCAACAAATATTAACAAACTTTCAACATCTTGACTGAA

GACCCCACTACTAGCATCATCAATAATAATTACACTCATGGCCCTAGGGGGCCTACCCCC

CACATCAGGATTCCTACCAAAATGACTTATCCTACAAGAAATAACCAAACAGCACCTAGG

AGCTGTTTCCACACTAATAGCTATATCTGCACTACTAAGCCTATTCTTCTACCTACGACT

ATCATATGCGATCTCACTAACTACCGCACCAAACACATCAAGCTCCAACATAACCTGACG

AATAGCCCACAACCAAATACAAATCCCACCAATAATAATTACACTAACTACAATAATACT

ACCGATCACACCAACACTACTAACAATA????????????????????????????????

????????????????????????????????????????????????????????????

????????????????????????????????????????????????????????????

????????????????????????????????????????????????????????????

????????????????????????????????????????????????????????????

????????????????????????????????????????????????????????????

????????????????????????????????????????????????????????????

????????????????????????????????????????????????????????????

????????????????????????????????????????????????????????????

????????????????????????????????????????????????????????????

????????????????????????????????????????????????????????????

????????????????????????????????????????????????????????????

????????????????????????????????????????????????????????????

????????????????????????????????????????????????????????????

????????????????????????????????????????????????????????????

????????????????????????????????????????????????????????????

????????????????????????????????????????????????????????????

????????????????????????????????????????????????????????????

????????????????????????????????????????????????????????????

?????????????????????????????????????

>AB769544

????????????????????????????????????????????????????????????

????????????????????????????????????????????????????????????

????????????????????????????????????????????????????????????

????????????????????????????????????????????????????????????

????????????????????????????????????????????????????????????

????????????????????????????????????????????????????????????

????????????????????????????????????????????????????????????

?????????????ATGAATCCATATGCACTATCAATCCTGTTGTCGAGCCTAGCAGTAGG

AACACTCACAACTCTCTCAAGCTCCCATTGATTCCTGGCATGAGTAGGACTAGAAATTAA

CACAATAGCAATAATCCCGTTGATAACAAAAATACACCACCCACGATCAACAGAATCAGC

CACAAAATACTTCCTAACGCAAGCCACCGCATCAGCCTTAATCTTATTTTCTACAATCAC

AAACGCATGAGCCACTGGAGAATGAACAATTACCACCATAAATGACAACACATCAACATT

TATACTAACAATTGCCCTAGCTATTAAACTTGGAATCGCCCCTTTTCATCTATGACTACC

AGACGTTATACAAGGACTAAATCTAACAACATGCCTAATCCTTGCGACATGGCAAAAATT

AGCCCCAATGTCCTTAATAATTATAACCAACCACCAATTAAACACAAACATGCTAATTAC

AATAGCCGTGTTATCAACAGTAATTGGCGGATGAGGGGGCCTTAACCAAACACAAATTCG

AAAAATAATAGCATACTCATCTATCGCCCACCTCGGATGAATAACACTAGTCCTATCTTT

CTCCCCAAATTTAACAACCCTTAACCTAATAATCTACCTAATACTAACATCATCAATATT

CTTAATAATAAAAACCCTAAACTCAACAAATATTAACAAACTTTCAATATCTTGACTGAA

AGCCCCCCTATTAGCATCATCAATAATAATTACACTCATAGCCCTAGGAGGTCTACCCCC

AACATCAGGGTTCCTACCAAAATGACTTATCCTACAAGAAATAACCAAACAACACCTAGG

CACCGTATCCACACTAATAGCTATATCTGCACTACTCAGTTTATTCTTCTACCTACGACT

ATCATATGCAATCTCACTAACTACCACACCAAACACCTCAAACTCCAACATAACCTGACG

AATAACCCACAGCCAAATACAAATCCTACCAACAATAATTACACTAACTACAATAATACT

ACCGATCACACCAACACTACTAGCAATA????????????????????????????????

????????????????????????????????????????????????????????????

????????????????????????????????????????????????????????????

????????????????????????????????????????????????????????????

????????????????????????????????????????????????????????????

????????????????????????????????????????????????????????????

????????????????????????????????????????????????????????????

????????????????????????????????????????????????????????????

????????????????????????????????????????????????????????????

????????????????????????????????????????????????????????????

????????????????????????????????????????????????????????????

????????????????????????????????????????????????????????????

????????????????????????????????????????????????????????????

????????????????????????????????????????????????????????????

????????????????????????????????????????????????????????????

????????????????????????????????????????????????????????????

????????????????????????????????????????????????????????????

????????????????????????????????????????????????????????????

????????????????????????????????????????????????????????????

?????????????????????????????????????

>AB830727

????????????????????????????????????????????????????????????

????????????????????????????????????????????????????????????

????????????????????????????????????????????????????????????

????????????????????????????????????????????????????????????

????????????????????????????????????????????????????????????

????????????????????????????????????????????????????????????

????????????????????????????????????????????????????????????

?????????????ATGAACCCATATGCACTATCAATCCTGTTGTCAAGCCTAGCAGTAGG

AACAATTACAACTCTCTCAAGCTCCCATTGATTTCTGGCATGAGTAGGACTAGAAATTAA

TACACTAGCAATAATCCCGCTAATAACAAAAATACACCACCCACGATCAACAGAATCAGC

CACAAAATACTTCCTAACGCAAGCCACCGCATCAGCCTTAATTTTATTTTCTACAACCAT

AAACGCATGGACTACAGGAGAATGAACAATTATCACCATAAATGACAACTCATCAACATT

TATATTAACAGCTGCCCTAGCTATTAAACTTGGACTCGCCCCTTTTCATCTGTGACTACC

AGACGTCATACAAGGATTAAACCTAACAACATGCCTAATCCTTGCAACATGACAAAAATT

AGCCCCAATATCCTTAATAATTATAACCAACCACCAATTAAACACAAACCTTCTAATTAC

AATAGCCGTATTATCAACAGTAATTGGGGGGTGGGGGGGACTTAACCAAACACAAATCCG

AAAAATAATAGCATATTCATCTATCGCCCACCTTGGATGAATAACACTAGTCCTCTCTTT

CTCCCCAAACTTAACAACCCTTAACCTAATAATTTACCTAATATTAACATCATCAATGTT

CTTAATAATAACAACCCTAAACTCAACAAATATTAACAAACTTTCAACATCTTGACTGAA

GACCCCACTACTAACATCATCAATAATAATTACACTAATGGCCCTAGGAGGCCTACCCCC

CACAACAGGATTCCTACCAAAATGACTCATCCTACAAGAAATAACCAAACAACACCTAGG

AGCCGTATCCGCACTAATAGCCATATCTGCACTACTAAGCCTATTCTTCTACTTACGACT

ATCATATGCAATCTCGCTAACTACCGCACCAAACACATCAAACTCCAACATAACCTGACG

AATAACCCACAACCAAATAAAAATCCTACCAACAATAATTACACTAACTACAATAATACT

GCCGATCACACCAACACTACTAGCAATA????????????????????????????????

????????????????????????????????????????????????????????????

????????????????????????????????????????????????????????????

????????????????????????????????????????????????????????????

????????????????????????????????????????????????????????????

????????????????????????????????????????????????????????????

????????????????????????????????????????????????????????????

????????????????????????????????????????????????????????????

????????????????????????????????????????????????????????????

????????????????????????????????????????????????????????????

????????????????????????????????????????????????????????????

????????????????????????????????????????????????????????????

????????????????????????????????????????????????????????????

????????????????????????????????????????????????????????????

????????????????????????????????????????????????????????????

????????????????????????????????????????????????????????????

????????????????????????????????????????????????????????????

????????????????????????????????????????????????????????????

????????????????????????????????????????????????????????????

?????????????????????????????????????

>LC017835

????????????????????????????????????????????????????????????

????????????????????????????????????????????????????????????

????????????????????????????????????????????????????????????

????????????????????????????????????????????????????????????

????????????????????????????????????????????????????????????

????????????????????????????????????????????????????????????

????????????????????????????????????????????????????????????

?????????????ATGAACCCATATGCACTATCAATCCTGTTGTCAAGCCTAGCAGTAGG

AACAATTACAACTCTCTCAAGCTCCCATTGATTTCTGGCATGAGTAGGACTAGAAATTAA

TACACTAGCAATAATCCCGCTAATAACAAAAATACACCACCCACGATCAACAGAATCAGC

CACAAAATACTTCCTAACGCAAGCCACCGCATCAGCCTTAATTTTATTTTCTACAACCAT

AAACGCATGGACTACAGGAGAATGAACAATTATCACCATAAATGACAACTCATCAACATT

TATATTAACAGCTGCCCTAGCTATTAAACTTGGACTCGCCCCTTTTCATCTGTGACTACC

AGACGTCATACAAGGATTAAACCTAACAACATGCCTAATCCTTGCAACATGACAAAAATT

AGCCCCAATATCCTTAATAATTATAACCAACCACCAATTAAACACAAACCTTCTAATTAC

AATAGCCGTATTATCAACAGTAATTGGGGGGTGGGGGGGACTTAACCAAACACAAATCCG

AAAAATAATAGCATATTCATCTATCGCCCACCTTGGATGAATAACACTAGTCCTCTCTTT

CTCCCCAAACTTAACAACCCTTAACCTAATAATTTACCTAATATTAACATCATCAATGTT

CTTAATAATAACAACCCTAAACTCAACAAATATTAACAAACTTTCAACATCTTGACTGAA

GACCCCACTACTAACATCATCAATAATAATTACACTAATGGCCCTAGGAGGCCTACCCCC

CACAACAGGATTCCTACCAAAATGACTCATCCTACAAGAAATAACCAAACAACACCTAGG

AGCCGTATCCGCACTAATAGCCATATCTGCACTACTAAGCCTATTCTTCTACTTACGACT

ATCATATGCAATCTCGCTAACTACCGCACCAAACACATCAAACTCCAACATAACCTGACG

AATAACCCACAACCAAATAAAAATCCTACCAACAATAATTACACTAACTACAATAATACT

GCCGATCACACCAACACTACTAGCAATA????????????????????????????????

????????????????????????????????????????????????????????????

????????????????????????????????????????????????????????????

????????????????????????????????????????????????????????????

????????????????????????????????????????????????????????????

????????????????????????????????????????????????????????????

????????????????????????????????????????????????????????????

????????????????????????????????????????????????????????????

????????????????????????????????????????????????????????????

????????????????????????????????????????????????????????????

????????????????????????????????????????????????????????????

????????????????????????????????????????????????????????????

????????????????????????????????????????????????????????????

????????????????????????????????????????????????????????????

????????????????????????????????????????????????????????????

????????????????????????????????????????????????????????????

????????????????????????????????????????????????????????????

????????????????????????????????????????????????????????????

????????????????????????????????????????????????????????????

?????????????????????????????????????

>AB830728

????????????????????????????????????????????????????????????

????????????????????????????????????????????????????????????

????????????????????????????????????????????????????????????

????????????????????????????????????????????????????????????

????????????????????????????????????????????????????????????

????????????????????????????????????????????????????????????

????????????????????????????????????????????????????????????

?????????????ATGAACCCGTATGCACTATCAATCCTGTTATCGAGCCTAGCAGTAGG

AACAATTACAACTCTCTCAAGCTCCCATTGATTTCTGGCATGAGTAGGACTAGAAATTAA

CACACTAGCAATAATCCCGCTAATAACAAAAACACACCACCCACGATCAACAGAATCAGC

CACAAAATACTTCCTAACGCAAGCCGCCGCATCAGCCTTAATCTTATTTTCTACAATCAT

AAACGCATGGGCCACAGGAGAATGAACAATTAACACCATAAATGACAACTCATCAACATT

TATATTAACAGTTGCCCTAGCTATTAAACTTGGAGTCGCCCCTTTTCATCTATGACTACC

AGATGTTATACAAGGGTTAAACCTAACAACATGCCTAATCCTTGCAACATGACAAAAATT

AGCCCCAATATCCTTAATAATTATAATCAACCACCAATTAAACACAAACCTCCTAATTAC

AATAGCCGTATTATCCGCAGTAATTGGAGGATGAGGTGGACTTAACCAAACACAAATCCG

AAAAATAATAGCATATTCATCTATCGCCCACCTCGGGTGAATGACACTAGTCCTATCCTT

CTCCCCAAATTTAACAACCCTTAACCTAATAATTTACCTAATACTAACATCATCAATATT

CTTAATAATAACAACCCTAAACTCAACAAATATTAACAAACTTTCAACATCTTGACTGAA

GGCCCCACTACTAACATCATCAATAATAATTACACTCATAGCCCTAGGGGGCCTACCACC

CACATCAGGGTTCCTACCAAAATGGCTCATCCTACAAGAAATAACCAAACAACACCTAGG

AGCTGTGTCCACACTAATAGCTATATCTGCACTACTAAGCCTATTCTTCTACCTACGACT

ATCATATGCAATCTCACTAACTACCGCACCAAACACATCAAACTCTAACATAACCTGACG

AATAACCCACACCCAAATACAAACCCCACCAATAATAATTACACTGACTACAATAATACT

ACCAATCACGCCAACACTACTAGCAATA????????????????????????????????

????????????????????????????????????????????????????????????

????????????????????????????????????????????????????????????

????????????????????????????????????????????????????????????

????????????????????????????????????????????????????????????

????????????????????????????????????????????????????????????

????????????????????????????????????????????????????????????

????????????????????????????????????????????????????????????

????????????????????????????????????????????????????????????

????????????????????????????????????????????????????????????

????????????????????????????????????????????????????????????

????????????????????????????????????????????????????????????

????????????????????????????????????????????????????????????

????????????????????????????????????????????????????????????

????????????????????????????????????????????????????????????

????????????????????????????????????????????????????????????

????????????????????????????????????????????????????????????

????????????????????????????????????????????????????????????

????????????????????????????????????????????????????????????

?????????????????????????????????????

>AB830729

????????????????????????????????????????????????????????????

????????????????????????????????????????????????????????????

????????????????????????????????????????????????????????????

????????????????????????????????????????????????????????????

????????????????????????????????????????????????????????????

????????????????????????????????????????????????????????????

????????????????????????????????????????????????????????????

?????????????ATGAACCCATATGCACTATCAATCCTGTTGTCGGGCCTAGCAGTAGG

AACAATTACAACTCTCTCAAGCTCCCACTGATTTCTGGCATGAGTAGGACTAGAAATTAA

CACACTAGCAATAATCCCGCTAATAACAAAAACACACCACCCGCGGTCAACAGAATCAGC

CACAAAATACTTCCTAACGCAGGCCGCTGCATCAGCCCTAGTCTTATTTTCTACAGTCAT

AAACGCAGGGGCTACAGGAGAGTGAACAATTATCACCATAAATGACAGCCCATCAACATT

TATATTAACAATTGCCCTCTGTATTAAACTCGGGGTCGCCCCTTTTCATCTATGACTACC

AGATGTTATACAAGGGTTAAACCTAACAACATGCCTAATCCTTGCAACATGACAAAAATT

AGCCCCAATGTCCTTAATAATTATAACCAACCACCAATTAAACACAAACCTGCTAATTAC

AATAGCCGCACTATCAGCAGTAATTGGGGGGTGGGGGGGACTTAACCAAACACAAATCCG

AAAAATAATAACATACTCATCTATCGCCCACCTCGGGTGAATAACACTAGTCCTATCTTT

CTCCCCAAATTTAACAACACTTAACCTAATAATCTACCTAATACTAACATCATCAATATT

CTTAATAATAACAACCCTAAACTCAACAAATATTAATAAACTTTCAACATCTTGACTAAA

AACCCCACTACTAGCAGCATCAATAATAATCACGCTCATGGCCCTAGGAGGCCTACCCCC

CACATCAGGGTTCCTACCAAAATGACTTATCCTACAAGAAATAACCAAACAGCACCTAGG

GGCCGTATCCACACTAATAGCTATGTCTGCACTACTAAGCCTTTTCTTCTACCTACGGCT

ATCATATGCGATCTCACTAACCACCGCGCCAAACACATCAAACTCTAACACAACCTGACG

AACAGCCCACCCCCAAATACAAACCACACCAACAATAATTACACTAACCACAATAATATT

ACCAATCACACCAACACTACTAGCAGTA????????????????????????????????

????????????????????????????????????????????????????????????

????????????????????????????????????????????????????????????

????????????????????????????????????????????????????????????

????????????????????????????????????????????????????????????

????????????????????????????????????????????????????????????

????????????????????????????????????????????????????????????

????????????????????????????????????????????????????????????

????????????????????????????????????????????????????????????

????????????????????????????????????????????????????????????

????????????????????????????????????????????????????????????

????????????????????????????????????????????????????????????

????????????????????????????????????????????????????????????

????????????????????????????????????????????????????????????

????????????????????????????????????????????????????????????

????????????????????????????????????????????????????????????

????????????????????????????????????????????????????????????

????????????????????????????????????????????????????????????

????????????????????????????????????????????????????????????

?????????????????????????????????????

>AB830730

????????????????????????????????????????????????????????????

????????????????????????????????????????????????????????????

????????????????????????????????????????????????????????????

????????????????????????????????????????????????????????????

????????????????????????????????????????????????????????????

????????????????????????????????????????????????????????????

????????????????????????????????????????????????????????????

?????????????ATGAACCCATATGCACTATCAATCCTGTTGTCGGGCCTAGCAGTAGG

AACAATTACAACTCTCTCAAGCTCCCACTGATTTCTGGCATGAGTAGGACTAGAAATTAA

CACACTAGCAATAATCCCGCTAATAACAAAAACACACCACCCGCGGTCAACAGAATCAGC

CACAAAATACTTCCTAACGCAGGCCGCTGCATCAGCCCTAGTCTTATTTTCTACAGTCAT

AAACGCAGGGGCTACAGGAGAGTGAACAATTATCACCATAAATGACAGCCCATCAACATT

TATATTAACAATTGCCCTCTGTATTAAACTCGGGGTCGCCCCTTTTCATCTATGACTACC

AGATGTTATACAAGGGTTAAACCTAACAACATGCCTAATCCTTGCAACATGACAAAAATT

AGCCCCAATGTCCTTAATAATTATAACCAACCACCAATTAAACACAAACCTGCTAATTAC

AATAGCCGCACTATCAGCAGTAATTGGGGGGTGGGGGGGACTTAACCAAACACAAATCCG

AAAAATAATAGCATACTCATCTATCGCCCACCTCGGGTGAATAACACTAGTCCTATCTTT

CTCCCCAAATTTAACAACACTTAACCTAATAATCTACCTAATACTAACATCATCAATATT

CTTAATAATAACAACCCTAAACTCAACAAATATTAATAAACTTTCAACATCTTGACTAAA

AACCCCACTACTAGCAGCATCAATAATAATCACGCTCATGGCCCTAGGAGGCCTACCCCC

CACATCAGGGTTCCTACCAAAATGACTTATCCTACAAGAAATAACCAAACAGCACCTAGG

GGCCGTATCCACACTAATAGCTATGTCTGCACTACTAAGCCTTTTCTTCTACCTACGGCT

ATCATATGCGATCTCACTAACCACCGCGCCAAACACATCAAACTCTAACACAACCTGACG

AACAGCCCACCCCCAAATACAAACCACACCAACAATAATTACACTAACCACAATAATATT

ACCAATCACACCAACACTACTAGCAGTA????????????????????????????????

????????????????????????????????????????????????????????????

????????????????????????????????????????????????????????????

????????????????????????????????????????????????????????????

????????????????????????????????????????????????????????????

????????????????????????????????????????????????????????????

????????????????????????????????????????????????????????????

????????????????????????????????????????????????????????????

????????????????????????????????????????????????????????????

????????????????????????????????????????????????????????????

????????????????????????????????????????????????????????????

????????????????????????????????????????????????????????????

????????????????????????????????????????????????????????????

????????????????????????????????????????????????????????????

????????????????????????????????????????????????????????????

????????????????????????????????????????????????????????????

????????????????????????????????????????????????????????????

????????????????????????????????????????????????????????????

????????????????????????????????????????????????????????????

?????????????????????????????????????

>AB830733

????????????????????????????????????????????????????????????

????????????????????????????????????????????????????????????

????????????????????????????????????????????????????????????

????????????????????????????????????????????????????????????

????????????????????????????????????????????????????????????

????????????????????????????????????????????????????????????

????????????????????????????????????????????????????????????

?????????????ATGAACCCATATGCACTATCAATCCTGTTGTCGGGCCTAGCAGTAGG

AACAATTACAACTCTCTCAAGCTCCCACTGATTTCTGGCATGAGTAGGACTAGAAATTAA

CACACTAGCAATAATCCCGCTAATAACAAAAACACACCACCCGCGGTCAACAGAATCAGC

CACAAAATACTTCCTAACGCAGGCCGCTGCATCAGCCCTAGTCTTATTTTCTACAGTCAT

AAACGCAGGGGCTACAGGAGAGTGAACAATTATCACCATAAATGACAGCCCATCAACATT

TATATTAACAATCGCCCTCTGTATTAAACTCGGGGTCGCCCCTTTTCATCTATGACTACC

AGATGTTATACAAGGGTTAAACCTAACAACATGCCTAATCCTTGCAACATGACAAAAATT

AGCCCCAATGTCCTTAATAATTATAACCAACCACCAATTAAACACAAACCTGCTAATTAC

AATAGCCGCACTATCAGCAGTAATTGGGGGGTGGGGGGGGCTTAACCAAACACAAATCCG

AAAAATAATAGCATACTCATCTATCGCCCACCTCGGGTGAATAACACTAGTCCTATCTTT

CTCCCCAAATTTAACAGCACTTAACCTAATAATCTACCTAATACTAACATCATCAATATT

CTTAATAATAACAACCCTAAACTCAACAAATATTAATAAACTCTCAACATCTTGACTAAA

AACCCCACTACTAGCAGCATCAATAATAATCACGCTCATGGCCCTAGGAGGCCTACCCCC

CACATCAGGGTTCCTACCAAAATGACTTATCCTACAAGAAATAACCAAACAGCACCTAGG

GGCCGTATCCACACTAATAGCTATATCTGCACTACTAAGCCTTTTCTTCTACCTACGGCT

ATCATATGCGATCTCACTAACCACCGCGCCAAACACATCAAACTCTAACACAACCTGACG

AACAGCCCACCCCCAAATACAAACCACACCAACAATAATTACACTAACCACAATAATATT

ACCAATCACACCAACACTACTAGCAGTA????????????????????????????????

????????????????????????????????????????????????????????????

????????????????????????????????????????????????????????????

????????????????????????????????????????????????????????????

????????????????????????????????????????????????????????????

????????????????????????????????????????????????????????????

????????????????????????????????????????????????????????????

????????????????????????????????????????????????????????????

????????????????????????????????????????????????????????????

????????????????????????????????????????????????????????????

????????????????????????????????????????????????????????????

????????????????????????????????????????????????????????????

????????????????????????????????????????????????????????????

????????????????????????????????????????????????????????????

????????????????????????????????????????????????????????????

????????????????????????????????????????????????????????????

????????????????????????????????????????????????????????????

????????????????????????????????????????????????????????????

????????????????????????????????????????????????????????????

?????????????????????????????????????

>AB830735

????????????????????????????????????????????????????????????

????????????????????????????????????????????????????????????

????????????????????????????????????????????????????????????

????????????????????????????????????????????????????????????

????????????????????????????????????????????????????????????

????????????????????????????????????????????????????????????

????????????????????????????????????????????????????????????

?????????????GTGAACCCATATGCACTATCTATTCTATTATCAAGCCTAGCAGCAGG

AACAATTACAACTCTTTCAAGCTCCCACTGATTCCTTGCATGAGTTGGCCTAGAAATTAA

CACACTAGCAATCATCCCACTAATGACAAAAATACACCACCCACGAGCAACAGAATCAGC

CACAAAATATTTCCTAACGCAAGCCACCGCATCAGCCCTGATCTTATTCTCTACAATTAC

AAACGCATGAGCCACAGGAGAATGAACAATTATTGCCATAAATGATAGCATATCAACGCT

TATCCTAACAATTGCCCTAGCCATTAAACTCGGAATCGCCCCATTCCACCTATGGTTACC

AGACGTCATACAAGGATTAAACCTAACGACATGCTTAATCCTTACAACGTGACAAAAACT

AGCTCCAATATCATTAATAATTATAACCGGCCACCAATTAAACACAAACCTACTAATTAC

AATAGCTATATTATCAACAATTCTTGGGGGGTGGGGAGGCCTTAACCAAACACACATACG

AAAAATAATAGCATATTCATCTATCGCCCACCTCGGGTGAATAACACTAATCCTATCTTT

CTCCCCAAACTTAACAACCCTAAACCTAATAATTTACCTAATACTAACCTCATCGATATT

CTTAATAATAATAACCCTAACCGCAACAAATATAAACAAACTTTCAACATCCTGACTAAA

AACACCCCTATTAGCATCATCAATAATAATTACACTTATAGCCTTAGGAGGGCTGCCCCC

TACCTCAGGCTTCTTACCAAAGTGACTTATCCTACAAGAAATAACTAAACAACACCTAGG

AACCGTATCCACACTAATAGCCATATCTGCGCTACTAAGCCTGTTCTTCTACCTGCGACT

ATCGTATGTAATCTCATTAACTACTGCACCAAACACCTCAAACTCCAATATGGCCTGACG

AACACCCCACAACCAAATACAAACACTCCCCGCAATAATTACACTAACCACAATAATACT

ACCAATCACACCAACACTATTAGCAATA????????????????????????????????

????????????????????????????????????????????????????????????

????????????????????????????????????????????????????????????

????????????????????????????????????????????????????????????

????????????????????????????????????????????????????????????

????????????????????????????????????????????????????????????

????????????????????????????????????????????????????????????

????????????????????????????????????????????????????????????

????????????????????????????????????????????????????????????

????????????????????????????????????????????????????????????

????????????????????????????????????????????????????????????

????????????????????????????????????????????????????????????

????????????????????????????????????????????????????????????

????????????????????????????????????????????????????????????

????????????????????????????????????????????????????????????

????????????????????????????????????????????????????????????

????????????????????????????????????????????????????????????

????????????????????????????????????????????????????????????

????????????????????????????????????????????????????????????

?????????????????????????????????????

>AB830736

????????????????????????????????????????????????????????????

????????????????????????????????????????????????????????????

????????????????????????????????????????????????????????????

????????????????????????????????????????????????????????????

????????????????????????????????????????????????????????????

????????????????????????????????????????????????????????????

????????????????????????????????????????????????????????????

?????????????GTGAACCCATATGCACTATCTATTCTATTATCAAGCTTAGCAGCAGG

AACAATTACAACTCTTTCAAGCTCCCACTGATTCCTTGCATGAGTTGGCCTAGAAATTAA

CACACTAGCAATCATCCCACTAATGACAAAAATACACCACCCACGAGCAACAGAATCAGC

CACAAAATATTTCCTAACGCAAGCCACCGCATCAGCCCTGATCTTATTCTCTACAATTAC

AAACGCATGAGCCACAGGAGAATGAACAATTATTGCCATAAATGATAGCATATCAACGCT

TATCCTAACAATTGCCCTAGCCATTAAACTCGGAATCGCCCCATTTCACCTATGGTTACC

AGACGTCATACAAGGATTAAACCTAACGACATGCTTAATCCTTGCAACGTGACAAAAACT

AGCTCCAATATCATTAATAATTATAACCAGCCACCAATTAAACACAAACCTACTAATTAC

AATAGCTATATTATCAACAATTCTTGGGGGGTGGGGAGGCCTTAACCAAACACACATACG

AAAAATAATAGCATATTCATCTATCGCCCACCTCGGGTGAATAACACTAATCCTATCTTT

CTCCCCAAACTTAACAACCCTAAACCTAATAATTTACCTAATATTAACCTCATCGATATT

CTTAATAATAATAACCCTAACCGCAACAAATATAAACAAACTTTCAACATCCTGACTAAA

AACACCCCTATTAGCACCATCAATAATAATTACACTTATAGCCTTAGGAGGGCTGCCCCC

TACCTCAGGCTTCTTACCAAAGTGACTTATCCTACAAGAAATAACTAAACAACACCTAGG

AACCGTATCCACACTTATAGCCATATCTGCGCTACTAAGCCTGTTCTTCTACCTACGACT

ATCGTATGTAATCTCATTAACTACTGCACCAAACACCTCAAACTCCAATATGGCCTGACG

AACCCCCCACAACCAAATACAAACACTCCCCGCAATAATTACACTAACCACAATAATACT

ACCAATCACACCAACACTATTAGCAATA????????????????????????????????

????????????????????????????????????????????????????????????

????????????????????????????????????????????????????????????

????????????????????????????????????????????????????????????

????????????????????????????????????????????????????????????

????????????????????????????????????????????????????????????

????????????????????????????????????????????????????????????

????????????????????????????????????????????????????????????

????????????????????????????????????????????????????????????

????????????????????????????????????????????????????????????

????????????????????????????????????????????????????????????

????????????????????????????????????????????????????????????

????????????????????????????????????????????????????????????

????????????????????????????????????????????????????????????

????????????????????????????????????????????????????????????

????????????????????????????????????????????????????????????

????????????????????????????????????????????????????????????

????????????????????????????????????????????????????????????

????????????????????????????????????????????????????????????

?????????????????????????????????????

>AB922822

????????????????????????????????????????????????????????????

????????????????????????????????????????????????????????????

????????????????????????????????????????????????????????????

????????????????????????????????????????????????????????????

????????????????????????????????????????????????????????????

????????????????????????????????????????????????????????????

????????????????????????????????????????????????????????????

?????????????ATGAATCCATATGCACTATCAATCCTGTTGTCGAGCCTAGCAGTAGG

AACACTCACAACTCTCTCAAGCTCCCATTGATTCCTGGCATGAGTAGGACTAGAAATTAA

CACACTAGCAATAACCCCCTTGATAACAAAAATACACCACCCACGATCAACAGAATCAGC

CACAAAATACTTCCTAACGCAAGCCACCGCATCAGCCTTAATCTTATTTTCTACAATCAC

AAACGCATGAGCCACTGGAGAATGAACAATTACCACCATAAATGACAACACATCAACATT

TATACTAACAATTGCCCTAGCTATTAAACTTGGAATCGCCCCTTTTCATCTATGACTACC

AGACGTTATACAAGGACTAAATCTAACAACATGCCTAATCCTTGCGACATGGCAAAAATT

AGCCCCAATGTCCTTAATAATTATAACCAACCACCAATTAAACACAAACATGCTAATTAC

AATAGCCGTGTTATCAACAGTAATTGGCGGATGAGGGGGCCTTAACCAAACACAAATTCG

AAAAATAATAGCATACTCATCTATCGCCCACCTCGGATGAATAACACTAGTCCTATCTTT

CTCCCCAAATTTAACAACCCTTAACCTAATAATCTACCTAATACTAACATCATCAATATT

CTTAATAATAAAAACCCTAAACTCAACAAATATTAACAAACTTTCAATATCTTGACTGAA

AGCCCCCCTATTAGCATCATCAATAATAATTACACTCATAGCCCTAGGAGGTCTACCCCC

AACATCAGGGTTCCTACCAAAATGACTTATCCTACAAGAAATAACCAAACAACACCTAGG

CACCGTATCCACACTAATAGCTATATCTGCGCTACTCAGTTTATTCTTCTACCTACGACT

ATCATATGCAATCTCACTAACCACCACACCAAACACCTCAAACTCCAACATAACCTGACG

AATAACACACAGCCAAATACAAATCCTACCAACAATAATTACACTAACTACAATAATACT

ACCGATCACACCAACACTACTAGCAACA????????????????????????????????

????????????????????????????????????????????????????????????

????????????????????????????????????????????????????????????

????????????????????????????????????????????????????????????

????????????????????????????????????????????????????????????

????????????????????????????????????????????????????????????

????????????????????????????????????????????????????????????

????????????????????????????????????????????????????????????

????????????????????????????????????????????????????????????

????????????????????????????????????????????????????????????

????????????????????????????????????????????????????????????

????????????????????????????????????????????????????????????

????????????????????????????????????????????????????????????

????????????????????????????????????????????????????????????

????????????????????????????????????????????????????????????

????????????????????????????????????????????????????????????

????????????????????????????????????????????????????????????

????????????????????????????????????????????????????????????

????????????????????????????????????????????????????????????

?????????????????????????????????????

>AB922823

????????????????????????????????????????????????????????????

????????????????????????????????????????????????????????????

????????????????????????????????????????????????????????????

????????????????????????????????????????????????????????????

????????????????????????????????????????????????????????????

????????????????????????????????????????????????????????????

????????????????????????????????????????????????????????????

?????????????ATGAATCCATATGCACTATCAATCCTGTTGTCGAGCCTAGCAGTAGG

AACACTCACAACTCTCTCAAGCTCCCATTGATTCCTGGCATGAGTAGGACTAGAAATTAA

CACACTAGCAATAACCCCCTTGATAACAAAAATACACCACCCACGATCAACAGAATCAGC

CACAAAATACTTCCTAACGCAAGCCACCGCATCAGCCTTAATCTTATTTTCTACAATCAC

AAACGCATGAGCCACTGGAGAATGAACAATTACCACCATAAATGACAACACATCAACATT

TATACTAACAATTGCCCTAGCTATTAAACTTGGAATCGCCCCTTTTCATCTATGACTACC

AGACGTTATACAAGGACTAAATCTAACAACATGCCTAATCCTTGCGACATGGCAAAAATT

AGCCCCAATGTCCTTAATAATTATAACCAACCACCAATTAAACACAAACATGCTAATTAC

AATAGCCGTGTTATCAACAGTAATTGGCGGATGAGGGGGCCTTAACCAAACACAAATTCG

AAAAATAATAGCATACTCATCTATCGCCCACCTCGGATGAATAACACTAGTCCTATCTTT

CTCCCCAAATTTAACAACCCTTAACCTAATAATCTACCTAATACTAACATCATCAATATT

CTTAATAATAAAAACCCTAAACTCAACAAATATTAACAAACTTTCAATATCTTGACTGAA

AGCCCCCCTATTAGCATCATCAATAATAATTACACTCATAGCCCTAGGAGGTCTACCCCC

AACATCAGGGTTCCTACCAAAATGACTTATCCTACAAGAAATAACCAAACAACACCTAGG

CACCGTATCCACACTAATAGCTATATCTGCGCTACTCAGTTTATTCTTCTACCTACGACT

ATCATATGCAATCTCACTAACCACCACACCAAACACCTCAAACTCCAACATAACCTGACG

AATAACACACAGCCAAATACAAATCCTACCAACAATAATTACACTAACTACAATAATACT

ACCGATCACACCAACACTACTAGCAATA????????????????????????????????

????????????????????????????????????????????????????????????

????????????????????????????????????????????????????????????

????????????????????????????????????????????????????????????

????????????????????????????????????????????????????????????

????????????????????????????????????????????????????????????

????????????????????????????????????????????????????????????

????????????????????????????????????????????????????????????

????????????????????????????????????????????????????????????

????????????????????????????????????????????????????????????

????????????????????????????????????????????????????????????

????????????????????????????????????????????????????????????

????????????????????????????????????????????????????????????

????????????????????????????????????????????????????????????

????????????????????????????????????????????????????????????

????????????????????????????????????????????????????????????

????????????????????????????????????????????????????????????

????????????????????????????????????????????????????????????

????????????????????????????????????????????????????????????

?????????????????????????????????????

>HM462061

????????????????????????????????????????????????????????????

????????????????????????????????????????????????????????????

????????????????????????????????????????????????????????????

????????????????????????????????????????????????????????????

????????????????????????????????????????????????????????????

????????????????????????????????????????????????????????????

????????????????????????????????????????????????????????????

?????????????ATGAGCCCATATGCACTATCAATTCTATTGTCGAGCCTAGCAGTAGG

AACAATCACAACCCTTTCAAGCTCCCATTGATTCCTCGCATGAATGGGCCTAGAAATTAA

CACACTAGCAATCATCCCACTAATAACAAAAATACACCACCCACGAGCAACAGAATCAGC

TACAAAATACTTCCTAACGCAAACCACCGCATCAGCCTTAATCTTATTTTCTACAATCAC

AAACGCATGAGCCACAGGAGAATGAACAATTATTACCATAAATGACAACACATCAACATT

TATCTTAACAATTGCCCTGGCCATCAAACTGGGAATCGCCCCATTTCACCTCTGATTACC

AGACATCATACAAGGATTAACCCTAACAACATGTCTAATCATTACAACATGACAAAAATT

AGCCCCAATATCCTTAATAATTATAGCCAGCCATCAATTAAACACAAACCTACTGATTAC

AATGGCCATATTATCAACAGTGCTCGGGGGGTGGGGAGGCCTTAACCAAACACAAATGCG

AAAAATAATAGCATATTCATCTATCGCCCACCTCGGATGAATAACACTAATCCTATCCTT

CTCCCCCAACTTAACAACATTTAACCTAATAATTTACCTAATACTAACCTCATCAATATT

CTTAATAATAATAACAATAACCTCAACAAACATAAATAAACTTTCAACATCTTGACTGAA

AACCCCTCTACTAGCATCATCAATGATAATTACACTTATAGCCCTAGGAGGCCTACCCCC

AACCTCAGGCTTCTTGCCAAAATGGATAATCCTGCAAGAAATAACCAAACAACACCTGGG

AGCTGTATCCACACTAATAGCTATATCTGCCCTACTAAGCCTATTCTTCTACCTACGACT

ATCATATGCAGTCTCATTAACCACTGCACCAAACACCTCAAACTCCAACATAGCCTGACG

AATAACCCACAGCCAAATACAAATCCACCCTACAATAATTACACTAACCACAATAATACT

ACCAATCACACCAACACTACTAGCAATA????????????????????????????????

????????????????????????????????????????????????????????????

????????????????????????????????????????????????????????????

????????????????????????????????????????????????????????????

????????????????????????????????????????????????????????????

????????????????????????????????????????????????????????????

????????????????????????????????????????????????????????????

????????????????????????????????????????????????????????????

????????????????????????????????????????????????????????????

????????????????????????????????????????????????????????????

????????????????????????????????????????????????????????????

????????????????????????????????????????????????????????????

????????????????????????????????????????????????????????????

????????????????????????????????????????????????????????????

????????????????????????????????????????????????????????????

????????????????????????????????????????????????????????????

????????????????????????????????????????????????????????????

????????????????????????????????????????????????????????????

????????????????????????????????????????????????????????????

?????????????????????????????????????

>HM462062

????????????????????????????????????????????????????????????

????????????????????????????????????????????????????????????

????????????????????????????????????????????????????????????

????????????????????????????????????????????????????????????

????????????????????????????????????????????????????????????

????????????????????????????????????????????????????????????

????????????????????????????????????????????????????????????

?????????????ATGAGCCCATATGCACTATCAATTCTATTGTCGAGCCTAGCAGTAGG

AACAATCACAACCCTTTCAAGCTCCCATTGATTCCTCGCATGAATGGGCCTAGAAATTAA

CACACTAGCAATCATCCCACTAATAACAAAAATACACCACCCACGAGCAACAGAATCAGC

TACAAAATACTTCCTAACGCAAACCACCGCATCAGCCTTAATCTTATTTTCTACAATCAC

AAACGCATGAGCCACAGGAGAATGAACAATTATTACCATAAATGACAACACATCAACATT

TATCTTAACAATTGCCCTGGCCATCAAACTGGGAATCGCCCCATTTCACCTCTGATTACC

AGACATCATACAAGGATTAACCCTAACAACATGTCTAATCATTACAACATGACAAAAATT

AGCCCCAATATCCTTAATAATTATAGCCAGCCATCAATTAAACACAAACCTACTGATTAC

AATGGCCATATTATCAACAGTGCTCGGGGGGTGGGGAGGCCTTAACCAAACACAAATGCG

AAAAATAATAGCATATTCATCTATCGCCCACCTCGGATGAATAACACTAATCCTATCCTT

CTCCCCCAACTTAACAACATTTAACCTAATAATTTACCTAATACTAACCTCATCAATATT

CTTAATAATAATAACAATAACCTCAACAAACATAAATAAACTTTCAACATCTTGACTGAA

AACCCCTCTACTAGCATCATCAATGATAATTACACTTATAGCCCTAGGAGGCCTACCCCC

AACCTCAGGCTTCTTGCCAAAATGGATAATCCTGCAAGAAATAACCAAACAACACCTGGG

AGCTGTATCCACACTAATAGCTATATCTGCCCTACTAAGCCTATTCTTCTACCTACGACT

ATCATATGCAGTCTCATTAACCACTGCACCAAACACCTCAAACTCCAACATAGCCTGACG

AATAACCCACAGCCAAATACAAATCCACCCTACAATAATTACACTAACCACAATAATACT

ACCAATCACACCAACACTACTAGCAATA????????????????????????????????

????????????????????????????????????????????????????????????

????????????????????????????????????????????????????????????

????????????????????????????????????????????????????????????

????????????????????????????????????????????????????????????

????????????????????????????????????????????????????????????

????????????????????????????????????????????????????????????

????????????????????????????????????????????????????????????

????????????????????????????????????????????????????????????

????????????????????????????????????????????????????????????

????????????????????????????????????????????????????????????

????????????????????????????????????????????????????????????

????????????????????????????????????????????????????????????

????????????????????????????????????????????????????????????

????????????????????????????????????????????????????????????

????????????????????????????????????????????????????????????

????????????????????????????????????????????????????????????

????????????????????????????????????????????????????????????

????????????????????????????????????????????????????????????

?????????????????????????????????????

>LC017832

????????????????????????????????????????????????????????????

????????????????????????????????????????????????????????????

????????????????????????????????????????????????????????????

????????????????????????????????????????????????????????????

????????????????????????????????????????????????????????????

????????????????????????????????????????????????????????????

????????????????????????????????????????????????????????????

?????????????ATGAACCCGTATGCACTATCAATCCTGTTGTCGAGCCTAGCAGTAGG

AACAATTACAACTCTCTCAAGCTCCCATTGATTTCTGGCATGAGTAGGACTAGAAATTAA

CACACTAGCAATAACCCCGCTAATAACAAAAACACACCACCCACGATCAACAGAATCAGC

CACAAAATACTTCCTAACGCAAGCCGCCGCATCAGCCTTAATCTTATTTTCTACAATCAT

AAACGCATGGACCACAGGAGAATGAACAATTATCACCATAAATGACAACTCATCAACATT

TATATTAACAGTTGCCCTAGCTATTAAACTTGGAGTCGCCCCTTTTCATCTATGACTACC

AGATGTCATACAAGGGTTAAACCTAACAACATGCCTAATCCTTGCAACATGACAAAAATT

AGCCCCAATATCCTTAATAATTATAATCAACCACCAATTAAACACAAACCTCCTAATTAC

AATAGCCGTATTATCCGCAGTAATTGGAGGATGAGGTGGACTTAACCAAACACAAATCCG

AAAAATAATAGCATATTCATCTATCGCCCACCTCGGGTGAATGACACTAGTCCTATCCTT

CTCCCCAAATTTAACAACCCTTAACCTAATAATTTACCTAATACTAACATCATCAATGTT

CTTAATAATAACAACCCTAAACTCAACAAATATTAACAAACTTTCAACATCTTGACTGAA

GGCCCCACTACTAGCATCATCAATAATAATTACACTCATAGCCCTAGGGGGCCTACCCCC

CACATCAGGGTTCCTACCAAAATGGCTCATCCTACAAGAAATAACCAAACAACACCTAGG

AGCTGTATCCACACTAATAGCTATATCTGCACTACTAAGCCTATTCTTCTACCTACGACT

ATCATATGCAATCTCACTAACTACCGCACCAAACACATCAAACTCTAACATAACCTGACG

AATAACCCACACCCAAATACAAACCCTACCAATAATAATTACACTGACTACAATAATACT

ACCAATCACGCCAACACTACTAGCAATA????????????????????????????????

????????????????????????????????????????????????????????????

????????????????????????????????????????????????????????????

????????????????????????????????????????????????????????????

????????????????????????????????????????????????????????????

????????????????????????????????????????????????????????????

????????????????????????????????????????????????????????????

????????????????????????????????????????????????????????????

????????????????????????????????????????????????????????????

????????????????????????????????????????????????????????????

????????????????????????????????????????????????????????????

????????????????????????????????????????????????????????????

????????????????????????????????????????????????????????????

????????????????????????????????????????????????????????????

????????????????????????????????????????????????????????????

????????????????????????????????????????????????????????????

????????????????????????????????????????????????????????????

????????????????????????????????????????????????????????????

????????????????????????????????????????????????????????????

?????????????????????????????????????

>LC017833

????????????????????????????????????????????????????????????

????????????????????????????????????????????????????????????

????????????????????????????????????????????????????????????

????????????????????????????????????????????????????????????

????????????????????????????????????????????????????????????

????????????????????????????????????????????????????????????

????????????????????????????????????????????????????????????

?????????????ATGAACCCGTATGCACTATCAATCCTGTTGTCGAGCCTAGCAGTAGG

AACAATTACAACTCTCTCAAGCTCCCATTGATTTCTGGCATGAGTAGGACTAGAAATCAA

CACACTAGCAATAACCCCGCTAATAACAAAAACACACCACCCCCGATCAACAGAATCAGC

CACAAAATACTTCCTAACGCAAGCCGCCGCATCAGCCTTAATCTTATTTTCTACAATCAT

AAACGCATGGGCCACAGGAGAATGAACAATTATCACCATAAATGACAACGCATCAACATT

TATATTAACAGTTGCCCTAGCTATTAAACTTGGAGTCGCCCCTTTTCATCTATGACTACC

AGATGTCATACAAGGGTTAAACCTAACAACATGCCTAATCCTTGCAACATGACAAAAATT

AGCCCCAATATCCTTAATAATTATAATCAACCACCAATTAAACACAAACCTCCTAATTAC

AATAGCCGTATTATCCGCAGTAATTGGAGGATGAGGTGGACTTAACCAAACACAAATCCG

AAAAATAATAGCATATTCATCTATCGCCCACCTCGGGTGAATGACACTAGTCCTATCCTT

CTCCCCAAATTTAACAACCCTTAACCTAATAATTTACCTAATGCTAACATCATCAATGTT

CTTAATAATAACAACCCTAAACTCAACAAATATTAACAAACTTTCAACATCTTGACTGAA

GGCCCCACTACTAGCATCATCAATAATAATTACACTCATAGCCCTAGGGGGCCTACCCCC

CACATCAGGGTTTCTACCAAAATGGCTCATCCTACAAGAAATAACCAAACAACACCTAGG

AGCTGTGTCCACACTAATAGCTATATCTGCACTACTAAGCCTATTCTTCTACCTACGACT

ATCATATGCAATCTCACTAACTACCGCACCAAACACATCAAACTCTAACATAACCTGACG

AATAACCCACACCCAAACACAAGCCCTACCAATAATAATTACACTGACTACAATAATACT

ACCAATCACACCAACACTACTAGCAATA????????????????????????????????

????????????????????????????????????????????????????????????

????????????????????????????????????????????????????????????

????????????????????????????????????????????????????????????

????????????????????????????????????????????????????????????

????????????????????????????????????????????????????????????

????????????????????????????????????????????????????????????

????????????????????????????????????????????????????????????

????????????????????????????????????????????????????????????

????????????????????????????????????????????????????????????

????????????????????????????????????????????????????????????

????????????????????????????????????????????????????????????

????????????????????????????????????????????????????????????

????????????????????????????????????????????????????????????

????????????????????????????????????????????????????????????

????????????????????????????????????????????????????????????

????????????????????????????????????????????????????????????

????????????????????????????????????????????????????????????

????????????????????????????????????????????????????????????

?????????????????????????????????????

>ZHJY1

CAACGGCCGCGGTATCATGACCGTGCAAAGGTAGCGTAATCACTTGTCTTTTAAATAAAG

ACCTGTATGAAAGGCAAAACGAAAGTTCAACTGTCTCTCTAATCCAATCAATGAAATTTA

TCTTTTCGTGCAGAAGCGAAAATAAGTATATAAGACGAGAAGACCCTATGGAGCTTTAAA

CACACTATAGTATAAGTTTTAGGTTGGGGCGACCACGAAGAAAAAAAATCCTTCGAGATA

AGAACAACACTTCAAAAATTAAAACATTTTGATCCACTAAGTGACCAACGAACCAAGTTA

CCCTAGGGATAACAGCGCAATCCTTTCTAAGAGTCCATATCGACGAATGGGTTTACGACC

TCGATGTTGGATCAGGACACCCAAATGGTGCAGAAGCTATTAAGGTTCGTTTGTTCAACG

ATTAAAGTCCTACATGAGCCCATATGCACTCTCAATTCTACTGTCAAGCCTGGCAATAGG

AACAATCACAACTCTATCAGGCTCCCACTGATTCCTAGCATGAGTGGGCCTAGAAATTAA

TACACTGGCAATTATTCCACTTATAACAAAAATACACCACCCACGATCAACAGAATCAGC

CACAAAATACTTCTTAACGCAAGCTACTGCATCAGCCTTAATCTTATTCTCCGCAATCAC

AAACGCATGAACCACAGGAGAATGAACAATTATTACCATAAGCGACAACACATCAACATC

TATCCTAACAATTGCTTTAGCCATAAAACTAGGAATCGCCCCATTTCACTTATGATTACC

AGATGTTATACAAGGACTAAACTTAACAACATGCCTAATTCTTACAACATGACAAAAATT

AGCCCCAATATCCTTAATAATTATATCCAGCCACCAACTAAACACAAATCTACTAATTAC

AATAGCCATACTCTCAACAGTAATTGGAGGCTGAGGCGGCCTTAACCAGACACAAATACG

AAAAGTAATAGCATATTCATCCATCGCCCACCTCGGATGAATGACACTAATCCTATCTTT

CTCCCCCACTCTAACAACCCTAAACTTAATTATTTACCTAATATTAACCTCATCAATATT

CTTAATAATAATAACCCTAAACTCAACGAACATCAACAAACTTTCACTATCTTGACTAAA

AACACCACTAATAGCATCATCCATAATAATAACCCTCATAGCCCTTGGAGGGCTGCCCCC

AACCTTGGGATTCTTACCAAAATGACTAATTCTACAAGAAATAACCAAACAACACCTAGA

AACTATATCCGCCCTAATTGCAATATCCGCCCTACTAAGCCTATTCTTCTACCTCCGATT

ATCATATGCAATTTCACTAACTACTGCACCAAACATCTCAAATTCTAATATTACATGACG

AATAAACTGCAACCAAACACAAATTCTCCCAACAATAATTACGCTAACCACAATAATACT

ACCAATAACACCAGCACTCCTCCCAATAGGACAAGGCAGCTTGGCGTACCCAGGTCTGAG

GACCCACGGGACTCTGGAGAGCATAGGTGGGCCTAAAGGCAATTCAAGAGGAGGGTTGCC

CTCACTAGCAGACACTTTTGAACAAGTCATTGAGGAGATGCTTGAAGATGAGCAGAGCAT

GAGGCCCAGTGAGGAGAACAAGGATGCGGACATGTTTACATCTCGCGTAATGCTGAGCAG

TCAAGTGCCTTTGGAGCCTCCTCTTCTCTTTCTGCTGGAGGAGTACAAAAACTACTTGGA

TGCTGCAAATATGTCCATGAGGGTCCGACGTCACTCTGACCCCGCACGCCGCGGGGAGCT

GAGTGTGTGTGACAGTATTAGTGAGTGGGTGACGGCATCTGACAAAAAGACTGCCGTGGA

CATGTCGGGGCAGACGGTAACTGTTCTAGAAAAAGTTCCAGTACCCAAAGGCCAACTGAA

GCAATACTTCTACGAGACCAAATGCAACCCTATGGGTTACATGAAGGAGGGCTGCAGAGG

CATTGACAAGAGGTACTGGAACTCCCAGTGCCGAACTACTCAGTCGTATGTGCGCGCGCT

CACCATGGATAACAAGAAGAAAGTGGGCTGGCGGTTTATCAGAATAGACACTTCATGTGT

ATGTACATTGACCATTGTTTGCTTGGGTTGCAGACCGAAGGCTGTTGTTCTACAAATATG

TCTACAAGCGATATAGAGCTGGGAAACAAAGGGGTATGATTATAGAAACCGAAGGTGACA

GGCCTTCTTCAAAAGCTGACATTGAAATGGATGGAAAGATGGTCAACTCTCATACTGAAA

ATTTCATTGATGGCTCTCTAGTTCTGGAAGTCGATGAGAAAGATCAGGATGATGAAGAAG

CCAGGCGCGATATGGCCAGAATCCTAAAGGAGCTAAAGCAGAAACATCCTGACAAGGAAA

TAGAGCAACTGATTGAGTTAGCGAACTACCAGGTCTTGAGTCAACAGCAAAAAAGTCGAG

CATTCTATCGTATTCAAGCTACTCGCTTGATGACTGGGGCCGGAAATATTCTAAAGAGAC

ATGCAGCAGACCAGGCAAGGAAAGCTGTGAGCATGCAGGAAGTTAACTCTGAAGTGATCG

AAAATGAGCCCGTCAGTAAGATCTATTTCGAGCAATC

>ZHJY2

CAACGGCCGCGGTATCATGACCGTGCAAAGGTAGCGTAATCACTTGTCTTTTAAATAAAG

ACCTGTATGAAAGGCAAAACGAAAGTTCAACTGTCTCTCTAATCCAATCAATGAAATTTA

TCTTTTCGTGCAGAAGCGAAAATAAGTATATAAGACGAGAAGACCCTATGGAGCTTTAAA

CACACTATAGTATAAGTTTTAGGTTGGGGCGACCACGAAGAAAAAAAATCCTTCGAGATA

AGAACAACACTTCAAAAATTAAAACATTTTGATCCACTAAGTGACCAACGAACCAAGTTA

CCCTAGGGATAACAGCGCAATCCTTTCTAAGAGTCCATATCGACGAATGGGTTTACGACC

TCGATGTTGGATCAGGACACCCAAATGGTGCAGAAGCTATTAAGGTTCGTTTGTTCAACG

ATTAAAGTCCTACATGAGCCCATATGCACTCTCAATTCTACTGTCAAGCCTGGCAATAGG

AACAATCACAACTCTATCAGGCTCCCACTGATTCCTAGCATGAGTGGGCCTAGAAATTAA

TACACTGGCAATTATTCCACTTATAACAAAAATACACCACCCACGATCAACAGAATCAGC

CACAAAATACTTCTTAACGCAAGCTACTGCATCAGCCTTAATCTTATTCTCCGCAATCAC

AAACGCATGAACCACAGGAGAATGAACAATTATTACCATAAGCGACAACACATCAACATC

TATCCTAACAATTGCTTTAGCCATAAAACTAGGAATCGCCCCATTTCACTTATGATTACC

AGATGTTATACAAGGACTAAACTTAACAACATGCCTAATTCTTACAACATGACAAAAATT

AGCCCCAATATCCTTAATAATTATATCCAGCCACCAACTAAACACAAATCTACTAATTAC

AATAGCCATACTCTCAACAGTAATTGGAGGCTGAGGCGGCCTTAACCAGACACAAATACG

AAAAGTAATAGCATATTCATCCATCGCCCACCTCGGATGAATGACACTAATCCTATCTTT

CTCCCCCACTCTAACAACCCTAAACTTAATTATTTACCTAATATTAACCTCATCAATATT

CTTAATAATAATAACCCTAAACTCAACGAACATCAACAAACTTTCACTATCTTGACTAAA

AACACCACTAATAGCATCATCCATAATAATAACCCTCATAGCCCTTGGAGGGCTGCCCCC

AACCTTGGGATTCTTACCAAAATGACTAATTCTACAAGAAATAACCAAACAACACCTAGA

AACTATATCCGCCCTAATTGCAATATCCGCCCTACTAAGCCTATTCTTCTACCTCCGATT

ATCATATGCAATTTCACTAACTACTGCACCAAACATCTCAAATTCTAATATTACATGACG

AATAAACTGCAACCAAACACAAATTCTCCCAACAATAATTACGCTAACCACAATAATACT

ACCAATAACACCAGCACTCCTCCCAATAGGACAAGGCAGCTTGGCGTACCCAGGTCTGAG

GACCCACGGGACTCTGGAGAGCATAGGTGGGCCTAAAGGCAATTCAAGAGGAGGGTTGCC

CTCACTAGCAGACACTTTTGAACAAGTCATTGAGGAGATGCTTGAAGATGAGCAGAGCAT

GAGGCCCAGTGAGGAGAACAAGGATGCGGACATGTTTACATCTCGCGTAATGCTGAGCAG

TCAAGTGCCTTTGGAGCCTCCTCTTCTCTTTCTGCTGGAGGAGTACAAAAACTACTTGGA

TGCTGCAAATATGTCCATGAGGGTCCGACGTCACTCTGACCCCGCACGCCGCGGGGAGCT

GAGTGTGTGTGACAGTATTAGTGAGTGGGTGACGGCATCTGACAAAAAGACTGCCGTGGA

CATGTCGGGGCAGACGGTAACTGTTCTAGAAAAAGTTCCAGTACCCAAAGGCCAACTGAA

GCAATACTTCTACGAGACCAAATGCAACCCTATGGGTTACATGAAGGAGGGCTGCAGAGG

CATTGACAAGAGGTACTGGAACTCCCAGTGCCGAACTACTCAGTCGTATGTGCGCGCGCT

CACCATGGATAACAAGAAGAAAGTGGGCTGGCGGTTTATCAGAATAGACACTTCATGTGT

ATGTACATTGACCATTGTTTGCTTGGGTTGCAGACCGAAGGCTGTTGTTCTACAAATATG

TCTACAAGCGATATAGAGCTGGGAAACAAAGGGGTATGATTATAGAAACCGAAGGTGACA

GGCCTTCTTCAAAAGCTGACATTGAAATGGATGGAAAGATGGTCAACTCTCATACTGAAA

ATTTCATTGATGGCTCTCTAGTTCTGGAAGTCGATGAGAAAGATCAGGATGATGAAGAAG

CCAGGCGCGATATGGCCAGAATCCTAAAGGAGCTAAAGCAGAAACATCCTGACAAGGAAA

TAGAGCAACTGATTGAGTTAGCGAACTACCAGGTCTTGAGTCAACAGCAAAAAAGTCGAG

CATTCTATCGTATTCAAGCTACTCGCTTGATGACTGGGGCCGGAAATATTCTAAAGAGAC

ATGCAGCAGACCAGGCAAGGAAAGCTGTGAGCATGCAGGAAGTTAACTCTGAAGTGATCG

AAAATGAGCCCGTCAGTAAGATCTATTTCGAGCAATC

>ET880314

CAACGGCCGCGGTATCATGACCGTGCAAAGGTAGCGTAATCACTTGTCTTTTAAATAAAG

ACCTGTATGAAAGGCAAAACGAAAGTTCAACTGTCTCTTTAATCCAATCAATGAAATTTA

TCTTTTCGTGCAGAAGCGAAAATAAATATATAAGACGAGAAGACCCTATGGAGCTTTAAA

CACACTATAGTATAAGTTTTAGGTTGGGGCGACCACGGAGAAAAACAACCCTCCGAGATA

AGAACAACACTTCAAAAATTAAAACATTTTGACCCACTAAGTGATCAACGAACCAAGTTA

CCCTAGGGATAACAGCGCAATCCTTTCTAAAAGTCCATATCGACGAATGGGTTTACGACC

TCGATGTTGGATCAGGACACCCAAATGGTGCAGCAGCTATTAAGGTTCGTTTGTTCAACG

ATTAAAGTCCTACATGAACCCATATGCACTCTCAATTTTATCATCAAGCCTAGCAGTAGG

AACAATCATAACTCTCTCAAGCTCCCACTGATTCCTGGCATGACTTGGCCTAGAAATTAA

TACCCTAGCAATTATCCCACTTATGACAAAAATACACCACCCACGAGCAACAGAATCAGC

CACAAAATACTTCCTAATGCAAGCCATCGCATCAGCCTTAATCTTATTCTCTTCAACCAC

AAACGCATGAGCCACAGGAGAATGAACAATTACCACTATAAGCGACAATACATCAACATC

TCTCCTAACAATTGCTCTAGCCATCAAACTAGGAATCGCCCCATTTCACCTATGATTACC

AGATGTTATACAAGGATTAAACTTAATAACATGCCTAATCCTTGCAACATGACAAAAATT

AGCCCCAATATCCCTAATAATTATAACCAGCCATCAACTAAACACAAACCTGCTAATTGT

AATAGCTGTACTATCAACAATAGTTGGAGGATGAGGTGGCCTTAACCAAACACAAATACG

AAAAGTAATAGCATATTCATCTATTGCCCACCTCGGATGAATAACCCTAGTCCTCTCTTT

CTCCCCAAACTTAGCTACTTTAAACTTAACAATTTACCTTATATTAACCTCATCAATATT

CTTAATAATAATGACCCTAAACTCAACGAACATTAATAAACTTTCAGTATCTTGACTAAA

AACACCCATAATGGCATCATCCATAATAATAACCCTCATAGCCCTAGGAGGACTACCCCC

AACCGCAGGATTCTTACCAAAATGACTAATTTTACAAGAGATAACTAAACAACACCTGGA

GCCCGCATCTGCCCTAATTGCAATATCTGCGCTACTAAGCCTATTCTTCTATCTACGGCT

ATCATATGCAATCTTATTAACTACTGCCCCAAACACCTCAAACTCTAACATCACCTGACG

AATACACTGTAACCAAACACAAATTTCCCAAACAACAATTACACTAACCGTAATAATACT

ACCAATTACCCCAGCACTACTAATAATA????????????????????????????????

????????????????????????????????????????????????????????????

????????????????????????????????????????????????????????????

????????????????????????????????????????????????????????????

????????????????????????????????????????????????????????????

????????????????????????????????????????????????????????????

????????????????????????????????????????????????????????????

????????????????????????????????????????????????????????????

????????????????????????????????????????????????????????????

????????????????????????????????????????????????????????????

????????????????????????????????????????????????????????????

????????????????????????????????????????????????????????????

????????????????????????????????????????????????????????????

????????????????????????????????????????????????????????????

????????????????????????????????????????????????????????????

????????????????????????????????????????????????????????????

????????????????????????????????????????????????????????????

????????????????????????????????????????????????????????????

????????????????????????????????????????????????????????????

?????????????????????????????????????

>ET880329

TAACGGCCGCGGTATTATGACCGTGCAAAGGTAGCGTAATCACTTGTCTTTTAATTAGAG

ACCTGTATGAAAGGCAAAACGAAGGTTCAACTGTCTCTTTAGTCCAATCGATTAAATTTA

TCTCTCCGTGCAGAAGCGGAGATAAATATATAAGACGAGAAGACCCTATGGAGCTTTAAA

CACACTATAATACAAGTTTTGGGTTGGGGCGACCACGGAAGAAAACCATCCTCCGAGATA

AGAACAACACTTCAAAAATTAAAATATTTTGATCCACTAAGTGATCAACGAACCAAGTTA

CCCTAGGGATAACAGCGCAATCCTTTCTAAGAGTCCATATCGACGAACGGGTTTACGACC

TCGATGTTGGATCAGGACACCCAAATGGTGCAGCCGCTATTAAGGTTCGTTTGTTCAACG

ATTAAAGTCCTACATGAGCCCATACGCACTATCAGTACTATTATCAAGCCTAGCAGTCGG

AACGACTGCTACACTATCAAGCTCTCACTGATTCTTAGCATGAATTGGACTAGAGATTAA

TACACTCGCTATCATTCCCCTCATAACTAAAATACACCACCCACGAGCAACAGAATCAGC

AACAAAATACTTCTTAACACAAGCTGCCGCCTCAGCCCTAATTTTATTTTCCACAATTCT

GAATGCATGAACTACTGGGGAATGAACCATTACTGGCATGAATAGCAATACATCACTAAT

AATCTTAACAATTGCCCTGGCAATTAAACTCGGAATTGCACCATTTCACCTCTGACTACC

AGACGTTACACAAGGACTAAACTTAATAACATGCCTTATCTTGACAACATGACAAAAACT

AGCCCCAATATCACTATTAATTTTAACAAGCCACCAACTAAACACTAACACCCTTATTGT

GATAGGTATCCTTTCAGCCATTATTGGCGGATGAGGAGGACTTAACCAAACGCAAATACG

AAAAATAATGGCACACTCTTCCATCGCTCACCTTGGATGAATAACATTAGTCTTATCATT

TTCTCCTGCCCTCACAATACTAAACCTAATAATTTACTTAATACTGACCTCCTCAATATT

CCTAATAATAATAACCTTAAACTCAACAAACATCAATAAACTATCAACCTCATGACTAAA

AACACCCTTACTAGCTTCCTCAATAATAATCATCCTTATGGCCCTAGGAGGCCTTCCACC

AACTTCAGGGTTCTTACCAAAATGATTAATTCTTCAGGAAATAACTAAACAACACTTAAT

TACAATATCAACCCTAATAGCCATATCTGCCCTCCTAAGCTTATTCTTCTACCTACGCCT

ATCATATGCAATTTCCTTAACCACTGCACCCAATATTTCAAACTCCAACATTTCCTGACG

AACAACCTACAACCAGCCAATCATTTCTCCCACCACAATTACGCTAACTATATTAATAAT

GCCAATTACACCGACACTAATAGCAATA????????????????????????????????

????????????????????????????????????????????????????????????

????????????????????????????????????????????????????????????

????????????????????????????????????????????????????????????

????????????????????????????????????????????????????????????

????????????????????????????????????????????????????????????

????????????????????????????????????????????????????????????

????????????????????????????????????????????????????????????

????????????????????????????????????????????????????????????

????????????????????????????????????????????????????????????

????????????????????????????????????????????????????????????

????????????????????????????????????????????????????????????

????????????????????????????????????????????????????????????

????????????????????????????????????????????????????????????

????????????????????????????????????????????????????????????

????????????????????????????????????????????????????????????

????????????????????????????????????????????????????????????

????????????????????????????????????????????????????????????

????????????????????????????????????????????????????????????

?????????????????????????????????????

>ET880330

TAACGGCCGCGGTATCATGACCGTGCAAAGGTAGCGTAATCACTTGTCTTTTAAATAGAG

ACCTGTATGAATGGCAAAACGAAAGTTCAACTGTCTCTTTAGTCCAATCAATTAAATTTA

TCTCTCCGTGCAGAAGCGGAGATAAACATATAAGACGAGAAGACCCTATGGAGCTTTAAA

CACACTGTAATACAAGTTTTGGGTTGGGGCGACCACGGAAGAAAATCATCCTCCGAGATA

AGAACAACATTTCAAAAATTAAAACATTTTGATCCACTAAGTGATCAACGAACCAAGTTA

CCCTAGGGATAACAGCGCAATCCTTTCTAAGAGTTCATATCGACGAACGGGTTTACGACC

TCGATGTTGGATCAGGACACCCAAATGGTGCAGCCGCTGTTAAGGTTCGTTTGTTCAACG

ATTAAAGTCCTACATGAGCCCATACGCATTATCAATACTACTGTCGAGTCTAGCAGTTGG

AACAACTACTACACTATCAAGCTCACACTGATTCCTAGCATGAATTGGACTTGAAATTAA

CACACTCGCTATTATCCCCTTAATAACTAAAATACACCACCCACGAGCAACAGAATCAGC

AACAAAGTACTTCTTAACACAGGCCGCTGCTTCGGCCCTAATCTTATTCTCCACAATTCT

GAACGCATGAACTACAGGAGAATGAACCATTATTAATATGAATGACAGTACATCATTAAC

AATCTTAACAGTCGCCCTAGCAATTAAACTTGGAATTGCACCATTCCATCTTTGACTCCC

AGATGTTTTACAAGGATTAAGTCTAATAACATGCCTTATCTTAACAACATGACAAAAACT

AGCCCCAATATCACTACTAATTTTAACAAGCCACCAATTAAACACCAACCTTCTTATTAT

AATAGCCATCCTTTCAACGATCATCGGCGGATGGGGGGGGCTTAACCAAACGCAAGTACG

GAAAATAATAGCATACTCCTCCATCGCCCACTTAGGGTGAATAACGCTAGTTTTATCATT

TTCCCCCGCTCTCACAATACTAAACCTAACCATCTACTTGATACTTACCTCCTTAATATT

CCTAATAATAATAACCTTTAACTCAACAAACATTAATAAACTCTCAACCTCATGATTAAA

AACACCTTTACTGGCTTCCTCAATAATAATCGTCCTTATAGCCCTAGGGGGCCTCCCCCC

AACTTCAGGATTTCTACCAAAATGACTTATTCTTCAGGAAATGACTAAACAACACCTAAT

TACAATATCTGCACTAATGGCCATATCCGCACTCCTAAGCTTATTCTTTTACCTACGCCT

ATCCTATGCAATCTCTCTAACCACTGCACCCAATATTTCAAACTCTAACATTACCTGACG

AACAACCCACAACCAGTCAATCATTACTCCAACCATAATTTCATTAACTACATTAATAAT

ACCAATTACACCAGCACTAATAGCAATA????????????????????????????????

????????????????????????????????????????????????????????????

????????????????????????????????????????????????????????????

????????????????????????????????????????????????????????????

????????????????????????????????????????????????????????????

????????????????????????????????????????????????????????????

????????????????????????????????????????????????????????????

????????????????????????????????????????????????????????????

????????????????????????????????????????????????????????????

????????????????????????????????????????????????????????????

????????????????????????????????????????????????????????????

????????????????????????????????????????????????????????????

????????????????????????????????????????????????????????????

????????????????????????????????????????????????????????????

????????????????????????????????????????????????????????????

????????????????????????????????????????????????????????????

????????????????????????????????????????????????????????????

????????????????????????????????????????????????????????????

????????????????????????????????????????????????????????????

?????????????????????????????????????

>ET880334

CAACGGCCGCGGTATCATGACCGTGCAAAGGTAGCGTAATCACTTGTCTTTTAAATAAGG

ACCCGTATGAAAGGCAAAACGAAAGTTCAACTGTCTCTTTAATCCAATCAATGAAATTAA

TCTCTCCGTGCAGAAGCGGAAATAACAATATAAGACGAGAAGACCCTATGGAGCTTCAAA

CACAAAATAATTTACGTTTTCGGTTGGGGCGACCACGGAAGAAGCCAATCCTCCGAGATA

AGAACTACACTTCAAAAACTAAAACATTTTGACCCACTAAGTGACCAACGAACCAAGTTA

CCCTAGGGATAACAGCGCAATCCTTTCTAAGAGTCCATATCGACGAATGGGTTTACGACC

TCGATGTTGGATCAGGACACCCAGATGGTGCAGCCGCTATCAAGGTTCGTTTGTTCAACG

ATTAAAGTCCTACATGAGCCCCTATGCATTATCAATATTATTATCAAGCCTAGCAATCGG

AACAATTACCACGCTCTCTAGCTCTCACTGATTTTTAGCATGAATGGGGCTAGAAATTAA

TACACTAGCCATTATCCCCCTAATAACCAAAATACACCACCCACGAGCAACAGAATCGGC

AACAAAGTACTTCCTAATACAAGCCACGGCATCCGCCCTAATTTTATTTTCCACAATCAT

AAATGCTTGGGCCACAGGAGAATGAACAATCACAAGCATAAGCAACAACGTATCAACAAC

ACTCTTAACAATCGCCCTAGCAATAAAACTAGGAGTCGCACCATTTCACCTATGACTCCC

AGACGTACTACAAGGACTAAACCTAATAACAGGCCTAATTCTGTCAACATGACAAAAACT

GGCTCCAATGGCACTCCTTATTTTAACCAACCACCAACTCAATACAAACCTTCTAATTAT

AATGGCACTAGCATCCACAATTATTGGAGGGTGGGGGGGCCTAAATCAAACACAAATACG

AAAAATTATGGCATATTCATCAATTGCACACTTAGGCTGAATAATCATAATCTTATCCTT

CGCCCCAGCTCTGACCATCTTAAATCTTATAATCTACCTTATACTTACCTCCACCATATT

TATAATGATAATAACTATATACTCAACAAACATTAACAAACTCTCAACCTCATGACTAAA

ATCCCCAACACTTGCAGCATCCACAATAATTGCCCTGATGGCCTTAGGGGGCCTCCCCCC

CACCTCGGGCTTCCTACCAAAATGACTAATTCTACAAGAAATAACAAAACAACACCTAGA

AGCACTATCAATCCTAATAGCCATGTCTACCCTACTAAGCCTATTTTTTTACCTCCGCCT

ATCCTATGCAATCTCATTAACCTCCCCACCAAACACCTCAAACTCAAACTCAACCTGACG

AATAATAAACAACAAACCACAAATCTTATCAACCCTAATTACACTTTCCGCAATAACATT

ACCAATCACCCCAACACTACTAACAATA????????????????????????????????

????????????????????????????????????????????????????????????

????????????????????????????????????????????????????????????

????????????????????????????????????????????????????????????

????????????????????????????????????????????????????????????

????????????????????????????????????????????????????????????

????????????????????????????????????????????????????????????

????????????????????????????????????????????????????????????

????????????????????????????????????????????????????????????

????????????????????????????????????????????????????????????

????????????????????????????????????????????????????????????

????????????????????????????????????????????????????????????

????????????????????????????????????????????????????????????

????????????????????????????????????????????????????????????

????????????????????????????????????????????????????????????

????????????????????????????????????????????????????????????

????????????????????????????????????????????????????????????

????????????????????????????????????????????????????????????

????????????????????????????????????????????????????????????

?????????????????????????????????????

>ET880333

CAACGGCCGCGGTATCATGACCGTGCAAAGGTAGCGTAATCACTTGTCTTTTAAATAAAG

ACCCGTATGAAAGGCAAAACGAAAGCTCAACTGTCTCTTTAGTCTAATCAATGAAATTAA

TCTCTCCGTGCAGAAGCGGAGATGATAATATAAGACGAGAAGACCCTATGGAGCTTCAAA

TACAAAATAATTTGAATTTTCGGTTGGGGCGACCACGGAAGAAACCCATCCTCCGAGATA

AGAACCACACTTCAAAAACTAAAACATTTTGACCCACTAAGTGACCAACGAACCAAGTTA

CCCTAGGGATAACAGCGCAATCCTTTCTAAGAGCCCATATCGACGAATGGGTTTACGACC

TCGATGTTGGATCAGGACACCCAGATGGTGCAGCCGCTATCAAGGTTCGTTTGTTCAACG

ATTAAAGTCCTACATGAGCCCCTATGCATTATCAATATTATTATCAAGCCTAGCAATCGG

AACAATTACCACTCTCTCAAGCTCCCACTGATTTCTGGCATGGATAGGACTAGAGATTAA

TACGCTAGCCATTGTCCCGCTAATAACCAAAATACACCACCCGCGCGCAACAGAATCGGC

AACAAAATATTTCCTCATACAGGCCACAGCATCTGCCCTAGTTTTATTTTCCACAATCAT

AAATGCATGGGCCACGGGGGAGTGGACAATTACAAACATAAGCAACAACATATCAACAAC

AATCCTGACAATCGCCCTAGCAATAAAACTAGGAATTGCCCCATTCCACCTATGACTCCC

TGACGTCCTACAGGGGTTAAACCTAATGACAGGCTTAATCCTCTCAACATGACAAAAATT

GGCCCCAATAGCACTCCTTATCCTAACCAGCCATCAACTCAATACAAACCTTCTAATTAT

AATGGCACTAGCCTCCACAATCATCGGGGGGTGGGGAGGCCTAAACCAAACACAAATACG

AAAAATTATAGCATACTCGTCAATTGCACACCTAGGCTGAATAACCATAGTCTTATCCTT

TGCCCCCGCCCTAACCATATTAAACCTCATAATCTACCTTATACTCACCTCTACTATGTT

CATAATGATGATGGCCCTGTACGCAACGAACATAAATAAACTATCAACCTCATGACTAAA

ATCCCCAACACTCGCAGCATCCACAATAATTACCCTCATAGCCCTAGGTGGCCTTCCACC

AACCTCGGGCTTCCTACCAAAATGGCTGATTCTACAAGAAATAACAAAACAACACCTAGA

GACACTATCGGCCCTGATAGCCATGTCCGCCCTACTAAGCCTATTTTTTTATCTCCGCCT

ATCCTATGCAATCTCACTAACCTCCCCACCAAGTACCTCAAACTCAAACTCAACTTGACG

AACAACAAACAACAAACAACAAACCTTATCGACCTTAATCACGCTCACCGCAATATTAAT

ACCAATTACCCCAACACTACTAGCAACA????????????????????????????????

????????????????????????????????????????????????????????????

????????????????????????????????????????????????????????????

????????????????????????????????????????????????????????????

????????????????????????????????????????????????????????????

????????????????????????????????????????????????????????????

????????????????????????????????????????????????????????????

????????????????????????????????????????????????????????????

????????????????????????????????????????????????????????????

????????????????????????????????????????????????????????????

????????????????????????????????????????????????????????????

????????????????????????????????????????????????????????????

????????????????????????????????????????????????????????????

????????????????????????????????????????????????????????????

????????????????????????????????????????????????????????????

????????????????????????????????????????????????????????????

????????????????????????????????????????????????????????????

????????????????????????????????????????????????????????????

????????????????????????????????????????????????????????????

?????????????????????????????????????

>ET880323

CAACGGCCGCGGTATCATGACCGTGCAAAGGTAGCGTAATCACTTGTCTTTTAAATAAAG

ACCTGTATGAAAGGCAAAACGAAAGTTCAACTGTCTCTTTAATCCAATCAATGAAATTAA

TCTTTCCGTGCAGAAGCGGAAATAAAAATATAAGACGAGAAGACCCTATGGAGCTTTAAA

TATAAAATAATTTGAATTTTCGGTTGGGGCGACCACGGAGAAAAATAATCCTCCGAGATA

AGAATTACACTTCAAAAATTAAAACATTTTGATCCACTAAGTGACCAACGAACCAAGTTA

CCCTAGGGATAACAGCGCAATCCTTTCTAAGAGTCCATATCGACGAATGGGTTTACGACC

TCGATGTTGGATCAGGACATCCTAATGGTGCAGCCGCTATTAAGGTTCGTTTGTTCAACG

ATTAAAGTCCTACATGAATCCCTATGCATTATCCATATTATTATCAAGCCTAGCAATTGG

AACAACTATTACACTCTCAAGTTCTCATTGATTTCTTGCATGAATAGGCCTAGAAATTAA

TACACTAGCTATTATTCCACTAATAACTAAAATACACCATCCCCGGGCAACAGAATCTGC

AACAAAATACTTCTTAACACAAGCCACAGCATCCGCTTTAATCTTATTCTCCACAACCAT

AAATGCATGAACCACAGGAGAATGAACAATTATAAGTATAAATAATAATACAGCAACAAC

AATCCTAACAATTGCTCTAGCAATTAAACTAGGTATTGCACCGTTTCATCTATGACTACC

AGACGTACTACAAGGACTAAACTTAATAACAGGACTACTCCTCTCAACATGACAAAAACT

AGCCCCTATAGCACTACTTATTATAACCAACCACCAATTAAATACAAACTTACTAATTAC

AATAGCACTAGCATCAACAATTATTGGAGGATGAGGAGGCCTCAATCAAACACAAATACG

AAAAATTATAGCATATTCATCTATTGCACATCTAGGATGAATAATCTTAGTCTTATCTTT

CGCCCCAACCTTAACAATATTAAACTTAATAATCTATTTAATATTAACCTCTTCTATATT

TATAATAATAATAGCCACACATTCCACAAATATTAATAAACTCTCAACCTCATGACTAAA

AACACCTACATTAGCAGCATCCACAATAATTATCCTTATAGCCCTTGGAGGACTTCCTCC

AACCTCAGGCTTCCTACCAAAATGACTAATTTTACAAGAAATAACAAAACAACACCTAGA

GACACTATCAACTATAATAGCTATATCCGCTTTACTCAGTTTATTCTTTTATCTACGCCT

ATCTTATACCATTTCACTAACCTCCCCCCCAAATATCTCCAATTCAAACTTCACCTGACG

AATAAAAACCAATAAACTACAAATTCTCCCAACTCTTATTGTACTTTCAGCAATAATAAT

ACCTATCGCCCCAACACTATTGACAATA????????????????????????????????

????????????????????????????????????????????????????????????

????????????????????????????????????????????????????????????

????????????????????????????????????????????????????????????

????????????????????????????????????????????????????????????

????????????????????????????????????????????????????????????

????????????????????????????????????????????????????????????

????????????????????????????????????????????????????????????

????????????????????????????????????????????????????????????

????????????????????????????????????????????????????????????

????????????????????????????????????????????????????????????

????????????????????????????????????????????????????????????

????????????????????????????????????????????????????????????

????????????????????????????????????????????????????????????

????????????????????????????????????????????????????????????

????????????????????????????????????????????????????????????

????????????????????????????????????????????????????????????

????????????????????????????????????????????????????????????

????????????????????????????????????????????????????????????

?????????????????????????????????????

>ET880322

CAACGGCCGCGGTATCATGACCGTGCAAAGGTAGCGTAATCACTTGTCTTTTAAATAAAG

ACCTGTATGAAAGGCAAAACGAAAGTTCAACTGTCTCTTTAATCTAATCAATGAAATTAA

TCTTTCCGTGCAGAAGCGGAAATAAGAATATAAGACGAGAAGACCCTATGGAGCTTTAAA

TGTAAAATAATTTGAATTTTCGGTTGGGGCGACCACGGAGAAAAATAATCCTCCGAGATA

AGAATTACACTTCAAAAATTAAAACATTTTGATCCACTAAGTGACCAACGAACCAAGTTA

CCCTAGGGATAACAGCGCAATCCTTTCTAAGAGTCCATATCGACGAATGGGTTTACGACC

TCGATGTTGGATCAGGACATCCTAATGGTGCAGCCGCTATTAAGGTTCGTTTGTTCAACG

ATTAAAGGCCTACATGAACCCTTATGCACTATCCATATTATTATCAAGCCTAGCAATTGG

AACAACTATTACACTATCAAGCTCCCACTGATTCCTTGCATGAATAGGACTAGAAATTAA

TACATTAGCTATTATTCCCCTAATAACCAAAATACATCACCCCCGAGCAACAGAATCCGC

AACAAAATATTTTTTAACACAAGCCACAGCTTCTGCTTTAATCCTGTTCTCCACAACCAT

AAATGCATGAACCACAGGAGAATGAACAATTATAAATATAAATAATAATACAGCAACAAC

AATACTAACAATTGCCCTAGCAATTAAATTAGGTATTGCACCGTTTCATCTTTGATTACC

AGACGTACTACAAGGACTAAATTTAATAACAGGACTACTTCTCTCAACATGACAAAAACT

AGCCCCAATAGCACTACTTATTATAACCCACCACCAATTAAATACAAACTTATTAATTAT

GATAGCATTAACATCCACAATTATTGGGGGATGAGGAGGCCTTAATCAGACACAAATACG

AAAAATTATAGCATACTCATCTATCGCCCACCTGGGATGGATAATTTTAGTCTTATCTTT

TGCCCCCGCCCTAACAATGTTAAACCTAATAATTTATTTAATGTTGACCTCTTCTATGTT

TATAATAATAATAGCTACGCACTCTACAAATATTAATAAACTTTCTGCCTCGTGACTAAA

AACACCTACACTAGCAGCATCTACAATAATTACCCTTATAGCCCTTGGAGGACTCCCCCC

AACTTCAGGCTTTTTACCAAAATGACTGATTTTACAAGAAATAACAAAACAACATCTAGA

AGCACTATCAACCATAATAGCTATATCCGCCTTACTAAGCTTATTTTTTTATCTACGCCT

ATCTTACACTATCTCTATAACCTCCCCCCCCAATATCTCAAACTCAAACTTTACCTGACG

AGTAAAAAACAATAAACCTAAAATTCTCCCAACCTTTATTGTACTTTCAGCAATAATAAT

ACCTATCGCCCCAACACTATTAACAGTA????????????????????????????????

????????????????????????????????????????????????????????????

????????????????????????????????????????????????????????????

????????????????????????????????????????????????????????????

????????????????????????????????????????????????????????????

????????????????????????????????????????????????????????????

????????????????????????????????????????????????????????????

????????????????????????????????????????????????????????????

????????????????????????????????????????????????????????????

????????????????????????????????????????????????????????????

????????????????????????????????????????????????????????????

????????????????????????????????????????????????????????????

????????????????????????????????????????????????????????????

????????????????????????????????????????????????????????????

????????????????????????????????????????????????????????????

????????????????????????????????????????????????????????????

????????????????????????????????????????????????????????????

????????????????????????????????????????????????????????????

????????????????????????????????????????????????????????????

?????????????????????????????????????

>ET880317

TAACGGCCGCGGTATCATGACCGTGCAAAGGTAACGTAATCACTTGTCTTTTAAATAAAG

ACCTGTATGAAAGGCAAAACGAAAACTTAACTGTCTCTTTAATCCAATCAGTAAAATTAA

TCTTCCCGTGCAGAAACGGGGATAATTATATAAGACGAGAAGACCCTATGGAGCTTTAAA

TACAAGATAATCTAAGTTTTCGGTTGGGGCGACCACGGAAGAAAAATATCCTCCGAGATA

GGAATTACATTTCAAAACCTAAAACATTTTGACCCACTAAGTGATCAACGAACCAAGTTA

CCCTAGGGATAACAGCGCAATCCTTTCTAAGAGTTCCTATCGACGAATGGGTTTACGACC

TCGATGTTGGATCAGGACATCCAAATGGTGCAGCCGCTATTAAGGTTCGTTTGTTCAACG

ATTAAAGTCCTACATGAGCCCCTATGCGCTATCAATTCTAATATCAAGCCTGGCAATTGG

AACAATTACCACACTCTCAAGCTCTCATTGATTTCTAGCATGAATAGGATTAGAAATTAA

TACACTAGCCATTATTCCCTTAATAACTAAAATACACCACCCACGAGCCACAGAATCAGC

AACAAAATATTTCCTTACACAGGCCACTGCTTCCGCCTTGGTTTTATTTTCTACAATAAT

AAATGCCTGAATAACAGGAGAGTGAACAATCATAAATATAAGTAACCATTTTGCAACAAC

GGTACTAACAATTGCTTTAGCAATTAAACTAGGAATTGCCCCCTTCCACTTATGAATGCC

AGATGTATTACAAGGACTAAATTTAATAACATGCTTAATCTTATCAACATGACAAAAATT

AGCCCCAATAGCCCTATTAATCTTAACAAACCATCAACTTAACACAAACCTATTAATTAT

AATAGCCCTAATATCCACAATTATTGGGGGATGAGGAGGTCTTAACCAAGTACAAATACG

AAAAATTATAGCATACTCCTCAATCGCCCACTTAGGATGAATAACCCTCATCCTGTCCTT

CGCCCCAAGCTTAACAATGTTAAATCTAATAATTTACCTTATATTAACCTCATCAATATT

TATAATATTAATAACCTTAAACTCAACAAATGTCAATAAACTCTCAACCTCATGATTAAA

AACACCAACACTAGCAGCTTCTATAATAATTACTTTGACAGCTCTAGGAGGACTTCCGCC

TACCTCAGGGTTTTTACCAAAATGGTTAATTTTACAAGAAATAACAAAACAACACTTAAA

TCTAATATTAACCTTAATAGCCCTATCCGCCCTACTTAGTTTATTTTTTTATTTACGCTT

ATCATATGCAATCTCCTTAACTTCTGCACCAAACATCTCAAACTCAAATCTAATTTGACG

AAAAAAAAACAATCTACCGCAAACTATCTCCATCTTAACTATATTATCTGCCATAATATT

ACCAATTACACCAGCACTACTA??????????????????????????????????????

????????????????????????????????????????????????????????????

????????????????????????????????????????????????????????????

????????????????????????????????????????????????????????????

????????????????????????????????????????????????????????????

????????????????????????????????????????????????????????????

????????????????????????????????????????????????????????????

????????????????????????????????????????????????????????????

????????????????????????????????????????????????????????????

????????????????????????????????????????????????????????????

????????????????????????????????????????????????????????????

????????????????????????????????????????????????????????????

????????????????????????????????????????????????????????????

????????????????????????????????????????????????????????????

????????????????????????????????????????????????????????????

????????????????????????????????????????????????????????????

????????????????????????????????????????????????????????????

????????????????????????????????????????????????????????????

????????????????????????????????????????????????????????????

?????????????????????????????????????

>ET880335

TAACGGCCGCGGTATTATGACCGTGCAAAGGTAGCGTAATCACTTGTCTTTTAAATAAAG

ACCCGTATGAAAGGCAAAACGAGAGTTCAACTGTCTCTTTAATCTTATCAGTGAAATTTA

TTTCTCCGTGCAGAAGCGGAGATAACCATATAAGACGAGAAGACCCTATGGAGCTTTAAA

TACAGAGTGGTTTAGATTTTCGGTTGGGGCGACCGCGGAGTAAAATTAACCTCCACGACA

TGAACGACACTTCGAAAACTAAAACATTTTGACCCACTAAGTGATCAACGAACCAAGTTA

CCCTAGGGATAACAGCGCAATCCTTTCTAAGAGTCCATATCGACGAATGGGTTTACGACC

TCGATGTTGGATCAGGACACCCAAATGGTGCAGCCGCTATTAAGGTTCGTTTGTTCAACG

ATTAAAGTCCTACATGAACCCATTTGCACTATCAATACTCATATCAAGCCTAGCACTAGG

AACAACCATCACGCTCTCAAGCTCCCATTGATTCTTAGCGTGAATAGGGTTAGAAATCAA

CACCCTAGCTATTATTCCGCTAATAACCAAAATACACCACCCACGAGCCGCAGAATCAGC

CACAAAATACTTCTTAACACAAGCTACAGCCTCTGCCTTAATCTTATTTTCTACAATTAT

AGAAGCATGAACAACCGGAGAATGAACAATTATGAATATAACCAATAACGCACCAACAAC

AATATTGACAATTGCCCTAGCAATTAAACTTGGTATCGCACCATTTCACTCCTGACTCCC

AGATATCCTCCAAGGTCAAAGCATAATAACATGCCTAATTATCTCAACATGACAAAAACT

AGCCCCAATAGCCCTACTAATTATAGTAAGTCACCAACTAAACACTAACTTATTAATTAT

TATAGCACTAACCTCAACAATTATGGGGGGGTGGGGAGGACTTAATCAAACACAAACACG

AAAAATCATAGCATATTCATCCATCGCACACTTAGGATGAATTACCCTGATTCTATCCTT

TGCCCCAAACTTAGCGCTATTTAACCTATTAATCTACCTTTTACTAACTTCCTCTATATT

TATAACGTTAATAACTCTGAACACCCCAAACATTAATAAACTCTCAACCTCATGATTAAA

ATCGCCAATACTAGCCGTCTCCATAATAAGCATCCTTATAGCCCTAGGAGGACTTCCCCC

CACATCAGGATTTTTACCAAAATGACTGATCTTGCAAGAAATAACAAACCAGCACCTAGG

AGCACTATCCTCTCTAATAGCCCTGTCTGCCCTACTTAGTTTATTTTTTTATTTGCGAAT

CTCATATACAATCTCATTAACCTCCCCCCCAAATATTTCAAACTCTAACCTAACTTGACG

GAAAAAAAACACCCTACTATATGCTTTACCCCTAATAATCATAATATCAACAATAATAAT

GCCAATCATACCAGCACTTCTAATAAAT????????????????????????????????

????????????????????????????????????????????????????????????

????????????????????????????????????????????????????????????

????????????????????????????????????????????????????????????

????????????????????????????????????????????????????????????

????????????????????????????????????????????????????????????

????????????????????????????????????????????????????????????

????????????????????????????????????????????????????????????

????????????????????????????????????????????????????????????

????????????????????????????????????????????????????????????

????????????????????????????????????????????????????????????

????????????????????????????????????????????????????????????

????????????????????????????????????????????????????????????

????????????????????????????????????????????????????????????

????????????????????????????????????????????????????????????

????????????????????????????????????????????????????????????

????????????????????????????????????????????????????????????

????????????????????????????????????????????????????????????

????????????????????????????????????????????????????????????

?????????????????????????????????????

>ET880307

TAACGGCCGCGGTATCATGACCGTGCAAAGGTAGCGTAATCACTTGTCTTTTAAATAAAG

ACCCGTATGAAAGGCAAAACGAAAATTCAACTGTCTCTTTAATCTAATCAGTGAAATTAA

TCTCTCCGTGCAGAAGCGGAGATAACCATATAAGACGAGAAGACCCTATGGAGCTTTAAA

CATGAAATACCCTGAGTTTTCGGTTGGGGCGACCACGGAAGAAGAAAATCCTCCGAGATA

AGAACAACACTTCAAAGACTAAAACACTTTGATCCACTAAGTGACCAACGAACCAAGTTA

CCCTAGGGATAACAGCGCAATCCTTTCTAAGAGTCCATATCGACGAATGGGTTTACGACC

TCGATGTTGGATCAGGACACCCAAATGGTGCAGCCGCTATTAAGGTTCGTTTGTTCAACG

ATTAAAGTCCTACATGAGCCCCTATGCACTATCAATATTAATATCAAGCCTAGCAGTAGG

AACAACTATCACACTATCAAGCTCTCATTGATTTTTGGCCTGAATAGGACTAGAAATCAA

CACACTGGCCATCATTCCACTAATAACCAAAATACACCACCCACGAGCCGTAGAATCAGC

AACAAAATACTTTTTGACACAAGCCACAGCATCCGCCTTAATTTTATCTTCTACAGTTAT

AAACGCATGAATGACAGGAGAATGAACAATTACAAGCATAAACAACTACGCCCCAACAAC

AATCTTAACAGTTGCCCTAGCAATTAAACTAGGAGTTGCCCCATTTCACCTGTGACTACC

AGATGTCCTACAAGGACTAAATCTAATAACATGCTTAATCCTTTCAACATGACAAAAACT

TGCCCCAATAGCCCTACTAATTCTAACAAGCCACCAACTTAATATTAACCTATTAACCCT

AATAGCCCTAGCCTCTACAATCATTGGGGGGTGGGGGGGCCTCAATCAAACACAAATACG

TAAAATCATAGCATATTCATCTATTGCACACCTAGGATGAATAATCCTAATTGTCTCCTA

TGCCCCCAGCTTAGCACTATTAAATATACTTATCTACCTTGTAATAACCTCCTCCATGTT

CACAATATTGATAGCCCTAAACTCCACAAGCATAAACAAACTCTCAACTTCGTGACTAAA

AACACCAACGCTAGCAGCCTCCCTGATAGGCACTTTCATGGCCCTTGGGGGACTCCCCCC

CCTATTTGGATTTTTGCCAAAATGATTGATTTTACAAGAAATAACAAAACACCACCTAGG

AACATTATCAGTAATTATAGCCATATCCGCTCTACTCAGCCTATTTTTTTATCTGCGTCT

TTCATACACAATCTCAATGACCTCCCCCCCCAACATCTCAAACTCTAATCTAACCTGACG

GAAAAAAAACAACCTCTCACTTACTATCCCAACCCTAACTATACTGTCCACCATATTAAT

GCCAATCACACCAGCACTACTGCTACTA????????????????????????????????

????????????????????????????????????????????????????????????

????????????????????????????????????????????????????????????

????????????????????????????????????????????????????????????

????????????????????????????????????????????????????????????

????????????????????????????????????????????????????????????

????????????????????????????????????????????????????????????

????????????????????????????????????????????????????????????

????????????????????????????????????????????????????????????

????????????????????????????????????????????????????????????

????????????????????????????????????????????????????????????

????????????????????????????????????????????????????????????

????????????????????????????????????????????????????????????

????????????????????????????????????????????????????????????

????????????????????????????????????????????????????????????

????????????????????????????????????????????????????????????

????????????????????????????????????????????????????????????

????????????????????????????????????????????????????????????

????????????????????????????????????????????????????????????

?????????????????????????????????????

>ET880320

TAACGGCCGCGGTATAATGACCGTGCAAAGGTAGCGTAATCACTTGTCTTTTAAATAAGG

ACCTGTATGAAAGGCAAAACGAAAATTCAACTGTCTCTTTGATTCAATCAGTGAAATTAA

TCTCTCCGTGCAGAAGCGGAGATAACAATATAAGACGAGAAGACCCTGTGGAGCTTAAAA

CATAAGATAATCTAAGTTTTCGGTTGGGGCGACCACGGAAGAAAAAAATCCTCCGAGATA

AGAACAACACTTCAAAGACTAATATATTTTGATCCACTAAGTGACCAACGAACCAAGTTA

CCCCAGGGATAACAGCGCAATCCTTTCTAAGAGTCCATATCGACGAATGGGTTTACGACC

TCGATGTTGGATCAGGACACCCAAATGGTGCAGCCGCTATTAAGGTTCGTTTGTTCAACG

ATTAAAGTCCTACATGAGCCCATATGCAGTATCAATCTTAATATCAAGCCTGGCAGTAGG

AACAATTATTACAATATCAAGCTCCCACTGATTCCTAGCCTGAATAGGCCTAGAAATTAA

CACACTAGCTATCATCCCACTAATAACTAAAATACACCACCCACGAGCCACAGAATCAGC

AACAAAATACTTCTTAACGCAAGCCACAGCTTCAGCCTTAATTTTATTTTCAACAATTAT

AAATGCCTGAATAACAGGAGAATGAACTATCACAAGCATAATAAACTACCCGCCAACGAC

AATTTTAACAATTGGCCTAGCAATTAAACTAGGAATTGCCCCATTCCACTTATGATTACC

AGACGTCCTACAAGGACTAGAAATAATAACATGCTTAATCCTCTCAACCTGACAAAAACT

AGCCCCAATAACCCTGCTCATTTTAGTAAGTCACCAACTAAACACCAACCTATTAGTAAT

AATAGCCATAATATCAACAATTATCGGCGGATGAGGGGGACTAAATCAGACACAAATACG

AAAAATTATAGCATACTCATCAATTGCACACCTAGGCTGAATAACCATAATTGTTTCTTT

TGCACCCAGCCTAGCAATGTTAAACCTAGTAATTTACCTAATAATAACATCATCTATATT

TTTTATATTAATTTCTCTTAACTCAACAAACCTAAATAAACTATCCATTTCATGAATAAA

AACGCCAACACTAGCAGCCTCAATGATAGCCACTCTTATAGCCCTTGGAGGACTACCACC

CATATCAGGATTCCTACCAAAATGATTAATTTTACAAGAAATAACAAAACAATACCTAGG

AACTGTATCAACTATAATAGCCATATCCACACTACTTAGCCTATTTTTTTACCTTCGACT

CTCATACGCAATTTCAATAACCTCTCCTCCTAACATCTCAAACTCAAACCTAACCTGACG

AAAAAAAAACCACATTCCCCTAATCACACCAATTTTAATTATAATATCTACTATATTAAT

ACCAATTACACCAACCCTGTTAATAACA????????????????????????????????

????????????????????????????????????????????????????????????

????????????????????????????????????????????????????????????

????????????????????????????????????????????????????????????

????????????????????????????????????????????????????????????

????????????????????????????????????????????????????????????

????????????????????????????????????????????????????????????

????????????????????????????????????????????????????????????

????????????????????????????????????????????????????????????

????????????????????????????????????????????????????????????

????????????????????????????????????????????????????????????

????????????????????????????????????????????????????????????

????????????????????????????????????????????????????????????

????????????????????????????????????????????????????????????

????????????????????????????????????????????????????????????

????????????????????????????????????????????????????????????

????????????????????????????????????????????????????????????

????????????????????????????????????????????????????????????

????????????????????????????????????????????????????????????

?????????????????????????????????????

>ET880337

TAACGGCCGCGGTATCATGACCGTGCAAAGGTAGCGTAATCACTTGTCTTTTAAATAAAG

ACCTGTATGAAAGGCAAAACGAAAATTCAACTGTCTCTTTAATCCAATCAGTAAAATTAA

TCTCTCCGTGCAGAAGCGGAGATATTTTTATAAGACGAGAAGACCCTATGGAGCTTTAAA

CATAAAATAATTTAAGTTTTCGGTTGGGGCGACCACGGAAGAAGAAAATCCTCCGCGATA

AGAACAACACTTCAAAGACTAAAACATTTTGACCCACTAAGTGATCAACGAACCAAGTTA

CCCTAGGGATAACAGCGCAATCCTTTCTAAGAGTCCATATCGACGAATGGGTTTACGACC

TCGATGTTGGATCAGGACACCCAAATGGTGCAGCCGCTATTAAGGTTCGTTTGTTCAACG

ATTAAAGTCCTACATGAGCCCCTATGCACTATCAATGCTAATATCAAGCCTAGCTGCTGG

AACAGTCATCACACTATCGAGCTCCCACTGATTCCTAGCTTGAATAGGACTAGAAATTAA

TACACTAGCTATTATCCCGCTAATAACTAAGATACACCACCCACGAGCCACAGAATCCGC

AACAAAATACTTCTTAACACAAGCCACAGCTTCCGCCCTTCTTCTATTCGCCACCACTAT

AAACGCGTGACTGACAGGAGAATGGACTATTATAAGCATAACCAGCTATGCCCCTATGAC

AATCTTAACAATTGCCCTAGCAATTAAACTGGGGATCGCCCCCTTTCATCTTTGACTACC

AGATGTCTTGCAAGGCCTAGATATAATAACATGCTTAATCCTCTCAACATGACAAAAACT

CGCCCCAATAGCCCTTTTAATTTTAACAAACCACCAACTTAATGCCAATCTACTTATCCT

AATAGCCCTAGCATCCGCAATCATCGGGGGATGAGGGGGCCTAAATCAAACCCAAACGCG

GAAAATTATAGCATACTCATCTATTGCACACCTTGGCTGAATGACCCTAGTTGTCTCCTT

TTCCCCAAACCTGGCGCTACTAAACCTAGCAATCTATCTTATAATAACTTCATCCATATT

TATAGGGCTCATATTCCTAAACGCTACAAGCGTGAATAAACTATCAATCTCCTGACTAAA

AACACCAACACTAGCAGTATCAACAATAGCAGGTCTCATATCCCTTGGAGGCCTCCCTCC

ACTATCAGGATTTTTACCAAAATGACTAATCCTGCAAGAAATAACTAAACACCACCTAGG

AACTATCTCCGCCATAATAGCCTTATCAGCCCTTCTAAGTCTCTTTTTCTACCTCCGCTT

ATCTTATGTAATCTCAATAACCTCCCCCCCCAACATTTCAAACTCTAACTTAACCTGACG

GAAGAAAAACAACTTACCCCTAATTATCCCAATATTAATTGTATTATCTACCATACTAAT

ACCAATTATACCCATACTACTAATAAAA????????????????????????????????

????????????????????????????????????????????????????????????

????????????????????????????????????????????????????????????

????????????????????????????????????????????????????????????

????????????????????????????????????????????????????????????

????????????????????????????????????????????????????????????

????????????????????????????????????????????????????????????

????????????????????????????????????????????????????????????

????????????????????????????????????????????????????????????

????????????????????????????????????????????????????????????

????????????????????????????????????????????????????????????

????????????????????????????????????????????????????????????

????????????????????????????????????????????????????????????

????????????????????????????????????????????????????????????

????????????????????????????????????????????????????????????

????????????????????????????????????????????????????????????

????????????????????????????????????????????????????????????

????????????????????????????????????????????????????????????

????????????????????????????????????????????????????????????

?????????????????????????????????????

>ET880336

TAACGGCCGCGGTATCATGACCGTGCAAAGGTAGCGTAATCACTTGTCTTTTAAATAAAG

ACCTGTATGAAAGGCAAAACGAAAATTCAACTGTCTCTTTAATTCAATCGGTAAAATTAA

TCTCTCCGTGCAGAAGCGGAGATAGCCATATAAGACGAGAAGACCCTATGGAGCTTTAAA

CGCAAAATAATCTAAGTTTTCGGTTGGGGCGACCACGGAAGAAGTAGATCCTCCGCGATA

AGAACAACACTTCGAAGACTAAAACATTTTGATCCACTAAGTGACCAACGAACCAAGTTA

CCCTAGGGATAACAGCGCAATCCTTTCTAAGAGTCCATATCGACGAATGGGTTTACGACC

TCGATGTTGGATCAGGACACCCAAATGGTGCAACCGCTATTAAGGTTCGTTTGTTCAACG

ATTAAAGTCCTACATGAGCCCTTATGCACTGTCAATATTAATATCAAGCCTGGCTATAGG

AACGGTTATTACACTGTCTAGCTCCCACTGGTTCCTGGCTTGAATGGGACTGGAAATTAA

TACATTGGCTATTATCCCGCTTATGACTAAAATACACCACCCACGAGCCACAGAATCTGC

AACAAAATACTTCTTAACACAAGCTACAGCTTCTGCTTTACTTCTATTCTCTGCAACTAT

AAACGCATGAATAACAGGAGAATGAACTATTATGAACATAAATAGCCACACCCCTACAAC

AATCTTAACAATCGCCCTAGCAATTAAGCTAGGAGTTGCCCCATTTCACCTCTGACTACC

AGACGTCCTACAAGGGCTCGACATAATAACATGCCTAATCCTCTCAACATGGCAAAAACT

TGCCCCAATAGCCCTGCTAATTTTAACAAGCCACCAACTGAATACCAACCTATTAACCCT

AATGGCCCTCGCATCCGCAATTATTGGAGGATGAGGGGCCCTTAATCAAACCCAAACACG

GAAAATCATGGCATATTCGTCTATTGCACACCTTGGCGGGATAGTACTGGTCATCTCATT

TGCCCCAAGCCTGGCGCTGTTAAATCTAATAATCTACCTCATAATAACCTCATCTATATT

TATAATACTAATAGCCCTAAACGCCACAAATATGAATAAACTGTCAATTTCCTGACTAAA

AACACCAACACTGGCAGCCTCAATAATAGCCGGCCTCCTCTCCCTAGGAGGCCTCCCCCC

TCTCTCAGGGTTTTTACCAAAGTGATTAATCCTGCAAGAAATGACTAAACATCACCTAGG

GGCCCTCTCCACTGTGATGGCCCTATCAGCCCTACTAAGTCTTTTTTTCTACCTCCGACT

CTCGTACATGATCTCAATAACCTCCCCACCCAACATCTCAAACGCTAACTTAGCCTGACG

AACGAAAAACAACCTATCACTTATTAATCCAATTTTGATTGTGTTATCCACCATACTAAT

GCCAATCACACCCATGCTGCTAATAAGA????????????????????????????????

????????????????????????????????????????????????????????????

????????????????????????????????????????????????????????????

????????????????????????????????????????????????????????????

????????????????????????????????????????????????????????????

????????????????????????????????????????????????????????????

????????????????????????????????????????????????????????????

????????????????????????????????????????????????????????????

????????????????????????????????????????????????????????????

????????????????????????????????????????????????????????????

????????????????????????????????????????????????????????????

????????????????????????????????????????????????????????????

????????????????????????????????????????????????????????????

????????????????????????????????????????????????????????????

????????????????????????????????????????????????????????????

????????????????????????????????????????????????????????????

????????????????????????????????????????????????????????????

????????????????????????????????????????????????????????????

????????????????????????????????????????????????????????????

?????????????????????????????????????

>ET880311

TAACGGCCGCGGTATCATGACCGTGCAAAGGTAGCGTAATCACTTGTCTTTTAAATAAAG

ACCTGTATGAAAGGCAAAACGAAAGCTCAACTGTCTCTTTAATCTAATCAGTGAAATTAA

TTTCTTCGTGCAGAAGCGAAGATAACCATATAAGACGAGAAGACCCTATGGAGCTTAAAA

TACAAAATAATTCAAATTTTCGGTTGGGGCGACCACGGAAGAGAAATATCCTCCGAGATA

AGAATTACACTTCAAAAATTAAAATATTTTGATCCACTAAGTGACCAACGAACCAAGTTA

CCCTAGGGATAACAGCGCAATCCTTTCTAAGAGTTCATATCGACGAATGGGTTTACGACC

TCGATGTTGGATCAGGACACCCAAATGGTGCAGCCGCTATTAAGGTTCGTTTGTTCAACG

ATTAAAGTCCTACATGAGCCCCTACACGCTATCCATACTGATGTCAAGTCTTGCAGTAGG

TACAATTACAACACTATCAAGCTATCACTGATTCTTAGCATGAATAGGACTAGAAGTTAA

CACACTAGCTATAATTCCACTAATAACAAAAATACACCATCCACGAGCCACAGAATCCGC

AACAAAATATTTTTTAACACAAGCCACAGCCTCCGCCTTAATTCTATTCTCAACTATTTT

AAACGCATGAATAACAGGAGAGTGAACAATTATTAATATAAACCATGATACATCAATAAC

AATTTTGACAATTGCCCTGGCAATTAAACTAGGGATCGCGCCCTTTCACCTGTGATCACC

AGACGTTCTACAGGGATTAAATATAATAACATGCTTAATCCTGTCGACATGACAAAAACT

AGCCCCAATGGCCCTACTTATTTTAACAAGTCCCCATCTAAATACAAACCTATTGATTTT

TATAGCACTAACATCCATAATCATGGGAGGATGGGGAGGCCTAAATCAAACACAAATACG

AAAAATTATAGCATACTCATCCATTGCACATCTAGGGTGAATGGTTATAGTCTTACCTTT

TGCCCCAAATTTAGCCCTGCTAAACCTATCAATTTATCTTATCTTAACTTCCTCTATATT

TTTAACATTAATAGCTTTAAACTCAATAAACATGAATAAACTCTCAACCTCATGACTAAA

AACACCAACACTAGCAGCCTTATCTATAGCTGTCCTTATAGCCCTGGGGGGACTCCCACC

AACGTCAGGGTTTTTACCAAAATGGCTAATTCTGCAAGAAATAACCAAACAACACCTTAA

TGCATTATCTGTTGTAATAGCCATATCAGCCCTACTTAGTCTATTTTTTTATCTACGCCT

GTCATACACAATCTCATTAACCTCCCCCCCACATGTCTCAAACTCAAACCTCACCTGACG

AAAAAAAAATAACCTTCGATTTATC???CCCATGATAATTACATTATCCGTAATAATAAT

ACCAGTAACTCCAATACTACTA???ATA????????????????????????????????

????????????????????????????????????????????????????????????

????????????????????????????????????????????????????????????

????????????????????????????????????????????????????????????

????????????????????????????????????????????????????????????

????????????????????????????????????????????????????????????

????????????????????????????????????????????????????????????

????????????????????????????????????????????????????????????

????????????????????????????????????????????????????????????

????????????????????????????????????????????????????????????

????????????????????????????????????????????????????????????

????????????????????????????????????????????????????????????

????????????????????????????????????????????????????????????

????????????????????????????????????????????????????????????

????????????????????????????????????????????????????????????

????????????????????????????????????????????????????????????

????????????????????????????????????????????????????????????

????????????????????????????????????????????????????????????

????????????????????????????????????????????????????????????

?????????????????????????????????????

>ET880313

TAACGGCCGCGGTATCATGACCGTGCAAAGGTAGCGTAATCACTTGTCTCTTAAATAGAG

ACCTGTATGAAAGGCAAAACGAAAGCTCAACTGTCTCTTTAATTTAATCAGTGAAATTAA

TCTCTCCGTGCAGAAGCGAAGATAATTATATAAGACGAGAAGACCCTATGGAGCTTTAAA

TGTAAGATAATTTAAATTTTCGGTTGGGGCGACCACGGAAGAAAAATATCCTCCGAGATA

AGAATTACATTTCAAAAATTAAAACATTTTGATCCACCAAGTGACCAACGAACCAAGTTA

CCCTAGGGATAACAGCGCAATCTTTTCTAAGAGTTCATATCGACGAATGGGTTTACGACC

TCGATGTTGGATCAGGACACCCCAATGGTGCAGCAGCTATTAAGGTTCGTTTGTTCAACG

ATTAAAGTCCTACATGAGCCCCTATACACTATCCATATTAATATCAAGCCTTGCAGTAGG

AACAATTACCACCTTATCGAGCTATCACTGATTTTTAGCATGAATGGGGTTAGAAATTAA

TACACTAGCTATTATCCCACTAATAACCAAAATACACCACCCCCGAGCCACAGAATCCGC

AACAAAATATTTTTTAACACAAGCCACAGCATCTGCCTTAGTTTTATTCTCAACAATTAT

AAACGCATGAATAACAGGAGAATGAACAATTTTTAATATAAACCACTATACATCAACAAC

AATTTTAACAATTGCTTTGGCAATTAAATTAGGAGTTGCACCATTTCATATATGACTGCC

AGACGTTTTACAAGGATTAAACATAATAACATGCTTGATCTTGTCAACATGACAAAAACT

AGCTCCAATAGCCTTGCTTGTTTTGACAAGCCAACAGCTAAATACAAAACTACTAATTAT

AATAGCTGTAATATCCATAATTGTGGGAGGATGGGGAGGCCTAAATCAAACTCAAATGCG

AAAAATTATAGCATATTCATCCATTGCACATCTAGGATGAATAATCATAATTATACCTTT

TGCCACAAGTCTAGCGCTACTAAACCTGCTAATCTATATTATCCTAACTTCCTCTATGTT

TTTAACATTAATAACTTTGAATTCCACAAACATAAACAAACTCTCAACCTCCTGACTAAA

AACACCAACACTAGCAGCCTTCACAATAATTGTTCTTTTAGCTCTTGGAGGACTTCCACC

AACATCAGGATTCTTGCCAAAATGGTTAATCCTACAGGAGATAACCAAACAACACCTAAA

CGCACTATCAGTCGTAATAGCCATATCAGCTCTACTTAGCTTATTTTTTTACCTTCGTTT

ATCCTATACAATCTCGCTAACCTCGCCCCCCAACATCTCAAACTCAAACCTCACATGACG

AAAAAAAAATAATCTACATTCCACC???CCTATAATAATTGTATTATCCACAATACTAAT

ACCAATCACCCCAACACTACTG???ATA????????????????????????????????

????????????????????????????????????????????????????????????

????????????????????????????????????????????????????????????

????????????????????????????????????????????????????????????

????????????????????????????????????????????????????????????

????????????????????????????????????????????????????????????

????????????????????????????????????????????????????????????

????????????????????????????????????????????????????????????

????????????????????????????????????????????????????????????

????????????????????????????????????????????????????????????

????????????????????????????????????????????????????????????

????????????????????????????????????????????????????????????

????????????????????????????????????????????????????????????

????????????????????????????????????????????????????????????

????????????????????????????????????????????????????????????

????????????????????????????????????????????????????????????

????????????????????????????????????????????????????????????

????????????????????????????????????????????????????????????

????????????????????????????????????????????????????????????

?????????????????????????????????????

>ET880327

TAACGGCCGCGGTATCATGACCGTGCAAAGGTAGCGTAATCACTTGTCTTTTAAATAAAG

ACCTGTATGAAAGGCAAAACGAAAGCTCAACTGTCTCTTTAATCTAATCAGTGAAATTAA

TCTCTCCGTGCAGAAGCGAAGATAATTATATAAGACGAGAAGACCCTATGGAGCTTTAAA

TATAAAATAATTCAAATTTTCGGTTGGGGCGACCACGGAAGAAAACCATCCTCCGAGACA

AGAATTACACTTCAAAAACTAAAACATTTTGATCCACCAAGTGACCAACGAACCAAGTTA

CCCTAGGGATAACAGCGCAATCCTTTCTAAGAGTCCATATCGACGAATGGGTTTACGACC

TCGATGTTGGATCAGGACACCCAAATGGTGCAGCCGCTATTAAGGTTCGTTTGTTCAACG

ATTAAAGTCCTACATGAGCCCCTATACACTATCCGTGCTAATATCAAGTCTTGCAGTAGG

GACAATTACCACACTATCGAGTCACCACTGATTTCTAGCATGAATAGGACTAGAAATTAA

TACACTGGCTATTATCCCACTAATAACTAAAATACACCACCCCCGGGCTACAGAATCCGC

AACAAAGTACTTTCTAACACAAGCCACAGCCTCCGCCTTAATTTTATTCTCAACAATCAT

AAATGCATGAATAATAGGAGAATGAACAATCATAAACATAAACCACCACACATCAACAAC

AATCTTAACGATTGCTTTAGCAATTAAATTAGGAGTCGCACCATTTCACCTGTGATTACC

AGACGTTCTACAAGGACTAAATATGATAACATGCCTGATCCTGTCAACATGACAAAAACT

AGCCCCTATAGCCCTACTTATTTTGACAAGCCATCAACTAAACCCAAATCTACTAATTAT

AATAGCACTAACATCCATGGTTGTAGGAGGATGGGGTGGCCTAAATCAAACACAAACACG

AAAAATCATAGCATACTCATCCATTGCACACTTAGGATGAATAACTATAATTGTGCCCTT

TGCCCCAAATCTGGCCCTACTAGCCCTATTAATTTATATTATCCTAACCTCATCTATATT

TATAACATTAATAACCTTAAACTCAATAAACATAAACAAACTCTCAACCTCATGACTAAA

AACACCAACACTAGCAGCCTTTACAATAATTACCCTAATAGCCCTTGGAGGGCTCCCACC

AACATCAGGATTTTTACCAAAATGACTAATTCTACAAGAAATAACCAAACAACACCTTAA

TGCCCTCTCCACCATAATAGCCATATTAGCCCTACTCAGCCTATTCTTTTACTTACGTCT

ATCATATACAATCTCAATAACCTCACCCCCTCACATCTCAAACTCAAACCTAGTCTGACG

GAAAAAAAATAACCTACAGACTATC???CCAATAATAATTACACTATCGGCAATATTAAT

ACCAATAACTCCAACACTGCTA???ATA????????????????????????????????

????????????????????????????????????????????????????????????

????????????????????????????????????????????????????????????

????????????????????????????????????????????????????????????

????????????????????????????????????????????????????????????

????????????????????????????????????????????????????????????

????????????????????????????????????????????????????????????

????????????????????????????????????????????????????????????

????????????????????????????????????????????????????????????

????????????????????????????????????????????????????????????

????????????????????????????????????????????????????????????

????????????????????????????????????????????????????????????

????????????????????????????????????????????????????????????

????????????????????????????????????????????????????????????

????????????????????????????????????????????????????????????

????????????????????????????????????????????????????????????

????????????????????????????????????????????????????????????

????????????????????????????????????????????????????????????

????????????????????????????????????????????????????????????

?????????????????????????????????????

>ET880328

TAACGGCCGCGGTATCATGACCGTGCAAAGGTAGCGTAATCACTTGTCTTTTAAATAAAG

ACCCGTATGAAAGGCAAAACGAAAGCTCAACTGTCTCTTTAATCCAATCAGTGAAATTAA

TCTCCCCGTGCAGAAGCGGGGATAGTTATATAAGACGAGAAGACCCTATGGAGCTTTAAA

TATAAAGTAACTTAAATTTTTGGTTGGGGCGACCACGGAAGAAAACCATCCTCCGAGATA

AGAACCACACTTCAAAAACTAAAACACTTTGATCCACTAAGTGACCAACGAACCAAGTTA

CCCTAGGGATAACAGCGCAATCCTTTCTAAGAGTTCATATCGACGAATGGGTTTACGACC

TCGATGTTGGATCAGGACACCCAAATGGTGCAGCCGCTATTAAGGTTCGTTTGTTCAACG

ATTAAAGTCCTACATGAGCCCCTATACACTATCCATGTTGCTGTCAAGCCTTGCAGTAGG

AACAACTACCACACTATCAAGCAATCACTGATTTTTAGCATGAATCGGGCTAGAAATTAA

CACACTAGCCATCATCCCGCTAATAACCAAAATACACCACCCCCGGGCCACAGAGTCCGC

AACAAAATACTTTTTAACACAAGCCACAGCCTCTGCCTTAGTTTTATTTTCAACAATTAT

AAACGCATGGATGACGGGAGAATGGGCAATCATAAACATAAGCCACAACACATCGACAAC

AATTTTAACAATTGCCCTAGCAATTAAACTGGGGGTTGCCCCATTCCACCTATGATTGCC

AGATGTTATACAAGGACTAAACATAATAACGTGCTTAATCCTGTCAACATGACAGAAGCT

GGCCCCAATAGCCCTGCTTATCCTGACAAGCCATCAACTTAATACAAACCTGCTGATTAT

AATGGCACTAACCTCCATAATTGTAGGGGGATGGGGGGGCCTGAATCAAACACAAACACG

AAAAATCATAGCATACTCGTCTATTGCACACTTAGGGTGGATATTCATAATTGTCTCATT

TGCCCCAAACCTAGCACTGCTGAACCTATTGGTTTATCTCATCCTGACTTCATCTATATT

TATAACACTAATAACCTTGAACGCTACAAACATAAACAAGCTCTCGACCTCATGACTAAA

AACACCAATACTAACAGCCCTGACAATAGTGACCCTTATAGCCCTTGGAGGACTCCCGCC

AACATCCGGATTTTTACCAAAATGGCTAATCCTACAGGAAATAACCAAACAACACCTTAA

TGCACTATCTACCACAATAGCCATATTGGCCCTGCTCAGCTTATTCTTCTACCTGCGCCT

ATCATACATAGTCTCAATAACCTCCCCTCCCCACATCTCAAACTCAAACCTAACCTGACG

GAAAAAAAACAACCTACACGCCATC???CCCCTAATAATTATTTTATCCACAATAATAGT

ACCAATGGCCCCAGCGCTGCTT???ACA????????????????????????????????

????????????????????????????????????????????????????????????

????????????????????????????????????????????????????????????

????????????????????????????????????????????????????????????

????????????????????????????????????????????????????????????

????????????????????????????????????????????????????????????

????????????????????????????????????????????????????????????

????????????????????????????????????????????????????????????

????????????????????????????????????????????????????????????

????????????????????????????????????????????????????????????

????????????????????????????????????????????????????????????

????????????????????????????????????????????????????????????

????????????????????????????????????????????????????????????

????????????????????????????????????????????????????????????

????????????????????????????????????????????????????????????

????????????????????????????????????????????????????????????

????????????????????????????????????????????????????????????

????????????????????????????????????????????????????????????

????????????????????????????????????????????????????????????

?????????????????????????????????????

>ET880325

TAACGGCCGCGGTATCATGACCGTGCAAAGGTAGCGTAATCACTTGTCTTTTAAATAAAG

ACCTGTATGAAAGGCAAAACGAAAGCTCAACTGTCTCTTTAATCTAATCAGTGAAATTAA

TCCCTCCGTGCAGAAGCGGAGATAATTATATAAGACGAGAAGACCCTATGGAGCTTTAAA

TATAAAATAGTTTAAATTTTCGGTTGGGGCGACCACGGAAGAAAAACATCCTCCGAGATA

GGAATTACACTTCAAAAATTAAAACATTTTGATCCACTAAGTGACCAACGAACCAAGTTA

CCCTAGGGATAACAGCGCAATCCTTTCTAAGAGTCCCTATCGACGAGTGGGTTTACGACC

TCGATGTTGGATCAGGACACCCAAATGGTGCAGCCGCTATTAAGGTTCGTTTGTTCAACG

ATTAAAGTCCTACATGAGCCCATATACACTATCTATATTAATGTCAAGCCTTGCAGTAGG

TACAACTACCACATTATTAAGCTATCACTGATTCCTGGCATGAATAGGACTAGAGATTAA

TACACTAGCTATTATTCCGTTAATAACCAAAATACACCATCCTCGGGCCACAGAATCCGC

AACAAAATACTTTTTGACACAGGCCACAGCCTCCGCCTTAATTTTATTCTCAACAATTAT

AAACGCATGAGCCACAGGAGAGTGAACAATTATTGATATGAGCCACTACACATCCACAAC

AATTTTAACAATTGCTTTAGCTATTAAACTAGGAGTTGCACCCTTTCACCTGTGATTACC

GGATGTTTTACAAGGACTAAATATAATAACATGCCTGATCCTATCAACATGACAAAAACT

AGCCCCAATGGCCCTGCTCATCCTAACAAGTCATCAACTAAATACCAATTTACTAACTGT

AATAGCACTGATATCCATGGTTGTAGGGGGGTGAGGAGGCCTGAATCAAACACAAACACG

AAAAATCATAGCATACTCATCCATCGCACATTTAGGATGAATAACCATGATTGTATCATT

TGCCCCAAATTTAGCACTACTAAACCTATTGATCTATATTATTTTAACCTCATCTATGTT

TGCAACACTAATAACCATGAGCGCTACAAATATAAATAAGCTCTCAGCCTCCTGGCTAAA

AACACCAACACTAGCAGCCCTTACAATAGTTGTCCTCATGGCCCTTGGAGGGCTTCCACC

AACATCGGGGTTTTTGCCAAAATGACTAATTTTACAAGAAATAACCAAACAACACCTCAA

CACTTTGTCCATTACAATAGCCATATCAGCCCTACTTAGTTTATTCTTCTACCTGCGTTT

ATCCTACGCAATTTCAATAACCTCACCCCCCCACATCTCAAACTCAAACCTAAGTTGACG

TAAAAAAAACAACCTACAAATCATC???CCTATGATAATTGTATTATCTACTATAATGAT

GCCAATAACTCCGACATTGCTGGTAATA????????????????????????????????

????????????????????????????????????????????????????????????

????????????????????????????????????????????????????????????

????????????????????????????????????????????????????????????

????????????????????????????????????????????????????????????

????????????????????????????????????????????????????????????

????????????????????????????????????????????????????????????

????????????????????????????????????????????????????????????

????????????????????????????????????????????????????????????

????????????????????????????????????????????????????????????

????????????????????????????????????????????????????????????

????????????????????????????????????????????????????????????

????????????????????????????????????????????????????????????

????????????????????????????????????????????????????????????

????????????????????????????????????????????????????????????

????????????????????????????????????????????????????????????

????????????????????????????????????????????????????????????

????????????????????????????????????????????????????????????

????????????????????????????????????????????????????????????

?????????????????????????????????????

>ET880332

TAACGGCCGCGGTATAATGACCGTGCAAAGGTAGCGTAATCACTTGTCTTTTAAATAAAG

ACCCGTATGAATGGCAAGACGAAAGTTCAACTGTCTCTTTGATCTAATCAATGAAATTTA

TCTTCCCGTGCAGAAGCGGAAATATGCCTATAAGACGAGAAGACCCTATGGAGCTTTAAA

TATATAATAATTAAAATTTTGGGTTGGGGCGACCACGGAAGACAGAGATCATCCGAGATG

AGAAAGACACTTCAAAATCTAAAACATTTTGATCCACTAAGTGACCAACGAACCAAGTTA

CCCTAGGGATAACAGCGCAATCCTTTCCAAGAGTCCATATCGACGAATGGGTTTACGACC

TCGATGTTGGATCAGGACACCCAAATGGTGCAGCCGCTATTAAGGTTCGTTTGTTCAACG

ATTAAAGTCCTACATGAATCCATATGCAATATCAATTATACTTTCTAGCCTAGCTATTGG

AACCATTACAACACTCTCAAGCTCACATTGATTCTTGGCATGGATAGGATTAGAAATTAA

TACACTAGCAATTATACCAATAATAACAAAAACCAATCACCCACGCGCAACAGAATCAGC

AACTAAATATTTTCTTACACAAGCAACAGCATCAGCTATCATTTTATTCTCCACAATCAC

AAACATATGAATAACAGGGGAATGAACAATTATTGGTATATCAGATAACATGTCAATAAC

ATTATTAACTATTGCCCTAGCAATAAAACTAGGAATTGCACCATTCCATATATGAATTCC

AGACGTACTACAAGGCTTAAACCTCACCACATGCCTAATCCTATCAACATGACAAAAACT

AGCCCCAATAGCACTGCTTATCTTAACAAACAACCATCTTAATACAAACCTTCTTATTAT

AATAGCACTGGCATCAACAATTATTGGCGGATTTGGAGGCCTAAATCAAACACAAATACG

TAAAATCATAGCATACTCATCAATTGCCCACCTAGGATGAATAACTCTAATCCTATCATT

CTCACCAGACTTAGCTATTCTAAACCTTTGTATTTACCTAATCCTTACAACATCAGCTTT

TATAATAATATATTTTTTAAAATCTACAAATATTAATAAACTATCAATCTCATGATTTAA

GTCCCCAACACTAGCATCATCCATAATAATTACGCTTATAGCATTAGGAGGATTACCTCC

AACACTCGGATTTTTACCAAAATGACTTATTCTTCAAGAAACAACAAAACAACATATAGA

GATAGTATCTTTTATTATTGCTATATCAGCCCTACTTAGCCTTTTTTTTTACCTACGAAT

TTCTTATACAATCTCAATAACAACTTCACCAAACATGGCAAATTCTAACCTAAACTGACG

AATAAAAAAAACCACTAATATTACTATAGCAGTATGCACTACATTAAGTACACTAATACT

ACCAATATCCCCAATACTACTTACAATC????????????????????????????????

????????????????????????????????????????????????????????????

????????????????????????????????????????????????????????????

????????????????????????????????????????????????????????????

????????????????????????????????????????????????????????????

????????????????????????????????????????????????????????????

????????????????????????????????????????????????????????????

????????????????????????????????????????????????????????????

????????????????????????????????????????????????????????????

????????????????????????????????????????????????????????????

????????????????????????????????????????????????????????????

????????????????????????????????????????????????????????????

????????????????????????????????????????????????????????????

????????????????????????????????????????????????????????????

????????????????????????????????????????????????????????????

????????????????????????????????????????????????????????????

????????????????????????????????????????????????????????????

????????????????????????????????????????????????????????????

????????????????????????????????????????????????????????????

?????????????????????????????????????

>ET880308

TAACGGCCGCGGTATCATGACCGTGCAAAGGTAGCGTAATCACTTGTCTTTTAAATAAAG

ACCTGTATGAATGGCAAAACGAAAGTTCAACTGTCTCTATGATCCAATCAATAAAATTAA

TTTTCCCGTGCAGAAGCGGAAATAAATTTATAAGACGAGAAGACCCTATGGAGCTTTAAA

TATATACTAGTTAGAATTTTGGGTTGGGGCGACCACGGAGAAAACTTATCCTCCGAGATA

AGAAAAACACATCTAAAACTAAAATATTTTGATCCACTAAGTGATCAACGAACCAAGTTA

CCCTAGGGATAACAGCGCAATCCTTTCTAAGAGTCCTTATCGACGAATGGGTTTACGACC

TCGATGTTGGATCAGGACACCCCAATGGTGCAGCCGCTATTAAGGCTCGTTTGTTCAACG

ATTAAAGTCCTACATGAGCCCTTATGCATTATCTATCCTCCTATCAAGTCTAGCAATTGG

TACTATTACTACAATATCAAGCTCTCATTGATTTCTTGCATGAATAGGATTAGAAATTAA

CACACTCGCAATCATTCCTTTAATAACAAAAATACACCACCCACGAGCAACAGAATCTGC

AACAAAATATTTCCTCACACAAGCCGCTGCCTCCACCCTAATCCTCTTTTCAACAGTTAT

TAATGCATGATTAATAGGGGAATGAATAATAAATAATTTACAT???TATATTCCCTCAAC

AATATTAACTATTGCCTTAGCTATAAAACTAGGAATTGCACCATTCCATATATGATTACC

AGATGTACTCCAGGGTTTAAACCTAACAACAGGACTTATCCTCTCAACATGACAAAAACT

AGCCCCACTAGCTCTTCTAATTATAACCTATTCTCAACTAAACCCTAACTTAATTATTAT

TATAGCAGTATTATCCATGATCATTGGAGGATGAGGAGGGCTAAACCAAACACAAATACG

AAAAATTATAGCATATTCTTCAATTGCCCACCTAGGCTGAATAATACTAATTTTACCATT

TTCTCCTCAATTAACTATTATAAATTTATTAATTTACTTAACTTTAACCACCTCTATATT

TTTAATAATAATTAATTTGAACTCAACAAACATTAATAAATTATCAATTTCATGATTAAA

AACACCAATTATTTCAGCCTCTATAATAATATCCCTTATAGCCCTGGGAGGACTTCCCCC

AACCTCAGGATTCCTCCCCAAATGACTAATTTTACAAGAAATAACAAAACAACATATAGA

AACACTATCGACTATTACAGCTATAACTGCTCTATTGAGCCTGTTTTTTTACCTCCGATT

AACCTATATAATCTCACTTACAACCGCACCAAACATTACCAACTCTAATATCACATGACG

AACAATATTAAAACCATTAGTTATTTTACCTACTATTATCATT???TCTATTATGTTAAT

ACCTGTTACACCTACAGTATTAACAATA????????????????????????????????

????????????????????????????????????????????????????????????

????????????????????????????????????????????????????????????

????????????????????????????????????????????????????????????

????????????????????????????????????????????????????????????

????????????????????????????????????????????????????????????

????????????????????????????????????????????????????????????

????????????????????????????????????????????????????????????

????????????????????????????????????????????????????????????

????????????????????????????????????????????????????????????

????????????????????????????????????????????????????????????

????????????????????????????????????????????????????????????

????????????????????????????????????????????????????????????

????????????????????????????????????????????????????????????

????????????????????????????????????????????????????????????

????????????????????????????????????????????????????????????

????????????????????????????????????????????????????????????

????????????????????????????????????????????????????????????

????????????????????????????????????????????????????????????

?????????????????????????????????????

>AF154053

CAACGGCCGCGGTATTATGACCGTGCAAAGGTAGCGTAATCACTTGTCTTTTAAATGAAG

ACCTGTATGAATGGCATAACGAAAGTTCAACTGTCTCTTTAATCCTATCAATAAAATTTA

TCTTTCCGTGCAGAAGCGGAAATAAGCCTATAAGACGAGAAGACCCTATGGAGCTTTAAA

CATAAAATAATTTGAGTTTTGGGTTGGGGCGGCCACGGAAGAAAGAAATCCTCCGAGATA

AGAACCACATTTCAAAAACTAAGACATTTTGATCCACTAAGTGACCAACGAACCAAGTTA

CCCTAGGGATAACAGCGCAATCCTTTCTAAGAGTTCCTATCGACGAATGGGTTTACGACC

TCGATGTTGGATCAGGACACCCAAATGGTGTAGCCGCTATTAAGGTTCGTTTGTTCAACG

ATTAAAGTCCTACATGAGCCCATACGTGTTATCAGTGCTTATATCGAGCCTAGCAATCGG

AACCATCGCCACACTATCAAGCACCCATTGATTTCTGGCATGAATAGGACTAGAGATTAA

TACACTTGCAATTATTCCACTAATAACCAAAACACACCACCCACGAGGAACAGAATCAGC

AACAAAGTATTTTCTAACACAAGCCACTGCATCAGGACTAATCCTATTTTCAACTACTAT

TAACGCATGAGCCTTAGGAGAGTGAACAGTAACTAACCTAAAC???CCTACATCATCAAC

AATCTTAACGATCGCCTTGGCAATAAAACTAGGCATTGCACCATTTCATTTATGATTACC

AGACGTAATACAAGGACTAAACCTGATAACAGGCTTAATTTTATCAACATGACAAAAACT

AGCCCCAATAGCCTTACTTATCATAATTAATCACCAATTAAACTCTAACCTACTAATATT

AATAGCTATCTTATCAACAGTTGTTGGGGGATGGGGAGGTCTTAATCAGGTCCAAATGCG

AAAAATTATAGCGTACTCCTCAATTGCTCACCTAGGATGAATAATCATAATTCTAACTTT

TTCTCCGACCTTAACCGTTTTAAATCTTTTAATTTATATTATTCTAACCACCTCAATATT

TATAATAATAATTAACCTTTTCTCAACTAATATCAACAAGTTGGCAGTTTCCTGATCAAA

AACACCAACTCTCTCAGCACTTATAATAATCACCCTTATAGCCCTGGGAGGTCTACCTCC

AACCTCTGGATTTTTACCAAAATGATTAATTCTTCAAGAAATAACTAATCAACATATAAG

CTTAATATCTACAATCATAGCCATATCAACCCTACTAAGCCTATTCTTCTACCTACGACT

ATCATATGTAGTTTCACTAACAACTGCCCCAAGTATCTCAAATTCTAACCTAATATGACG

AGCACAGTTAAAGCCTATAACAGTTATACCCGTAATTATAATTCTCTCATTGATACTAAT

ACCAATTACCCCTATTCTACTAACAATT????????????????????????????????

????????????????????????????????????????????????????????????

????????????????????????????????????????????????????????????

????????????????????????????????????????????????????????????

????????????????????????????????????????????????????????????

????????????????????????????????????????????????????????????

????????????????????????????????????????????????????????????

????????????????????????????????????????????????????????????

????????????????????????????????????????????????????????????

????????????????????????????????????????????????????????????

????????????????????????????????????????????????????????????

????????????????????????????????????????????????????????????

????????????????????????????????????????????????????????????

????????????????????????????????????????????????????????????

????????????????????????????????????????????????????????????

????????????????????????????????????????????????????????????

????????????????????????????????????????????????????????????

????????????????????????????????????????????????????????????

????????????????????????????????????????????????????????????

?????????????????????????????????????

>ET880331

CAACGGCCGCGGTATTATGACCGTGCAAAGGTAGCGTAATCACTTGTCTTTTAAATAAAG

ACCTGTATGAATGGCAAAACGAAAGTTCAACTGTCTCTTTAATCCTATCAATAAAATTAA

TTTTTCCGTGCAGAAGCGGAAATAAATCTATAAGACGAGAAGACCCTATGGAGCTTAAAA

CATAAAATAATTTGAGTTTTAGGTTGGGGCGACCACGGAAGAAAGAAATCCTCCGAGATA

AGAAATACATTTCGAAAACTAAGACATTTTGATCCATTAAGTGACCAACGAACCAAGTTA

CCCTAGGGATAACAGCGCAATCCTTTCTAAGAGTTCATATCGACGAATGGGTTTACGACC

TCGATGTTGGATCAGGACATCCCAATGGTGTAGCCGCTATTAAGGTTCGTTTGTTCAACG

ATTAAAGTCCTACATGAGCCCATATGCATTATCAGTACTTATATCGAGCCTGGCAATTGG

AACAATCACCACATTATCAAGTACTCACTGATTCCTGGCGTGGATAGGATTAGAAATTAA

TACATTAGCAATTATCCCCTTAATAACTAAAATACACCACCCACGAGCAACAGAATCCGC

AACAAAATATTTTCTTACACAAGCTACTGCCTCCGCTCTAATCTTATTCTCAACTACCAT

TAATGCATGAGTTTTAGGAGAGTGGACAATTACTAATTTAAAC???CCAATATCCTCAAC

AATCTTAACAATTGCCCTAGCAATAAAACTAGGAATTGCACCATTTCATTTATGACTGCC

AGATGTACTTCAAGGATTAAATTTAATAACAGGCTTAATTTTATCAACATGACAAAAACT

AGCCCCAATAGCTCTACTTATTATAATTAATCAACAACTTAACTCTAGCCTACTGGTAAT

GATAGCCATCTTATCAACAATTGTGGGCGGATGGGGGGGCCTAAACCAAATCCAAATGCG

AAAAATCATAGCGTTCTCCTCAATTGCCCACTTAGGATGAATAATTCTGATTTTAACTTT

CTCCCCCACTTTAACCATTTTAAACCTTATAATTTATATTCTCCTTACCACCTCTATATT

TATAATAATAATTAATCTTTACTCAACAAACATTAATAAATTGGCAACCTCCTGATTAAA

AACACCAACCCTTTCCGCATCAATGATAATTACACTTATAGCCCTTGGAGGCCTCCCCCC

CCACTCAGGATTTTTACCAAAATGACTTATTCTTCAAGAAATAACAAAACAACACATAGG

TTTAATTTCAACAATCATAGCTATATCGGCTTTATTAAGCCTATTCTTCTACCTCCGACT

ATCATATTCCATCTCTCTGACTACCGCTCCAAACATCTCAAACTCTAACCTAATGTGACG

AACGCAATTGAAGCCCTTAATAATTCTACCTGCTACTATCGTCCTATCCTTATTACTAAT

ACCAGTTACCCCACTCTTGCTAACAACA????????????????????????????????

????????????????????????????????????????????????????????????

????????????????????????????????????????????????????????????

????????????????????????????????????????????????????????????

????????????????????????????????????????????????????????????

????????????????????????????????????????????????????????????

????????????????????????????????????????????????????????????

????????????????????????????????????????????????????????????

????????????????????????????????????????????????????????????

????????????????????????????????????????????????????????????

????????????????????????????????????????????????????????????

????????????????????????????????????????????????????????????

????????????????????????????????????????????????????????????

????????????????????????????????????????????????????????????

????????????????????????????????????????????????????????????

????????????????????????????????????????????????????????????

????????????????????????????????????????????????????????????

????????????????????????????????????????????????????????????

????????????????????????????????????????????????????????????

?????????????????????????????????????

>AY659991

TAACGGCCGCGGTATCATGACCGTGCAAAGGTAGCGTAATCACCTGTCTTTTAATTAAAG

ACCTGTATGAATGGCAAAACGAGAGTCTAACTGTCTCTTTAAATAAATCAGTGAAACTAA

TCTCCCCGTGCAGAAGCGGGAATGATACCATAAGACGAGAAGACCCTATGGAGCTTTAAA

TTTTACTTAATAAAAATTTTAGGTTGGGGCGACCACGGAATAAGAAAAACTTCCGAGATG

AGAAGGACACTTCACAAAATAGAAAAATCTGACCCAATAATTGATCAACGAACCAAGTTA

CCCTAGGGATAACAGCGCAATCTTCTCCAAGAGTTCTTATCGACGAGTAGGTTTACGACC

TCGATGTTGGATCAGGACACCCAAATGGTGCAGCAGCTATTAAGGTTCGTTTGTTCAACG

ATTAAAGTCCTACATGAGCCCATATGCATTATCTATTATTATTTCAAGCTTAGCAACAGG

AACCATATTAACACTAGCTAGCAATCATTGATTTATAGCATGAATAGGATTAGAATTAAA

TACATTAGCTATTATTCCCTTAATAACAAAAACCCACCACCCACGAGCAACAGAAGCAGC

TACGAAATATTTTTTAATACAAGCACTAGCTTCAGCAATAATCTTATTTTCATCAACAAT

AAATGCATGGTTTATAGGGGAATGAGAAATTACTAATATATCACACCCAATTTCAACCAC

TATACTTACAATCGGACTAGCAATAAAACTAGGAATTGCACCGTTCCATATATGATTCCC

TGATGTTTTACAAGGATTAAATCTACTTACATGTTTAATTTTATCAACATGACAAAAAAT

TGCACCAATAATTCTTATAATTCAAATTTATCCACAATTAAACACAAACTTATTAATTGT

AATAGCTATTTTATCCACCACTATTGGAGGATGAGGAGGACTAAATCAAACCCAACTCCG

AAAAATTATAGCTTATTCATCAATTGCACATCTTGGTTGAATAACATTAGTCTTATGTTT

TATACCTTCCTTAACCCTACTAAACCTAGCTGTATATATAACAATAACTACCGTAATATT

TTTAATATTTATGAATATAATATCAACTACTATTAATAAAATAGCTATATCATGATTAAA

GAACCCAGTAATAGCTGCATCAATAATAATTGTATTAATATCATTAGGCGGTCTCCCACC

AACAACCGGATTTATACCAAAATGATTAATTATTCAAGAAATGACTAAACAAAATTTAAT

TGCTATTACAACAATTATTGCTCTATCATCACTATTAAGCTTATTTTTTTATCTTCGAAT

ATCATATTCAATCTCTCTAACAACTTCCCCTAATATCTCAAACACTTTCTCAATTTGACG

ACAAAATAATAAAAATCAAATATTTCTATCAATAACAATTATCCTATCTACATTAATACT

TCCGATTACCCCAACATTAATTAATATT????????????????????????????????

????????????????????????????????????????????????????????????

????????????????????????????????????????????????????????????

????????????????????????????????????????????????????????????

????????????????????????????????????????????????????????????

????????????????????????????????????????????????????????????

????????????????????????????????????????????????????????????

????????????????????????????????????????????????????????????

????????????????????????????????????????????????????????????

????????????????????????????????????????????????????????????

????????????????????????????????????????????????????????????

????????????????????????????????????????????????????????????

????????????????????????????????????????????????????????????

????????????????????????????????????????????????????????????

????????????????????????????????????????????????????????????

????????????????????????????????????????????????????????????

????????????????????????????????????????????????????????????

????????????????????????????????????????????????????????????

????????????????????????????????????????????????????????????

?????????????????????????????????????
